# Exploring the Potential Energy Surface of Pt_6_ Sub-Nano Clusters Deposited over Graphene

**DOI:** 10.3390/ijms24010870

**Published:** 2023-01-03

**Authors:** Daniel Barrena-Espés, Sergio Boneta, Victor Polo, Julen Munárriz

**Affiliations:** 1 Departamento de Química Física y Analítica, Universidad de Oviedo, 33006 Oviedo, Spain; 2 Departamento de Bioquímica y Biología Molecular y Celular, Facultad de Ciencias, Universidad de Zaragoza, 50009 Zaragoza, Spain; 3 Instituto de Biocomputación y Física de Sistemas Complejos (BIFI), Universidad de Zaragoza, 50009 Zaragoza, Spain; 4 Departamento de Química Física, Universidad de Zaragoza, 50009 Zaragoza, Spain

**Keywords:** sub-nano clusters, global optimization, catalysis, DFT, dispersion interactions, ensemble effects

## Abstract

Catalytic systems based on sub-nanoclusters deposited over different supports are promising for very relevant chemical transformations such as many electrocatalytic processes as the ORR. These systems have been demonstrated to be very fluxional, as they are able to change shape and interconvert between each other either alone or in the presence of adsorbates. In addition, an accurate representation of their catalytic activity requires the consideration of ensemble effects and not a single structure alone. In this sense, a reliable theoretical methodology should assure an accurate and extensive exploration of the potential energy surface to include all the relevant structures and with correct relative energies. In this context, we applied DFT in conjunction with global optimization techniques to obtain and analyze the characteristics of the many local minima of Pt_6_ sub-nanoclusters over a carbon-based support (graphene)—a system with electrocatalytic relevance. We also analyzed the magnetism and the charge transfer between the clusters and the support and paid special attention to the dependence of dispersion effects on the ensemble characteristics. We found that the ensembles computed with and without dispersion corrections are qualitatively similar, especially for the lowest-in-energy clusters, which we attribute to a (mainly) covalent binding to the surface. However, there are some significant variations in the relative stability of some clusters, which would significantly affect their population in the ensemble composition.

## 1. Introduction

In the last years, supported noble metal sub-nanoclusters (SNC) have proven to be promising heterogeneous catalysts due to their high surface area, which allows to reduce the amount of catalyst loading, and their ability to activate inert chemical bonds [1,2,3]. Hence, a wide range of catalytic processes involving SNC have been reported in the literature [4,5]. For example, Pt SNC are being investigated as catalysts for technologically important processes, such as the oxygen reduction reaction (ORR) [6,7,8,9]. This way, a detailed theoretical understanding of the structure and properties of these Pt-based SNC is required.

Realistic investigations on the electronic structure of Pt SNC should consider its fluxional behavior [10,11] as ensemble effects are of the utmost importance in SNC-catalyzed processes, and the catalytically most active species is likely to be a structure different from the global minimum (GM) [12]. In addition, the support plays an important role in the system activity, as metal–metal and metal–support interactions lead to complex potential energy surfaces (PES). In this regard, we note that there are some reports on the interaction between Pt-clusters and carbon-based supports that reveal that the cluster size plays an important role on the nature of the interaction [13,14,15,16,17]. For example, based on energy decomposition analysis and changes in the electron density upon adsorption of Pt SNC on a graphene model, it was found that smaller clusters are prone to bond covalently, while in larger structures, van der Waals interactions are predominant [16]. Moreover, some authors have reported that there is a charge transfer from the metallic cluster to the support [18,19,20]. Besides, the Pt–C interaction is significantly affected by the support curvature of the carbon support, which was used by Yang et al. to strengthen adsorption of methanol onto Pt_7_ SNC [15].

Therefore, a thorough and accurate sampling of the PES is required to faithfully model a realistic ensemble. In this context, we performed a detailed analysis of Pt_6_ SNC supported on graphene by using density functional theory (DFT) combined with global optimization (GO) techniques. Moreover, special attention was paid to analyzing dispersion effects on the cluster ensemble composition, which was made by comparison between results performed with and without dispersion corrections. We chose a catalytically relevant system, as Pt_6_ clusters deposited over carbon-based supports have proven to be active in such important processes as ORR [20] and hydrogen electro-oxidation [21]. This system has been previously studied by other authors: Nakajima and co-workers studied Pt_6_ clusters deposited over graphene by pre-optimizing the clusters in the gas phase and then depositing them in the surface [20]; a similar strategy (combined with molecular dynamics simulations) was applied by Da Silva et al., but they found a different geometry for the GM [22]. In addition, to the best of our knowledge, complete GO in which clusters are directly generated over the surface, which is required for modelling the PES of high-fluxional SNC [11], has not been performed yet. Note that, as previously introduced, SNC exhibit a remarkable ability to change shapes and re-adapt their structure in the presence of adsorbates and/or a support [23], and thus, for an accurate exploration of the PES, it is mandatory to generate (hundreds of) the initial structures directly over the support to avoid (or at least mitigate) incomplete sampling effects derived from depositing previously optimized SNC on it.

We note that while a detailed knowledge on the low-lying energy structures of interfaces decorated with Pt_6_ SNC is required for an accurate representation and understanding of the system, modelling electrocatalytic systems is a very difficult task. As a consequence, a variety of novel computational methodologies has been proposed in the last years [24,25,26,27,28,29,30], and other different effects are also expected to influence the ensemble composition, such as the applied potential [31,32] or the experimental conditions (such as pH) [33,34], whose investigation is out of the scope of this contribution.

## 2. Results and Discussion

We first performed a GO for Pt_6_ sub-nano clusters deposited over a graphene layer by means of PBE exchange-correlation functional, including a D3BJ scheme for accounting for dispersion interactions [35,36]. During geometry optimizations, we allowed a free relaxation of the magnetic structure of the system, which was further refined by means of single-point calculations by using the tetrahedron method with Blöchl corrections (see Computational Details section). The main motivation for such procedure was obtaining more accurate relative energies, which will affect the ensemble composition as well as magnetic states. In this regard, some reports have put forward the relevance of magnetic interactions within the catalysts (and between the catalysts and reactants) in their activity [37,38,39,40,41,42,43].

**Figure 1:**
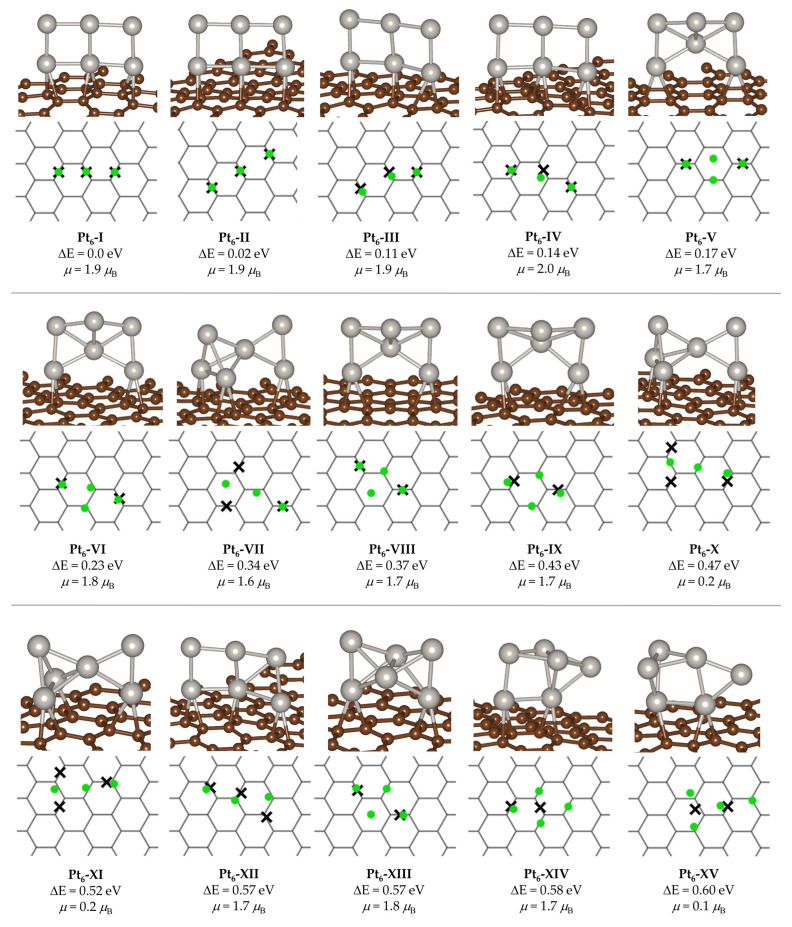
Frontal and schematic top views for the fifteen lowest-in-energy local minimum for Pt_6_/graphene optimized with dispersion-corrected DFT. ΔE corresponds to the energy difference with respect to the GM; the total magnetic moment of the system (per unit cell) is provided in Bohr magnetons. The green dots correspond to the Pt atoms at the top, while the black crosses correspond to the atoms at the bottom.

There were 123 structures within a relative energy cutoff of 1.0 eV, out of which the 15 most stable systems are provided in Figure 1 (the coordinates of the 50 most stable structures are provided in the Supplementary Materials). The various structures are named as Pt_6_-#, where # corresponds to the order of the structure in increasing energy relative to the GM (Pt_6_-I).

As shown in Figure 1, the GM corresponds to a double-square-shaped structure that interacts with the graphene support by means of a bridge coordination to C–C bonds (i.e., Pt atoms lie in the center of the bond). Such kind of Pt–C interaction has been reported to be the most favorable one for the interaction between Pt atoms and graphene-like surfaces [18,22,32,44], which agrees with our observations. On the contrary, to the best of our knowledge, the GM shape had not been previously proposed by other authors but has been reported as a low-lying structure for the gas-phase system, with an energy difference from the GM that ranges between 0.034 eV [22] and ~0.3 eV [45]. Nakajima et al. obtained a GM in which Pt interacts with the support by means of two Pt atoms, forming a square Pt_4_-core that was completed by two bridge Pt atoms that formed a triangular-like geometry with two different Pt–Pt bonds although they then matched the catalytically relevant active species to a different structure by means of spectroscopy measurements (see ref. [20]). The GM obtained by Da Silva and co-workers is related to the former one [22], but the structure exhibits a more planar character, being closer to a triangle-like geometry that has been reported as the GM for gas-phase Pt_6_ clusters by several authors by means of pure functionals [20,45,46,47,48]. However, we did not find any structure directly derived from the aforementioned triangular gas-phase cluster in the set of low energy structures, with the partial exception of Pt_6_-V (and related structures such as Pt_6_-VI, Pt_6_-VIII, and Pt_6_-IX), which resembles a distorted Pt_6_-triangle, in which the central Pt of a hypothetical three-atom base is displaced upwards, and the apical Pt atom bulges. In order to rule out that the difference is due to an incomplete PES sampling, we searched for similar structures to that reported by the aforementioned authors in the whole set of local minima we obtained (a total of 244 structures). We found relative energies of 0.88 eV and 1.60 eV for two structures that are very related to the GM as proposed by da Silva [22], as they correspond to Pt_6_-triangles that interact with the surface by two of the three Pt atoms that form the triangle base. Note that the energy difference between them is due to a different pattern of interaction with the support and structural distortions in the Pt_6_ core. The geometries of these structures, which are identified as *Pt6-min da Silva* and *Pt6-min’ da Silva*, are provided in the Supplementary Materials (Table S2). We also found a relative energy of 1.04 eV for the following structure in energy ordering (or a closely related structure) reported by the same authors (identified as *Pt6-min2 da Silva* in Table S2), which corresponds to a Pt_6_-triangle that interacts with the support by means of three Pt atoms. Moreover, we obtained a structure that corresponds to a flat Pt_6_-traingle that interacts with the support by means of van der Waals interactions at a distance of the surface of about 3.1 Å. Such structure presents a relative energy of 1.15 eV, with its geometry provided in Table S2 (*Pt6-min6 da Silva*). When comparing our local minima with those reported by Nakajima and co-workers, we found that their GM is closely related to a structure that, in our set, has a relative energy of 0.87 eV (Table S2, *Pt6-min Nakajima*) [20]. They also proposed a low-energy structure with triangular, prismatic shape for which we obtained a relative energy of 0.92 eV (Table S2, *Pt6-str7 Nakajima*) and the aforementioned van der Waals structure although with a relative energy significantly higher than da Silva: 0.433 eV vs. 0.0668 eV, respectively, while in our case, it was 1.15 eV, and the structure is not exactly the same, as it is partially displaced with respect to the support. Overall, these differences put forward the extreme sensitivity of these systems to the computational approach, including both DFT calculations and PES sampling procedure.

If we turn back to our ensemble (Figure 1), we can see that the four most stable structures, Pt_6_-I to Pt_6_-IV, with a maximum energy difference between them of 0.14 eV, exhibit a closely related shape. Both Pt_6_-I and Pt_6_-II show a planar double-square shape, which only differs on the interaction mode with the graphene sheet (see top view of Figure 1). Namely, while Pt_6_-I binds to C–C bonds of the support in a zig-zag manner, Pt_6_-II binds to parallel C–C bonds, with the energy difference between both structures being only 0.02 eV. The geometrical structure of Pt_6_-III and Pt_6_-IV consists of two Pt_4_ squares that are joined by a Pt–Pt bond, having a shape that resembles a hinge, and as for the previous couple of structures, the main difference between both minima (which translates into an energy difference of only 0.03 eV) is due to the different orientation with respect to the support (top view of Figure 1). The following local minima by relative energy ordering are Pt_6_-V, (ΔE = 0.17 eV). This system exhibits a significantly different shape, whose geometry (which has already been introduced) might be described as a core of 5 Pt atoms forming two triangles that are joined together by means of a central atom—which has been reported by some of us as a relevant local minimum for Pt_5_/graphite [32]—and an additional Pt atom on the top that forms an angle that breaks the planarity of the system. As for the previous SNC, Pt_6_-V interacts with C–C bonds in a bridge fashion, while in this case, it is bonded to the support by two Pt atoms (instead of three). Note that this structure is intimately related to Pt_6_-VI, Pt_6_-VIII, and Pt_6_-IX, which present essentially the same geometrical structure but show a different interaction pattern with the support (see Figure 1, top view). This change in the interaction mode translates into significant energy differences in the relative energy, which is, for example, 0.2 eV higher for Pt_6_-VIII than for Pt_6-_V.

We do not comment in detail the geometrical structures of all the other local minima shown in Figure 1, but we can see that all of them interact with the C–C bonds of the support in the bridge positions and by two or three Pt atoms, which agrees with reports from other authors [22], and that there are some other structures whose main difference is the interaction pattern with the support (i.e., Pt_6_-VII, Pt_6_-X, and Pt_6_-XI). To our understanding, this result puts forward the relevant role of the support in SNC stability and thus the importance of an exhaustive sampling of the surface.

Furthermore, the most stable structures are associated to highly planar geometries, which is in line with previous observations that more flat structures are favored by pure functionals (such as PBE), while hybrid ones favor more globular geometries [45,49].

With respect to magnetic states, most of the clusters have magnetic moments close to 2.0 *μ*_B_. Namely, this is the case for the nine most stable structures (Pt_6_-I to Pt_6_-IX), in line with previous reports for both gas-phase and graphene-supported Pt_6_ clusters [22,45]. Moreover, most of the structures reported herein have different magnetic states, which are very close in energy, which is also consistent with previous spin-state analysis of Pt_6_ systems [45,50].

**Figure 2:**
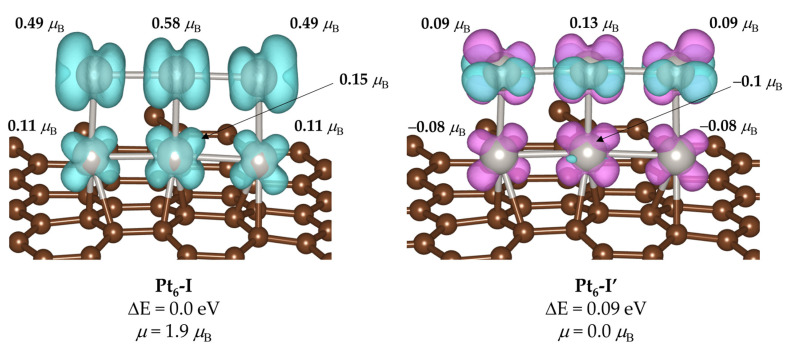
Spin density, total and individual atomic magnetic moments, and energy different for the two low-lying spin states of Pt_6_-I. Note that α spin density is shown in light blue and β spin density in light pink (isovalue = 0.03 au).

In this regard, turning back to the GM, we found that its ground state corresponds to a triplet state with two unpaired electrons overall and a total magnetic moment of 1.9 *μ*_B_. However, there is a non-magnetic state that is only 0.09 eV higher in energy, which we refer to as Pt_6_-I’ (see Figure 2), and would involve that both structures may be accessible even at low temperatures (if we do not consider limitations of forbidden spin crossover effects). Note that while Pt_6_-I corresponds to a magnetic structure with ferromagnetic coupling between all the individual magnetic moments of the various Pt atoms, Pt_6_-I’ exhibits an antiferromagnetic state, in which the Pt atoms directly bonded to the support show negative individual magnetic moments, while those that are only bonded to other Pt atoms have positive magnetic moments. While the absolute values of the magnetism of atoms bonded to graphene have similar magnitudes (about 0.1 *μ*_B_), those of Pt atoms that are not bonded to C differ from 0.49–0.58 *μ*_B_ in Pt_6_-I to 0.09–0.13 *μ*_B_ in Pt_6_-I’. The magnitude of the individual magnetic moments is in the same range as that reported by Kumar and Kawazoe for gas-phase Pt_n_ clusters [48]. We note that a further discussion of magnetic states is out of the scope of this contribution, but overall, these results support the importance of an adequate consideration of the magnetic state of the metal atom, as it is likely to affect the catalysis [38,51].

**Figure 3:**
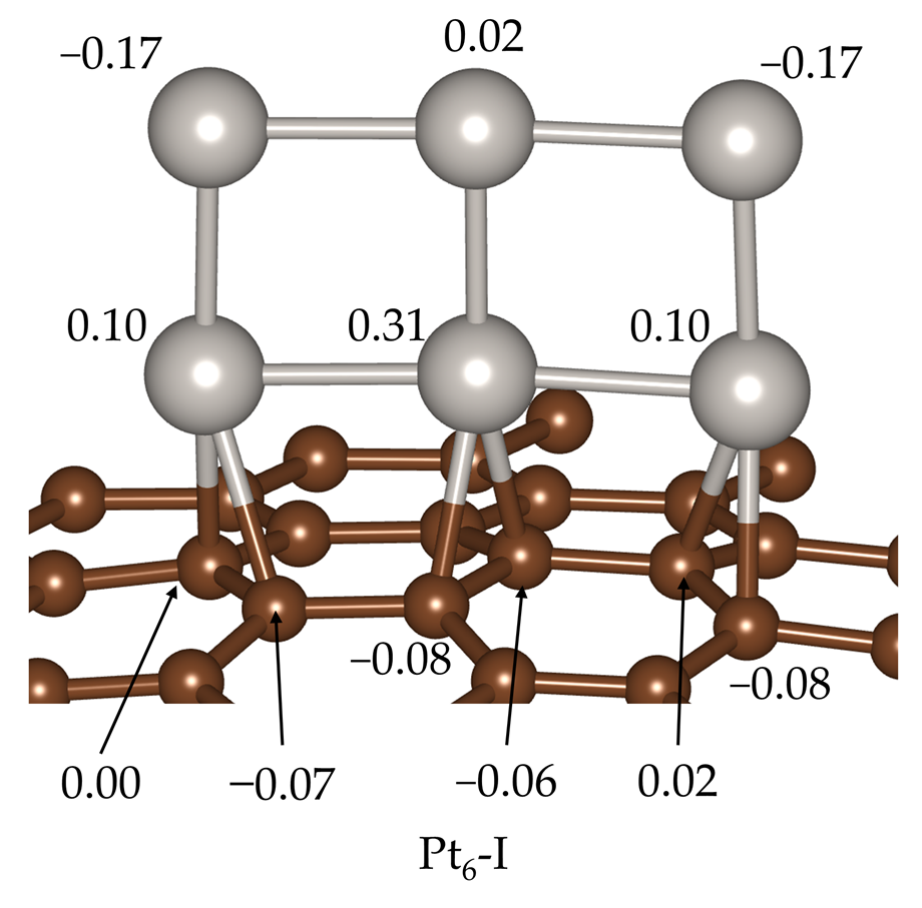
Bader charges in (in au) for relevant atoms of the GM (Pt_6_-I). Positive charges are depicted in.

We then analyzed the direction of charge transference when the cluster binds to the support. For that, we calculated Bader charges, with the results for the GM shown in Figure 3 (see Figure S1 for the charges of selected local minima with significantly different geometrical structure). We can see that the Pt atoms that are directly bonded to the graphene support have positive charges: 0.31 au for the central Pt atom and 0.10 au for Pt atoms at the ends of the cluster, and this result holds for all the analyzed local minima (Figure S1). With respect to the other three Pt atoms, the top central one exhibits a tiny positive charge (0.02 au), while those at the corners are negatively charged (−0.17 au). Our results indicate that the charge transfer takes place from the cluster—which would have an effective charge of 0.19 au (obtained by summing up the individual charges of the six Pt atoms)—to the support. This result agrees with reports from other authors [18,19,20,22].

**Figure 4:**
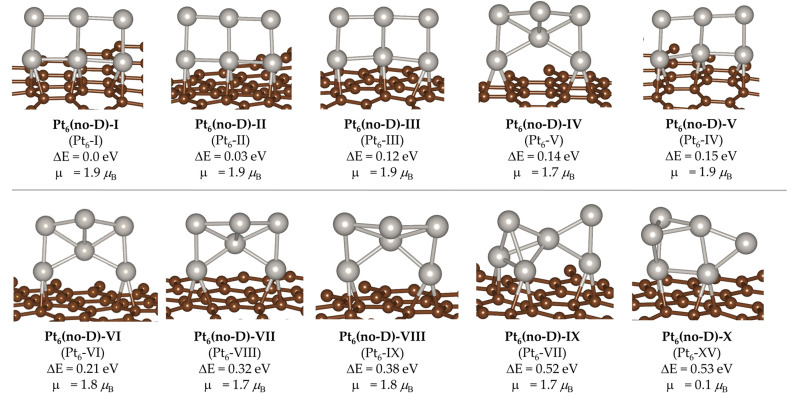
Frontal view for the ten lowest-in-energy local minimum for Pt_6_/graphene optimized without dispersion corrections. ΔE corresponds to the energy difference with respect to the GM; the total magnetic moment of the system (per unit cell) is provided in Bohr magnetons.

Finally, we studied the effect of dispersion corrections in the relative energy and geometry of the clusters. For that, we performed the GO calculations without including dispersion corrections. To differentiate these structures from the dispersion correction-optimized ones, we refer to them as Pt_6_(no-D)-#, where # indicates the structure position in the relative energy ordering. The 10 most stable structures are shown in Figure 4, in which we also include the equivalent dispersion-corrected structure (in parentheses), and in Table 1, in which we also include the energy values for the analogous dispersion-corrected structure. At first glance, we can see that the geometries of the structures obtained with and without dispersion corrections are very similar. However, there are some significant changes in relative stability that lead to variations in the relative order. For example, there is a switch in order between Pt_6_(no-D)-IV and Pt_6_(no-D)-V, which were Pt_6_-V and Pt_6_-IV in the dispersion-corrected ensemble (see Figure 4), although such change only involves a minor relative energy difference of up to 0.03 eV (see Table 1). There is also an order transposition in Pt_6_(no-D)-IX (former Pt_6_-VII), which switches from position 7 to 9. Contrary to the previous case, this structure is significantly more stable (0.18 eV) in the dispersion-corrected scheme although both geometries are very similar. Another relevant position change corresponds to Pt_6_(no-D)-X, which was Pt_6_-XV in the dispersion-corrected scheme, and in this case, the relative energy within the ensemble is 0.07 eV lower in the non-dispersion corrected set of structures.

In order to rationalize this observation, we recurred to the dispersion energy term obtained from D3BJ scheme (E_disp_ in Table 1). We see that most values are relatively similar, about −7.92 eV. However, for Pt_6_-VI,I it is −8.115 eV, which explains the significant destabilization when excluding such correction. The opposite trend is also observed, as, for example, structures Pt_6_(no-D)-VII, VIII, and X (Pt_6_-VIII, IX, and XV in the dispersion-corrected set, respectively) are comparatively stabilized (by 0.05–0.07 eV, see Table 1) in the non-dispersion ensemble, which correlates with the lower weight of dispersion correction (−7.87 eV on average).

Although overall our findings agree with those reported in ref. [22], in which the authors observed that dispersion correction effects barely affect the cluster structure, we found that, for some cases, dispersion corrections are important in providing correct relative energies (and thus cluster populations), as some structures are significantly more affected than others, and this effect might be relevant in the catalytic activity.

As previously introduced, the interaction of small clusters with carbon-based surfaces has previously been attributed to predominant covalent interactions with the support [16]. In qualitative terms, this result correlates with our findings, as the most stable structures obtained by means of dispersion and non-dispersion-corrected procedures show very similar geometries, and we did not find any low-lying structure bonded to the support in the characteristic parallel manner that would result from predominant van der Waals interactions.

## 3. Computational Details

Spin-polarized density functional theory (DFT) calculations were performed by means of the Vienna Ab initio Simulation Package (VASP), Version 5.4.4 [52,53,54]. We applied the PBE exchange-correlation functional [55] in conjunction with the projector augmented wave (PAW) method [56,57] to represent interactions between core and valence electrons. Clusters were directly generated and optimized over a *p* (6 × 6) graphene surface (*a* and *b* lattice parameters of 14.777 Å) with a vacuum of 18 Å (between graphene layers) to avoid interactions between parallel images. For geometry optimizations, we considered a Gaussian smearing (width 0.1 eV) and a cutoff of 400 eV for plane waves. For the electronic minimization algorithm, we selected the “ALGO = Fast” option, which selects a mixture of the Davidson and RMM-DIIS algorithms, while for geometry relaxation, we selected “IBRION = 2”, which applies a conjugate-gradient algorithm. The convergence criteria for energy calculations was set to 10^−6^ eV (“EDIFF = 1e-06”), as the criteria for the geometry optimization were a difference lower than 10^−5^ eV between two consecutive steps (the default VASP value, which is EDIFF × 10). We also considered a real-space evaluation of projector operators (“LREAL = Auto”). We further performed single-point energy calculations to refine the previous results by using the tetrahedron method with Blöchl corrections, a cutoff of 500 eV and a blocked Davidson algorithm for optimizing the orbitals (“ALGO = Normal”). The Brillouin zone was integrated by a 1 × 1 × 1 K-point mesh for geometry optimizations, which was increased to 5 × 5 × 1 in single-point calculations. Dispersion interactions were accounted for by means of D3BJ scheme developed by Grimme and co-workers [35,36]. In a first step, we considered 250 and 200 initial structures for GO optimization without and with dispersion corrections, respectively. In order to minimize PES incomplete sampling, we also re-optimized the 20 most stable structures of each set with the settings of the other. Then, we added 100 additional structures to make sure that the PES was properly sampled. Structures were generated and filtered thorough PGOPT program suite developed in Alexandrova’s group, which uses a bond length distribution algorithm (BLDA) [58,59].

## 4. Conclusions

The potential energy surface for Pt_6_ sub-nano clusters deposited over a graphene layer was explored using DFT methodology and global optimization techniques. The global minimum energies correspond to structures featuring a planar double-square shape for Pt_6_ atoms interacting with the graphene support by bridge coordination of three Pt atoms to C–C bonds (Pt_6_-I to IV). We also found other low-lying structures with relatively high planarity as well as some others with more prismatic character. In addition, although Pt atoms always interact with the C–C bond by means of a bridge coordination, we observed some different patterns of interaction with the support due to several interaction modes with the graphene support (involving different combinations of C–C bonds). Therefore, an extensive sampling in global optimization techniques is required to accurately sample the PES of graphene-supported Pt_6_ SNC. Analysis of magnetic properties shows that the global minimum (GM) structure features a triplet state with two unpaired electrons and a total magnetic moment of 1.9 *μ*_B_. Inspection of atomic charges of the GM reveals a transfer of electron density from the cluster to the support of 0.19 au. Finally, the effect of dispersion interactions introduced by D3BJ corrections was analyzed by comparison to global optimization performed without dispersion interactions. Although the geometries are not very affected (which we associate to predominant covalent bonding with the support), and the nature of the GM does not change, and the dispersion corrections affect structures in a different manner. While the relative energy does not change much in general, it does for some structures, which would distort ensemble population and puts forward the importance of including dispersion corrections in the calculations.

## Figures and Tables

**Table 1:** Correspondence of the 10 most stable structures obtained from the ensemble without dispersion (Pt_6_(no-D)-#) with those obtained with dispersion corrections (Pt_6_-#’). All energy values are provided in eV.

Pt_6_(no-D)-#	Pt_6_-#’	∆E(Pt_6_(no-D)-#)	∆E(Pt_6_-#’)	∆(∆E)	E_disp_
I	I	0.00	0.00	0.00	−7.920
II	II	0.03	0.02	0.01	−7.930
III	III	0.12	0.11	0.01	−7.928
IV	V	0.14	0.17	−0.03	−7.884
V	IV	0.15	0.14	0.01	−7.936
VI	VI	0.21	0.23	−0.02	−7.900
VII	VIII	0.32	0.37	−0.05	−7.877
VIII	IX	0.38	0.43	−0.05	−7.872
IX	VII	0.52	0.34	0.18	−8.115
X	XV	0.53	0.60	−0.07	−7.858

Note that ∆(∆E) corresponds to the difference between the relative energy of equivalent structures obtained without dispersion and with dispersion: ∆(∆E) = ∆E(Pt_6_(no-D)-#)—∆E(Pt_6_-#’). Thus, a positive value indicates that the structure is comparatively most stable when computed with dispersion corrections, and a negative value indicates that the structure is comparatively less stable without including dispersion. E_disp_ is the dispersion energy term obtained from D3BJ scheme.

## Data Availability

Not applicable.

## Supplementary Materials

The following supporting information can be downloaded at: https://www.mdpi.com/article/10.3390/ijms24010870/s1.
