# Supplementary material for: Exploring the Potential Energy Surface of Pt6 Sub-Nano Clusters Deposited over Graphene
Source: Int J Mol Sci. 2023 Jan 3;24(1):870. doi: 10.3390/ijms24010870 (PMC9820941; doi:10.3390/ijms24010870)
Supplement: Supplementary file 1 [file ijms-24-00870-s001.zip › ijms-2096828-supplementary.pdf]

Supporting Information for Exploring the Potential Energy Surface of Pt6 Sub-Nano Clusters Deposited over Graphene

Daniel Barrena-Espés[1], Sergio Boneta[2,3], Victor Polo[3,4,*] and Julen Munárriz[1]

[1] Departamento de Química Física y Analítica, Universidad de Oviedo, 33006 Oviedo, Spain
[2] Departamento de Bioquímica y Biología Molecular y Celular, Facultad de Ciencias, Universidad de Zaragoza, 50009 Zaragoza, Spain
[3] Instituto de Biocomputación y Física de Sistemas Complejos (BIFI), Universidad de Zaragoza, 50009 Zaragoza, Spain
[4] Departamento de Química Física, Universidad de Zaragoza, 50009 Zaragoza, Spain
***** Correspondence: vipolo@unizar.es

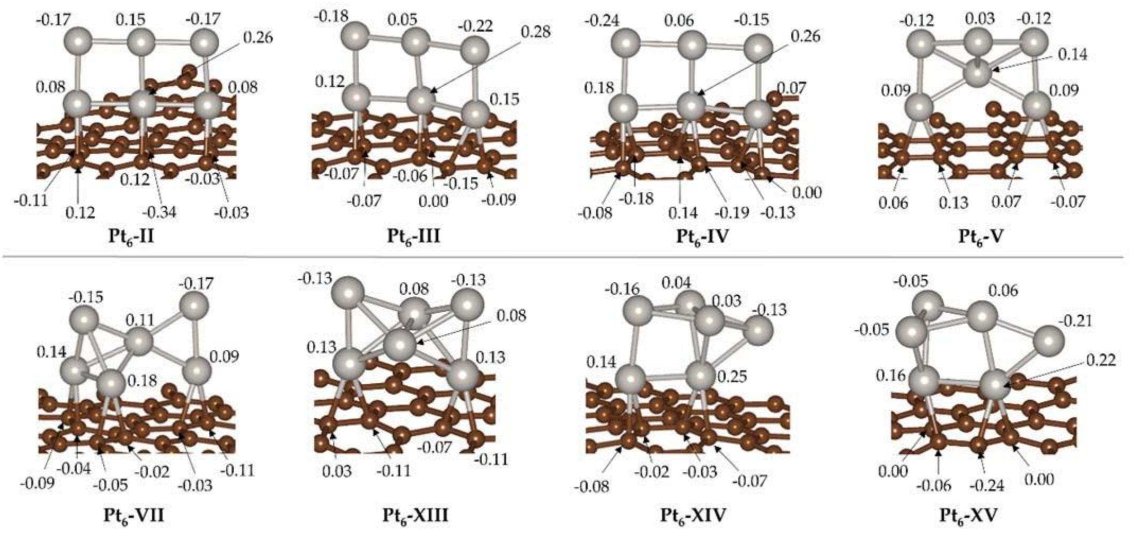

Figure S1. Bader charges (in au) for the 4 most stable structures after the GM (Pt6-II to Pt6-V) and other clusters with differentially distinctive geometrical shapes.

Table S1. Coordinates (VASP CONTCAR format) for the 50 most stable structures optimized with dispersion corrections.

```
PT6-I – ΔE = 0.00 eV
  1.00000000000000
   14.7772044699999991    0.0000000000000000    0.0000000000000000
   -7.3886022400000000   12.7974344700000007    0.0000000000000000
    0.0000000000000000    0.0000000000000000   18.0009597799999987
  C   Pt
   72     6
Direct
  0.2146615745520123  0.2668688035394491  0.9906205502341638
  0.0486574997340821  0.1001414994919608  0.9902846631752432
  0.8815147511944911  0.9328843683266071  0.9956363001337314
  0.0480802721858669  0.2661417797429280  0.9896332120821483
  0.8814986104598084  0.1000849228786921  0.9919601356724408
  0.7159847645940519  0.7685579112294816  0.0077904658378640
  0.5473294324107523  0.5981516379524937  0.0198207895720121
  0.7157117682979500  0.9336247360906945  0.9983328743410186
  0.8820448873443492  0.2666164072419571  0.9907545625330413
  0.7147268462551589  0.0992055964428204  0.9934912930688753
  0.5483819776928281  0.7680152659085343  0.0127518274636439
  0.3811427819393640  0.4325654090631101  0.9984827600601035
  0.3795316029373665  0.5995675943401321  0.0018156454993841
  0.5480869594149864  0.9342044368559643  0.9976658982474635
  0.7149791689310498  0.2665727561168431  0.9932215459101960
  0.2153413433282800  0.4335429169319218  0.9921238203542211
  0.3815618351031410  0.7671218912231950  0.9971568324742041
  0.5489006505705190  0.1004868467675801  0.9927373978991281
  0.5481983860854799  0.2661447627078609  0.9942839222225021
  0.2146208557294381  0.5994800649109541  0.9906321747223783
  0.3814148810165885  0.9331587091765736  0.9922733875275548
  0.3814703794409761  0.1004237513220574  0.9906940051789578
```

0.0481652955498783 0.4334231406543805 0.9893679656595360
0.2146234725559313 0.7668729701269011 0.9900273055416110
0.2154146461125184 0.9337032332929152 0.9897949514924163
0.3818687079946314 0.2668702687206941 0.9924786063278904
0.0488935552705794 0.6001226617144523 0.9894662889816698
0.0482306573566788 0.7663336444366909 0.9910087712151305
0.2147330312686463 0.0996808075953073 0.9896020210370887
0.8816542964120447 0.4327529387949106 0.9921251086867663
0.8823788648578483 0.6005743524050974 0.9956966837594990
0.0481716428102386 0.9336499830721282 0.9914228273716509
0.7161354680813740 0.4325669901584379 0.0008302102530873
0.8826643640656708 0.7669527045826937 0.9991949381167170
0.7175399996396925 0.5993833889849276 0.0163231335936781
0.5465412596454196 0.4322620323880220 0.0021713187817054
0.1590368321961784 0.1552357113021876 0.9896915201488191
0.9930569332186627 0.9885375050348124 0.9920518569255623
0.8265978578159549 0.8228779864699484 0.0009606302060596
0.9930010021600265 0.1557233592254194 0.9903485988821497
0.8259067965247915 0.9885949326963798 0.9950293177583548
0.4917298946222872 0.4868486177053186 0.0051620487323296
0.6611486687863330 0.8231928928097747 0.0053283488234278
0.8263207995166975 0.1550317840674478 0.9918883528545182
0.6596160283051828 0.9893513208033576 0.9966047546103322
0.4899662541278330 0.6557053981713825 0.0167176372318795
0.3262534863063265 0.3225758182307508 0.9932560153228098
0.3250848081381300 0.4884709924811400 0.9974821240018154
0.6589254954179296 0.1549837905322435 0.9928939890981922
0.4916307044263484 0.8229092392165908 0.0025485135857510
0.4928348513573600 0.9889113764790318 0.9938944497649443
0.3254326849339702 0.6549272183468217 0.9954383897078891
0.1595550239607562 0.3217660657932971 0.9903887639014067
0.3261984182363773 0.8227491308175658 0.9924855850096043
0.1595620352135082 0.4891420136916113 0.9902728418292683
0.4930853347420807 0.1562596185160672 0.9921119820714230
0.1589180482454643 0.6553697755315497 0.9894298464278086
0.3257504346620215 0.9888492633051355 0.9904351339203294
0.9923639424412629 0.3217402499358825 0.9897406055317549
0.3262628382332375 0.1553494860126342 0.9903217075328612
0.9931917138782076 0.4886102005519570 0.9898444050390882
0.1596219721020265 0.8220322439394153 0.9897571384876719
0.1596515821963569 0.9892604986516824 0.9897413656327260
0.8264187527077418 0.3223379504226604 0.9920190247194721
0.9932014231509237 0.6560157556248463 0.9910991766304744
0.9923965476612183 0.8218900733000254 0.9934161770353070
0.8263004655865840 0.4884115776011211 0.9965302906473918
0.8281660040010506 0.6557851654711789 0.0026377086493526
0.6596810862664014 0.3212567161813807 0.9958513135467547
0.6591751683932552 0.4873399739950785 0.0104418164587901
0.4920658484374728 0.3218418374658781 0.9952908491158509
0.6602136869693902 0.6568138323843991 0.0205493036390717
0.5072904364905676 0.7253898530226759 0.1299064827003191
0.6860141439973609 0.5165149240270946 0.2663618144836813
0.5933910804315498 0.6149231589593317 0.2695266494743149
0.4987405289470879 0.7113784750523655 0.2676351700075760
0.6014312086798406 0.6240841941565168 0.1338518018567852
0.6941619010980644 0.5214486799357833 0.1284973156058411

PT6-II - ΔE = 0.02 eV
1.00000000000000
14.7772044699999991 0.0000000000000000 0.0000000000000000
-7.3886022400000000 12.7974344700000007 0.0000000000000000
0.0000000000000000 0.0000000000000000 18.0009597799999987
C Pt
72 6
Direct
0.2168671083507263 0.2805280566882402 0.0012750966097101
0.0518104564172575 0.1147561112598794 0.9930166132848939
0.8852611658333345 0.9482062220340737 0.9928321074189768
0.0513804451408788 0.2811735514551970 0.9933336236507060
0.8851270929515849 0.1148885598115044 0.9918450225669631
0.7194689408162489 0.7831318767774604 0.0009421898047108
0.5512266486649651 0.6159598964380635 0.0047898935911590
0.7188017848310295 0.9485866556481852 0.9930871661439653
0.8850032953374125 0.2815246237270799 0.9921165276870809
0.7184516913978314 0.1149714630509706 0.9920696573099406
0.5522656070448662 0.7819275311133183 0.9954077070952323
0.3840116200277777 0.4487340452499922 0.0049482843885755
0.3851610901443507 0.6148466463112925 0.9948312843944720
0.5520880774345045 0.9482289418968861 0.9925402810540334
0.7184571545240530 0.2815238354038314 0.9937868014231839
0.2180587888367214 0.4477510697636333 0.9956338641848532
0.3855060213758463 0.7813557975842826 0.9919535509982182
0.5517685352309059 0.1146629718165926 0.9939287113605957
0.5519475751672900 0.2810316222419331 0.9987489619398318
0.2186547113203758 0.6145049913632690 0.9920051495341511
0.3853783580631074 0.9477847937935451 0.9923132429675618
0.3849739412995277 0.1139603250734851 0.9972758634379915
0.0517554806286498 0.4479050831104061 0.9926450759278538
0.2187923021635143 0.7812108635066153 0.9911712265171175
0.2186619192319199 0.9478060861178790 0.9925436741831746
0.3850328871754485 0.2796211332817720 0.0119100232346696
0.0522290307478741 0.6146330266008917 0.9921956728082932
0.0521983381535094 0.7813349258125371 0.9923869507261074
0.2181835380843893 0.1140081829393083 0.9966292927596641
0.8853371530408509 0.4482323169965383 0.9938404027630057
0.8860123330666738 0.6150070798212042 0.9970308478682242
0.0518829966428882 0.9481286287955157 0.9921415750842399
0.7189831683828203 0.4480586890463556 0.9986289379460729
0.8859801653060160 0.7818110902735924 0.9963410590980146
0.7203892355465911 0.6149806734625898 0.0116451772571011
0.5528779530752530 0.4471190811610413 0.0128103674548896
0.1626929653426217 0.1695049247630678 0.9965185646982491
0.9967910370099702 0.0032177706873711 0.9921388306767369
0.8304856371004234 0.8373062202891646 0.9962189589161738
0.9964629044550577 0.1700098512765322 0.9926010359406234
0.8299872071736303 0.0035435786023044 0.9924300273772957

0.4957589802807618 0.5042200452673455 0.0122196613988166
0.6636446881205202 0.8373165316530091 0.9963759104026266
0.8298742152906229 0.1701228045376482 0.9918220200910497
0.6632705815448787 0.0035397935361203 0.9923131144213855
0.4967535203844093 0.6702482121723250 0.9972268246415865
0.3283160465800137 0.3364333892592626 0.0114461430809385
0.3297540371887990 0.5032445806190324 0.9973743453844648
0.6631719136622678 0.1699795025932644 0.9930667541827276
0.4968918820656043 0.8366349044489709 0.9929502926254443
0.4965568542716454 0.0030751303495151 0.9927905126230598
0.3302195202243823 0.6697741899317009 0.9924425367549077
0.1626614893523097 0.3363378037868117 0.9966852469358969
0.3302075918009919 0.8363368903872441 0.9913574534525011
0.1633539883895523 0.5031199596104301 0.9930918706693035
0.4964172551919077 0.1693393321844923 0.9966681403408160
0.1636615093215568 0.6697825398572235 0.9913473327828086
0.3298242059604206 0.0027066679583996 0.9934671558138888
0.9964449863800908 0.3367185778914674 0.9924555839659277
0.3291885484467372 0.1681755829628244 0.0017057891806047
0.9969276969404319 0.5034400217053161 0.9927441793761034
0.1635506316287589 0.8364541935199057 0.9915626004830358
0.1634195626779089 0.0031472909281050 0.9930518701144422
0.8300335651071649 0.3368272420486633 0.9930719832803803
0.9972775922828276 0.6701448626328030 0.9932711697779197
0.9968552300083076 0.8365743074463836 0.9928886465937410
0.8306557894729991 0.5035945213474804 0.9964827085636898
0.8318164170916091 0.6708098942928729 0.0014159044392557
0.6635545024456491 0.3364673927562407 0.9963370464706927
0.6647107995865014 0.5025799203388033 0.0058369910831644
0.4974122967469725 0.3353091644157971 0.0059460131767963
0.6635625861810723 0.6716958686191035 0.0111993019252310
0.5213928111365504 0.4786296637320362 0.2646280677856510
0.3564867650624421 0.3138346341657225 0.2650460245589343
0.3526954274185101 0.3057302942740847 0.1277384475152061
0.5235898047913992 0.4763979948938228 0.1291426007518055
0.6942109382634314 0.6473145322348728 0.1274849462688863
0.6859843281070326 0.6435679044224685 0.2648171864399984

PT6-III - ΔE = 0.11 eV
1.00000000000000
14.7772044699999991 0.0000000000000000 0.0000000000000000
-7.3886022400000000 12.7974344700000007 0.0000000000000000
0.0000000000000000 0.0000000000000000 18.0009597799999987
C Pt
72 6
Direct
0.8890779133945769 0.6059091903100025 0.9935320985781226
0.0551563623007245 0.6055747688386175 0.9928194212005631
0.2215677621428629 0.6060980951711894 0.9987236310532117
0.8889167439502009 0.4390664292736366 0.9938047452764077
0.0554601086581224 0.4391312772762450 0.9947727689951519
0.3890007739903722 0.6076532792542586 0.0130497978328110
0.5568763258730390 0.6074099546747104 0.0155032309198290
0.2203308396839564 0.4383078665541382 0.0038311463663508
0.8885650177777461 0.2722557332257333 0.9928168962124382
0.0550651780918346 0.2724726259360015 0.9946837340779396
0.3872201963272507 0.4371839280617211 0.0088759672057731
0.7236303692771408 0.6078985291090363 0.0010110732683680
0.5560411584956833 0.4371436930570566 0.0121854771892781
0.2218324330686970 0.2724447003126377 0.9980292727800678
0.8887666406223774 0.1058936644348987 0.9921159440289025
0.7228741855779024 0.4385079057410702 0.0003886220444426
0.3889537681721666 0.2720416891927830 0.9983464172618085
0.0554107116165243 0.1060276819256008 0.9924871386207930
0.8889810507487551 0.9395895837337093 0.9921182646738886
0.5551402479776468 0.2716165897993836 0.9962870681867532
0.2221858476128414 0.1060475781618509 0.9925782736934767
0.0556324741981278 0.9395637988579970 0.9907520877069770
0.7221651285012493 0.2722277030623106 0.9939379854215358
0.3887916581496544 0.1058433077502589 0.9923259915886199
0.2220729275117000 0.9392610936494096 0.9908475061805503
0.8890248088393814 0.7729710465018488 0.9926806528487069
0.5553457559757592 0.1054130333360285 0.9922489594702143
0.3886695735861336 0.9393378828902081 0.9923184189504520
0.0554062949574722 0.7727011963266783 0.9908741333039612
0.7218978792864708 0.1055056999392487 0.9922594742372430
0.5553273946929518 0.9389912197866047 0.9941597211087720
0.2220538797340878 0.7727191871087917 0.9930809499945694
0.7220669949203711 0.9389389054097687 0.9941855048680798
0.3884957389305101 0.7729140532999604 0.9984444055603795
0.5556440875675454 0.7728555054548195 0.0002599874152212
0.7227842805380357 0.7731575239389458 0.9980800916808092
0.9998030835605726 0.6618428192000934 0.9917671913577480
0.1666461440285642 0.6618458708652674 0.9943343925699603
0.3326884763514006 0.6628708086444799 0.0035315386126326
0.0001449351355250 0.4953860857520682 0.9933446092093914
0.1659094109335298 0.4949072398306546 0.9987269174763633
0.6684438779800513 0.6632508432245768 0.0041432101933486
0.3316748844603524 0.4939688158320639 0.0145513703034912
0.9999068079596315 0.3283215683665497 0.9940260963046882
0.1662120842191774 0.3284397224982510 0.9988851349313312
0.4989602616073761 0.4939274487012879 0.0176149096767619
0.8336778976361684 0.6625545514362317 0.9947085129955591
0.6681644197662405 0.4957384683567635 0.0068912980742355
0.9998851868097857 0.1617630551490832 0.9927865662843099
0.3331722741761991 0.3284884754217366 0.0011525174005758
0.1668621784814207 0.1622138023785182 0.9940493562018988
0.4995373933286326 0.3266795167958160 0.0014380824919471
0.8342091992535944 0.4953706056595452 0.9957048568921126
0.3335186623598171 0.1619467149517731 0.9938382407134099
0.6667621429510852 0.3279388287118353 0.9971892904098747
0.0002882763662960 0.9955396892997428 0.9913300121517564
0.4997781099123973 0.1613943444169624 0.9928837659294807
0.1668706668098970 0.9955535310959556 0.9909007035462085
0.8332995134600081 0.3283293084266465 0.9935076679968691
0.0003440385397967 0.8288905290175790 0.9908661929852554

0.6665282310475502 0.1614380525545188 0.9925565220832891
0.3332400331771642 0.9951464679897984 0.9913676789097308
0.1666266122846204 0.8286249858965299 0.9911084268214148
0.8333335196256677 0.1617729686236153 0.9922623605382063
0.4998262625615251 0.9950793763734505 0.9926576589813666
0.3330026649008444 0.8286601522151827 0.9941405073548101
0.6664957670383488 0.9950083974306881 0.9933184370166649
0.4998576037284558 0.8286848724328139 0.9974694660467339
0.8335511830369025 0.9951843863040630 0.9925899181169633
0.6672175748642041 0.8290897833718178 0.9975178419992927
0.8337067343873928 0.8288620876454832 0.9936993320520318
0.5011070785494717 0.6638977187685048 0.0082150197422504
0.3582348541824502 0.5577724237948231 0.1296626653618617
0.3547337242416029 0.5432536685482638 0.2667583974909045
0.5037325908380319 0.5147474762596693 0.2668347007892437
0.5226100941501021 0.5443822663002342 0.1324653651044805
0.6434968084058444 0.4722013126823716 0.2635501192327956
0.6396999729427679 0.4660644753311358 0.1259540906877678

PT6-IV - ΔE = 0.14 eV

1.00000000000000

14.7772044699999991 0.0000000000000000 0.0000000000000000
-7.3886022400000000 12.7974344700000007 0.0000000000000000
0.0000000000000000 0.0000000000000000 18.0009597799999987

C Pt

72 6

Direct

0.2176889833004765 0.2797932917979438 0.0091216781446235
0.0531645338955400 0.1147678439092061 0.9981970733249597
0.8870841921587029 0.9482626153078755 0.9965078626326189
0.0535166151025024 0.2810781812101055 0.9995724996181323
0.8863871463786879 0.1143992830967733 0.9946901119726519
0.7206413169800641 0.7828087522832874 0.0025008667218813
0.5540158910283282 0.6169817407111609 0.0129846792108950
0.7199847876654459 0.9483229867516414 0.9936808892722482
0.8868159656265320 0.2813851839264458 0.9939353552818986
0.7201759661453266 0.1145526439693043 0.9918227915919431
0.5530614223934549 0.7814627251919504 0.9960272162669170
0.3863298932333876 0.4489257343899808 0.0233163881204135
0.3859371112952701 0.6145885185855917 0.0030098393257560
0.5528954042589405 0.9477816343754952 0.9915335896735513
0.7198391952415477 0.2809113984647951 0.9925124665086855
0.2200851481394963 0.4484779470600273 0.0062266980492254
0.3864303878602016 0.7813213216673560 0.9935043323201640
0.5532172139686580 0.1148926029270143 0.9928548770171446
0.5538724441109437 0.2811730821079124 0.9970309585764454
0.2200271356321366 0.6143937184014874 0.9971584174480270
0.3867232362672510 0.9475706761172802 0.9929050924300498
0.3861844519481608 0.1136273390615443 0.9983115575932544
0.0530279230595099 0.4475164985262268 0.9969017422714543
0.2194844493587595 0.7807192953996349 0.9938451689644623
0.2197706500941408 0.9478267019496656 0.9959245716037728
0.3870964125843841 0.2800742550767907 0.0134437980261453
0.0532240260178440 0.6145380027863254 0.9946788305173229
0.0533641783066088 0.7809662768995409 0.9953151503385627
0.2195446074284106 0.1137664236622342 0.0023876065147377
0.8869092134707586 0.4476599683631903 0.9937571954650508
0.8863832381329715 0.6140150702088150 0.9957913507467140
0.0527081740323325 0.9475416893791788 0.9969867051981325
0.7202165025994702 0.4478504135735477 0.9956383986985244
0.8867836658407455 0.7816637928222860 0.9983761013991099
0.7213230451301769 0.6141744011841723 0.0033124943052556
0.5540567567943029 0.4468127644523534 0.0049207542970535
0.1633693168177714 0.1696969479516994 0.0027290324003033
0.9975058674261135 0.0030726186393224 0.9967616405190327
0.8319353149020330 0.8373977817461622 0.9990300393127214
0.9978085039273807 0.1700583166414873 0.9973810937619589
0.8310389191067884 0.0037797288472561 0.9948280552022055
0.4981920170904885 0.5026394573229496 0.0173584268701816
0.6642978605050232 0.8370209760965039 0.9969691388092627
0.8307627866200349 0.1697961334531541 0.9932579384283144
0.6644195020246144 0.0034723518160575 0.9918243952703634
0.4974900348376252 0.6712372185871729 0.0031019414354674
0.3288022225512250 0.3339925808414108 0.0216813040020298
0.3301796201947695 0.5044393085627235 0.0110120962877573
0.6642621127158392 0.1703016130892152 0.9919699632999297
0.4973532540596892 0.8368889312092023 0.9927366249967164
0.4971711725875636 0.0028842731831276 0.9921076283431631
0.3305794774373538 0.6697834099535527 0.9970526395815824
0.1635958730175275 0.3359215113548117 0.0055863954911288
0.3309515908657801 0.8364364707332044 0.9929265771122715
0.1647102898240931 0.5034115727876056 0.9998291970626951
0.4979257878016412 0.1698111887840952 0.9959400629316448
0.1641960168469865 0.6701172345484352 0.9947991912239900
0.3306632934004767 0.0031326688970381 0.9953420124435155
0.9976097580889061 0.3368831315795759 0.9967265581127691
0.3303071676719753 0.1682127094918968 0.0045826856756932
0.9976070983995129 0.5030087737596887 0.9947831800241715
0.1637840942022066 0.8362123435222912 0.9945945763467492
0.1643596539118022 0.0031058693364869 0.9980298475832470
0.8313529029552313 0.3367288283501111 0.9929984824203757
0.9978449550899029 0.6698430361192607 0.9948543616462118
0.9972471099045137 0.8366668132310622 0.9965378845231072
0.8311179538663467 0.5034211979965093 0.9946881976508379
0.8307165904354648 0.6693484959035985 0.9989364857471728
0.6643452546948012 0.3364093167600046 0.9942782592100130
0.6649205109246452 0.5030470474131690 0.0002500011704498
0.4991042540533996 0.3371717028030332 0.0036461073464054
0.6660530954361761 0.6713834978479198 0.0104734329432077
0.4832527893741556 0.4782216302539055 0.2691893982887308
0.3543376264480358 0.2857533890683968 0.1319266489426454
0.4612662295249379 0.4825107307386531 0.1343945515370564
0.6112542752642298 0.6650088154247911 0.2656563223979447
0.6183397945975670 0.6652034251650676 0.1283135223138672
0.3540293263819123 0.2911012681807605 0.2695594456395725

PT6-V - ΔE = 0.17 eV

1.00000000000000

14.7772044699999991 0.0000000000000000 0.0000000000000000

-7.3886022400000000 12.7974344700000007 0.0000000000000000

0.0000000000000000 0.0000000000000000 18.0009597799999987

C Pt

72 6

Direct

0.8937178199079737 0.6161230552988477 0.9908650370027161

0.0601963998201241 0.6160132395206734 0.9927399532877004

0.2268334866208168 0.6162754873496973 0.9972809514627358

0.8937206842924250 0.4494748610979075 0.9938725068617354

0.0600176437652095 0.4492216621162283 0.9947032741589689

0.3942336340971097 0.6174517463975108 0.0017814604411089

0.5609019459524561 0.6165737107281891 0.9955990977537585

0.2253180681615403 0.4486965230568652 0.0011077843633563

0.8936925786571956 0.2826994458968170 0.9937436524790554

0.0602418035770318 0.2827740708488474 0.9934649149082649

0.3941613261898987 0.4491116237477082 0.0098976881376402

0.7270418709980930 0.6160362403622299 0.9916343415140859

0.5610994178041031 0.4508017658647632 0.0010319879113809

0.2266688278336498 0.2823317246311262 0.9961239176349139

0.8935884440878743 0.1161108653730375 0.9911344417746052

0.7274780968892304 0.4497049386631397 0.9965947287703518

0.3921149282889259 0.2818529087431029 0.0014937847128412

0.0601959548683326 0.1160372931559202 0.9906576020553144

0.8935785934021941 0.9495563103958264 0.9888625149273889

0.5606360262124831 0.2820620628791692 0.0090779444717369

0.2270973559126885 0.1161656034956654 0.9926783478663420

0.0603567933680637 0.9494447628794839 0.9891618963691220

0.7273786309700815 0.2828308033817507 0.9982098968793467

0.3935206702867824 0.1155703258828196 0.9965622064427800

0.2269055318520282 0.9493511468579847 0.9914069374295883

0.8935565668298298 0.7826732853559524 0.9886644427978598

0.5600591407126947 0.1156084014513439 0.9976296639694455

0.3936749171477490 0.9494230170353631 0.9939328528283065

0.0603128738882949 0.7828640313024948 0.9900021525163528

0.7268637515152577 0.1163092019515423 0.9942229957385829

0.5603220040562572 0.9492604672804912 0.9935616387393169

0.2270249402301491 0.7827946562090707 0.9930256557594341

0.7269130393943076 0.9494942405186209 0.9908663125114050

0.3937203271757826 0.7829929926613739 0.9951187492108554

0.5604287127289546 0.7829174252771176 0.9933070554050148

0.7270098492917335 0.7828348896660361 0.9898850213068070

0.0050222688250708 0.6713878797616353 0.9908372899337093

0.1718388893191875 0.6713414561098447 0.9941916542299606

0.3383580152964022 0.6718563487025414 0.9982318606282914

0.0047936167921319 0.5048039078087265 0.9934907502964994

0.1711363255575802 0.5045393562466431 0.9975820817230443

0.6715614674744722 0.6715204099996502 0.9916417980413615

0.3375835831318643 0.5051167511127244 0.0090862042088577

0.0049494150369423 0.3381569907507486 0.9938499230190020

0.1710991976281433 0.3380012165756778 0.9965043100302551

0.5063254300481322 0.5055794313381859 0.0010380834459415

0.8383631265481686 0.6714843863869480 0.9898744761997589

0.6720930038364216 0.5053743487872121 0.9955827049907527

0.0048790536130845 0.1713826645322354 0.9913672059307582

0.3373774189757766 0.3365890242081808 0.0014763738720597

0.1716958756077655 0.1715731105867971 0.9926573496140657

0.5046386362835428 0.3386387666388557 0.0098963037166716

0.8384480267933876 0.5049061045764826 0.9932793966140264

0.3378606778299495 0.1711411389688448 0.9961677249057469

0.6729807943221278 0.3387136669618331 0.0017435668477732

0.0049752380410701 0.0048317964758089 0.9891591617318609

0.5042253849577237 0.1697930727852829 0.0011386606237976

0.1715657825444570 0.0046698605587150 0.9906793741001646

0.8385197462896695 0.3382017776684512 0.9950750275756677

0.0050885326480167 0.8380562820141343 0.9888584483263101

0.6718027180205579 0.1713143494251197 0.9973016733181039

0.3382983767252144 0.0047186345653399 0.9935339213178379

0.1716408446497904 0.8380653073174261 0.9911515914286479

0.8383221641037031 0.1715044803306924 0.9930143980740070

0.5047451843048486 0.0044914977686629 0.9947866717910715

0.3382230923924309 0.8381692894857883 0.9937946819888064

0.6715398692635343 0.0046711374754582 0.9927920443704455

0.5050011542958851 0.8382001191312088 0.9939214093836929

0.8383914812806119 0.0047892080343612 0.9900091977294322

0.6716524095048262 0.8381941564728207 0.9908848984410006

0.8382005447333479 0.8380305623680003 0.9886690302986497

0.5052332512844721 0.6719562528016433 0.9966244992162868

0.5379284589114939 0.3171021939872357 0.2625200627121310

0.3725365676461931 0.4891621974636939 0.1237849022140907

0.3724021550419560 0.4819043287194461 0.2625212713913842

0.4227095889433025 0.3670969571375789 0.1861909012068014

0.5411666420503840 0.4855972770381792 0.2907812239951983

0.5449082092370011 0.3171914120637360 0.1238012014833832

PT6-VI - ΔE = 0.23 eV

1.00000000000000

14.7772044699999991 0.0000000000000000 0.0000000000000000

-7.3886022400000000 12.7974344700000007 0.0000000000000000

0.0000000000000000 0.0000000000000000 18.0009597799999987

C Pt

72 6

Direct

0.2215793318957263 0.2709829718436262 0.9946408432988658

0.0551530614782322 0.1045648239655756 0.9921086733911950

0.8884276219037714 0.9379855448300878 0.9926505894181251

0.0549883098351885 0.2712550970976815 0.9941015761791437

0.8886359917920535 0.1049010940078629 0.9919766998218659

0.7219259344936659 0.7721260888033823 0.9940909356485121

0.5556413785656098 0.6053196690098940 0.9946736918902396

0.7220288775202094 0.9383712805026979 0.9913291745019264

0.8887345107594850 0.2713358791803628 0.9940098074128646

0.7219061923128539 0.1048953932821317 0.9920718202408368

0.5552989617923472 0.7718116694522321 0.9922439006011037

0.3887377378296279 0.4373813020810881 0.0033120660202182
0.3892326578636447 0.6066764811411325 0.9975022382752033
0.5554363683900824 0.9386719621489041 0.9916417725226723
0.7222539769996856 0.2716235423923229 0.9947107469262730
0.2207058150212516 0.4374957851266499 -0.0001867270059459
0.3889806153718309 0.7720393778842704 0.9931686490539633
0.5554734334326065 0.1050991618392904 0.9928385739561066
0.5549636392212395 0.2714969754206007 0.9961567576826408
0.2217578754379721 0.6048359529111522 0.9967722682253873
0.3886457002705533 0.9383603095321854 0.9922353749397022
0.3885920428363991 0.1050154990550388 0.9925376402811665
0.0550762573486059 0.4382323232569865 0.9963166411582222
0.2221292350156328 0.7718025073638968 0.9940622530741635
0.2221588903407187 0.9382139384625410 0.9924325375185771
0.3881272366698281 0.2709611064443380 0.9942930397517348
0.0554545439472252 0.6046780467509659 0.9963348932495004
0.0553818301938783 0.7715477198973476 0.9950115532857980
0.2217808230500991 0.1045762964695216 0.9919977215842443
0.8885258285565484 0.4379464179478503 0.9964603625111671
0.8891994998193680 0.6052608037589978 0.9989251705618235
0.0555266427483656 0.9384589675574424 0.9927442728685624
0.7227423795333445 0.4371447347177351 0.0006919774327735
0.8892482509622249 0.7720034788992790 0.9963363404624003
0.7225901915535787 0.6062255654843711 0.0059530777579473
0.5543952152305638 0.4377984074158447 -0.0013487616060030
0.1665702835948210 0.1602146282692849 0.9924202908068926
0.0003772657801439 0.9939177457848012 0.9923518784791235
0.8336180330483645 0.8275989004737031 0.9945159714806023
0.0000009516354372 0.1604651329234241 0.9925022706887088
0.8333842959006953 0.9937930070797574 0.9917210071617901
0.5011184373596161 0.4941873675824451 0.9964491740859379
0.6669875161292369 0.8277382546639189 0.9918182509852825
0.8336074312284660 0.1605964743569238 0.9924896867938541
0.6667731673960747 0.9941854032776206 0.9913863945053339
0.5004311421928674 0.6613592718370308 0.9939596414139658
0.3325722713593046 0.3256464492012038 0.9969942080116839
0.3330734429437162 0.4945387342855707 0.0046891410014925
0.6667882514934641 0.1605000363639791 0.9930430392311304
0.5000994854876565 0.8275293161642985 0.9921721054771538
0.5003337794466861 0.9943370996926472 0.9920133081761831
0.3339726905753755 0.6612250776624836 0.9954827171016677
0.1664968216174088 0.3270117433522348 0.9960127926901434
0.3335832266332853 0.8276907945899397 0.9929444389415335
0.1664111452251268 0.4940255321444497 0.9979462851712342
0.5002082730055267 0.1610722406770492 0.9937918653982215
0.1668662866464245 0.6603541719913050 0.9956666127637867
0.3333913082498634 0.9938727952825932 0.9921731898261760
-0.0001024719365164 0.3269885362936690 0.9949304774611727
0.3332583131430518 0.1601461012778762 0.9924361645049492
0.0003392353157459 0.4940015676032901 0.9963130423308574
0.1670334805541707 0.8271944046385242 0.9936461320796106
0.1668631119945532 0.9939034967165700 0.9921378327596232
0.8334318116326489 0.3271987546115642 0.9951249401373027
0.0002674489546926 0.6606911506802883 0.9966319231751333
0.0004412207416369 0.8273169393152372 0.9944666393053869
0.8335722593445868 0.4933562941876891 0.9987929485534370
0.8350260978025172 0.6616340057943648 0.0001272178285239
0.6672840027897675 0.3273879124339280 0.9969098132798535
0.6666402208098028 0.4933109569975158 0.0079006862684471
0.4997055557788271 0.3269177465816474 0.9965315345365967
0.6662403196395721 0.6615646634090269 0.9973286203203061
0.6987104652902282 0.5551513519304133 0.1215605499968026
0.3595988147711666 0.4668158894159508 0.1193928806974185
0.5210151755768365 0.4820626618274322 0.1798346293250898
0.3661713484711109 0.4607936748995481 0.2579988510798732
0.5507806692067380 0.5963434675504462 0.2931781456596784
0.6835584612964005 0.5409313477102811 0.2607121253067661

PT6-VII - ΔE = 0.34 eV
1.00000000000000
14.7772044699999991 0.0000000000000000 0.0000000000000000
-7.3886022400000000 12.7974344700000007 0.0000000000000000
0.0000000000000000 0.0000000000000000 18.0009597799999987
C Pt
72 6
Direct
0.8872485438921816 0.6118585530021327 0.9935070161777845
0.0537675227911620 0.6123229641568797 0.9934697318819684
0.2203636055721626 0.6124761793517634 0.9956547445346389
0.8874112222591485 0.4453784960607619 0.9928411843754861
0.0533788835278219 0.4452360026476071 0.9959935402557738
0.3872783226896093 0.6136343585098828 0.0005090410578524
0.5522330781919180 0.6119131535844893 0.0075742060796191
0.2195114944759702 0.4447102355497996 0.0037924399494038
0.8874679140849563 0.2790944731590939 0.9942883164658229
0.0538593789069456 0.2782385744407088 0.9953754391757627
0.3876711706324245 0.4449377085973263 0.9941518553601359
0.7219385637725155 0.6120443692617417 0.9988947394447260
0.5537332634108358 0.4454087720639815 0.0026520508547570
0.2199730495803063 0.2776100386520679 0.9944323276015063
0.8867742449388570 0.1115763678047080 0.9960611925039041
0.7211140582633319 0.4456604814821801 0.9939786012285373
0.3856337371274934 0.2778933975952113 0.9995752858585366
0.0534896054612171 0.1118767528250367 0.9957598997492350
0.8872190287669799 0.9454159512513078 0.9992919434888121
0.5543853275441262 0.2779078761450615 0.0008070116175755
0.2202504383632579 0.1116866911002714 0.9958703818431094
0.0534809018920007 0.9451224919821166 0.9964954028146830
0.7203707792424368 0.2785550252908044 0.9941250080625987
0.3868761667015541 0.1116770149719045 0.9981752365549443
0.2204276989970150 0.9456413595315903 0.9953281574012343
0.8874096362452529 0.7791627386209089 0.9987845726038271
0.5541413168856716 0.1122359033324585 0.9962971679203996
0.3874545671223982 0.9456741754248001 0.9953000592808963
0.0539806942595066 0.7788743163838058 0.9944443773328555
0.7208723499485976 0.1122515122944605 0.9953106810244120

0.5538995123655476 0.9456221082271629 0.9958755002696804
0.2203013033016979 0.7787713333977284 0.9938110875403229
0.7204471048118101 0.9455489275194305 0.9985781314336147
0.3871452704320913 0.7793252909440744 0.9951817496392728
0.5540611896077559 0.7799026489259031 0.9995068015868823
0.7207020073091261 0.7792648020005358 0.0062061332829628
0.9982745130353182 0.6676622100210400 0.9934956478623962
0.1646372219466035 0.6680246783803683 0.9937689341270372
0.3312686045779126 0.6684035191686660 0.9962804937407015
0.9977865585291984 0.5009363161258367 0.9937795051576087
0.1642809019683184 0.5016880937771705 0.9988479213986193
0.6654802503664874 0.6677370959128279 0.0096291670063295
0.3320116110394397 0.5014400029534762 0.0039481999211387
0.9980362093925110 0.3344217121127828 0.9953554866652677
0.1634961508131870 0.3324106262985594 0.9969193449206989
0.4957001108227351 0.4999099547165352 0.9954008534465654
0.8315657686064828 0.6675067602968278 0.9965672278774865
0.6667877944524424 0.5014940925768636 0.9964163987415895
0.9980187506814698 0.1670511065986813 0.9953302493130143
0.3310114301546838 0.3330405691859592 0.9942707029679640
0.1644095420055969 0.1671325225021909 0.9950480347871686
0.4974288028646707 0.3333388181206376 0.0063723084927552
0.8317035392812567 0.5011618838718768 0.9926886251654473
0.3305940103378877 0.1668938409162664 0.9981203083185359
0.6646806314348949 0.3335388283919793 0.9956797886957957
0.9977335842952864 0.0005640115735290 0.9971595260257986
0.4982592443782206 0.1673400038831176 0.9986341983380412
0.1646179373574849 0.0005656703161591 0.9956778826552460
0.8318394082190999 0.3343950388463513 0.9933478610410349
0.9978469141682010 0.8343193436573071 0.9964385382630326
0.6647087559566357 0.1675927758144258 0.9952227241486730
0.3313655849238689 0.0012394402516695 0.9961207338852681
0.1643624596188056 0.8341657054844606 0.9942730876620804
0.8311634418231770 0.1673253169478428 0.9950422079677000
0.4978317684397920 0.0008303179469964 0.9956028826626918
0.3315185499011328 0.8345878674789302 0.9944116922588518
0.6648355095167501 0.0009908829838837 0.9964297903486805
0.4982331503441968 0.8348878348268809 0.9963874810736914
0.8313789309395561 0.0010165391565796 0.9983108878387497
0.6646416480994064 0.8346681431406293 0.0014864240536419
0.8317514789567638 0.8344311860610760 0.0020133292840737
0.4976124154492003 0.6690528264766631 0.0023921287710608
0.2714344734672688 0.4748411414294462 0.1187614518401886
0.4238452116920384 0.4749617080500064 0.1856747982730985
0.5479448144061398 0.4201542560072078 0.1196725473792040
0.5955816804481848 0.6164863314516039 0.1231136421569249
0.2795079229916192 0.4709873831461354 0.2556882886487494
0.5977566848945060 0.5185648533665933 0.2432580118910241

PT6-VIII - ΔE = 0.37 eV
1.00000000000000
14.7772044699999991 0.0000000000000000 0.0000000000000000
-7.3886022400000000 12.7974344700000007 0.0000000000000000
0.0000000000000000 0.0000000000000000 18.0009597799999987
C Pt
72 6
Direct
0.8861561223904019 0.6084094153217109 0.9933879547264892
0.0525331121906935 0.6087295861750754 0.9913323562369416
0.2195032292316540 0.6085959761694160 0.9932893908510437
0.8865411902542556 0.4420700137171837 0.9922561994288669
0.0524732386304677 0.4413717458735154 0.9902234006884224
0.3841596392085513 0.6075941758652091 0.0004385225637106
0.5529860387357030 0.6089314568121341 0.0119628117620120
0.2192763833755425 0.4421665350418696 0.9914811670619494
0.8858046475454553 0.2754220381153413 0.9914834516722612
0.0533004388423037 0.2755076735460896 0.9894200659651495
0.3869337336395230 0.4423341422378783 0.9980807617332569
0.7204741768617751 0.6090977986186999 0.0003107800577172
0.5529893620631215 0.4417590943307514 0.0117009952886186
0.2192836415313700 0.2748470335145821 0.9912569955888167
0.8858187284518417 0.1080894978967065 0.9915853968957448
0.7198560016742279 0.4427731155457473 0.9984142602188194
0.3841671480523509 0.2742842384628265 0.9999842142483455
0.0524794337274486 0.1088194392865642 0.9900729904536208
0.8865577349574086 0.9421916534341541 0.9924062778709893
0.5536572545138267 0.2743765206177159 0.0032700834402874
0.2195167270416718 0.1086446087145394 0.9928486807539481
0.0525460801473049 0.9415294320467641 0.9911802952930584
0.7192351835826155 0.2749151856506239 0.9961622902654730
0.3857768526821801 0.1079037574574406 0.9983911364125473
0.2191516728858983 0.9420752288398274 0.9938451293853131
0.8861671459104840 0.7754674407743727 0.9934589935126729
0.5527280208735603 0.1085582525337416 0.9986183916859396
0.3865921242014319 0.9421544774393712 0.9975199523950249
0.0532150052136503 0.7754658233298091 0.9919568642112774
0.7198960870430507 0.1088009525711085 0.9953873803851734
0.5527372631826741 0.9418809378224897 0.9988449844199783
0.2191513744799646 0.7747954598830882 0.9940469359898343
0.7192502866863084 0.9420277866107868 0.9964826347882211
0.3857763132290396 0.7755839432581182 0.9988529916995756
0.5536672213910235 0.7770027098636152 0.0037579278402143
0.7198766228885671 0.7748062311834329 0.9987476069408098
0.9974268081684288 0.6639929532775770 0.9918391625366355
0.1638244098230770 0.6645262106659777 0.9925254052171579
0.3294959557190110 0.6636079596447195 0.9972595161434751
0.9966986063895156 0.4972536671775248 0.9909297947184853
0.1639807848178868 0.4972268855195137 0.9912727214191440
0.6655370652043059 0.6652875012772578 0.0034627730312841
0.3304591896091051 0.4979025214454051 0.9957136938397002
0.9973893925877041 0.3312680314932774 0.9899234650507938
0.1633787601537025 0.3305570224484258 0.9899207548461461
0.4957127505321583 0.4967184139198206 0.0059217045895374
0.8307401490633317 0.6642310637399959 0.9952812171042801
0.6655380398759597 0.4979559994075436 0.0032512238104739
0.9973888609383731 0.1638307837929290 0.9898928463277444

0.3304606424389506 0.3302874499067113 0.9954537953832786
0.1639963083938448 0.1644889728667059 0.9909458223922117
0.4960194406691277 0.3293690259012388 0.0096406801780627
0.8306987001309276 0.4979332066383734 0.9941234988615641
0.3295136316272180 0.1636180271690635 0.9967364144996624
0.6636622632864887 0.3302319178228410 0.9995311795268723
0.9967092990038604 0.9971693703529703 0.9908643041529872
0.4971858880283207 0.1632612425074090 0.0004048406563957
0.1638314155581944 0.9970228860020998 0.9922157014140726
0.8308018740557597 0.3305965385111529 0.9930411044081803
0.9974436390637349 0.8311593485178006 0.9918025109501439
0.6639186015457315 0.1643524650037482 0.9969264355600432
0.3307218796146949 0.9979489507675409 0.9964977672706420
0.1636255984206059 0.8306733017240273 0.9930123109876183
0.8300593314461957 0.1638730714641402 0.9926653156662202
0.4966702672838921 0.9971904877826626 0.9983161843470401
0.3307142645086595 0.8304753642259395 0.9967580888246239
0.6639212577428933 0.9972662408826992 0.9971219919633794
0.4972016721733548 0.8316422689893945 0.0008304957597921
0.8308264756771570 0.9979186062810507 0.9932659689143338
0.6636811579379154 0.8311480821248758 -0.0000509567255247
0.8307111588337925 0.8304794321076119 0.9943324434716945
0.4960226014258448 0.6643623386332236 0.0100876219225672
0.5331750150721225 0.6560871467050206 0.1251232511738680
0.4802410931465052 0.4888526418208965 0.1935803035751509
0.5333383504373437 0.3748999494660841 0.1247588273394368
0.5359968994221627 0.3716217537709284 0.2648629357746287
0.6361945374419961 0.5669676761136583 0.2822014401698181
0.5349776736471838 0.6612557883891165 0.2653107830595829

PT6-IX - $\Delta E = 0.43$ eV
1.00000000000000
14.7772044699999991 0.0000000000000000 0.0000000000000000
-7.3886022400000000 12.7974344700000007 0.0000000000000000
0.0000000000000000 0.0000000000000000 18.0009597799999987
C Pt
72 6
Direct
0.8896981302937753 0.6177885539068432 0.9921213972222731
0.0563038571445739 0.6176723237703916 0.9922179623532286
0.2228094161896020 0.6178776846339885 0.9945999726821139
0.8895155798161473 0.4508908384635137 0.9936408581610070
0.0559070358053411 0.4509509851134398 0.9935131105902357
0.3900564684572976 0.6191672980242834 0.9995461214800052
0.5574283671184598 0.6182645618829858 0.9942223253532976
0.2209819588811885 0.4502914019453688 0.9985751494682585
0.8896067746490217 0.2842548558982687 0.9933526942790014
0.0559886796595910 0.2842676944248734 0.9948178284192446
0.3898400589898859 0.4514860831659208 0.0083653927903470
0.7232607345115420 0.6178092241351195 0.9924349633760444
0.5574675093822848 0.4528732263027188 0.0006608371478819
0.2231683236129454 0.2846716249853258 0.0011184212160367
0.8898895693946197 0.1179902453600875 0.9927192113078223
0.7234136507322814 0.4511927956762505 0.9967221882184106
0.3886401402036749 0.2831589702480498 0.0122817895058483
0.0568144934706668 0.1179115102340134 0.9952156751422522
0.8897911184996342 0.9512384010895125 0.9915349020724477
0.5568951897792803 0.2837742520901932 0.0034942904369340
0.2227431901989128 0.1171663596601069 0.9988545763131992
0.0562984045368965 0.9510818047245296 0.9926941972107766
0.7230973982055288 0.2844142055124144 0.9958935246282298
0.3892176883686657 0.1168787974828319 0.9975547782458103
0.2228637542109873 0.9508074301025904 0.9932881659847861
0.8898215856647483 0.7844646985560385 0.9907239277052267
0.5562458731920027 0.1176134919608822 0.9951554291214766
0.3896100630591660 0.9509177037577763 0.9927645650396555
0.0561370546330704 0.7842766702231404 0.9913997648659247
0.7227541798686136 0.1176601828161329 0.9930601354705585
0.5563297309623577 0.9510598662422513 0.9924298539353038
0.2229731408790606 0.7844658016449702 0.9923003252382301
0.7233012864930942 0.9512368392672642 0.9914031882899843
0.3898458800409940 0.7847758982465307 0.9937976480118866
0.5564916766162927 0.7844616453992715 0.9926342678360476
0.7233419660945357 0.7848221768808762 0.9905824139247156
0.0005178015814660 0.6731202897565242 0.9915950316185036
0.1671173770341984 0.6729680819699355 0.9926923553069994
0.3335724087439129 0.6736381289015950 0.9961732832959495
0.0003151746998654 0.5064467998456759 0.9928414074675594
0.1665222924128492 0.5063353259807305 0.9950707523289122
0.6674614887491520 0.6732265355399576 0.9914766650818205
0.3325753842042230 0.5066506177905733 0.0040709119396226
0.0003316167872027 0.3396532944791134 0.9936518595423438
0.1666858080777597 0.3398825477758502 0.9976699922753108
0.5022792194080521 0.5074003763073770 0.9997496534148733
0.8341674912317831 0.6732036211882546 0.9914249671354828
0.6678440056204857 0.5071772536089514 0.9961500949382055
0.0004968015868938 0.1732185874134093 0.9941994205329034
0.3323618019662890 0.3391779114428299 0.0073273828228966
0.1670532665885661 0.1728523055071960 0.9987910010074152
0.5017203289080884 0.3405312507026308 0.0098563629526941
0.8336718681871276 0.5062783914040686 0.9939369519160967
0.3330030573537697 0.1713789477666572 0.0027585450944193
0.6677589608369695 0.3397926100541256 0.9990633828999960
0.0008049662618102 0.0064951082767003 0.9930609999256682
0.5003902350159919 0.1724583875233026 0.9987094623603683
0.1669965023782930 0.0061228262681537 0.9945715927985983
0.8339647685288050 0.3399065583280351 0.9941235445351484
0.0004631552923159 0.8395628253421481 0.9912364628332989
0.6671497063494937 0.1729180904186539 0.9948416367221045
0.3336419702666133 0.0061751714018933 0.9938176575243176
0.1671481346662378 0.8396569899609779 0.9919851667040780
0.8339811403108023 0.1731980861414233 0.9929481293372390
0.5003074696786625 0.0061532010003742 0.9931935297563967
0.3337362057736153 0.8399629600593949 0.9926397019911209
0.6672013649021959 0.0065468919035688 0.9921903578696757
0.5003986373227439 0.8397733352665750 0.9928986322673321

0.8339908291964820 0.0063981695656765 0.9917451191012738
0.6676199952305549 0.8399802864834049 0.9911968707556887
0.8340649682603712 0.8400020280464938 0.9906334516560339
0.5012501559424883 0.6741162920416700 0.9952607730948644
0.3858890186904069 0.5222188585852443 0.1170122730900841
0.4615651499130962 0.3032322359640089 0.1245310829366235
0.4163586652327942 0.5688612832861968 0.2535966125318490
0.5099144324830728 0.3295447973958306 0.2603767881282108
0.5542516244992015 0.5175575923335857 0.2644500162996646
0.3833631154401683 0.3878654545933720 0.2024386504967334

PT6-X - ΔE = 0.47 eV
1.00000000000000
14.7772044699999991 0.0000000000000000 0.0000000000000000
-7.3886022400000000 12.7974344700000007 0.0000000000000000
0.0000000000000000 0.0000000000000000 18.0009597799999987
C Pt
72 6
Direct
0.8930186382355401 0.6140925784400887 0.9956823841187277
0.0595973410349722 0.6145111199603893 0.9978576506906347
0.2266429904921683 0.6150060266402680 0.0015936452395558
0.8930843966612605 0.4475031174401158 0.9980957762165524
0.0589303536410960 0.4473195149326579 0.0011562581902246
0.3933670054277928 0.6150863667247178 0.0128347365088264
0.5612137905145040 0.6142022409283614 -0.0001116935912549
0.2242267320473877 0.4459208905675954 0.0054039129054289
0.8930541530492300 0.2810946692010570 0.9971385026484452
0.0586690809760047 0.2799287501690473 0.9965036385008222
0.3931329225655633 0.4471602345213404 0.9960698231556986
0.7272548278498389 0.6145472057651934 0.9951019608011420
0.5606738006641393 0.4492061951082474 0.9979162073102752
0.2256800880632027 0.2802513805457327 0.9962153233747632
0.8927253051099840 0.1139666298638029 0.9952819728439510
0.7265845624696654 0.4479321637968822 0.9978903081439207
0.3920393468844284 0.2807595619324545 0.0058343036445603
0.0598341023227310 0.1142858840422211 0.9947688905819775
0.8935030909635128 0.9477361815593780 0.9951122255610397
0.5607882265821932 0.2806148256098044 0.0018699711974346
0.2265807856495239 0.1138295741745353 0.9961943523595316
0.0596662870529018 0.9473016763846988 0.9940297452487136
0.7264739072868984 0.2806986857957052 0.9981930304651568
0.3927625595897867 0.1138087341856924 0.9989263736128898
0.2264266114676472 0.9476544344475264 0.9951746182754867
0.8933200779424310 0.7811702697173801 0.9944723695310860
0.5598481493613426 0.1144917554242321 0.9983006592379289
0.3935194166890472 0.9477858990165789 0.9968913937745139
0.0600138362705939 0.7809126532562791 0.9946415410240078
0.7266628675318318 0.1143253766933470 0.9972029346452658
0.5597737129758638 0.9475179593176142 0.9988867398937255
0.2263502392177439 0.7809218878047374 0.9965288448914155
0.7268626744100732 0.9482782586762353 0.9976245320448660
0.3929737936374482 0.7813872996093645 0.0002380318981162
0.5614916756878781 0.7826530733449596 0.0045706768301135
0.7268585869718859 0.7811316719805755 0.9977211668309380
0.0041350361725221 0.6696555042952481 0.9958370404845933
0.1704447007653114 0.6701115839791733 0.9981704086664264
0.3364578495435507 0.6706124691928470 0.0033652438521146
0.0031704119315905 0.5028522103302966 0.9991960869331521
0.1694396466309475 0.5033204220691659 0.0047925581784575
0.6711818896187676 0.6699646547780591 0.9972186854240133
0.3364141642952836 0.5011942679076685 0.0071452299631914
0.0032980099462314 0.3361164334677339 0.9981390622945341
0.1686812618425709 0.3342500267397245 0.9987368895390900
0.5044674301290576 0.5034919215311610 0.9953725451086100
0.8374863658367113 0.6697394927003173 0.9947230035859095
0.6713354058877005 0.5037127283663672 0.9965133452098485
0.0037493680134829 0.1693257619599804 0.9948865241336975
0.3366947288751722 0.3369302508874287 0.9968140325503229
0.1703472664427831 0.1694875924510834 0.9956289794989934
0.5049446538706558 0.3374805949211332 0.0068836388525050
0.8369333269516589 0.5031107186269127 0.9971209254198111
0.3361195823143220 0.1682442152125506 0.0000601492211130
0.6705077231385356 0.3361275745768849 0.9993643303815906
0.0037191792690079 0.0027243145002742 0.9944127078875690
0.5042470079409203 0.1697133659500397 0.9998896900803647
0.1705886574135654 0.0025894168647970 0.9946100555756477
0.8373701541033549 0.3363462448944236 0.9978998599174954
0.0039150517591502 0.8363848897829261 0.9940485733448584
0.6706266083224581 0.1698076942569332 0.9980229679268098
0.3372207028963300 0.0031673037898835 0.9965845278830980
0.1703606975009183 0.8361779290529527 0.9950739025482184
0.8369729911437445 0.1694064135477774 0.9962711714966053
0.5036477362430872 0.0029325119599609 0.9977402407475143
0.3374882158161208 0.8365275289830464 0.9972313537500632
0.6710299315204926 0.0032058168874874 0.9980062305970929
0.5044755638334866 0.8371344040874485 0.0015924049092932
0.8374362643312716 0.0032362028422830 0.9957459514202477
0.6711013667232111 0.8368201543245376 0.9999161469490881
0.8374953652610552 0.8363726300662980 0.9954835707181454
0.5061898959793628 0.6705310213276583 0.0101198331142341
0.4510853835679476 0.3385928189077663 0.2570139449566090
0.4109147500841703 0.4530566076751693 0.1830020226557266
0.3451040473871175 0.5616168604421825 0.2465095186293651
0.2611586259525700 0.4683741309114462 0.1225504183574672
0.4498999619525720 0.3193248739564455 0.1210797427207856
0.4470884659127497 0.6377317887077281 0.1252798567612675

PT6-XI - ΔE = 0.52 eV
1.00000000000000
14.7772044699999991 0.0000000000000000 0.0000000000000000
-7.3886022400000000 12.7974344700000007 0.0000000000000000
0.0000000000000000 0.0000000000000000 18.0009597799999987
C Pt
72 6
Direct

0.8857449003193805 0.6072710464332274 0.9951790624982798
0.0524219279273512 0.6075051508733781 0.9957131679833198
0.2186878664000901 0.6072884621155074 0.0019147892481791
0.8859019409722817 0.4404832765836741 0.9963253799353213
0.0517883126994087 0.4404878301599425 0.9982235692755844
0.3846347635066781 0.6086016737158760 0.0138766471341411
0.5542941698802381 0.6084479234241599 0.0125899634584304
0.2169901600457358 0.4401663877624721 0.0081327883062897
0.8858258555401135 0.2742227450431986 0.9951163758790016
0.0524602282076101 0.2740444938311689 0.9954566148572915
0.3839479496293195 0.4388807684548054 0.0201961795702191
0.7197580436742446 0.6074727617059231 0.0008353332196036
0.5545715646467018 0.4403216796183485 0.0154495750584586
0.2186750338956565 0.2735046872618244 0.9975647488272500
0.8857865872437771 0.1075133157082938 0.9916829860966382
0.7200885915848527 0.4408913742119367 0.0013055592789067
0.3854486429553166 0.2737642636002741 0.0022559493106378
0.0523956335807912 0.1076526064301600 0.9901761760613551
0.8857977007268819 0.9406830630632367 0.9903244942881541
0.5526163964475820 0.2735968342665049 0.0039283092281140
0.2192465108839951 0.1074111490215785 0.9922195015258288
0.0523219490557570 0.9407497519490917 0.9892790747298068
0.7189198573091294 0.2739139976298246 0.9981747162875251
0.3856514836327827 0.1072739880283098 0.9969138739085665
0.2192497339801463 0.9410752068303575 0.9923014911235715
0.8859218106993828 0.7742307729004586 0.9918530278318446
0.5526055341241465 0.1077468251896276 0.9977027784088741
0.3858681339781718 0.9408668987113271 0.9964607857000354
0.0524681481553911 0.7739652921719162 0.9918949766687106
0.7191830289899421 0.1073017701388939 0.9952725710830416
0.5522962080642695 0.9412027172481318 0.9986546222281470
0.2187823854258796 0.7740318203627058 0.9955880733451836
0.7197184244504152 0.9413872922741029 0.9949881191127184
0.3854854287402618 0.7747623530527182 0.0014800542922207
0.5536319733368115 0.7758117755628935 0.0082489220183060
0.7193666199269119 0.7740907953798648 0.9967955096428358
0.9972062089732093 0.6629012929664239 0.9937716868127451
0.1635269537142046 0.6628606613045942 0.9972537471794676
0.3299337843976989 0.6627843163737343 0.0052840415605845
0.9968603046355339 0.4960822755963988 0.9964394752749111
0.1628691113094743 0.4960672467316248 0.0029915784930997
0.6634782657999084 0.6627018636320084 0.0023315017409722
0.3283713958595547 0.4966335016555896 0.0184515706150203
0.9967150871093807 0.3295761169200669 0.9963053860025124
0.1627612310010332 0.3289937575876070 0.0002579917048474
0.4990349357728029 0.4968463315180858 0.0215078278183502
0.8304159099353035 0.6627127169572794 0.9952722916731997
0.6659593333029947 0.4968437928470071 0.0062379557652574
0.9971579131200252 0.1627733265686983 0.9916448275805100
0.3295452710222080 0.3279897369776826 0.0041302026633983
0.1636696121452475 0.1630753677582654 0.9920480696997884
0.4967518280735125 0.3281570504538374 0.0092335549227104
0.8306493960880489 0.4963557775703082 0.9971847544990059
0.3302620079024004 0.1626735576968215 0.9962887626817599
0.6640323149091731 0.3292967140993953 0.0016295035976655
0.9970214578664119 0.9960500547513877 0.9891283026337424
0.4971496175623296 0.1626553172857425 0.9994767780293434
0.1639559784018217 0.9963431036667426 0.9905523446247837
0.8306153474528684 0.3295744279219743 0.9964919871175896
0.9969492853392311 0.8296054533701321 0.9903025351892542
0.6636221867326580 0.1629052123907559 0.9972820183195771
0.3302136238597805 0.9963934201150673 0.9948331649162087
0.1636833781338434 0.8293346531164330 0.9927219231722196
0.8304013996015709 0.1626587216988335 0.9938229782866355
0.4968129803285585 0.9962288066275545 0.9973413821293642
0.3303515571168876 0.8296482123930552 0.9973143305089619
0.6640235986362401 0.9963509526099577 0.9960573989568638
0.4962960322437404 0.8306604436283777 0.0029805251405559
0.8305513603986572 0.9963428300414793 0.9916454961934604
0.6652374929399971 0.8301706838388617 -0.0003939191209658
0.8304191225126322 0.8293496404395925 0.9920601904993659
0.4973377277162207 0.6636988199898054 0.0168175353812053
0.3347109956461699 0.4385832707859839 0.1310202090025166
0.4920039017225178 0.5861896682974221 0.2014472179826060
0.5178692951335797 0.4449286975173900 0.1323111117961903
0.4075041501030874 0.3996503454214373 0.2508254098171970
0.5471010681854838 0.7534146232123951 0.2640987313017240
0.5363499118962011 0.7474356876288438 0.1265263550365419

PT6-XII - ΔE = 0.57 eV

1.00000000000000
14.7772044699999991 0.0000000000000000 0.0000000000000000
-7.3886022400000000 12.7974344700000007 0.0000000000000000
0.0000000000000000 0.0000000000000000 18.0009597799999987
C Pt
72 6
Direct
0.8877146735204184 0.6140525320158735 0.9976472485549426
0.0544960124300204 0.6139657725887306 0.9922666343540602
0.2207496934014742 0.6138755229715614 0.9909003353771942
0.8874337076375505 0.4472079975011370 0.9962021080626667
0.0540830111706564 0.4475270744526194 0.9949746046728249
0.3878355312484181 0.6150177235055252 0.9961321075878218
0.5546258171899475 0.6169928042167847 0.0158976454323209
0.2205719250996591 0.4472764439965742 0.9972219657461527
0.8877526562744245 0.2809243110001992 0.9958742314554873
0.0539046192471851 0.2807136531036232 0.9996544519509482
0.3862753447424322 0.4483602686289525 0.0044130381690729
0.7219556130821856 0.6132815263043542 0.0072691268375764
0.5538722938178714 0.4461189600993679 0.0158626589302527
0.2197833034657193 0.2800857667115243 0.0078876074881969
0.8877667877294499 0.1145622351139607 0.9965257684260391
0.7212921905411246 0.4474715061303047 0.0022519687336971
0.3888337559940210 0.2797247205125097 0.9973954941381399
0.0544414412224809 0.1139563012700444 0.9980309904806927
0.8876252903994862 0.9474901730624232 0.9966525393220532

0.5542015791491596 0.2802839868647417 0.9964308480163837
0.2203982982233235 0.1133831300254883 0.9933941671530119
0.0540314890377035 0.9475570678515678 0.9934234962972877
0.7208134803277610 0.2811242907901810 0.9956782688076934
0.3877998949118221 0.1142512555032115 0.9891258677924317
0.2212483072252871 0.9473456997844714 0.9883520635112419
0.8880441555578233 0.7808552631139540 0.9979002439913549
0.5547657561531796 0.1142795885248206 0.9900973423186141
0.3875281518073520 0.9472341813446502 0.9871757775715877
0.0540395964157026 0.7803724184492635 0.9918899987819758
0.7208749922418179 0.1141364825594284 0.9930401056645064
0.5545143181768846 0.9480232057039234 0.9896930599024041
0.2209638464808840 0.7808598187576099 0.9882992200399944
0.7214556510792393 0.9478546469963510 0.9949203431225433
0.3879167891643104 0.7808791757599800 0.9892325353194974
0.5538530059006445 0.7812195919357409 0.9956949394197776
0.7219584395728121 0.7825608761061056 0.0025858008938713
0.9988076689114309 0.6696804594572937 0.9935806785107129
0.1652623473433582 0.6692060046291144 0.9899464777747596
0.3323881970668417 0.6697451870273087 0.9911911774568117
0.9988902827750880 0.5029219308861180 0.9942768360982528
0.1652583794065036 0.5029055672549407 0.9938547120334829
0.6668844295707934 0.6708258129556046 0.0126090561423694
0.3317077869848903 0.5027996804962640 0.9981272221981243
0.9982958618038480 0.3362180070575253 0.9968411811884071
0.1647946672611481 0.3366030604488436 0.0026193330846581
0.4972170533183992 0.5024367929956965 0.0180888401158831
0.8322086991185558 0.6690317462721075 0.0004176671818313
0.6662229395463337 0.5029378772875036 0.0091664563187486
0.9987057099732998 0.1698212294117594 0.9987261571962838
0.3322679803127341 0.3358657320751345 0.0072125976510781
0.1644073850136267 0.1680274141916719 -0.0001941385922149
0.4984917501792283 0.3349228275323494 0.0011437700164971
0.8319297776561861 0.5027841528782916 0.9986158162021487
0.3326543410979117 0.1693263225768292 0.9918163092403822
0.6657195280793254 0.3359649067386684 0.9986574344587683
0.9989615466286341 0.0026351334841408 0.9956628405926976
0.4988173112320762 0.1697153868450622 0.9909666095429404
0.1653301786901899 0.0031189079838292 0.9909134680205097
0.8320930913877121 0.3366043432520794 0.9960042414431023
0.9981052087476008 0.8357006571780846 0.9940388970140565
0.6651108601569355 0.1694211870965460 0.9926288813155323
0.3317131551091709 0.0024697674627586 0.9874665285483173
0.1654785601332311 0.8360715857011715 0.9889263435625274
0.8322601173587770 0.1696578446464540 0.9953595210934301
0.4989960538208944 0.0029196493521779 0.9882892911102324
0.3320189969212836 0.8363654599810051 0.9875731259881186
0.6654457532964814 0.0033576459025421 0.9921835250021523
0.4985517291688716 0.8362696156494396 0.9904682961661525
0.8320011543116178 0.0029231417313374 0.9961200029727197
0.6658586099113825 0.8368317122084953 0.9979573386289887
0.8326922606266037 0.8369914188677237 0.9988816422585278
0.4980952977367530 0.6710840106492951 0.0011158135464902
0.4015250823672770 0.3133163699411690 0.2360370573594382
0.6211776542233128 0.6643060361992241 0.1286756437832617
0.4997146285163554 0.4668805410978620 0.1342423599417966
0.6181059292480232 0.6793828217152090 0.2654039271908403
0.4729585072237286 0.4997195749671048 0.2666264722569688
0.3022556261186434 0.3097273348242905 0.1218507868698676

PT6-XIII - ΔE = 0.57 eV
1.00000000000000
14.7772044699999991 0.0000000000000000 0.0000000000000000
-7.3886022400000000 12.7974344700000007 0.0000000000000000
0.0000000000000000 0.0000000000000000 18.0009597799999987
C Pt
72 6
Direct
0.8889694498501449 0.6125284398257489 0.9891998276947238
0.0549843207106188 0.6118404298602625 0.9902023359518213
0.2216020714624387 0.6124480487871884 0.9935489577803305
0.8884455643720383 0.4457734078048053 0.9915424866312469
0.0556968676599497 0.4458064714665649 0.9921684279571124
0.3888355715092440 0.6129642938792836 0.9995140269680607
0.5561242857093498 0.6136703153589405 0.0097758981988867
0.2217064274968223 0.4451861848794160 0.9956321596535124
0.8885576470610501 0.2785619012355851 0.9937787546096312
0.0549553101165439 0.2790824253519452 0.9937475754633645
0.3869909202061705 0.4451419566246115 0.0033158372442769
0.7219874170303249 0.6128582164851951 0.9940854269999922
0.5570548246294792 0.4465446099271955 0.0026016850078795
0.2216508930733181 0.2788740174991881 0.9967685201705803
0.8887967012913814 0.1124552264665683 0.9947317536880624
0.7219892565435606 0.4452309994953153 0.9939696225046796
0.3869942008943848 0.2779082488139577 0.0031619645441270
0.0549411743161047 0.1119869876226563 0.9937394458753762
0.8885477435871244 0.9460970104941477 0.9937932588599628
0.5561384066686148 0.2784843145222518 0.0093043854293248
0.2216946707291490 0.1126287929295970 0.9955438461596936
0.0556815892392341 0.9459953624758197 0.9921562591038302
0.7228736996170619 0.2793521966625189 0.9975272868963704
0.3888163701583942 0.1119449776140279 -0.0007323910294505
0.2215831435475203 0.9452564455977212 0.9935066907455449
0.8884370786896255 0.7787543625039848 0.9915885642686191
0.5550739389622237 0.1114657816374410 0.0001879238919002
0.3884453572435609 0.9457590762699613 0.9967043175899964
0.0549853143303367 0.7792508806122497 0.9902016973455563
0.7216665582490343 0.1125488526132501 0.9973856948879469
0.5556553665579200 0.9458594029123728 0.9981810415392318
0.2222921759883346 0.7791989448843297 0.9926424004293696
0.7216611542631175 0.9451835881950793 0.9974606562484907
0.3884513545483577 0.7787743628226359 0.9967878484163128
0.5550712272472641 0.7796607824595005 0.0004319462515579
0.7228641282805682 0.7795891953104660 0.9977484475679549
0.9989975540158877 0.6675457046170283 0.9893460166464809
0.1664949798056669 0.6676354391976457 0.9917498217593540

0.3331350111319159 0.6680942531729525 0.9962396015906519
0.9998265634693702 0.5016137514349164 0.9908865713026519
0.1658316770999833 0.5009884223319852 0.9934468496615522
0.6680168120730361 0.6687615705374789 0.0003106884600181
0.3327175915876338 0.5004420398392913 0.9992707532073231
0.9998171183941330 0.3343661771526414 0.9929154289200283
0.1661796994937413 0.3346798512078988 0.9951822930869058
0.4996944723475080 0.5011315242223291 0.0104581387336142
0.8330177438177050 0.6682592832597610 0.9906790309996614
0.6656649780832453 0.5008749771167225 0.9949532507280190
0.9991286112408903 0.1676250428581351 0.9939283660384399
0.3318487259190853 0.3339525014764813 0.0007921484728507
0.1661689588714173 0.1676089980213567 0.9951438696247971
0.4997094666143724 0.3345743461844539 0.0102609755883817
0.8330225341736718 0.5008495087523059 0.9906355110966775
0.3327026117646650 0.1683483430067908 -0.0009151501716679
0.6680359637054291 0.3353379102173852 0.0000159464122461
0.9998044367160854 0.0015646791860778 0.9929002947071951
0.4986167269454709 0.1659745612502348 0.0029970596672195
0.1658150390731036 0.0009485116185033 0.9933981450288706
0.8328426984455134 0.3344021500916713 0.9939273496125929
0.9998196313642115 0.8343106444510787 0.9908968583795135
0.6665084657378555 0.1674565890503728 0.9989001738096436
0.3331189781291365 0.0011274633534824 0.9961130975054178
0.1664870902023400 0.8349568963982821 0.9917415297785110
0.8331645653251539 0.1682271382287901 0.9953582770568904
0.4995123979024116 0.0011161226292948 0.9983326548200697
0.3324323634207708 0.8342631940677819 0.9952035385734108
0.6657017562927110 0.0008851227303524 0.9979718827145515
0.4995178830057591 0.8344635384101213 0.9984366722563925
0.8331566692322571 0.0010383762691046 0.9953833756161196
0.6665043081555052 0.8351157003646843 0.9991114687867571
0.8328302467384595 0.8345260206872611 0.9940114268382378
0.4986179214509910 0.6686906622780520 0.0033614102978130
0.5301707931294357 0.5610451221261898 0.1244263101236375
0.5413492198531130 0.5733088491448771 0.2645527277275410
0.4351696333144985 0.3848147155227008 0.2087900860248101
0.6306007435452069 0.4823836789197582 0.1971641259076939
0.5410233135342309 0.3017550751467560 0.2641330151558410
0.5300647334521231 0.3038624381583974 0.1240422214451483

PT6-XIV - ΔE = 0.58 eV

1.00000000000000
14.7772044699999991 0.0000000000000000 0.0000000000000000
-7.3886022400000000 12.7974344700000007 0.0000000000000000
0.0000000000000000 0.0000000000000000 18.0009597799999987
C Pt
72 6
Direct
0.8888977570948340 0.6122762725557254 0.9934210630285893
0.0561689399687005 0.6124322880944348 0.9916909619122406
0.2223427355779177 0.6119041047099111 0.9949809965022908
0.8890325540262094 0.4451158978181322 0.9928031849960137
0.0552982861276604 0.4455167929587079 0.9928622229120692
0.3884706411324970 0.6124150755968371 0.0038094985742113
0.5570961185243994 0.6141586474425781 0.0086339556449259
0.2223375020098052 0.4461012859182279 0.0002403703535365
0.8892799407325954 0.2791186842405617 0.9923224204576927
0.0554850577577847 0.2784179919811076 0.9947907429744189
0.3866899309105096 0.4451290354410696 0.0209684536395392
0.7227825624434662 0.6119135021198563 0.9996789405308838
0.5571650416280888 0.4451104837545352 0.0177829288980243
0.2205692782931861 0.2778896705861204 0.0035069163664302
0.8886078152822345 0.1121829264815242 0.9921407447008495
0.7232379825040633 0.4459947235313133 0.9988261232126762
0.3899187969236446 0.2777048497111580 0.0057025300610816
0.0557416301378482 0.1122505156426586 0.9940434785245298
0.8887106795562988 0.9449587067057078 0.9921299675305799
0.5554903470349478 0.2780914944395275 0.0003648155433675
0.2221020500659573 0.1115151208506110 0.9966887930839775
0.0553197464453785 0.9456329262404470 0.9921477412518013
0.7219289755360618 0.2790901882074530 0.9949539122043868
0.3889951448838035 0.1120501566767334 0.9958703739981285
0.2228982820696827 0.9457758411574403 0.9924100031418708
0.8894167297714546 0.7792094783380723 0.9926820995535344
0.5561121781113065 0.1123157443940305 0.9941602317528865
0.3890809070491151 0.9451197749698517 0.9929161974192106
0.0556802608186720 0.7784842242885861 0.9913135110787402
0.7221862883797652 0.1117963406187041 0.9926853824090220
0.5554871881485065 0.9455445706957196 0.9938575611613873
0.2222609110174798 0.7789705509174233 0.9920810436016804
0.7226074673346607 0.9455881458135020 0.9935607988281667
0.3895071737330971 0.7791835074142454 0.9953785041817487
0.5556833304762151 0.7785183808952325 0.9985933431350986
0.7219649035090815 0.7787485152424222 0.9960889932802246
0.0004654658581308 0.6682043307689576 0.9916553517106146
0.1665259747166684 0.6676795050033563 0.9924164401628780
0.3334726059869929 0.6673966678057897 0.9977399213299468
0.0003081006972002 0.5009046917851693 0.9919580134871830
0.1669684737991407 0.5014712562662951 0.9952434987961732
0.6667690369314652 0.6682649505279019 0.0009436848417247
0.3319224421487358 0.5016111672111950 0.0075455131337819
0.9996020611123480 0.3344672329903920 0.9929538077401773
0.1661722200864955 0.3338628359318366 0.9997372302038624
0.5018939415166376 0.5032216522881203 0.0223450540929058
0.8334108746604358 0.6673237159969783 0.9945133283300847
0.6685203769633326 0.5018761114229287 0.0044704729245548
0.0001324238543464 0.1679711470556580 0.9934694737904496
0.3321602493845399 0.3327802364468214 0.0145425663147337
0.1657805669042382 0.1671043332987525 0.9977125987547333
0.4995110668962903 0.3327204859927346 0.0072680405492160
0.8333617464332778 0.5012266435656896 0.9945718409473431
0.3338695694170168 0.1673897895461147 0.9987346696901550
0.6670523214677438 0.3340525237515321 0.9985161958988299
-0.0000009405784449 0.0005349061075521 0.9922688792139418
0.5001828572234943 0.1677295737004267 0.9965043409301328

0.1671976390628279 0.0017636328692221 0.9933882305960240
0.8337317458503852 0.3349753561075687 0.9931497710711937
0.9997280070550887 0.8343445874070706 0.9916122691998780
0.6662593628776939 0.1675937948755647 0.9938329183353591
0.3330654785936861 0.0008583536250117 0.9932586813040615
0.1669725926647212 0.8342320043555163 0.9914211948189167
0.8334243741611773 0.1674775892202595 0.9922179276930995
0.5000979000790218 0.0006905244285488 0.9933430605835140
0.3336649142016287 0.8346547096275060 0.9930458014600601
0.6668822662650526 0.0013889753753319 0.9933092152284420
0.4996576059977267 0.8343650774086844 0.9958004229967241
0.8329355512158919 0.0008783579679297 0.9923405873385915
0.6668078356997887 0.8342000906197324 0.9961473372217647
0.8336962417633156 0.8346934567952313 0.9931309234110688
0.5006611262187078 0.6686694919277066 0.0043273079519317
0.5398138616562799 0.4800299770905641 0.1335211925658865
0.5251331168063612 0.5608769391370148 0.2574175083677132
0.5440591466587618 0.3995202353036359 0.2616277471710758
0.3591352444907360 0.3791852556735305 0.2700728429400981
0.7022519964527177 0.5763371225796293 0.2185324397498880
0.3419459063608629 0.3733802175760856 0.1313695362007875

PT6-XV - ΔE = 0.60 eV

1.00000000000000
14.7772044699999991 0.0000000000000000 0.0000000000000000
-7.3886022400000000 12.7974344700000007 0.0000000000000000
0.0000000000000000 0.0000000000000000 18.0009597799999987
C Pt
72 6
Direct
0.8919451656094139 0.6101382842466307 0.9910886880900520
0.0582080871623063 0.6104432629862018 0.9913074596603408
0.2252586394343155 0.6111901698750088 0.9979833580678488
0.8924341660210331 0.4441403832542363 0.9907033118561515
0.0587585873609981 0.4436630020539454 0.9940293307666863
0.3925682357621696 0.6122778123939554 0.0133502132611056
0.5599268469678336 0.6124029784953464 0.0044450663121793
0.2234550638072719 0.4431707545960748 0.0058601365895967
0.8920016170163763 0.2772400938222290 0.9919220438717389
0.0589489125603322 0.2774986752253454 0.9968860914946305
0.3922855832567151 0.4421428211680052 0.0194418031283021
0.7259370945785619 0.6109153828895515 0.9955461341311604
0.5597679270679663 0.4430829782941572 0.0015638166711796
0.2252892311285706 0.2765673627091019 0.0025701777323448
0.8921653431644501 0.1104105425416790 0.9924561093611288
0.7252130742190204 0.4436619014515983 0.9925991309821001
0.3916035609855513 0.2767013863541479 0.9998361901449400
0.0584783373988174 0.1105945815750857 0.9945105348190708
0.8925044036957743 0.9443733154134009 0.9919323990918798
0.5591385022045472 0.2775934104083504 0.9935450741828262
0.2257245822780973 0.1108722468834600 0.9948115879227188
0.0587806615478854 0.9437785541177135 0.9917241099162766
0.7255268190305983 0.2768946754326933 0.9909827473172399
0.3921970569123445 0.1103533841767970 0.9924945566604677
0.2251959469302136 0.9440529976507506 0.9913305020222865
0.8917890746672157 0.7773518774683055 0.9917544072369893
0.5585033656574510 0.1105102747120625 0.9913319414558543
0.3924528748619949 0.9444125747579974 0.9917751520198124
0.0589982153603614 0.7775830263548684 0.9906531810180397
0.7257737938454909 0.1107959975388226 0.9912761679756557
0.5586312520039414 0.9433540920775911 0.9930914819700050
0.2253740919621094 0.7771837944791784 0.9922728271247507
0.7253384090309325 0.9440827986812721 0.9925313434176033
0.3917239620417163 0.7777145086048805 0.9967417484777314
0.5595057644491845 0.7777268618153883 0.9979876163873556
0.7256975047763135 0.7774268866390841 0.9945772018186361
0.0029205124462806 0.6657078025695853 0.9905718086336890
0.1700410634351712 0.6664184116351919 0.9931860363653016
0.3358285180924696 0.6670805027038966 0.0021034439665470
0.0027778449200042 0.4994816581095307 0.9914612612307607
0.1687940106813173 0.4987892767978508 0.9985661947244111
0.6702617975122322 0.6667689885324108 0.9975853911452930
0.3345640421227287 0.4991475614228804 0.0178110498980004
0.0032315007096386 0.3327743640301577 0.9942152462967044
0.1689160571091648 0.3323137258850224 0.0023528063970915
0.5050117891103625 0.4997174182248614 0.0139830094716089
0.8357703918656725 0.6658618261122342 0.9924861013613366
0.6700286282903676 0.4988315167784165 0.9959701639181233
0.0030792749892896 0.1656220382752736 0.9945345904256087
0.3358200531119309 0.3306743787573083 0.0067887823023298
0.1701456083136321 0.1662912515716144 0.9969977624637920
0.5037955592046117 0.3328159056084488 0.9980895961535126
0.8368631701425500 0.4996936617436845 0.9910311251857493
0.3362214062490878 0.1660518797115316 0.9951673332648809
0.6696686744584852 0.3326371026303860 0.9922009847224800
0.0030435790896135 0.9994608090938968 0.9923772390297856
0.5030294010933929 0.1655921541407679 0.9921737045350483
0.1698191484216522 0.9992018948291914 0.9922348789657960
0.8364886066995183 0.3323608941575458 0.9909212660707851
0.0033194953438667 0.8330004413572425 0.9909521103990429
0.6700907126052229 0.1662219082059408 0.9910530497969958
0.3366490744334314 0.9997668675070841 0.9914635581404525
0.1694169010319371 0.8327351937520291 0.9909556643977879
0.8362717181223402 0.1659058630139469 0.9917891717965972
0.5026925517967413 0.9992611992244775 0.9918862139369793
0.3362525801121734 0.8325940770430691 0.9931813472767672
0.6699061953625431 0.9990273635377207 0.9921623313361394
0.5034959924116791 0.8332462243059098 0.9961046455463816
0.8369016827902414 0.9996662581603104 0.9920012465322188
0.6699616718720520 0.8329147142231719 0.9947410839596529
0.8366471792006038 0.8325873202732126 0.9923295523762974
0.5048900657599802 0.6671035461498320 0.0046242676065447
0.5612667632027382 0.4323428194410752 0.2218223624333220
0.4165067806700975 0.6538702385055544 0.2549831868157924
0.3435840145940858 0.5607927536620352 0.1281160244209758
0.2629774812385222 0.4714896613729209 0.2537042681494839

0.4465443592893337 0.4974011732710082 0.2661162596498912
0.4596635644365890 0.4772831262112618 0.1301635593567667

PT6-XVI - ΔE = 0.63 eV
1.00000000000000
14.7772044699999991 0.0000000000000000 0.0000000000000000
-7.3886022400000000 12.7974344700000007 0.0000000000000000
0.0000000000000000 0.0000000000000000 18.0009597799999987
C Pt
72 6
Direct
0.8985559687932725 0.6211925655504458 0.9899855666466952
0.0650572139322814 0.6212496712880051 0.9918613212034347
0.2315107463198443 0.6215681067097739 0.9970293197003497
0.8982618373222673 0.4543046791611440 0.9902573340487408
0.0648272611766777 0.4547025196243599 0.9963352603279558
0.3985331722825552 0.6211998454025822 -0.0013294970326208
0.5657313434609257 0.6213738085326916 0.9956554274546561
0.2316586201181743 0.4559811223226409 0.0123174703264012
0.8983638091590511 0.2876078402620220 0.9924179061881404
0.0634901889471835 0.2869602345777000 0.0024008885293424
0.4005072803663880 0.4564142479148606 0.0125328709542899
0.7319804028845576 0.6213093197136903 0.9911866999880318
0.5657717294398890 0.4547532547883977 0.9955664998816384
0.2308901063498916 0.2856383048234541 0.0082454026020321
0.8980631432967912 0.1208420167063289 0.9938284769419934
0.7317788801211341 0.4542476090033280 0.9901249083236338
0.3992927262297965 0.2867799132033495 0.0021001172570917
0.0645269565267604 0.1207807567469518 0.9983469502366766
0.8983244637451803 0.9545532338695936 0.9929090498614794
0.5649995563676752 0.2876861758824099 0.9923531791917638
0.2320724039725429 0.1208864037496943 0.9982991547663164
0.0652478866899759 0.9546130791390179 0.9940150427029156
0.7317397246550628 0.2876063328501040 0.9898115397753450
0.3987911599148541 0.1208675079254781 0.9938320551686355
0.2319813228969594 0.9545343741694641 0.9932053708726680
0.8984918940777388 0.7879252993474997 0.9911638583927664
0.5650231016374109 0.1207901953684263 0.9908660494070453
0.3984684289990258 0.9543086129112609 0.9918576191025608
0.0652613198504397 0.7880598371295460 0.9914400117943821
0.7315965559570365 0.1208419534631723 0.9907134293913491
0.5649213136205489 0.9538884566713883 0.9912672015278541
0.2318662045612558 0.7880179098484750 0.9922854522202503
0.7315333697405749 0.9541259014190810 0.9913970280368843
0.3980790522612544 0.7873965925806006 0.9936174860719669
0.5650158868612890 0.7873739379866893 0.9930970534583172
0.7320304812341307 0.7879217751576428 0.9915186822214106
0.0098591651415835 0.6773972847544858 0.9906903418113682
0.1763085764330166 0.6774235049276535 0.9932204635351487
0.3428392014493304 0.6774875583159029 0.9965220699165661
0.0098437573989974 0.5106099010367869 0.9921468339641488
0.1756482590022123 0.5113475622846598 0.0014928227268231
0.6770509384055168 0.6773656335358962 0.9921585167408195
0.3444155088900407 0.5127491577255296 0.0062032686063722
0.0092736476984847 0.3435202207634099 0.9966214792060115
0.1748834637540077 0.3424752664032821 0.0131048350197012
0.5116719920891709 0.5115418354306768 0.0002751302490743
0.8433540200912442 0.6774344996048257 0.9903812682250269
0.6764376459511555 0.5105198931183547 0.9916227738944621
0.0091242738040465 0.1767072030312835 0.9982730855041075
0.3434599034426504 0.3421999323749605 0.0139352724669829
0.1765344659109954 0.1770858578109630 0.0011468727299575
0.5101266294550160 0.3434573866337909 0.9962446348819938
0.8432594102136256 0.5106257731093234 0.9896062370728568
0.3435205346186037 0.1768792110412735 0.9979946481270654
0.6766388103199813 0.3439676218074191 0.9903206205402260
0.0098446442942607 0.0108605293176128 0.9949725489701133
0.5097789498351508 0.1768955325396528 0.9922514691736922
0.1767680210291740 0.0108587854344771 0.9950610364720636
0.8430864096723293 0.3439147833389360 0.9903376487261195
0.0101436397020796 0.8443489456991002 0.9919346552345220
0.6764954382900870 0.1770353316206003 0.9902020905976584
0.3432170383681178 0.0103117691779502 0.9927577765093649
0.1764956362293215 0.8441282379986943 0.9920285686728549
0.8429610094404658 0.1769731335389807 0.9921607364941502
0.5096808298461926 0.0102977583987808 0.9912271299775355
0.3428658188684210 0.8437001683666120 0.9925000771696347
0.6763513000422191 0.0101722840645367 0.9909819193393946
0.5097527488379437 0.8436205496531246 0.9925790422511525
0.8430358301973359 0.0102484768448297 0.9924370051757171
0.6765929915890393 0.8437041681240788 0.9918198304190411
0.8433091338380483 0.8439354572059279 0.9915653128665770
0.5102819466880741 0.6773508408985389 0.9957782789176320
0.3758321522317901 0.4036598195014999 0.1272134876277629
0.4287564147034756 0.5127848692977836 0.2486478415677069
0.3328851787776554 0.3145352684565563 0.2563744605058110
0.2023219910263624 0.4018726459219295 0.1275072284953206
0.2272162779447309 0.4111840262938650 0.2649041612349359
0.5252084010907410 0.4135176666815771 0.2087469330492283

PT6-XVII - ΔE = 0.64 eV
1.00000000000000
14.7772044699999991 0.0000000000000000 0.0000000000000000
-7.3886022400000000 12.7974344700000007 0.0000000000000000
0.0000000000000000 0.0000000000000000 18.0009597799999987
C Pt
72 6
Direct
0.2248684315137339 0.2844946503093198 0.0040933077587155
0.0594891009567391 0.1183851266704394 0.9966990671100717
0.8929898930608431 0.9517779894814993 0.9945326883448899
0.0598088957988736 0.2851173233020461 0.9950822440654221
0.8930921294429766 0.1184795288095728 0.9938010333575917
0.7266211106436913 0.7851537893272251 0.9951311254266670
0.5599502810326769 0.6187067295825770 0.9991216922579085
0.7263828280335218 0.9514437907269979 0.9937065861931771

0.8933230917852271 0.2850921391789996 0.9928131519055867
0.7264272284435336 0.1182636355563460 0.9935859731955148
0.5595095654869624 0.7848614192787338 0.9948315532439906
0.3929175551967319 0.4536168874390896 0.0135199039900868
0.3929759281506691 0.6190245035341135 0.9968884059391954
0.5599280436677461 0.9518104700535446 0.9940824610694463
0.7266534303906127 0.2852026064714650 0.9948615799280276
0.2264214410854493 0.4522777898847892 0.9972604735993826
0.3932946582406274 0.7854396660665657 0.9940598606160691
0.5602405492994507 0.1183163746049800 0.9964091596035161
0.5607052518744311 0.2843934034181217 0.0038464295364733
0.2265169602018915 0.6187880600632525 0.9939389589647547
0.3933989964404887 0.9519985732847474 0.9958716254728515
0.3936034341556649 0.1184478883509783 0.0006812381906869
0.0598210223216569 0.4519414993115873 0.9930661558185534
0.2267467166573525 0.7856406657754533 0.9945067447903284
0.2267090481798064 0.9521055125445770 0.9971367555840587
0.3922264337437369 0.2832508875793280 0.0093611962373359
0.0600613012480053 0.6187167698695220 0.9934400757131101
0.0599112600308800 0.7855016201955252 0.9948736183465599
0.2259610813144306 0.1183169964433901 0.0008987653584853
0.8932803307700823 0.4517191691563411 0.9932698977134606
0.8935646277779767 0.6189966725256895 0.9949303086137036
0.0598511719296084 0.9522119516391570 0.9962471970771887
0.7274962069444157 0.4520854726185135 0.9983194946233027
0.8935292679761702 0.7855414293379681 0.9950548283022229
0.7272238218350395 0.6191633265473868 0.9986563980937646
0.5614369899532790 0.4536950649424298 0.0117132318836752
0.1705068016367974 0.1742171495302074 0.0005676437735573
0.0044738004986442 0.0078516325989568 0.9956554564234494
0.8379969022873510 0.8413129626745999 0.9948248075780128
0.0043402956615424 0.1745187034023417 0.9951517649252679
0.8377409401645366 0.0076523345495971 0.9939022341605650
0.5055915691328039 0.5102475820541486 0.0061602870806539
0.6712386281017757 0.8411674363359722 0.9944001870754704
0.8379807082855351 0.1745549913205338 0.9931796882741750
0.6710591255516860 0.0077851849900483 0.9936837549665896
0.5042769209968014 0.6750080221367583 0.9968672939830654
0.3362328290408669 0.3399148457861187 0.0146636754928693
0.3370616767155923 0.5089111417212919 0.0012878669275622
0.6712312122895838 0.1742655134568096 0.9948234072337172
0.5042585223268787 0.8410929281991386 0.9941582581652852
0.5046368894335629 0.0077662153507987 0.9952666113401065
0.3377246303143622 0.6748882224315764 0.9943213986158597
0.1708139432570306 0.3410164308181862 0.9983828570578623
0.3378591930941681 0.8415522853738580 0.9946058046076374
0.1712766636309198 0.5081534346221233 0.9940468755945560
0.5049046984515329 0.1743004458284148 0.0003640624101493
0.1712147759409532 0.6748345103173961 0.9937071220415348
0.3381927227965846 0.0082367526596911 0.9978251535724567
0.0045129218116884 0.3413037133177284 0.9932312728182708
0.3379225613962475 0.1745851060859778 0.0032561757194642
0.0047916137815136 0.5081484809066268 0.9928735290848607
0.1715753107986302 0.8418853617504283 0.9953442591686539
0.1712824888183277 0.0084115238741802 0.9980345912176958
0.8381382785863707 0.3414885165988366 0.9931749988380503
0.0047079653623925 0.6749718661963513 0.9941459667663324
0.0047711823474970 0.8414851908193199 0.9952002674643978
0.8379302864160110 0.5079147126443360 0.9949206258371603
0.8385646776537143 0.6750199461617328 0.9958439292124552
0.6718060081105364 0.3409881195192810 0.9983812475957095
0.6728708206448815 0.5089085956205395 0.0031000097981249
0.5047776042033263 0.3398682563710480 0.0136779267921625
0.6717476618057034 0.6747968213793527 0.9973306955518524
0.5837679879376978 0.4855481579925822 0.2577145708084529
0.5454198024047159 0.4046042867516562 0.1267693206491742
0.2485045600411411 0.4526267363005445 0.1944046701750466
0.4231597734913919 0.3040363267391726 0.2380562100772821
0.4009330506304778 0.4651998090582552 0.2606207457930765
0.3682751935365758 0.4010038617813692 0.1287246350401787

PT6-XVIII - ΔE = 0.65 eV

1.00000000000000
14.7772044699999991 0.0000000000000000 0.0000000000000000
-7.3886022400000000 12.7974344700000007 0.0000000000000000
0.0000000000000000 0.0000000000000000 18.0009597799999987
C Pt
72 6
Direct
0.2236058460661923 0.2758868973869824 0.9969202365068952
0.0567644592233307 0.1099522488308082 0.9926091976101077
0.8909402697781275 0.9437930673498158 0.9933224314705953
0.0579081037404165 0.2771834191850147 0.9948584372595519
0.8906627997476093 0.1098113748158411 0.9931910511686226
0.7247084202950020 0.7768680454638925 0.0002736946045516
0.5592014458079007 0.6120161387380634 0.0206536538533229
0.7236094927304251 0.9434263363736406 0.9959801959929564
0.8903120177903290 0.2767875979411656 0.9939015643792146
0.7242607939455041 0.1103784007728450 0.9938570536312454
0.5579177877778889 0.7783694845731830 0.0047089371286546
0.3887148931702029 0.4416097205344585 0.0160183774724842
0.3887233332303808 0.6092350964931867 0.0060305018343740
0.5573123605570558 0.9434198723338625 0.9958653594831688
0.7237970329219010 0.2764706327464310 0.9947208518537067
0.2233814289129938 0.4435481077928713 0.9997382567288540
0.3901731676666671 0.7769331283702101 0.9979752820505610
0.5570066311619755 0.1102820551910568 0.9935057037491362
0.5575007637456650 0.2770211095415647 0.9963861069802533
0.2237750831423924 0.6101282305213402 0.9969200785290937
0.3909975500814724 0.9437274037776717 0.9936160151740410
0.3906077357195699 0.1096522997685483 0.9930086114886123
0.0569997088653693 0.4430130867118507 0.9949976033391010
0.2237381663562772 0.7762501489101652 0.9939792524499182
0.2235741808194618 0.9433086007862883 0.9921844945207638
0.3898822740278121 0.2761823128527894 0.9983895328816033
0.0567564839426780 0.6099109704218151 0.9943662909598316

0.0574759888542644 0.7768361659274206 0.9933798739679176
0.2240919473995717 0.1101427270971485 0.9928795771461765
0.8906799043816775 0.4435843672019502 0.9950041168382810
0.8905566393063751 0.6096509653311770 0.9964201571191822
0.0571525662829160 0.9429657633612294 0.9921165186025931
0.7234615006408802 0.4433522837108441 0.9990511479968660
0.8903981932548035 0.7767061713431218 0.9955726874627899
0.7247453668855357 0.6107039032478312 0.0039335179030715
0.5576845337850358 0.4419262543523956 0.0083621849523396
0.1685027306093474 0.1656829946277512 0.9935625033119564
0.0012845838252318 0.9988738491014288 0.9922098576277207
0.8350910605907274 0.8320838860154041 0.9955670760050950
0.0017790637017114 0.1653302985166718 0.9932807695545094
0.8355259390187157 0.9994343699497605 0.9937625339491163
0.5014015173675759 0.4968658954155600 0.0218218465414068
0.6685585860320984 0.8330755835743062 0.0004628427846001
0.8346945050438421 0.1657206930394608 0.9935861518209776
0.6683719964321995 0.9989637795163802 0.9951910680873084
0.5011606848284273 0.6668112744343802 0.0142513546506748
0.3339865933856672 0.3303744805384383 0.0020736418688401
0.3337791588027983 0.4995127396883490 0.0063738366723740
0.6685409962278470 0.1660904113274100 0.9938795530050797
0.5018873615304713 0.8332873001404622 0.9992372671653342
0.5012143847597486 0.9991438803158843 0.9939479764703663
0.3341906806317070 0.6654492345777072 0.9997337666697419
0.1677144231599144 0.3320946709688499 0.9974789961509032
0.3350765638148800 0.8319647388729976 0.9948782736006763
0.1680915001016743 0.4988897552973839 0.9969263954663319
0.5015839740214858 0.1653624523644694 0.9937682549252855
0.1684177277825469 0.6660035073416921 0.9947406625440252
0.3352437996047328 0.9992955250023101 0.9925271206127420
0.0019509180413095 0.3327853475699172 0.9945365776736992
0.3345907621328479 0.1656257044222684 0.9939090758678972
0.0012324753187798 0.4990694487007588 0.9945939080921349
0.1679462489198329 0.8322331775649729 0.9927975903930530
0.1682906351290470 0.9985846714356725 0.9919411741353130
0.8350036742604630 0.3321836585782805 0.9945279008087411
0.0015983117255161 0.6654327266880244 0.9944354867954317
0.0018372733326117 0.8325482480381652 0.9932637400835773
0.8353031477200332 0.4994190616636800 0.9967311380742885
0.8347321158892527 0.6657708727575482 0.9983790762474740
0.6679744111259893 0.3323304679668482 0.9967658106452433
0.6684089611643103 0.4984172292651294 0.0044511549017940
0.5021169013101314 0.3323697768492408 0.0006683245191539
0.6703453988329358 0.6670242213430768 0.0072633987177184
0.3969415038549045 0.4668763841679606 0.2688597897691594
0.4869524731149042 0.6685817421851752 0.2666862197906354
0.5385293720359883 0.6596513876859760 0.1300869873992525
0.5923469144213982 0.5793600898759408 0.2378390689159886
0.3009123130735603 0.2978523374700259 0.2033485603658391
0.4311809868220777 0.4601131628535242 0.1325716324076609

PT6-XIX - ΔE = 0.68 eV

1.00000000000000
14.7772044699999991 0.0000000000000000 0.0000000000000000
-7.3886022400000000 12.7974344700000007 0.0000000000000000
0.0000000000000000 0.0000000000000000 18.0009597799999987
C Pt
72 6
Direct
0.2192263277050372 0.2795849711951064 0.9979245478098946
0.0528888117807327 0.1127584428819546 0.9961672772254744
0.8862032715280890 0.9464456614938612 0.9964962675566156
0.0524093544770281 0.2791577959227283 0.0019891625233441
0.8863965698788405 0.1130283516598283 0.9966869367790991
0.7202857709400021 0.7802524010789256 0.0008118495037976
0.5532796602092915 0.6147972342292505 0.0129951042644478
0.7195518300730726 0.9458213975314180 0.9942009537564900
0.8863623038076796 0.2792387350390513 0.9992030041581756
0.7194044000288073 0.1127728508526218 0.9932893591696867
0.5527680581612628 0.7800240865666268 0.9937899594900728
0.3849420193733764 0.4441571647673754 0.0027227735516036
0.3868982173669764 0.6148809924744612 0.9974209529627629
0.5532571102886266 0.9465823215728406 0.9890514223067797
0.7200026469028700 0.2797495216179087 0.9966792635597699
0.2183324342674169 0.4455786126539394 0.0101576443845772
0.3862096525046610 0.7795305511168493 0.9890232501378975
0.5531312365191852 0.1125776354919381 0.9893385194125628
0.5524680687731944 0.2787486813937292 0.9946855395996437
0.2187198189683457 0.6129429434570071 0.9957017598290392
0.3859885650498995 0.9462904140114006 0.9870925450903911
0.3865050438588256 0.1131994179474631 0.9885700307343086
0.0529469362874551 0.4465684464681203 0.0016688227019444
0.2197392420846782 0.7799651405823695 0.9901755814403614
0.2195863218757594 0.9461780647368414 0.9893711097254325
0.3863931143407839 0.2789556775518349 0.9943912919072631
0.0526379429196950 0.6125559020934546 0.9971940625730156
0.0526606906073610 0.7797119644167765 0.9947787379502415
0.2192534935071606 0.1129283925120603 0.9913093608797467
0.8860819143882637 0.4460630986694341 0.0005170901517602
0.8867555874131057 0.6133023168908380 0.0019181105468675
0.0531998727537442 0.9467253803251268 0.9937820113024856
0.7204642119072204 0.4455022483503086 0.0055861076644490
0.8865535274953515 0.7798327575526542 0.9995191113483628
0.7206366801911770 0.6145535879936048 0.0086931566871726
0.5529207259348183 0.4436704284646069 0.0153148962446608
0.1638770778145542 0.1682735028323137 0.9946930591296876
0.9977580761503191 0.0021049418129451 0.9953632769366152
0.8309451833105186 0.8354540298378339 0.9988604063592348
0.9970368488997394 0.1681933331152692 0.9983377932505775
0.8307779719440589 0.0015103746588139 0.9957068643783629
0.4960009030238695 0.5012514806676478 0.0152965233080309
0.6645303520173940 0.8357340956603636 0.9961539648581095
0.8309221823170745 0.1685650288766709 0.9966007135758019
0.6637599553992501 0.0017252988083385 0.9916785301710007
0.4971482625106844 0.6698677075465653 0.9989659101226138

0.3303421385415959 0.3350631757794531 0.9973267702348423
0.3305254102044586 0.5019221323280192 0.0082327890271046
0.6639454384705203 0.1680180924495360 0.9926289294163695
0.4972076115228958 0.8350250976660973 0.9895639596124965
0.4975589976861130 0.0020599964277679 0.9877417662629071
0.3310000399009567 0.6691618975721099 0.9926261648309094
0.1634913796250146 0.3341268510673956 0.0044673408283415
0.3302239883641747 0.8352096475845627 0.9880122824282651
0.1629489763927339 0.5023724697626335 0.0027922225625190
0.4971956066856578 0.1683691367376596 0.9896210402146153
0.1637783626488201 0.6681270605689917 0.9936703772295985
0.3305272700769208 0.0015638262565929 0.9876807453285323
0.9971637985941086 0.3347133652811749 0.0019127009357192
0.3309979231818332 0.1686524101913989 0.9904757480972748
0.9975854036273688 0.5022839361460072 0.9996444443613655
0.1641217872063692 0.8353702896703794 0.9909694390398798
0.1639064594724076 0.0020537009431365 0.9910598886306659
0.8305592807021185 0.3350104003358396 0.9991391073118763
0.9968258572003137 0.6684525848105665 0.9978301594157273
0.9974843547826708 0.8350726759380080 0.9958149002371925
0.8312085260512561 0.5015487201776878 0.0028657090586535
0.8310387079941250 0.6691892367097338 0.0032531986626338
0.6645284132477300 0.3351028499927722 0.9992487556854712
0.6654653075004617 0.5021115085163856 0.0144159066377725
0.4965725276756316 0.3329368194462603 0.9989634477270570
0.6654364767720153 0.6696369376551914 0.0077745998015075
0.3899616080074111 0.4240869655640935 0.2260386245500428
0.5010180777048703 0.5511919314845954 0.1277926562749627
0.3041454003999675 0.5042853220208912 0.1254966880262458
0.5982375353846692 0.4475194497353030 0.1289147143813096
0.5168718993454391 0.3663406734492796 0.2494498976708925
0.4123268798536301 0.6045483926060911 0.2365244262550092

PT6-XX - ΔE = 0.68 eV
1.00000000000000
14.7772044699999991 0.0000000000000000 0.0000000000000000
-7.3886022400000000 12.7974344700000007 0.0000000000000000
0.0000000000000000 0.0000000000000000 18.0009597799999987
C Pt
72 6
Direct
0.5610169028586183 0.9518192057081366 0.9895796863672572
0.5607426774499304 0.7852466260418099 0.9933156613236443
0.5621262372592051 0.6204009247268840 0.0006807379598700
0.7277052844449389 0.9517091515910491 0.9893673810532014
0.7274267279324697 0.7851908472900401 0.9907745779764329
0.5619594123621314 0.4513639988861441 0.0103223304910033
0.5604883201209930 0.2842816715482486 0.9981118242650606
0.7272980459932165 0.6178258537562336 0.9949797973163832
0.8937223516883736 0.9512362026020352 0.9902471600969764
0.8940123465608565 0.7846916834244740 0.9898579791876521
0.7278879448561345 0.4514945876139849 0.9976357359795542
0.5610305560011448 0.1181151905353656 0.9911527284559013
0.7274376989687630 0.2851433902499457 0.9938106205809608
0.8940121768853775 0.6181900383484136 0.9925445980891139
0.0605195190031692 0.9513905292482789 0.9915311296682385
0.7276949765531194 0.1184424053837644 0.9904858980073994
0.8938903194434146 0.4513690928588772 0.9944730312695886
0.0605385631829852 0.7845855677900669 0.9901155892861579
0.2277443397396202 0.9512756226086501 0.9903883511798889
0.8945554229637583 0.2851869779334704 0.9946199832076954
0.0607664939950189 0.6181540664597098 0.9941291901568333
0.2276661773094928 0.7851156718928405 0.9912936399570371
0.8944785039853423 0.1184932863710906 0.9926399942879247
0.0603083665547296 0.4518426281175290 0.0008147030659285
0.2270698474205456 0.6186761075547718 0.0009257043921101
0.3940883736076828 0.9512795118201226 0.9896290742019869
0.0606111080751768 0.2847191745556910 0.0014473212375799
0.2255659513598810 0.4515905927438388 0.0191088472332302
0.3943367248714999 0.7847623029244229 0.9934405535492674
0.0602966416689022 0.1173704809918425 -0.0017981538800106
0.2252631866501761 0.2825190374016419 0.0175449694597902
0.3948313089736090 0.6201087991476572 0.0081916025601677
0.2268173660188923 0.1169193026102435 -0.0013080603725183
0.3966255458260814 0.4522919583865182 0.0221277816068703
0.3945033511528949 0.2827484309921128 0.0078598531602907
0.3942116381908313 0.1180252541354521 0.9937168735886264
0.5053135655730198 0.8405599337526015 0.9917865257035529
0.5055589470641833 0.6749868267415239 0.0003451947920685
0.5068720889998867 0.5086465583385906 0.0091975371273203
0.6721528862327109 0.8406052506894910 0.9904240818454645
0.6718229616997063 0.6740066766110521 0.9944323516163890
0.5051948137150315 0.1735592924018408 0.9940010414370331
0.6728450294792749 0.5075819522673842 0.0010648538837391
0.8385047417193756 0.8404602159580672 0.9896069452552029
0.8382785024461621 0.6734122535098931 0.9916244636857656
0.6723482328274039 0.3404543637163148 0.9967474759673705
0.5054308104693123 0.0070875632181639 0.9896515844923087
0.6719823595481712 0.1737335840780636 0.9916367380008496
0.0049395125322060 0.8399745281240911 0.9898197763123278
0.8385776204980298 0.5070960624118026 0.9944160167847715
0.0051721414842942 0.6738985861063689 0.9916645880004211
0.8387433289292484 0.3409044443396115 0.9942145523585133
0.6720981805090314 0.0073781268866903 0.9894477644976000
0.0052243019089754 0.5073729955247256 0.9960033894038430
0.8389782820658773 0.1739792959449339 0.9922849262477733
0.1720304714862328 0.8402768372536723 0.9899062279973050
0.0049930798255015 0.3406900381650780 0.9988530972386328
0.1718708867713868 0.6735292572089442 0.9947407904306119
0.8386236768291918 0.0069087831681544 0.9903437455614161
0.3384443952611199 0.8404775923054251 0.9907926804391005
0.0050730392152192 0.1734394638719097 0.9971060403420522
0.1710078815316971 0.5081572622508419 0.0071972265822940
0.3391155194923934 0.6745366791845366 0.0004637057270888
0.0051230306068852 0.0065489755677149 0.9927224705915506
0.1695494906107015 0.3391971148420982 0.0115011126636625

0.3395210798337172 0.5096309560914192 0.0239799032991072
0.1706406375621210 0.1714059090996970 0.0050000104440037
0.3387205321247247 0.3380625152168097 0.0221597277492126
0.1718332909032064 0.0071026374953789 0.9927622264969898
0.3388816888975388 0.1729328904754705 -0.0007028923357976
0.3383483070112979 0.0065181377945148 0.9905817670556202
0.5050748846798297 0.3386334818458811 0.0052336853892253
0.4930098863940712 0.4766258595406806 0.1246591863836694
0.2733661146803714 0.4788931218059820 0.1361239971328050
0.1880225837925896 0.3518009566855261 0.2475623107498591
0.2687310989476303 0.3053188708984412 0.1346321955239626
0.3825742311937915 0.4491180589178935 0.2461639892504758
0.5689112984950123 0.5083415550772385 0.2523492767817380

PT6-XXI - ΔE = 0.69 eV
1.00000000000000
14.7772044699999991 0.0000000000000000 0.0000000000000000
-7.3886022400000000 12.7974344700000007 0.0000000000000000
0.0000000000000000 0.0000000000000000 18.0009597799999987
C Pt
72 6
Direct
0.8850977484331871 0.6061537002954657 0.9964907094815172
0.0517460258600232 0.6061692158319282 0.9925753099346024
0.2181711347018734 0.6061252569588600 0.9905523691043510
0.8851011620405146 0.4392463240146108 0.9924204039375111
0.0516999125250075 0.4394651135584881 0.9934253446412039
0.3847390296919975 0.6067519532273421 0.9923190979463554
0.5506581262078369 0.6061499649808770 0.0083323203904915
0.2185752477377149 0.4398817116218112 0.9959326492752069
0.8854770811777085 0.2727992415705504 0.9961863536161560
0.0520440744762718 0.2727721877951508 0.0004030292146170
0.3840396400896600 0.4398698170961357 0.0056769967851385
0.7193029092099953 0.6051850600827000 0.0038700771719554
0.5525237920113437 0.4382796669514197 0.9979065485505296
0.2173986582382044 0.2724881468945662 0.0079946743997146
0.8851365180980946 0.1063288131128864 0.0042151633984596
0.7188043086958303 0.4393082490705570 0.9922487938513049
0.3857981401491911 0.2720292634077610 0.0034210247506650
0.0519413631873742 0.1064865419697091 0.0059025895117854
0.8858586950248808 0.9399074248105374 0.0089875821783477
0.5519969961439360 0.2722908222251706 0.9927706457130095
0.2185910852049418 0.1060483502484249 0.0048509738056555
0.0519180802472691 0.9397928323203844 0.0043402855127124
0.7186826913753062 0.2728876230591754 0.9923883808245453
0.3854853695745898 0.1061795764328791 0.9968733505382268
0.2186750839080432 0.9396470870781145 0.9981894321405592
0.8855162390837090 0.7733037442327340 0.0064933021277421
0.5522200516519867 0.1062043067580223 0.9939731245712926
0.3851748250323955 0.9394199625834070 0.9937041970667484
0.0517461242315385 0.7728055157059635 0.9979373796042954
0.7185795700248333 0.1062080531917643 0.9983875756614790
0.5518943095629445 0.9398912580842040 0.9972951960426438
0.2183157443158587 0.7729667851873826 0.9925557079089121
0.7187344873023984 0.9406877769721181 0.0090825426772116
0.3849748506895748 0.7730452328482234 0.9916704779022062
0.5502285676373898 0.7724477555030254 0.0007983568342604
0.7196140885316167 0.7742349003331160 0.0216996204360940
0.9962254637021154 0.6620518808636964 0.9954496579930205
0.1626159164156047 0.6618887415762007 0.9914269763509921
0.3292307445666512 0.6621037259033966 0.9904023757581156
0.9963662545266914 0.4951924357595656 0.9924574780140637
0.1628544835587234 0.4952642918304587 0.9925561714746745
0.6630562202655461 0.6598074853629324 0.0162560044528426
0.3282683237159480 0.4956960300515857 0.9960313098716169
0.9961578624932413 0.3283700648110717 0.9963925770887690
0.1628236083262880 0.3280031425552891 0.0014656526434796
0.4959954311794245 0.4934362971585026 0.0060785949739000
0.8293432929115601 0.6611954728878801 0.0025243721141732
0.6630435011962730 0.4944934454375782 0.9959887235340739
0.9965049015042740 0.1619728318129027 0.0040129812001019
0.3296683255013394 0.3278832809428804 0.0127609655523198
0.1627044280034580 0.1617704512461804 0.0070567218004533
0.4963826562220467 0.3277398451432412 0.9963989770538487
0.8295499628311812 0.4950392056515218 0.9931183785047547
0.3301894319746455 0.1620158060298851 0.0017047025235529
0.6630727872761488 0.3281522762371664 0.9912579329527279
0.9965377453097244 0.9952465005192831 0.0062660197720876
0.4962980354632172 0.1615510998627771 0.9939800504583191
0.1633136849587020 0.9957328618591714 0.0025726829977972
0.8297442478547532 0.3285881305364783 0.9931315036344153
0.9960889179307649 0.8285929993962871 0.0027641618005226
0.6627055027840686 0.1616905854565836 0.9943219888416664
0.3294898456118087 0.9950880492719190 0.9960002326803560
0.1627823755723876 0.8286936598649296 0.9959869722278493
0.8297038883729327 0.1618843423422682 0.9997964318453405
0.4963103205743500 0.9949309194739392 0.9945693644296417
0.3293275002895578 0.8286329249044506 0.9921294968981246
0.6628322202346318 0.9957937281058737 0.0015357605720490
0.4956806257867541 0.8283734388659948 0.9957857050426561
0.8298355548626334 0.9956752544263996 0.0085543683143895
0.6618908538225683 0.8293226801277903 0.0138729316433318
0.8314037988745128 0.8295504251348419 0.0123503027448324
0.4950579788248523 0.6626022644169328 -0.0023525359000124
0.3971175082126678 0.4232086084110045 0.1222098474222715
0.5795902115347336 0.6059680622133671 0.1229228982348459
0.4641862459976590 0.5418770736155128 0.2398352244995011
0.6448905618514951 0.6980077337427288 0.2532496797518115
0.3091288739086526 0.3660688205521385 0.2458191037750307
0.6801638293663645 0.8015304381605776 0.1323073835633168

PT6-XXII - ΔE = 0.69 eV
1.00000000000000
14.7772044699999991 0.0000000000000000 0.0000000000000000
-7.3886022400000000 12.7974344700000007 0.0000000000000000
0.0000000000000000 0.0000000000000000 18.0009597799999987

C Pt
72 6
Direct
0.5591658891449158 0.9447619598824705 0.9940488005077802
0.5582755462756110 0.7779317557123484 0.0035202519008862
0.5568839097664128 0.6117802928581936 0.0201646799226809
0.7256859226561869 0.9446167412796548 0.0019481980818950
0.7266898014830916 0.7800751246565913 0.0138776989337501
0.5596654361056949 0.4435793932488822 0.0014683335774085
0.5591334688362626 0.2778852049546444 0.9909556190771016
0.7265745350407593 0.6105028743722568 0.0168696426979602
0.8922836759173138 0.9450289296937709 0.0057396847461596
0.8928022013481421 0.7783787296290977 0.0088995043332328
0.7254381862647408 0.4442821354486874 0.9967365979241504
0.5589712605963620 0.1112132084138848 0.9897721655721690
0.7257343606896650 0.2779621260997521 0.9909090157625092
0.8928795600225349 0.6114226264833923 0.0018017139377297
0.0590125837946809 0.9450015093501558 0.0032698093792886
0.7257849059410438 0.1113207489962074 0.9941271804455849
0.8920436214135700 0.4449013510979906 0.9939487032046365
0.0589444547948409 0.7782440695363604 0.9989681996361953
0.2254506291881936 0.9447136842161091 0.9952700328748244
0.8922272599117401 0.2780375844332395 0.9948484005075678
0.0588062867445786 0.6112696884991652 0.9937586861781506
0.2256929107942017 0.7782834940973155 0.9920850081545192
0.8927733744414569 0.1118155901385689 0.0014506926782165
0.0587028649352601 0.4448521236129230 0.9941004677787952
0.2252815414338080 0.6115604781182941 0.9918465140776788
0.3922819695023779 0.9449143708509719 0.9908564308695731
0.0590458509835799 0.2786719429985733 0.0022939069856834
0.2248369390833272 0.4448979700103647 0.9991247593949331
0.3921726885540713 0.7780585608791153 0.9928818949980217
0.0587304228114760 0.1111388943346933 0.0070504380365965
0.2246625908287798 0.2779351773125774 0.0155756769208111
0.3915600810952762 0.6115753374032724 0.9987978090702070
0.2250116251580717 0.1113075785648528 0.0020327252550993
0.3903712219873296 0.4444563423870122 0.0109567955889610
0.3927424944739149 0.2768395694146005 0.9991233158406476
0.3923325083468521 0.1114728333638036 0.9921438028837385
0.5032777668040365 0.8336053697467090 0.9961788168338305
0.5022442529122959 0.6679000474436684 0.0066492217713511
0.5032070908461498 0.4991709358554077 0.0131164354808109
0.6704395126387954 0.8341459841848241 0.0070433837210017
0.6714989133410781 0.6684197020403357 0.0248402142451512
0.5031703443974237 0.1668002533314494 0.9898877683628821
0.6695489080152556 0.4987617395509238 0.0036247786938157
0.8369964109821203 0.8344067606331983 0.0094563289856672
0.8378340384511503 0.6672890554348282 0.0102654212228141
0.6701139522847387 0.3336309349662465 0.9916401049756093
0.5032872479349737 0.0002999212177065 0.9908092121794947
0.6700610427427800 0.1668965157346941 0.9907855340834111
0.0033836881580146 0.8336341999404251 0.0035991869681032
0.8369832254798860 0.5004518105915337 0.9974403438125737
0.0032691259190714 0.6671378528858146 0.9978666676513074
0.8365400388088229 0.3337616212145771 0.9925720459973724
0.6699132489813062 0.0002807104613356 0.9964252499910635
0.0033521487562668 0.5007761920762603 0.9935157167069014
0.8367450917973177 0.1672013297440272 0.9966888840284669
0.1698847819294483 0.8340251207085517 0.9949834633390829
0.0032997061206605 0.3339847433064023 0.9966877864069090
0.1699974777936425 0.6671923248981747 0.9919049565986312
0.8368635715079413 0.0004193364156849 0.0033929530239497
0.3363607947138016 0.8336291433127611 0.9912528692057824
0.0030545238985991 0.1670603050015677 0.0046207405733022
0.1694459320778520 0.5005119907734409 0.9939560778253043
0.3364473690789010 0.6672854315591074 0.9933071030307448
0.0035225233817129 0.0004174005590602 0.0052784243140724
0.1690533921157405 0.3342360243330560 0.0057613883300875
0.3353180822952425 0.5010072444696229 0.0017761785115228
0.1694339107488325 0.1658160989393047 0.0091997622756544
0.3368159113568138 0.3324145268143102 0.0102374080708986
0.1698078561110243 0.0005667764004289 0.9997840577321560
0.3369613848773372 0.1671585558185592 0.9970396752983156
0.3365454745624340 0.0002660829306512 0.9921769462274490
0.5038072021794796 0.3333453103633009 0.9949703376993441
0.4407216962374620 0.4137992311391419 0.2451917928342934
0.5035522396552262 0.5282379899440052 0.1276985705165230
0.3206495247995095 0.3445680893820240 0.1273525771997812
0.6992090941577020 0.6298809437821867 0.1357112067916810
0.5980392537907209 0.5938381125760307 0.2581023438274275
0.2653320669350690 0.2590701271462984 0.2525859961040538

PT6-XXIII - ΔE = 0.71 eV
1.00000000000000
14.7772044699999991 0.0000000000000000 0.0000000000000000
-7.3886022400000000 12.7974344700000007 0.0000000000000000
0.0000000000000000 0.0000000000000000 18.0009597799999987
C Pt
72 6
Direct
0.2228650706626709 0.2760620803624789 0.9916514764267248
0.0556633637163557 0.1094052089461570 0.9874488414337250
0.8889685002082217 0.9430723560485035 0.9894092206903977
0.0557788176666634 0.2765176054770180 0.9890478107923002
0.8893095737068677 0.1097146913599829 0.9883033433910384
0.7225914202337975 0.7765672315640231 0.9952724578761050
0.5557534703153664 0.6096586830861952 0.0017865613758516
0.7221503273703789 0.9427049763254089 0.9905962867056957
0.8887677100370652 0.2759754629107434 0.9906789968750331
0.7222494026011574 0.1099691207111562 0.9915518167505226
0.5555794669144234 0.7765434627219676 0.9945248303077463
0.3884278771891516 0.4422973377203547 0.0085568293126883
0.3896017073645410 0.6111674790742612 0.0012800069754277
0.5560711051094458 0.9433056154898196 0.9929313614989113
0.7225202147788892 0.2765317283179201 0.9966375048801197
0.2208466320897458 0.4421796900549211 0.9976152110319703

0.3891180838454602 0.7764955051752125 0.9949213552581213
0.5556249835019358 0.1095961052974772 0.9956430698044372
0.5549356829660681 0.2761385241615599 0.0052996386859050
0.2221863597205882 0.6098132981151565 0.9959089177215432
0.3890982429765302 0.9432718124148849 0.9935519731283406
0.3892260722247727 0.1095749136830761 0.9957902140302863
0.0555370556852282 0.4428681255056901 0.9921944366283100
0.2227351567125879 0.7764940447466913 0.9930478940218848
0.2224554057930348 0.9428443626256495 0.9911479499799256
0.3886577103507349 0.2753457891093944 0.0002994417890605
0.0556336165257605 0.6092231500651764 0.9932392004693043
0.0559143593484492 0.7766153053343743 0.9920138153592788
0.2225705529815869 0.1097640486979401 0.9901859036152985
0.8888696412624739 0.4429071260264692 0.9941120955040432
0.8899626515662931 0.6099129725317667 0.9979736846869116
0.0562808021841192 0.9433348434868876 0.9890105270273963
0.7232241584823598 0.4418140785620679 0.0059511871822693
0.8896570162447830 0.7768706992478513 0.9940613920961994
0.7238319334232912 0.6119613533049417 0.0091354714007537
0.5539600039496761 0.4418521816972287 0.0242842032775243
0.1674369067115364 0.1653339940273000 0.9888188531156672
0.0007884282770121 0.9991091391352356 0.9881754086642331
0.8338525810524118 0.8322526421908378 0.9927453963352164
0.0001842966492518 0.1652100749559793 0.9876490252205272
0.8338840174180575 0.9986866468963242 0.9890967139295057
0.5015225888629260 0.5003757474895991 0.0094382046356500
0.6671412777633208 0.8326146476590637 0.9927928676694577
0.8337617367068546 0.1657008965984872 0.9898581267563742
0.6666637232666730 0.9986661820439409 0.9913590014204985
0.5001597464865597 0.6657038927104750 0.9982380208114208
0.3326437908060811 0.3300499103833729 0.9984146863099497
0.3328762383337391 0.4993479281424342 0.0065656939438554
0.6671066830416450 0.1653677279829893 0.9946545221769725
0.5004978452439346 0.8322479204457167 0.9940300540134501
0.5005399290632795 0.9993105884459652 0.9939399042869752
0.3340727471079958 0.6662421109071630 0.9974006776249027
0.1668729821421806 0.3323265083348730 0.9919875352456202
0.3335475952591480 0.8321944015026261 0.9935438801561816
0.1664172187060871 0.4986038325536200 0.9952637557583870
0.5000908084829874 0.1655346009993650 0.9986969993069366
0.1673224048977104 0.6652944942155656 0.9937538139912121
0.3339041911281910 0.9986994950350194 0.9931656400390168
0.0004053229574339 0.3320675795080419 0.9902997766064416
0.3338021428208435 0.1652035405360976 0.9947177162123637
0.0004792329655956 0.4990422467908218 0.9926594579949466
0.1672796196046420 0.8322941599067661 0.9916098654262839
0.1671610961436016 0.9986938042254820 0.9895352758802147
0.8333689388423124 0.3319042294069163 0.9936535303853233
0.0002900461555129 0.6652833599450076 0.9939579200328481
0.0010895654662804 0.8324048639415125 0.9911151531561515
0.8339943373936975 0.4982919828534449 0.9986203524484836
0.8353946775037784 0.6669241203800027 0.0001362074957285
0.6675717311097600 0.3324232961633484 0.0032368727413896
0.6692959442002504 0.4993865811203548 0.0207538880090894
0.4994247991336920 0.3304654487597816 0.0107912144116824
0.6672343461882306 0.6667029375265798 0.0020352727045534
0.7196571550084911 0.5657377509251660 0.1265734087301027
0.3565274348837733 0.4544869807433045 0.1217045980905382
0.5255125970701400 0.4471273276263261 0.1532324193447181
0.3530792864421599 0.3597851172362425 0.2361306929150899
0.5253435305084935 0.4703443397685376 0.2955324724792661
0.6731142166043682 0.4568532574870900 0.2423477732234289

PT6-XXIV - ΔE = 0.72 eV

1.00000000000000

14.7772044699999991 0.0000000000000000 0.0000000000000000
-7.3886022400000000 12.7974344700000007 0.0000000000000000
0.0000000000000000 0.0000000000000000 18.0009597799999987

C Pt

72 6

Direct

0.2196448612630775 0.2746544102118733 0.0000362309367006
0.0547425629350613 0.1089191428444991 0.9928550149613926
0.8878481750687897 0.9419385304588062 0.9899615509796362
0.0545182273617542 0.2751949633144832 0.9884290475475837
0.8877805437762163 0.1091587004897860 0.9894808644607025
0.7212612139756871 0.7758775196139993 0.9892872371222765
0.5563288202976509 0.6113652095061823 0.9975766101492667
0.7218041472402064 0.9427563188859338 0.9908157927269059
0.8886258173114214 0.2761600001416511 0.9908470726957646
0.7213453975572577 0.1089348164761432 0.9938920218319467
0.5551344176662170 0.7760077301928234 0.9955622243306692
0.3858746465199161 0.4417775614995492 0.0118477149299920
0.3887000339228663 0.6094747662947029 0.0078902251140605
0.5547724343508662 0.9427835616700619 0.9982022298194337
0.7213824687445722 0.2761571802533780 0.9986143561807452
0.2215345973107219 0.4425362865262557 0.9895544910254515
0.3894281159613939 0.7775163602964170 0.0068401683699975
0.5554029725711942 0.1094088082519420 0.0014056805378004
0.5547372009542428 0.2753177904826956 0.0068098053024599
0.2202866537886905 0.6082161554434222 0.0035106748212854
0.3883525442324256 0.9424029627106094 0.0042224569022338
0.3882033427337532 0.1090220682995735 0.0053451820168746
0.0547679690315945 0.4428029695747995 0.9912017263640465
0.2213507514261650 0.7758822289555312 0.0051710441429051
0.2219919937008612 0.9426179470218869 0.0026935953285019
0.3887977574913961 0.2747412048960811 0.0105668767920619
0.0549647938075282 0.6091817289104640 0.9991258515108044
0.0547412669467349 0.7754292458478389 0.9990895428984672
0.2211415070748401 0.1084529541457826 0.0021551268594138
0.8881977407009245 0.4422001327682565 0.9959731709456605
0.8880858712633000 0.6091908519148390 0.9969775986248877
0.0545404486686323 0.9424852736270992 0.9960954026043112
0.7223205257361229 0.4428846506784723 0.0022005343063256
0.8885187514901602 0.7759730143282439 0.9925632456080962
0.7218226434100714 0.6088192640762600 0.9962815828778773

0.5561035485986849 0.4427251002601054 0.0156781802604158
0.1646913536545824 0.1638972946467376 0.9982697050753160
0.9990796869799894 0.9975083696420910 0.9926006307606841
0.8327390841161346 0.8316102252060986 0.9899077804494070
0.9991281095006386 0.1647688645038485 0.9897270509838236
0.8320123034102380 0.9977712691777114 0.9895512160617059
0.5008034768452703 0.5004778326514199 0.0151958788420998
0.6659232403501534 0.8313117897539328 0.9908008627860525
0.8325635010722010 0.1645294364879248 0.9908332445341159
0.6659885142937085 0.9984495617935352 0.9938930068688876
0.5011028911666600 0.6663889351832495 0.9985562605970131
0.3307776220756793 0.3296181363948349 0.0127793725945864
0.3302437729673926 0.4969825478909513 0.9977665525784545
0.6654845452962803 0.1646327677204411 0.9979588164173947
0.4989374793805510 0.8318377318841144 0.0001295101837471
0.4993542650837242 0.9980139128357237 0.0015438673008816
0.3325215236203221 0.6652576152244620 0.0112549074616553
0.1654886465694290 0.3306677505763318 0.9910403053012118
0.3328885916480928 0.8320523536382467 0.0061234144289841
0.1656514811410048 0.4985209800113068 0.9930213102470304
0.4992918385339848 0.1647841732500481 0.0049290414832797
0.1651916793439199 0.6643586824443588 0.0033464970947605
0.3320795599917616 0.9978181021241781 0.0041780778106784
0.9987128972675450 0.3313107775113409 0.9885858848970273
0.3326658550394441 0.1642188187989007 0.0061857303153658
0.9993416780503850 0.4980625909650129 0.9952837013990106
0.1660023146680061 0.8310303862295356 0.0024406388832858
0.1660112787002603 0.9984176914333887 0.0003553397684684
0.8327345256680871 0.3319008579755547 0.9948233860539304
0.9993102005544827 0.6650669710769463 0.9983883807823943
0.9988281838757445 0.8311705739869097 0.9957219456511908
0.8325001885864722 0.4982005446130842 0.9983318965437107
0.8324775529573785 0.6643423594563984 0.9944028157306732
0.6662450412278957 0.3313266796643433 0.0032428540858049
0.6672439546033004 0.4989496779800433 0.0045978777514932
0.4986627666695682 0.3301702504030288 0.0116301014376887
0.6658866912510035 0.6651123463214930 0.9925054002211411
0.6810318206953028 0.6608862993109810 0.1946625440970493
0.5344177629960924 0.5059651284537665 0.1289524077165680
0.3122731673315613 0.4909852329200390 0.2393193550354340
0.3427207124427980 0.5995100643586895 0.1226900112512652
0.3321045883745626 0.4001084908304620 0.1239948184836734
0.4990122693923098 0.6233633253772695 0.2085468476071455

PT6-XXV - ΔE = 0.73 eV

1.00000000000000
14.7772044699999991 0.0000000000000000 0.0000000000000000
-7.3886022400000000 12.7974344700000007 0.0000000000000000
0.0000000000000000 0.0000000000000000 18.0009597799999987
C Pt
72 6
Direct
0.2270570866204267 0.2778225011168445 0.0030817892169708
0.0601669445222299 0.1120907530560231 0.9953710909341282
0.8937592695275356 0.9460350564999160 0.9936830201822886
0.0601827022863688 0.2787774703342194 0.9990052071553777
0.8939811010696417 0.1124633061349058 0.9941162298305528
0.7273954202397590 0.7795698003941567 0.9973354693771057
0.5627420435637589 0.6135619804332677 0.0078837941888139
0.7273644284969762 0.9462768980379153 0.9956991726913600
0.8942215677648093 0.2792960282002710 0.9939362439432244
0.7274522706864275 0.1128221428828979 0.9930786899054382
0.5616391531217886 0.7812691617272023 0.0056084846182287
0.3935194157955095 0.4434121166843710 0.0207551614222012
0.3934737098877434 0.6142787998475825 0.0218152935919207
0.5606400757339429 0.9457601694074924 0.9955970019562912
0.7274846489319131 0.2790292330784823 0.9926962251904854
0.2253129039710586 0.4448929511061834 0.0104969714578260
0.3934449350524645 0.7796364649502081 0.0012593268509349
0.5605007135261388 0.1123989013586808 0.9920412335995934
0.5606332492368367 0.2792346678094120 0.9944737444172063
0.2269869097175459 0.6134945928611870 0.0037917936636020
0.3940875623876465 0.9461785213565008 0.9929837009977902
0.3941035493773413 0.1122196248132710 0.9922843055979271
0.0602978296195786 0.4458696804319899 0.9987231207125689
0.2271422991634395 0.7792091432108492 0.9952628980089884
0.2270237626803606 0.9456683650881246 0.9919311908338599
0.3934443575246434 0.2781218307179287 0.9996399188144238
0.0601918536902417 0.6124359117893903 0.9955391815528358
0.0603761251080854 0.7791780487716693 0.9926421268779393
0.2271455322124112 0.1122941822363401 0.9943448326561466
0.8939826833800453 0.4459324419337065 0.9938258180823425
0.8937930139107451 0.6121546073612691 0.9935201529119908
0.0603856035101984 0.9455840824344435 0.9924388955631400
0.7273817062865078 0.4454449709548811 0.9949777016251957
0.8934749280246876 0.7789265289512741 0.9930989655496063
0.7273730568910892 0.6122044062386252 0.9969314239731659
0.5616276974866850 0.4447229261828909 0.0044298553114004
0.1717736415506721 0.1676774801138876 0.9970129716569147
0.0048824033531005 0.0013763050367217 0.9933880287147758
0.8379057574741822 0.8344852691627054 0.9941709125899649
0.0047875913201167 0.1675128408676940 0.9960023591757405
0.8386673477316862 0.0015288440402799 0.9940396854157427
0.5070056256230586 0.5013953801114823 0.0151886839541433
0.6721940760959697 0.8355154093271508 0.9994803951394005
0.8385141121827999 0.1681935069896525 0.9935768542511525
0.6718534374389051 0.0014604848599120 0.9947454672784204
0.5070140513922112 0.6699126960729060 0.0160216735311067
0.3378122990321515 0.3325876996290731 0.0071927957951843
0.3361231902044040 0.5001115713608328 0.0217764148511606
0.6719716879807507 0.1681580438425243 0.9923546831968579
0.5054651957563721 0.8357807856710195 0.0007770464355161
0.5047938362409994 0.0015585199959673 0.9930821637754121
0.3378055824874409 0.6694962171242480 0.0086535412492665
0.1704183410660107 0.3329782731304292 0.0054607564101801
0.3381935350845069 0.8345934130644892 0.9957769241757006

0.1703657123005442 0.5018643670239200 0.0050819390330261

0.5047715421995989 0.1675512254087863 0.9924003738384002

0.1717391745228269 0.6684452118191615 0.9977036881006711

0.3383613295616641 0.0013153716060756 0.9918151172485992

0.0051315419406279 0.3347226490243855 0.9966538036874297

0.3382021226552681 0.1679199696859683 0.9945597743850740

0.0048401444094850 0.5016962419019038 0.9958412795704490

0.1714388683031132 0.8347535450526706 0.9928025247555183

0.1714215881272452 0.0010348522527295 0.9923727645326679

0.8385011505445590 0.3347288150675922 0.9933097818331689

0.0048560529090480 0.6679198032213591 0.9935000989920653

0.0047015912938875 0.8345477872594103 0.9921905516767566

0.8386941086756039 0.5015270416157307 0.9936777835311403

0.8379274133483818 0.6678521940072883 0.9939869970218069

0.6718864668061499 0.3347999031152682 0.9939378985871627

0.6721804149247177 0.5010507998857676 0.9986612522940206

0.5054609726227000 0.3340218614569039 0.9992841930092737

0.6716805794921470 0.6680194152098480 0.9993457144810662

0.4651183768297997 0.4779887777821301 0.1319942530284806

0.1813074105804162 0.4170643507119465 0.2567417594103887

0.4649484117027072 0.6498253327732684 0.1329046360318671

0.5652058304909460 0.6133345450707068 0.2373043671760655

0.2515389153838612 0.4540556908541475 0.1276868067218828

0.3714394920073119 0.5166633705033448 0.2492688249520540

PT6-XXVI - ΔE = 0.73 eV

1.00000000000000

14.7772044699999991 0.0000000000000000 0.0000000000000000

-7.3886022400000000 12.7974344700000007 0.0000000000000000

0.0000000000000000 0.0000000000000000 18.0009597799999987

C Pt

72 6

Direct

0.8916963013305795 0.6150344975432661 0.9932816932002505

0.0586307336884160 0.6157770507831497 0.9932396525407754

0.2257779910547980 0.6156227040555099 0.9963135059016426

0.8925540531029793 0.4489237775524094 0.9928060618390502

0.0583359996619423 0.4484255296017981 0.9952186977064021

0.3914634943183450 0.6153547665856850 0.0013697653997027

0.5595687105464527 0.6173034049306502 0.0036078987813709

0.2251202880703875 0.4494461477267747 0.0034971259366717

0.8920398483433031 0.2825148987342189 0.9928581865391373

0.0590299664151253 0.2822226517965423 0.9968690858640353

0.3920439766992222 0.4504795904452020 0.0209415827874239

0.7260626270741884 0.6157992698275179 0.9973992696875386

0.5597051992976219 0.4481158746986816 0.0050068184203070

0.2232224481845464 0.2807569591032528 0.0057393055512460

0.8917777391459865 0.1152565466630949 0.9924313768857536

0.7255341871717650 0.4490432292057548 0.9953815600668515

0.3925264890747772 0.2812952037008015 0.0130367175304256

0.0584992189656486 0.1158114414791167 0.9944980375323235

0.8924680676814911 0.9490858372552440 0.9920933945325645

0.5595749747834791 0.2825492329811687 0.9981164977546299

0.2253643185305597 0.1150002424086162 -0.0006727506193616

0.0581164783906409 0.9483729807324566 0.9929702841419785

0.7252236108119100 0.2818071134469090 0.9931329911542763

0.3916750769695994 0.1145693451270180 0.9980284722807327

0.2252385120769979 0.9490107098758805 0.9941683059704817

0.8918356794557172 0.7825895700938994 0.9929992936413871

0.5585253749098870 0.1159972039023775 0.9941950147094982

0.3925301156740892 0.9487843888738274 0.9936550870413081

0.0590686913605996 0.7822406160169220 0.9926141738504460

0.7257962709682264 0.1157102901124318 0.9921922110867261

0.5583455287677097 0.9484848925098187 0.9930650084174550

0.2250837968411278 0.7817198308474509 0.9932900891023522

0.7253003008083906 0.9493118800791760 0.9925243569762030

0.3920565160754257 0.7825590020415553 0.9944534828348689

0.5591356205674808 0.7822591486583376 0.9955829655554619

0.7251109210675615 0.7821200608658093 0.9945330440614686

0.0034568650728031 0.6710028511495907 0.9926818140964881

0.1700131111418775 0.6715065509459698 0.9939337233268974

0.3361650986360055 0.6707936705304318 0.9968084735597303

0.0029690818528146 0.5042556553022659 0.9934136430128389

0.1702491257457764 0.5044585640028268 0.9980836611894526

0.6702139119998756 0.6720474916392167 0.9980538936399942

0.3359175553724009 0.5057956478146628 0.0080281272053248

0.0032562285084135 0.3383393560923934 0.9948112099002183

0.1689163632043179 0.3371793980205835 0.0024893995518456

0.5047481702865243 0.5055454041686869 0.0155122063248858

0.8358074984101849 0.6706616555589701 0.9943217655906851

0.6703886950571010 0.5042037327604595 0.9987581164880069

0.0033748972860812 0.1711112905310958 0.9943297360087332

0.3344812757181335 0.3355043653495025 0.0190175514795575

0.1690836813280624 0.1711514925590431 -0.0009338559519497

0.5050383573774030 0.3387435400038403 0.0042367362207463

0.8368055282222413 0.5049664656044226 0.9933931458613406

0.3357180431018664 0.1694375543679545 0.0029524698948412

0.6698713575503253 0.3377026605584187 0.9952004233849463

0.0026964299814546 0.0040319415103534 0.9926457033783560

0.5036508519542975 0.1709198776332884 0.9969312198255311

0.1700994795617295 0.0042927402913150 0.9949361170662113

0.8370116815275670 0.3378845205600046 0.9926232093754365

0.0031360826953891 0.8381509915776979 0.9924801243475816

0.6700656956652813 0.1717207737289519 0.9929677199586017

0.3364813300251470 0.0045715263267460 0.9949600311521373

0.1693847018108086 0.8373987840013634 0.9929356602950925

0.8361909802354092 0.1709925867515420 0.9923173302923912

0.5026582909131576 0.0040722414595517 0.9935058151864558

0.3367937155965455 0.8375461946527447 0.9934885471133861

0.6701819011558031 0.0044732148960045 0.9924583838793601

0.5031799549052582 0.8382872791196736 0.9941474675415918

0.8366325882505667 0.0049877253549503 0.9920474548313006

0.6695579494724162 0.8376522389520322 0.9941559334206186

0.8369641341555656 0.8378419461129674 0.9928324302791389

0.5038232784130229 0.6715648559915033 0.0000421847407976

0.5733966724241293 0.6486900298961944 0.2060146699195403

0.4572101005782113 0.4861302869324904 0.1325189197460518
0.3546243432615131 0.2880910001287099 0.1298977750970725
0.3465832924969587 0.2830582362984494 0.2671846725160845
0.4368262643924791 0.4735613415580071 0.2703945176583408
0.4474058927462329 0.6436001102211092 0.2985327908811689

PT6-XXVII - ΔE = 0.74 eV
1.00000000000000
14.7772044699999991 0.0000000000000000 0.0000000000000000
-7.3886022400000000 12.7974344700000007 0.0000000000000000
0.0000000000000000 0.0000000000000000 18.0009597799999987
C Pt
72 6
Direct
0.2165294116094003 0.2758947542724002 0.0161772204121533
0.0510996081912651 0.1096982620980356 0.0063031772475384
0.8851809179482117 0.9440256317043634 0.0037806752569267
0.0520282495292363 0.2775169272032016 0.0027454409954473
0.8854939613474286 0.1105574960362929 0.0004900219683464
0.7194842181293168 0.7788069577077366 0.0080558767039847
0.5523493721645636 0.6125812934475192 0.0158413317060351
0.7184247617181541 0.9435407429983087 0.9982588743138194
0.8852632688308049 0.2769007293631347 0.9951358033048692
0.7186883524705010 0.1103619133290294 0.9932170377990772
0.5512980219691741 0.7772097495330215 0.9975809612465198
0.3844263312781280 0.4443973142579978 0.0144977981342791
0.3849566876938084 0.6107408418366873 0.9974817217486276
0.5520262565765037 0.9439149091695711 0.9909748262884364
0.7186082155860660 0.2770715089592386 0.9917101105722281
0.2184151638801453 0.4440099168705487 0.0006968108899628
0.3850027473256858 0.7770088764614158 0.9903564237485796
0.5519573534167250 0.1104341472117270 0.9898590612160660
0.5518705659115639 0.2770173561873008 0.9931997644089918
0.2182850233192042 0.6103772549970827 0.9920214381726566
0.3851601769423567 0.9438268331979458 0.9895676567248941
0.3851722796885184 0.1103642855610829 0.9930267565155830
0.0517921165824973 0.4437413791056102 0.9948983649608678
0.2184410293626797 0.7770130980946135 0.9907561141752765
0.2183316828320727 0.9434619690766226 0.9944957082694614
0.3853329332435180 0.2757374136152677 0.0030401570806333
0.0516493705562482 0.6101113480770195 0.9926390171120900
0.0516458168732896 0.7769336795620205 0.9961019137262070
0.2179708849862578 0.1096783124662792 0.0029687113021239
0.8850798602758871 0.4435325676202879 0.9933225982065750
0.8852943392950436 0.6101588476164039 0.9968814214764095
0.0516449830844365 0.9436052990996444 0.0017672064868535
0.7186285684073823 0.4434191943380981 0.9955570397381095
0.8852325471095952 0.7771820662321787 0.0029619943870003
0.7197189874039935 0.6100084186464088 0.0057254783165845
0.5529212247573483 0.4430045784336158 0.0038869590850226
0.1617533160181708 0.1639725642364240 0.0092311749811458
0.9960654100190354 0.9989470511996075 0.0041251277466827
0.8300729661707749 0.8330765524538961 0.0052689015064331
0.9958701627750983 0.1655878330977529 0.0040926567116060
0.8297104344609636 0.9992847371620428 0.0013347937975468
0.4971186526127553 0.4987315996722614 0.0154341237373998
0.6632294092582356 0.8334425727101760 0.0012407488655910
0.8295460026121333 0.1659708888872160 0.9962355924882615
0.6626603989346833 0.9992522264268544 0.9937061423864435
0.4957277043556516 0.6671471650235858 0.0023966168643526
0.3289097688555245 0.3304757168101844 0.0159731775128549
0.3284692313965820 0.4999110853812923 0.0027490829894390
0.6627601622869221 0.1658321897131287 0.9909891217523068
0.4959873436674727 0.8325177182341079 0.9919093363431628
0.4960419782255201 0.9992579760019851 0.9894180571560298
0.3293917190729800 0.6660827646618088 0.9921893002370297
0.1616310383294746 0.3328601713713155 0.0067353431499555
0.3291431968889853 0.8324060977266114 0.9895658138684098
0.1625859676571864 0.4993715472571409 0.9951268408813831
0.4959987905881320 0.1658518188199665 0.9912220537579444
0.1626910586621904 0.6658706956317175 0.9912288342672682
0.3292273757110920 0.9990368499590616 0.9917903560749934
0.9960445532859765 0.3326929736527831 0.9973050554128093
0.3298461476460446 0.1659082713144926 0.9988761664927353
0.9962095941323028 0.4993953669496349 0.9931889026295764
0.1624878586588281 0.8325786960130515 0.9933684727080276
0.1626324744366201 0.9990340122886820 0.9994630398572824
0.8294574032808768 0.3325872219257278 0.9929263630635106
0.9958723001073650 0.6657568903687121 0.9948587041480437
0.9957470843582215 0.8321981772816329 0.0001817659353094
0.8295878718466057 0.4990945347524871 0.9949232230083567
0.8292601100577883 0.6654389133613634 0.0016649750814608
0.6629659273510811 0.3325717977771347 0.9927816796639206
0.6629753857603120 0.4987596286492391 0.0009642813329762
0.4964827657229236 0.3323897955159651 0.9986939256480412
0.6646990002877828 0.6669110645314618 0.0145377469350692
0.3364344895943958 0.4078686847015547 0.2524070493673563
0.4411377516097303 0.4743374041132213 0.1264048034610852
0.6981044843530739 0.7006940251298843 0.2584651504749971
0.6178612868244997 0.6559296523780844 0.1303155052557301
0.5251943912892969 0.5447626055801322 0.2523930544727762
0.2735571539896426 0.2993241450904449 0.1328846463257065

PT6-XXVIII - ΔE = 0.74 eV
1.00000000000000
14.7772044699999991 0.0000000000000000 0.0000000000000000
-7.3886022400000000 12.7974344700000007 0.0000000000000000
0.0000000000000000 0.0000000000000000 18.0009597799999987
C Pt
72 6
Direct
0.2189935841655597 0.2755742252923739 0.0040557918953112
0.0538146715460499 0.1099802177455445 0.9975339827063578
0.8870181813252174 0.9431749091224759 0.9957636399835081
0.0540302524238641 0.2763254457355941 0.9936289810563181
0.8871736023629211 0.1101369303967275 0.9955239002365914

0.7206019854695995 0.7772204166567747 0.9968842702703142
0.5566026751807058 0.6119912517838060 0.0041791813166228
0.7210852958795968 0.9439954903755918 0.9992067532436053
0.8878989025246113 0.2771048152138320 0.9926149386486713
0.7208148198026834 0.1099815530991179 0.9970786777924019
0.5546774205009228 0.7781884879170775 0.0072053520949282
0.3855570616556108 0.4426539160633638 0.0146125652869671
0.3857467585429148 0.6101241720828909 0.0095875600842561
0.5540227761950121 0.9436085340786562 0.0006021565798520
0.7206382871781258 0.2767979500474453 0.9957126318116494
0.2206951343552745 0.4437335745690518 0.9942348746314238
0.3874000608589085 0.7776749383957622 0.0020303002703557
0.5544597918718779 0.1101754123109515 0.9989244851874091
0.5540469788634397 0.2759151606811585 0.0013900560106919
0.2203820944335249 0.6102001234164476 0.9963868653140011
0.3874350698391353 0.9433895822159641 0.9992848853809022
0.3873226268767311 0.1097092687254886 0.0013860907239547
0.0541337920802221 0.4436071855314125 0.9907282407722349
0.2203558256866387 0.7768313261415827 0.9965478278100761
0.2208491559097610 0.9435556159253338 0.9980146170664668
0.3880444256463633 0.2755499246938697 0.0084913373030915
0.0542587530999796 0.6103673972611929 0.9919759659867395
0.0538288491810448 0.7764469599322652 0.9935699648867313
0.2203555575698815 0.1094362569932485 0.0016225355301884
0.8876424283723949 0.4431497963472779 0.9915408630735172
0.8873165864669588 0.6099647135958719 0.9914938571114291
0.0535401026455204 0.9433267958130287 0.9962045608387271
0.7216985843357975 0.4436115088424870 0.9956353817822503
0.8874346259630030 0.7769778945550314 0.9924682310862920
0.7211606668977311 0.6096206248552676 0.9940790538713387
0.5558951724190422 0.4426587242017561 0.0112138410817764
0.1640869122545610 0.1647475562000693 0.0011481394052666
0.9980289236197848 0.9983299471406468 0.9963309424931523
0.8314271946952303 0.8323035020698839 0.9944176336914126
0.9983589726499105 0.1654190424815241 0.9955083411545829
0.8314430621649294 0.9988845573146534 0.9968332252872045
0.5010081098374215 0.5000840814915648 0.0152525172272888
0.6652659246224033 0.8325836486572413 0.0015358144233559
0.8318103781667432 0.1653562949181904 0.9949758885867936
0.6652963179879734 0.9992737027397638 0.9994061236814744
0.4995741417977584 0.6667701698821489 0.0132711257432732
0.3306412955821543 0.3304820891900633 0.0135263982450411
0.3295029243828367 0.4984776089844800 0.0014962373450800
0.6647789576171874 0.1654433722160960 0.9972570413980151
0.4982927632821017 0.8329201844907672 0.0035250154973809
0.4982203980728031 0.9986692754324764 0.9995696998211088
0.3308323613270332 0.6662172485966025 0.0033203697676640
0.1644930934792370 0.3313040346751563 0.9965387326106807
0.3317217517907309 0.8323906964800614 0.9989857394215917
0.1652094585864887 0.4994243554852744 0.9924657758693201
0.4983540255254084 0.1651422917155188 0.0005647533993312
0.1645831967078024 0.6657449248953853 0.9945861928729869
0.3312079030642323 0.9985277698501562 0.9994065974254838
0.9982301019419211 0.3322321151395116 0.9913161336139140
0.3317479308675289 0.1648262223512660 0.0038309027253263
0.9986564969980094 0.4988755215350196 0.9909309017049210
0.1648303483946520 0.8319497858724034 0.9958280791435001
0.1649747055458732 0.9989274511526531 0.9985156526206822
0.8320968165978826 0.3325025735623584 0.9929347256139707
0.9985237136246639 0.6658054872556463 0.9919158312193446
0.9976208867766232 0.8319824569637930 0.9937566170424859
0.8319560062157052 0.4989580045909108 0.9921910646702869
0.8315588709339039 0.6650892860139734 0.9916410247343563
0.6653336227673918 0.3318360475002251 0.9976812370693935
0.6669909423261799 0.4993158860175768 0.9991218582423258
0.4979788562280021 0.3303800602447353 0.0068812830897755
0.6649401344832384 0.6657642390754503 0.9964066795712156
0.5373546134624192 0.5968966592780660 0.2367029525410658
0.4334108409590698 0.6189546270771018 0.1270508329472619
0.3388017857314978 0.5045991191003907 0.2434058448995842
0.7074134065532931 0.6156177578637312 0.1973888940533968
0.5434668272575891 0.4950221120846976 0.1278480626989094
0.3413496074004811 0.4060972729504684 0.1271561996202593

PT6-XXIX - ΔE = 0.74 eV

1.00000000000000
14.7772044699999991 0.0000000000000000 0.0000000000000000
-7.3886022400000000 12.7974344700000007 0.0000000000000000
0.0000000000000000 0.0000000000000000 18.0009597799999987
C Pt
72 6
Direct
0.2195137966104781 0.2739341875155503 0.9929261266658145
0.0534588285224302 0.1073969650215432 0.9921213899400669
0.8864365346806906 0.9405230138720739 0.9945470981536886
0.0527249716513012 0.2735356240291367 0.9935329829458226
0.8863199465887064 0.1074780938803741 0.9908289050293035
0.7205776871958989 0.7754646862423371 0.0021921502908953
0.5535158032686311 0.6089254244828126 0.0159041432233025
0.7202334343365777 0.9404517139584598 0.9896846275195514
0.8867213065660096 0.2740103761922086 0.9931785912402447
0.7194667548387699 0.1066900954875223 0.9895214428975976
0.5528969286080354 0.7732906974345184 0.9918226947298407
0.3844480754979074 0.4388026535298408 0.0021983283849778
0.3866839374579598 0.6073292544075102 0.0105490565591353
0.5528032830977665 0.9405085274018106 0.9894296558551900
0.7196112683435416 0.2741976281057035 0.9942328656392760
0.2194281380637406 0.4400431482705756 0.0001672721259780
0.3874417968201698 0.7753284558142894 0.0010276772881710
0.5535639673055499 0.1071757384894454 0.9918850046980552
0.5530365774299852 0.2729612165205424 0.9972285290740572
0.2184273069045304 0.6064418033500871 0.0073529635933269
0.3865070272569753 0.9403949893339885 0.9950867191113488
0.3863563282626998 0.1072124986160290 0.9939396635309805
0.0529333131596275 0.4407279500892969 0.9988148204838012
0.2197121865588940 0.7741566754326286 0.0025482753997608

0.2202096177835458 0.9406736968538567 0.9968383431473242
0.3865055674181122 0.2733894393873726 0.9962419682768626
0.0532552753097306 0.6071726552954502 0.0022816038526159
0.0530492964165816 0.7735608354462329 0.0010240803550445
0.2195247004450849 0.1068025897086287 0.9929977158163936
0.8863807697119697 0.4401714138599644 0.9983271519681978
0.8866917598538677 0.6073906898783150 0.0023308588535613
0.0530667345010514 0.9408666547010114 0.9963621305264425
0.7206838545921386 0.4410126006447896 0.0009890991701837
0.8872064498857455 0.7743879736609429 0.0023302324500278
0.7210940277720397 0.6070704160868559 0.0089142741549466
0.5538019481099852 0.4396056382861886 0.0102956563426915
0.1638591636561770 0.1625109142723034 0.9920549204584503
0.9979695649778648 0.9962071212298511 0.9940412422817104
0.8314315430847898 0.8305201599618073 0.9997080033696761
0.9977120207022807 0.1632611152678933 0.9917870700282378
0.8307198652439922 0.9960166886519488 0.9913567015079394
0.4957264870686373 0.4936219023077584 0.0151074984192405
0.6648821419359692 0.8295556190619777 0.9929920048037886
0.8310858785459700 0.1627130300241717 0.9906983153261422
0.6642700244336623 0.9964542619499284 0.9881984694039048
0.4987264180887934 0.6648397828272579 0.0003381801515729
0.3302086855019937 0.3292006962706395 0.9953683543714149
0.3294255003825484 0.4943291954593150 0.0039578570388414
0.6637876803723799 0.1625907859570148 0.9914020488393476
0.4969148001183825 0.8292882419550267 0.9920875029800698
0.4976615716339126 0.9959135387312656 0.9916896243472024
0.3309112479276024 0.6638604332152340 0.0121726560913586
0.1641584715134243 0.3294612224250812 0.9950235195697132
0.3314428186765070 0.8302866607386861 0.9997068120997241
0.1637579539595038 0.4965475146649183 0.0022998709506936
0.4976592132371351 0.1628910330181910 0.9939997852178521
0.1637028750511007 0.6626863830985599 0.0046923526768126
0.3305946528435939 0.9960164184100648 0.9950086437109604
0.9971892883858615 0.3293544885209272 0.9949544977929392
0.3310185842986755 0.1625363049653359 0.9937378331888169
0.9976964834285056 0.4961742815940084 0.0000533822664366
0.1645856107169550 0.8293153023089559 0.0001774520734870
0.1643748260965481 0.9963772098680010 0.9951128697108018
0.8311146929251478 0.3299709922751717 0.9950203750092541
0.9978436937447626 0.6633716130249994 0.0017797748258772
0.9972457594368649 0.8293929808969693 0.9999305614924410
0.8310960863803203 0.4962929881361049 0.0006421886401426
0.8315549838924738 0.6629292542537399 0.0046824942159631
0.6646213304686057 0.3293943040042535 0.9974554360388908
0.6662536783672124 0.4973042507989760 0.0063224534345068
0.4969179195488920 0.3279526862159088 0.0011003172360802
0.6658869328399675 0.6644628574350406 0.0147963745395288
0.3789297717607738 0.6319037310270232 0.1264344039972585
0.4668490655888178 0.5994609447146786 0.2397158708275740
0.5448044199967583 0.7835692673992014 0.1823729690967326
0.5074396382760042 0.4433353316773605 0.1261135233255075
0.5860301171476294 0.6425963360587090 0.1306407786765504
0.4417499049330473 0.4221999159909657 0.2546210255149930

PT6-XXX - ΔE = 0.75 eV
1.00000000000000
14.7772044699999991 0.0000000000000000 0.0000000000000000
-7.3886022400000000 12.7974344700000007 0.0000000000000000
0.0000000000000000 0.0000000000000000 18.0009597799999987
C Pt
72 6
Direct
0.2247984279351485 0.2805351352208874 0.9990242908200528
0.0583403887596958 0.1136770347590641 0.9961729882458386
0.8917366079955826 0.9475151346822557 0.9963472803200304
0.0582316517638475 0.2804934784072444 0.9920427433361709
0.8919735320219928 0.1139539726546772 0.9927604762553770
0.7256985702399135 0.7810938024586491 0.0019581079811033
0.5587650607169934 0.6159830783155442 0.0160200510790531
0.7248387906715692 0.9469280971717495 0.9949233438877627
0.8916363235277771 0.2802160453792908 0.9914817766778299
0.7248592264338711 0.1140102988127438 0.9919965205916341
0.5582785977122597 0.7809587705287129 0.9981222712826892
0.3896984330068634 0.4462877427063958 0.0050737231775173
0.3920972289020526 0.6142474369492774 0.9963616174741361
0.5586410713712482 0.9473609104933871 0.9923840337880279
0.7254742914176120 0.2806297756442504 0.9954904397895774
0.2250998604130885 0.4467705660698016 0.9920971107615912
0.3917121259764400 0.7803002529778809 0.9923713764923505
0.5580876019624057 0.1135021480152005 0.9937719646150214
0.5585191979690407 0.2793450493330099 0.0048926877327915
0.2250242900440185 0.6137152302348312 0.9907257434520957
0.3916331598004561 0.9470052948541081 0.9934857981596608
0.3915560861100076 0.1128305633796884 0.0014237239060932
0.0588879914731208 0.4471621559487673 0.9903585832505755
0.2252712035255371 0.7804867914971894 0.9926792619459519
0.2246374070337041 0.9466523734504833 0.9973792686908851
0.3894308769971602 0.2790396229630687 0.0155189406139939
0.0583149729847534 0.6134764967146040 0.9927563937823578
0.0582367009777798 0.7806652573974517 0.9956689750072698
0.2239611679286284 0.1130119219613164 0.0030258750640400
0.8920134555659018 0.4471713835261539 0.9946407684136176
0.8923668826569298 0.6142141595915334 0.9990823864729421
0.0583944587977072 0.9472002393191161 0.9974509983213764
0.7265098737592908 0.4467348600591592 0.0061066304841333
0.8918704478505859 0.7808357980027552 0.9989575833829392
0.7266799848843561 0.6161785995505582 0.0090054486019753
0.5601752007251122 0.4471054837452542 0.0246167356209170
0.1690364911088977 0.1687102039978541 0.9996201966875589
0.0027827768931701 0.0027324011864707 0.9966776655586997
0.8362010053839342 0.8361679033774863 0.9990042765270886
0.0024978523928141 0.1691696115223635 0.9934360146184957
0.8361978322526937 0.0025559655305116 0.9946172884104740
0.5006712931895834 0.5029847449555902 0.0224543737541296
0.6694339641659326 0.8364495122366193 0.9986706185983891

0.8361211512090350 0.1695133160111837 0.9917960119974509
0.6689359930046663 0.0024008921401233 0.9926872981134096
0.5020886803204476 0.6700081039731600 0.0029605098868402
0.3343070471995730 0.3360693731323323 0.0056788097667777
0.3358350261066008 0.5020266806619631 0.9965656801087803
0.6694209810669491 0.1690547541106397 0.9931808986245514
0.5025758719046038 0.8358305204393091 0.9936782393866963
0.5024677063813385 0.0025251804639410 0.9925544189663285
0.3363850519507068 0.6692782212513890 0.9921854979653375
0.1693627357710383 0.3360322986216886 0.9936539392448580
0.3357039710771375 0.8356348538017997 0.9922930072578708
0.1693044622790865 0.5024108601043267 0.9902363758915840
0.5023808420819407 0.1684045530798315 0.9986905302396849
0.1695153440888291 0.6690143417372383 0.9915403149388737
0.3360148290121430 0.0022912839317684 0.9969880004158256
0.0029135948662073 0.3358019532168015 0.9907994703972065
0.3347711742051374 0.1675113398299004 0.0087729281128759
0.0030500539637686 0.5028063660884214 0.9920420605481084
0.1693720395000042 0.8360017839003078 0.9948814694572050
0.1687797694977533 0.0019507732219068 0.9992312878747214
0.8359670500745722 0.3358041519880501 0.9935697302233422
0.0022627171282963 0.6689511806352328 0.9954604357923671
0.0029229715590944 0.8358330013044721 0.9969676927459901
0.8367208034470508 0.5023917454697369 0.9996584514548204
0.8366134811685981 0.6701620072234675 0.0017245819053571
0.6697845981799304 0.3358302341387684 0.0020234896046674
0.6722339959315775 0.5040300727392903 0.0172145207164860
0.5024228960606791 0.3332348374586900 0.0178851098660786
0.6709147440429390 0.6708135490436575 0.0092846465963632
0.5047437765393568 0.5465137874459316 0.1338719084767632
0.4350780022857919 0.2808286041570298 0.1309239646776774
0.3486579322486421 0.5349212434908068 0.1998888134012731
0.4010575349565642 0.4019224701956787 0.2228309584468064
0.5392982445303858 0.3532331311459131 0.2512382099191015
0.6224863091323130 0.4614217057577790 0.1335401957322588

PT6-XXXI - ΔE = 0.75 eV
1.00000000000000
14.7772044699999991 0.0000000000000000 0.0000000000000000
-7.3886022400000000 12.7974344700000007 0.0000000000000000
0.0000000000000000 0.0000000000000000 18.0009597799999987
C Pt
72 6
Direct
0.2164582091223437 0.2760953835822448 0.0029174148370998
0.0503613788952393 0.1096347585040363 0.0047107088883820
0.8836644465759207 0.9427139990298983 0.0060079582285937
0.0505339295230769 0.2762979673016019 0.0010122633960492
0.8839292924263731 0.1095567871425160 0.0039769897342410
0.7174630889623472 0.7769388784349971 0.0059957976677936
0.5500040157790735 0.6094776792790242 0.9911717701917979
0.7173195721613936 0.9429208274288499 0.0040317949387187
0.8842362523184022 0.2764437184420316 0.0000216162646325
0.7174160688574673 0.1094389209885875 0.0018462626349134
0.5503669780994116 0.7759723063904929 0.9993193388285704
0.3852014729351083 0.4444659296942319 0.9956986205181551
0.3837788049721880 0.6103921119195164 0.9933340848849426
0.5508698363967852 0.9429008219057664 0.0015158682159822
0.7180802271219804 0.2757738242223340 0.9998187820758062
0.2169660257218666 0.4433030592130365 0.9971411429187498
0.3840092776222832 0.7766154063160684 0.9981845010366399
0.5507756426228099 0.1092823974277408 0.0012719964293950
0.5495266954083036 0.2759927750744637 0.9977664223627656
0.2170754529364629 0.6098423585706811 0.9967139442479933
0.3839030520583790 0.9429665472818840 0.0013087986049172
0.3840125085773991 0.1094741705604321 0.0011600265009761
0.0504542075737788 0.4429701615471870 0.9980788419802451
0.2174304509076492 0.7766053822427992 0.9998538121101106
0.2174824141804521 0.9432524771959123 0.0029075210127658
0.3843108638358714 0.2759486811158993 0.9957594535697112
0.0508610800518241 0.6099765447056527 0.9978678351322969
0.0508273745720373 0.7766100911546658 0.0019191304113946
0.2173800865402526 0.1098880043049988 0.0034215355410794
0.8840417040692898 0.4432297228019024 0.9963059375508365
0.8847542073326338 0.6102993770927441 0.9971375380850489
0.0507765388304051 0.9433657741911858 0.0052149125667853
0.7185190305458491 0.4445334403620720 0.9885701388141186
0.8843267909769281 0.7764830245384786 0.0046244072985857
0.7171620388794722 0.6081929587772095 0.0007602666874007
0.5506886697301923 0.4428068946894044 0.9804908787823834
0.1616030445486416 0.1652916009112673 0.0039470317575834
0.9951220947768178 0.9985306725596956 0.0056406786207361
0.8282964247486504 0.8318631940938843 0.0061557621306250
0.9948615129735856 0.1650263944560422 0.0035924934579157
0.8282309865107820 0.9983648883586937 0.0052315578284379
0.4964328103008029 0.4994786643070768 0.9831805156566489
0.6618102891603925 0.8317651383071762 0.0034106517341570
0.8284616275804879 0.1650753909886831 0.0020965344403905
0.6616393291437390 0.9982422840729441 0.0025308623422333
0.4946684792029430 0.6652591778357220 0.9940908674884597
0.3284232896257464 0.3317124540214280 0.0019617856360554
0.3277070786344751 0.4995214269514818 0.9945572987212614
0.6617898942780513 0.1647929911837949 0.0007758226750028
0.4950713136443454 0.8317440347115976 0.9998089507967975
0.4952586304355577 0.9985546651044075 0.0013903841213008
0.3282594727465664 0.6656573639425858 0.9957741935175264
0.1611738224268322 0.3316690608716115 0.0001735542394101
0.3283491592097434 0.8320308196010799 0.9999219994026731
0.1614497313531871 0.4986495182675412 0.9970672770545903
0.4945879966118270 0.1644683543128593 0.0012120121696881
0.1617069119204047 0.6653624729409131 0.9982103771601132
0.3282437284652815 0.9982895297813670 0.0020242465878724
0.9949786872767348 0.3319497128198279 0.9994505531609974
0.3288493450487309 0.1653325649896757 0.0004110533479391
0.9949793335861656 0.4986147606739380 0.9974342076922795
0.1618428533458172 0.8320549791434644 0.0018286266389467

0.1618462188681136 0.9988899493245782 0.0042514931273985
0.8286932630366053 0.3318467024254375 0.9984538607023339
0.9955000875119850 0.6656343841250631 0.9989242117401389
0.9949833769115628 0.8319134900124894 0.0042248101748257
0.8289799146607209 0.4992325443792822 0.9932548981190337
0.8293810735487099 0.6653844636693833 0.0015955323157115
0.6625040080124407 0.3326309919402917 0.9989424579649508
0.6617985463569980 0.4978700403221907 0.9846630023184701
0.4944722007510975 0.3318799500990792 0.9879187642920471
0.6604685571359497 0.6645510824671987 0.0047204984087088
0.5006338443659644 0.5690115423393607 0.1978134348513194
0.7280248856017124 0.4892324179370462 0.1960351491174634
0.6650429338169062 0.5996405053496829 0.1181217435763386
0.3768644343356584 0.4222501348290777 0.1132387155480998
0.5331278772359482 0.4190955150262852 0.1704652282217012
0.6284876988117887 0.3315390762078749 0.1151367633251112

PT6-XXXII - ΔE = 0.75 eV
1.00000000000000
14.7772044699999991 0.0000000000000000 0.0000000000000000
-7.3886022400000000 12.7974344700000007 0.0000000000000000
0.0000000000000000 0.0000000000000000 18.0009597799999987
C Pt
72 6
Direct
0.8904270560712334 0.6117814800700121 0.0018092797423374
0.0570201390122725 0.6118159580995607 0.9979810344885348
0.2234136881232023 0.6116613319199972 0.9968396075758547
0.8905346008112885 0.4447790850845751 0.9993145836875653
0.0566550451808320 0.4450129844555302 0.9983820279243979
0.3896196443006539 0.6116768955150858 -0.0007114989592523
0.5575296685823141 0.6132611971279076 0.0047549275283989
0.2232085088709669 0.4453665649617416 0.0013472647855849
0.8905900950754595 0.2785534691405467 0.9963869636113341
0.0573404398778212 0.2784708947795223 0.9988554633693905
0.3881002590401987 0.4443566160713693 0.0153426809422275
0.7246661387704796 0.6131658368467172 0.0063363521057196
0.5568585362507823 0.4436458333499959 0.0154007806421585
0.2233669148582737 0.2779687767716770 0.0015048460230553
0.8903843174193634 0.1118807892882033 0.9947576171514781
0.7248770246823980 0.4445871915795061 0.0053523399065631
0.3897216622376738 0.2777960036025763 0.0007814537247409
0.0569858795579544 0.1117592797257694 0.9962041164666444
0.8902748607178951 0.9450580042157265 0.9948216007079974
0.5565629402442827 0.2777054834440417 0.9972687635118493
0.2233381838150475 0.1114104708698530 0.9952090852234580
0.0567182413684419 0.9450511542167433 0.9947141877089913
0.7235435943836895 0.2783486709651365 0.9956528692536509
0.3900341922563892 0.1116329813536613 0.9920962123873420
0.2236331013830566 0.9452154355851555 0.9925239144107683
0.8904714763540981 0.7788083263905200 0.9980592717536474
0.5572259092426938 0.1118125460819678 0.9907501843186195
0.3903779054878694 0.9450303641054054 0.9903186918501398
0.0569140358566630 0.7784022216388663 0.9963431985519189
0.7237455308557219 0.1116537798789588 0.9921786866050999
0.5569576497977478 0.9449631438108915 0.9903913090831190
0.2234656313893057 0.7784340135392395 0.9937808418665094
0.7238100951728587 0.9451148784866588 0.9926757195363980
0.3904116124049569 0.7785315851405235 0.9926089992170344
0.5569194250918403 0.7781799586213106 0.9943013731603711
0.7236040615218055 0.7785961740468201 0.9970488159660835
0.0006486700823322 0.6669428969321939 0.9986828869211535
0.1673168898210040 0.6670283381924613 0.9960985348185686
0.3340810870728999 0.6667600197046272 0.9956913625017743
0.0010141438061505 0.5002698988496510 0.9985776951983273
0.1674235172165583 0.5006549643118181 0.9986161393535262
0.6677157312110645 0.6673888357561727 0.0021682886967422
0.3327872226669875 0.5009514421983042 0.0044988133578882
0.0008495327092519 0.3337746525105539 0.9980973659040518
0.1671962830245020 0.3333914238112780 0.0014498674318887
0.5004991309399530 0.5017630099693065 0.0170301589384256
0.8340930033016296 0.6670625511065542 0.0021395737484456
0.6691258679611564 0.5009473432779629 0.0137941042137578
0.0010033979466814 0.1669752541031421 0.9968188250167078
0.3329857021130431 0.3320059479110645 0.0059706096716816
0.1672507645793160 0.1665537490735499 0.9974226936401847
0.5001171318427562 0.3320191968739376 0.0033436726502983
0.8346704871969711 0.5002553940645870 0.0023952062301106
0.3339708291845874 0.1666103826850345 0.9951640733327126
0.6679830743349569 0.3331720110826644 0.9993108310884350
0.0008681783158273 0.0000698423963468 0.9949851330868060
0.5009636621714751 0.1670189485869544 0.9922879419358562
0.1675838310388326 0.0002973966487881 0.9937690074607299
0.8345813375365418 0.3338090414818494 0.9971663986594933
0.0007438217119330 0.8336131862817684 0.9961690492305582
0.6673998266302799 0.1667364200941309 0.9922887354034857
0.3340527339336408 0.0003531468416043 0.9910531527445220
0.1673139313165553 0.8334142991047341 0.9939147103142011
0.8342612954620103 0.1668184739366838 0.9943689716623788
0.5007865281224143 0.0000782138178303 0.9898333783474483
0.3342096476865033 0.8336732563506684 0.9917538545444757
0.6675940229893735 0.0002407067010778 0.9912729413090966
0.5006644227860939 0.8334841876346941 0.9918187946897542
0.8341873797773098 0.0002303705510345 0.9938219489470186
0.6676164772443979 0.8335085420944701 0.9942628109570353
0.8344308745874840 0.8338388585008631 0.9964887830751430
0.5011988233460263 0.6673571818406639 -0.0011868420776409
0.5924338005956883 0.4127564147077864 0.2676529903791935
0.4435813122790260 0.4715584407071766 0.1303863409356732
0.6167623462768812 0.5881815198443412 0.2217046596729435
0.6093200396361576 0.4484616322053627 0.1282031176978080
0.4363779605696459 0.4462752355825635 0.2686535367367526
0.2884912772359922 0.4485441315059048 0.2056273024785743

PT6-XXXIII - ΔE = 0.75 eV
1.00000000000000

14.7772044699999991 0.0000000000000000 0.0000000000000000
-7.3886022400000000 12.7974344700000007 0.0000000000000000
0.0000000000000000 0.0000000000000000 18.0009597799999987
C Pt
72 6
Direct
0.2218393076920577 0.2753586962541945 0.0030487861311457
0.0562990106751684 0.1091301763384891 0.0029700847957486
0.8893487568516321 0.9422545002299927 0.0059116700783832
0.0560522638189767 0.2754073692931343 0.9993079633094410
0.8895105137963029 0.1092167070513028 0.9993054273950506
0.7233367841607929 0.7775312694408072 0.0100363586442640
0.5558409047982806 0.6089433048648800 0.0030642182921525
0.7233853148296987 0.9425982060038578 0.9986372799053456
0.8900886074534498 0.2758529060327959 0.9956861992585502
0.7229144420824767 0.1088211905148029 0.9937434922606272
0.5560323504076194 0.7761765462325769 0.9968598409686251
0.3902159189357519 0.4424600992420693 0.9986636264146114
0.3893129940617470 0.6101938939356799 0.9946889055267434
0.5564942606953167 0.9431454404849759 0.9941083478379511
0.7231743987543169 0.2760502879628888 0.9909085230177581
0.2228180564725193 0.4425555662952760 0.0000055807151256
0.3899363088807561 0.7763083820246806 0.9945369753002495
0.5569082852919109 0.1094421315168361 0.9916969342677504
0.5559475494065182 0.2753025926068986 0.9887315867660647
0.2225870791222135 0.6090178001892426 0.9973361264528648
0.3895387949985576 0.9424368849066909 0.9950270104032484
0.3897642918719697 0.1094294220594634 0.9947674591088713
0.0561402026679900 0.4425514003279432 0.9982648299137509
0.2229826725807555 0.7762419199751420 0.9984503268586948
0.2234359872655816 0.9427502648199635 0.0004213029373332
0.3903016458154411 0.2751819425899598 0.9966530084551337
0.0566621218339662 0.6091714081825970 0.9999607748761790
0.0562326259293116 0.7755790760349868 0.0046361120720135
0.2227342473506724 0.1087788880270679 0.0018616376332474
0.8896079720468322 0.4421228906314738 0.9966288550549649
0.8899961605812479 0.6093235259873211 0.0050547721656997
0.0561822069741424 0.9427801207317899 0.0056544654101813
0.7242776404121969 0.4418566949673917 0.9963212337312299
0.8900607390059392 0.7757329556649495 0.0119335816952955
0.7232546701260176 0.6075650159948509 0.0185063593788612
0.5553020078607389 0.4422237342938971 0.9883422574351570
0.1671498003907743 0.1643062056246336 0.0028900927727094
0.0011341452331877 0.9977258152206474 0.0051850282943775
0.8342789266764115 0.8317276396602509 0.0105389746724924
0.0008273516758521 0.1645711305630257 0.0007412371006552
0.8341413670929541 0.9977460210248879 0.0014490037405253
0.5021133733729641 0.4971777091630543 0.9886415925832779
0.6684086790505717 0.8319493443915604 0.0010956036475847
0.8345221417679198 0.1643548809962851 0.9958715399956759
0.6676660798529213 0.9982912996723670 0.9948638378128365
0.5002055799865843 0.6659599105178629 0.9957589964406921
0.3342172591290904 0.3308887739620374 0.0048730573087852
0.3332873034271984 0.4981984221243181 0.9995908119071473
0.6672795983294435 0.1642313370787960 0.9913974281528652
0.5006396532107900 0.8314301221979150 0.9946391053059784
0.5011818813057332 0.9980161056466272 0.9933755092278460
0.3342585549299457 0.6649807517505337 0.9950608158169345
0.1670733440829366 0.3304686449825311 0.0005062200835440
0.3341793576584990 0.8317858169746728 0.9958285666614088
0.1675207726684604 0.4981639195307039 0.9986479006179749
0.5009841783290128 0.1650325873772545 0.9911040925314865
0.1673620919401699 0.6645298958081653 0.9985984247087032
0.3339341316785465 0.9977796345656387 0.9966158243707355
0.0005276815442556 0.3310028944380363 0.9977244612276408
0.3346611889696334 0.1643544103436838 0.9979632947728092
0.0013890735893369 0.4979046775518512 0.9984207434369239
0.1677521004705369 0.8312781824548381 0.0012595659565804
0.1677157783173229 0.9984298174595949 0.0027696600324205
0.8344300678030407 0.3315305724283775 0.9940183944450069
0.0010563771545478 0.6648658238964913 0.0031393439821201
0.0004963526909592 0.8308886947156253 0.0076953581298795
0.8344720249948381 0.4976237622558983 0.9990180784051574
0.8352261904059191 0.6640835742130022 0.0130020322637350
0.6681987084116159 0.3309845601785995 0.9897856829998659
0.6667384670430536 0.4962656075249754 0.0047400835142284
0.4999229090126249 0.3302047154598995 0.9886814658477547
0.6665241376533686 0.6653275640383924 0.0174434198863267
0.5019141333797421 0.4023534740203232 0.2079875581522685
0.4534767486238209 0.5416423710042011 0.2353482870771373
0.3822338889015811 0.4335409511415662 0.1172130846589141
0.6226856719845699 0.3368246506313497 0.1821322899055460
0.5692736513575838 0.6219763499676105 0.1207272060543829
0.6548624401066121 0.5060455922111871 0.1228628401629024

PT6-XXXIV - ΔE = 0.78 eV
1.00000000000000
14.7772044699999991 0.0000000000000000 0.0000000000000000
-7.3886022400000000 12.7974344700000007 0.0000000000000000
0.0000000000000000 0.0000000000000000 18.0009597799999987
C Pt
72 6
Direct
0.2204716040200267 0.2775481315379125 0.9916576063613647
0.0539728341982624 0.1110403894561074 0.9901787687559320
0.8878985345450303 0.9449032580018866 0.9936280687296275
0.0538871045303821 0.2776640160705597 0.9938104567085446
0.8875894130736057 0.1109991074473982 0.9932250596840433
0.7221967200873678 0.7785431772607865 0.0027325057372565
0.5563971815001096 0.6115769251367880 0.0176272462274767
0.7210424283484187 0.9444197209674385 0.9983543396951831
0.8874067033123509 0.2776400098504723 0.9947535126871827
0.7211336564292239 0.1111881443485814 0.9966951997480535
0.5556485528253674 0.7798779069643729 0.0103000803940816
0.3866284400786171 0.4423374964363873 0.0033964219604528
0.3856654919275826 0.6108383091432827 0.0212684690642035

0.5546961280991667 0.9445559571337938 0.0012198581994909
0.7211593009434267 0.2777848834694367 0.9962797854376717
0.2190723660457579 0.4430376645649901 0.0038805686949317
0.3875116139229036 0.7779014250118195 0.0055927819942099
0.5545110105262623 0.1108737943570617 0.9977913476668405
0.5550588369330640 0.2773081702204081 0.9993284864299596
0.2207985558035688 0.6116298174970396 0.0021709374686480
0.3881376360132620 0.9448135114274763 0.9980789833839552
0.3881599072487916 0.1110738957102413 0.9948875359638549
0.0543397133172476 0.4443951376442286 0.9973077920943538
0.2214988676252574 0.7780831938096000 0.9970278824411736
0.2212169592266662 0.9445892879133808 0.9927970016054175
0.3865955320020902 0.2764951518326626 0.9945050872855390
0.0543554050160822 0.6111209698341682 0.9952349844937061
0.0548056512846742 0.7782169549073146 0.9930128744142550
0.2208734155061052 0.1113060880869270 0.9897130022611691
0.8880129883088443 0.4447091706242290 0.9950777973231340
0.8883704437094551 0.6113629891864960 0.9946306276028096
0.0546274073261586 0.9447482532581120 0.9908595351757796
0.7216803080253555 0.4448047618607305 0.9969745689788141
0.8881794725210952 0.7781587517830744 0.9949528066050704
0.7226116321805947 0.6118619496879560 0.0003124404285515
0.5561809840851524 0.4444336041637271 0.0036933202402523
0.1652950890810843 0.1667088162988222 0.9894455773527611
0.9987324444810852 0.0001897559649109 0.9909260448396395
0.8323331224681567 0.8335332084619296 0.9965761707001519
0.9983572797061413 0.1663762180216537 0.9916735638975069
0.8324198634768010 0.0001946550401897 0.9945995253163389
0.4983572819339557 0.4981649236549259 0.0103267683680102
0.6661095788473048 0.8344927130208859 0.0033368273166232
0.8319605829590984 0.1667914949484555 0.9947001083195417
0.6654019822215815 0.0000763385594453 0.9988361400203019
0.5006611102201362 0.6690526724035379 0.0221514513945067
0.3310301531715680 0.3325467267655071 0.9937177328678857
0.3306549638447649 0.4978050924204567 0.0116164009143844
0.6655997587626530 0.1667231020192261 0.9971776124789571
0.4992392119960130 0.8340211781052460 0.0062072779314732
0.4987699356155062 0.0001330390796710 0.9990184463189493
0.3311792647322349 0.6673880645633687 0.0090043473484727
0.1643737750943970 0.3327573818556928 0.9958662384635772
0.3321917260728853 0.8333704624289666 0.9999212653385854
0.1645546490560861 0.4997888636446248 0.0018475987669149
0.4987858172638511 0.1663068482888690 0.9974482136005278
0.1658528406406319 0.6670080339157636 0.9974482493147718
0.3325686998101745 0.0003993863839753 0.9949119003619913
0.9986299944339763 0.3334176911085436 0.9953029171852066
0.3321131271677658 0.1669920990154381 0.9919766512233892
0.9987793822932645 0.5004968941923664 0.9956646293759306
0.1659203706299905 0.8339578598291553 0.9938487595054184
0.1654803909100409 0.0002598984462168 0.9904913397537740
0.8320225625860616 0.3334037491218638 0.9953353923213228
0.9992495206087213 0.6670342360234613 0.9938977910259865
0.9992330668760445 0.8336884435889544 0.9924739867252015
0.8328319736414187 0.5005217567942211 0.9953199096919221
0.8330140549391700 0.6673922619621919 0.9962209035985055
0.6653344518478121 0.3332377569151106 0.9973719001759633
0.6679280066654201 0.5010455903999116 0.9997357725429978
0.4984532953137304 0.3322682304696372 0.0027758775062878
0.6675991184073951 0.6677171161835034 0.0071881729009817
0.4524522130162225 0.4845970656998730 0.2364756171968310
0.4328926163886919 0.6423914603559737 0.2468338757387656
0.3426884905436864 0.5360811096934341 0.1298260532989133
0.5413167712532356 0.6396550661233249 0.1326332073199339
0.4841365918980145 0.3373374400669960 0.2396087981173594
0.5003759862526920 0.4204312999272588 0.1160908316508227

PT6-XXXV - ΔE = 0.78 eV
1.00000000000000
14.7772044699999991 0.0000000000000000 0.0000000000000000
-7.3886022400000000 12.7974344700000007 0.0000000000000000
0.0000000000000000 0.0000000000000000 18.0009597799999987
C Pt
72 6
Direct
0.5573529998009680 0.9472708158187808 0.9932194842393570
0.5582814331138177 0.7811262370165795 0.9975999901008139
0.5592088133970827 0.6161120093229666 0.0097214838190026
0.7241707967264860 0.9475540845514483 0.9903829561082713
0.7243438748385781 0.7807542881355924 0.9937521173653129
0.5594772755846108 0.4472260176004407 0.0179240228385216
0.5578410479724201 0.2810883046090367 0.0015514828525995
0.7256829288930463 0.6151813315288229 0.0017463734687717
0.8910994718674349 0.9477574341577639 0.9899196828775007
0.8911586384387471 0.7813135170382489 0.9923194044328613
0.7250455106941742 0.4476083022940509 0.0015857584910827
0.5574492957498673 0.1143747857718938 0.9945419403536712
0.7241388924839720 0.2809181482703906 0.9948938147884652
0.8911599285987885 0.6142248472201585 0.9953607628303045
0.0576245342104355 0.9476928391186658 0.9919450578126829
0.7240739606847808 0.1142835384611521 0.9910082904587670
0.8911948094634409 0.4476588880797324 0.9943273485019403
0.0579553917972787 0.7812044008503481 0.9928677099130517
0.2245216007989228 0.9478014397881225 0.9941726850338722
0.8905508765006420 0.2807448379577184 0.9923655728853408
0.0574156619487008 0.6141284241597217 0.9946535263014886
0.2243137873881376 0.7809604348907114 0.9960107192660949
0.8907147119782595 0.1141164347566543 0.9906383805984537
0.0571720823430422 0.4472504903286861 0.9955750768315932
0.2236154644233805 0.6143494602889509 0.0021335682438716
0.3912456895100135 0.9478913595996197 0.9949363980353477
0.0570663267912905 0.2809182334014238 0.9970987607222318
0.2219665700858824 0.4469246525264208 0.0091290195193371
0.3912237149760629 0.7817846804421734 0.0003485418988001
0.0573608647896415 0.1142903700692717 0.9945055877547384
0.2220612964895539 0.2795494887683915 0.0079752073844923
0.3921485550967917 0.6158636226167583 0.0146076734094931

0.2244105260580471 0.1141658272382972 0.9980878712519792
0.3893204675304471 0.4461753797297467 0.0284297008017393
0.3912831946369407 0.2796333185436543 0.0107738439981922
0.3909563858409537 0.1140078571835108 0.9981494273956686
0.5019204308014354 0.8367161336285643 0.9966496785114298
0.5043428048949122 0.6712866407345409 0.0062411855671807
0.5045328256353847 0.5055427563752134 0.0252168286093511
0.6687180118983143 0.8364633431541774 0.9930889163684579
0.6696925670736719 0.6707443356105572 0.0007120273484794
0.5019299858280949 0.1694660554535697 0.9978336218977670
0.6706057415648990 0.5037918754540618 0.0082028752421407
0.8356884361597071 0.8364370743435784 0.9911287136948389
0.8355107380597132 0.6700000232761149 0.9958477941396540
0.6690212603536947 0.3366255181330209 0.9994804259563117
0.5016931091607908 0.0030283931275345 0.9940274458553616
0.6686596592615939 0.1699449073831735 0.9931827545840148
0.0024535647221433 0.8368803280071879 0.9917075219497562
0.8358990070310856 0.5035472521851677 0.9967633626908251
0.0021967732879452 0.6698639809191114 0.9937186380074723
0.8352870882916753 0.3364887376850859 0.9935905274347191
0.6685280597458715 0.0029519730128608 0.9910205454738310
0.0017862576073052 0.5031820647854507 0.9941464399613328
0.8350392010974815 0.1696789426657191 0.9909417477204205
0.1687259686016032 0.8364295826652276 0.9937868154009641
0.0015915762469971 0.3364008726039874 0.9945389797893732
0.1687126010286067 0.6697431184527766 0.9969996859773063
0.8353747534121483 0.0031230264903624 0.9896295833835399
0.3353394550650734 0.8365885444717803 0.9968222368924828
0.0017238647817047 0.1696688416334211 0.9937389713064844
0.1675040507202271 0.5023486855267375 0.0012177516875731
0.3346569010061013 0.6704776837663902 0.0072762111571955
0.0019289354793715 0.0030639518529796 0.9914352765013197
0.1672922987940846 0.3363857508078630 0.0049697147754029
0.3329449267118397 0.5037456848055951 0.0240457830997702
0.1680929931286561 0.1696670523040762 0.9997995045002076
0.3338036653223438 0.3348672760134928 0.0160669531596571
0.1690198445478545 0.0035226021692506 0.9940176362489779
0.3350157572856468 0.1690380366881448 0.0019960685230534
0.3353105010898869 0.0031809786859152 0.9952799167425214
0.5026139668350931 0.3353997177407597 0.0092345315959220
0.4223142357502194 0.5176679076455410 0.2232263493388719
0.5510680733201369 0.4889308460490341 0.1365337697936430
0.3586978223941609 0.3541897688184024 0.1334937152569770
0.4621233023186224 0.3746814398849627 0.2556135569279935
0.3636875615091216 0.6401576134312514 0.2618656612642530
0.3500699387802129 0.5928011538460923 0.1280075265432776

PT6-XXXVI - ΔE = 0.79 eV

1.00000000000000
14.7772044699999991 0.0000000000000000 0.0000000000000000
-7.3886022400000000 12.7974344700000007 0.0000000000000000
0.0000000000000000 0.0000000000000000 18.0009597799999987
C Pt
72 6
Direct
0.2183860683673515 0.2712690242977089 0.0021695731169800
0.0522652004828714 0.1049613801941263 0.9961985283111758
0.8859501770640250 0.9387108202091241 0.9960839893572881
0.0515418148933264 0.2713483732743072 0.9979026001022859
0.8856592867582478 0.1050798753682471 0.9973331959410530
0.7200690912772174 0.7723558930062850 0.0027709299596950
0.5549731552540393 0.6047419442365793 0.0209311106266554
0.7196663568418202 0.9386575271411246 0.0039712746749743
0.8853107412057497 0.2713333148608044 0.9952836972538250
0.7186947326164415 0.1048680132777520 0.9999186893388625
0.5544882663369606 0.7739824664176922 0.0190444059089501
0.3837839000790879 0.4360059857539298 0.0128210078907856
0.3833064135343207 0.6048041665525901 0.0219809204818233
0.5525854485467292 0.9390839363252681 0.0071297449609773
0.7190656128358199 0.2717858387746617 0.9957072920807448
0.2166506018689063 0.4368819022016126 0.0073091087622572
0.3851370891768298 0.7716513973745904 0.0035631311771880
0.5524278739703519 0.1049593140500846 0.9991424074000221
0.5523675958081498 0.2715424852790775 0.9979302361833149
0.2188341013331012 0.6053110496981731 0.9963573273121256
0.3857495001189122 0.9386109292885791 0.9982009182911540
0.3860409186896376 0.1051948713928468 0.9969243001386303
0.0522578345891489 0.4382551670271937 0.9943765114756360
0.2193194740003079 0.7717739037859772 0.9920345114772147
0.2192457977133913 0.9383121547001565 0.9927926472334150
0.3858496097288935 0.2712222086571998 0.0010497790048021
0.0522646473575463 0.6047369259107356 0.9892146055462234
0.0524691324869337 0.7718581479759195 0.9889508700849277
0.2190395248584167 0.1050870351579647 0.9962249771190628
0.8855644401101799 0.4383920101822980 0.9912595078427984
0.8858470150139297 0.6051019618144124 0.9891984832410401
0.0526617422022468 0.9386560347778286 0.9923480701965346
0.7191012826007110 0.4382808894302315 0.9945714410947062
0.8860115135825950 0.7720985664867683 0.9915937607594856
0.7199454806819006 0.6061757874655953 0.9961445906444624
0.5528716836731746 0.4376195271724512 0.0052905725570369
0.1635447361022813 0.1608116733352816 0.9978309974857780
0.9972259508906234 0.9944289737502316 0.9943318655146669
0.8307101280924698 0.8278350877702678 0.9959679553770897
0.9965875375806021 0.1604944928076506 0.9967773686897416
0.8305761895172665 0.9942505086301594 0.9987503277670271
0.4960618989976879 0.4928073160679389 0.0134776970128030
0.6658882531613770 0.8292052595983250 0.0091594950169025
0.8302649053964046 0.1609259046981464 0.9974850428965425
0.6635795418779793 0.9945055620228160 0.0044302641324379
0.4981367454186980 0.6623093141376160 0.0283142708614861
0.3297958682130258 0.3272978425649384 0.0050123701989477
0.3278580378874452 0.4917901710536015 0.0186364448796894
0.6634513914306908 0.1607579424361134 0.9985400786830780
0.4964497275819895 0.8283898237151988 0.0129362762088476
0.4969600704315766 0.9945910210932993 0.0016430606227900

0.3294247180951402 0.6614017457494015 0.0039706552133865
0.1626215506574695 0.3267660526405578 0.0025558287732110
0.3301975595215083 0.8273584033802734 0.9970113697298331
0.1624115288995398 0.4935831473526378 0.9989675517358449
0.4966976730707486 0.1606422636204812 0.9976957918426166
0.1637775372458705 0.6607700785749913 0.9911104294024469
0.3303957944010989 0.9940111919303121 0.9955976424820250
0.9965576697158269 0.3271127460050707 0.9957767983776620
0.3304839679308245 0.1610773454006207 0.9976392521953699
0.9968984559852530 0.4944542021725766 0.9906980846424460
0.1639471355400630 0.8277515353175104 0.9905594587883684
0.1638470917086323 0.9944309706930028 0.9933014351815714
0.8299971620654816 0.3274137915623214 0.9939431315776872
0.9968951699281661 0.6609127100592218 0.9882852016695409
0.9972341823972002 0.8277747640886730 0.9902866847290426
0.8304127284940250 0.4943272737803568 0.9910390750322904
0.8304952681888338 0.6614501244344098 0.9908048157017575
0.6636610320639420 0.3276695672098526 0.9959207738000586
0.6643158463466676 0.4943837643593696 0.9985523534076535
0.4965124575440640 0.3262408790060434 0.0011952594806175
0.6651480606645706 0.6619614342014444 0.0043433920687832
0.4794433909656561 0.5750422357152942 0.2110243514251380
0.3242318603135104 0.5535124607322572 0.1320147985186964
0.3826469036490749 0.6724127999042722 0.2480314709214255
0.5060218380778778 0.7357947359355208 0.1339724705789891
0.5979282864550868 0.5138720726719725 0.2568049185480703
0.5682988464833798 0.5184002054209031 0.1214692373052202

PT6-XXXVII - ΔE = 0.79 eV

1.00000000000000
14.7772044699999991 0.0000000000000000 0.0000000000000000
-7.3886022400000000 12.7974344700000007 0.0000000000000000
0.0000000000000000 0.0000000000000000 18.0009597799999987
C Pt
72 6
Direct
0.5549961734347149 0.9441856891287725 0.9933534508233506
0.5545242997961309 0.7778290255466574 0.9993753911883684
0.5556397144605436 0.6123322477800694 0.0152482804344528
0.7209425388231476 0.9435652926320064 0.9948381447405045
0.7222524270330846 0.7778745660365788 0.9999905935207765
0.5560864901190496 0.4433306560583756 0.0082772505583082
0.5547710923045964 0.2775821354960562 0.9981793620803581
0.7224098473219951 0.6113086375105734 0.0010101293200904
0.8882158192218900 0.9445187783083070 0.9943055661125393
0.8880414228478002 0.7773608885122899 0.9947666860086188
0.7212131740424337 0.4439579317800336 0.9955558561905088
0.5544565261999094 0.1106231851291028 0.9932881675594895
0.7214050447826471 0.2773928957219970 0.9929092568803216
0.8885131582257122 0.6105295900340326 0.9930532046509359
0.0550263511126496 0.9441091929968453 0.9933969724980176
0.7213356777234905 0.1109469072313234 0.9923642949370588
0.8880108456644561 0.4442749290722566 0.9921244155509837
0.0549134849484293 0.7776329112404847 0.9920393468759857
0.2212083788873987 0.9439186164142374 0.9929823652914447
0.8876089394643328 0.2771852671587540 0.9927666120586701
0.0544598154744839 0.6103276546820354 0.9925247641126995
0.2218068615503915 0.7773784883905840 0.9925507201152175
0.8882008162064405 0.1106189544042215 0.9934714037189524
0.0546082111379156 0.4437753342107364 0.9951562361991322
0.2212922018697913 0.6109958182990440 0.9974754839617839
0.3881650349228921 0.9444063596078434 0.9926460966241171
0.0547555404722104 0.2777407389384595 0.9967955954016688
0.2195193632797014 0.4434478314609853 0.0062331469914692
0.3879749098453981 0.7775387230652322 0.9954655920497151
0.0543051380883153 0.1105381391492605 0.9950839197925132
0.2211373584484809 0.2765313680239309 0.0031344981318760
0.3887803431747082 0.6125136672330045 0.0063712188932575
0.2214573679200057 0.1108837600186953 0.9968830205296485
0.3863005867829898 0.4421987334576558 0.0220109712926100
0.3875320937912104 0.2766828172448734 0.0047350420378896
0.3882706793723901 0.1106133162478476 0.9958737061888816
0.4991357558145610 0.8331389935061100 0.9956585956814675
0.4983726514879112 0.6672960977274416 0.0059481888408464
0.5006510011636028 0.4994918744369267 0.0198105575521003
0.6658441163885731 0.8333835557300805 0.9982338693447720
0.6674567386399538 0.6673096232451051 0.0058842014291944
0.4986182717572827 0.1657989183386492 0.9956455013459361
0.6663011738254652 0.4990547866272814 0.0008619769917034
0.8324495106603124 0.8327693547622963 0.9960749851770770
0.8324936670290991 0.6664962091315383 0.9956023143542934
0.6655269208602016 0.3329484976543177 0.9953498326005111
0.4988221620673857 0.9997508192774552 0.9927039020300258
0.6657192383516594 0.1662399158966003 0.9926657196593837
0.9994043413380748 0.8330035671598210 0.9928930206070226
0.8328947736785267 0.4998112211661202 0.9929808293725186
0.9989798354258816 0.6658941744338908 0.9919872926605756
0.8320039975695085 0.3326382294598736 0.9922367651829190
0.6652672683968304 0.9992439088529329 0.9932610436340015
0.9988533370864690 0.4997750471321251 0.9927421142076706
0.8321363776301718 0.1663686928690460 0.9926976038538377
0.1656864384891955 0.8330155695882420 0.9919769112803996
0.9990504501561109 0.3329385667120442 0.9948029507298608
0.1661973503381990 0.6660701017180548 0.9937432391945090
0.8326631100977778 0.9995330875833782 0.9940828713322446
0.3320900856913986 0.8327278070877331 0.9929998363588393
0.9986651522342419 0.1657467467031419 0.9948163941225592
0.1648552596358821 0.4990751360276554 0.9992144755390224
0.3325544478282266 0.6668912596760310 0.9991795114371224
0.9991065041999375 0.9998802840105713 0.9938800600414980
0.1648557622840679 0.3327578781849466 0.0023837562591654
0.3311778269568862 0.5004036313194339 0.0171757804505903
0.1659866478092885 0.1663502175150838 0.9977855579363621
0.3310882048185231 0.3308808994484325 0.0096888303311712
0.1655040939669699 0.9993741805159939 0.9939982203960136
0.3321434682841114 0.1662484507565396 0.9988867971083683

0.3324698032985960 0.9996067463840114 0.9934656954931248
0.4994210693967796 0.3325275260699773 0.0042730569638375
0.5530483108026575 0.5576507642625376 0.1298778094543890
0.3817203557864544 0.4311608798538161 0.2681404994911284
0.5818594415334104 0.5239718875008316 0.2638555929768968
0.3482359949749246 0.4724750457454805 0.1331062996476865
0.2820293356125836 0.5306755899735119 0.2457267811576967
0.4843741758138405 0.6278157922705255 0.2454941031862309

PT6-XXXVIII - ΔE = 0.80 eV
1.00000000000000
14.7772044699999991 0.0000000000000000 0.0000000000000000
-7.3886022400000000 12.7974344700000007 0.0000000000000000
0.0000000000000000 0.0000000000000000 18.0009597799999987
C Pt
72 6
Direct
0.5588994695677163 0.9468258537248003 0.9946279409688217
0.5588071001174651 0.7803640159301067 0.9994082082342061
0.5596268038062943 0.6155312717256671 0.0109215204385884
0.7257203865386209 0.9468506768758812 0.9948965582414523
0.7262202982933045 0.7810771621763245 0.9997480337357864
0.5613397915083889 0.4480548067522347 0.0115185776360013
0.5604627840330630 0.2794435830394990 0.0077430005727582
0.7269725736541111 0.6138418113807885 0.9983891802023381
0.8926874928045951 0.9471344898173635 0.9944814570535769
0.8924859977165741 0.7806845489873104 0.9932766154065883
0.7258891107898588 0.4466190883531219 0.9961926140666976
0.5592170907911076 0.1135584517464382 0.9973759165907354
0.7258134299507719 0.2798394820944848 0.9951852995799051
0.8921293943323523 0.6131490969065632 0.9904654802291017
0.0588404560634143 0.9469324987773130 0.9941835524072387
0.7259344261116846 0.1133515912105816 0.9935763637154339
0.8924334916267808 0.4465636747000420 0.9910347277524617
0.0590381630344294 0.7802640706629688 0.9904582674453755
0.2256019676175706 0.9470686433781428 0.9942401187045007
0.8925202895985125 0.2802327850537854 0.9922094341439825
0.0588314912664122 0.6135228578765838 0.9902957077774659
0.2254644846443924 0.7801994043070687 0.9916455254893006
0.8923608124476429 0.1134552153724444 0.9937091833960200
0.0586996770762629 0.4466318620675069 0.9932472163215281
0.2255349495967521 0.6136539525434946 0.9955126102814398
0.3925713957318234 0.9468412850855974 0.9940642637217113
0.0591679111407934 0.2802142880448079 0.9965644891558819
0.2249486591507046 0.4472232229346266 0.0039795581084411
0.3922925434700436 0.7805816145728129 0.9953943818185591
0.0589925354349988 0.1137027177658040 0.9976355638535243
0.2233664102559814 0.2790199819343147 0.0100264155363118
0.3917608610220894 0.6142885303286896 0.0043416894225144
0.2256038940191779 0.1130985859982695 0.0033477107141829
0.3911880940704529 0.4478416732883119 0.0227875168768915
0.3920031800571435 0.2775502221429258 0.0241534147438481
0.3919515687368814 0.1126328820651275 0.0020721646373465
0.5031112347896010 0.8358844007880528 0.9961711360737487
0.5027675882089643 0.6704529337793751 0.0049267720034040
0.5051236818552038 0.5036057270807665 0.0177205544082726
0.6702894728851978 0.8356414115219827 0.9979207397866457
0.6715663872552310 0.6700757642151089 0.0057077383667879
0.5038128234194164 0.1682973597632754 0.0031354711930550
0.6710337192309195 0.5022380066613115 0.0001403043217152
0.8372919374958202 0.8359938262561869 0.9956015367233400
0.8364781176539576 0.6687216819901833 0.9929285027683241
0.6707128353192688 0.3351575749389900 0.9995255363845353
0.5031604897468199 0.0019154390202256 0.9949395113894614
0.6701849623944096 0.1688450878224941 0.9953919429489438
0.0032886596559429 0.8358373675409623 0.9919343037840734
0.8366794874789647 0.5021958774560817 0.9916100310202233
0.0032838584639468 0.6688222707909509 0.9895841548413017
0.8371377791141639 0.3356135641277195 0.9921864596531176
0.6702034132729172 0.0021816771689441 0.9942469519911065
0.0033360488182553 0.5021818074616310 0.9908576276043108
0.8367675256150061 0.1687593083847034 0.9928720187329994
0.1697474077505728 0.8356182395257187 0.9913363571703269
0.0033982428477885 0.3358904438431248 0.9934572581425077
0.1699819036850967 0.6691759821882650 0.9918622004437476
0.8368540160303733 0.0024131521341917 0.9943010417263486
0.3366728980175768 0.8357885107447044 0.9930950745357083
0.0035316849358095 0.1688906132048285 0.9957484334198949
0.1701047219670994 0.5024341864741146 0.9969786384560926
0.3363190448509795 0.6691592529528045 0.9980108473751106
0.0034481557338296 0.0022027364973600 0.9949910080761095
0.1689509829688124 0.3351339847773356 0.0029918059731417
0.3355994503194556 0.5033073719622877 0.0108704912913661
0.1694468554958484 0.1686657880233683 0.0038210867095670
0.3336340714562970 0.3343327133897058 0.0246147543722459
0.1703250006129815 0.0024604320842187 0.9965239933746537
0.3360042655774408 0.1671144502322459 0.0090911571605069
0.3366966631516315 0.0024894103011549 0.9958247325173522
0.5056471483614161 0.3359417967808160 0.0179177689972753
0.6450630870556170 0.6448127901518326 0.2517843491672286
0.5045567147166961 0.4628404199054423 0.2446093776106372
0.5753627040009264 0.5775042293676221 0.1248737249715015
0.4606182593824211 0.3041846405152930 0.1349663762824708
0.3499512152904698 0.2834312794457175 0.2507737330396541
0.3565041493311566 0.3983011517160435 0.1368275520607608

PT6-XXXIX - ΔE = 0.80 eV
1.00000000000000
14.7772044699999991 0.0000000000000000 0.0000000000000000
-7.3886022400000000 12.7974344700000007 0.0000000000000000
0.0000000000000000 0.0000000000000000 18.0009597799999987
C Pt
72 6
Direct
0.2220736029985417 0.2781573262249069 0.9988237940180156
0.0554905332658606 0.1120888061374785 0.9965881334954716

0.8893652056364019 0.9463080639974919 0.9980268777545689
0.0559262368179176 0.2791866223562351 0.0001498133623343
0.8894523599943369 0.1125058381630168 0.9956682818476281
0.7236585415170183 0.7807307901693079 0.0035438745551417
0.5552087639461973 0.6128695777873148 0.0090985974849573
0.7223611642406169 0.9454596744732982 0.9934902528346043
0.8890406527858730 0.2786895100055915 0.9972769802751387
0.7224867244711746 0.1124954737701316 0.9914172754174047
0.5555125632523996 0.7794552027164201 0.9936960879134489
0.3880183989699830 0.4438391988443726 0.0075637704141656
0.3892266577296155 0.6136044210650127 0.0017497125090884
0.5560873314923853 0.9459634590486132 0.9898290813997193
0.7227796959823465 0.2791975216872800 0.9932334833527392
0.2212746847997025 0.4450261928675303 0.0080720994591843
0.3889387153963213 0.7790389984556967 0.9940565258228702
0.5558122131538710 0.1122491757907440 0.9886024802218714
0.5555741160204875 0.2787669332567617 0.9913676782151200
0.2221121508591111 0.6123791868981598 0.0019129954141519
0.3890536243525773 0.9457749315927586 0.9905723240013558
0.3891154171843638 0.1121622769953987 0.9892420767643699
0.0557920437734154 0.4454442920763952 0.0025023753262658
0.2226020526956489 0.7789818835931186 0.9969123704260028
0.2222308123843959 0.9453772527626612 0.9937419383639181
0.3884255521186688 0.2778446169422182 0.9940262792341343
0.0556179237731698 0.6118347313912480 0.0016619494406669
0.0558170399788906 0.7790547627632307 0.0000770523736549
0.2221557841322621 0.1120602251495200 0.9930684845851658
0.8892766085611470 0.4453756607764632 0.0000677653864116
0.8897960035069801 0.6121526832444175 0.0043177060724489
0.0559725161272624 0.9457463536039370 0.9972324644401098
0.7233061204229401 0.4446127061422942 0.0029486874043414
0.8894491339812944 0.7793775493373616 0.0032071785018815
0.7245600269794750 0.6133443760509323 0.0109242404030354
0.5551490983336151 0.4437521758855851 0.0086782004780162
0.1665991429340892 0.1675000665328668 0.9956157929157996
0.0001958381380547 0.0013860599450126 0.9971514914398796
0.8336686306662529 0.8349654223388114 0.0018551449935060
0.9997761741680051 0.1674982394215760 0.9973586035685145
0.8336970416473761 0.0012325788190068 0.9956524457236000
0.4992952422340053 0.5001447486970818 0.0053584686677226
0.6671796387301185 0.8353333795365643 0.9958818211088385
0.8333478540724570 0.1679260966847522 0.9945923674667796
0.6663203905946276 0.0010927996651446 0.9907032033164853
0.4990819192612079 0.6689850054798114 0.9981549941676775
0.3318184579827701 0.3321722725067957 0.9997043215782568
0.3329000332162195 0.5018495408759165 0.0119596487476699
0.6667192106477131 0.1677497485004196 0.9903420163416570
0.4999008143563444 0.8346020734182105 0.9915403251185069
0.4998652452787269 0.0013183692551522 0.9890361224853947
0.3331907582703764 0.6683228804643804 0.9988940238467023
0.1662046639629224 0.3342913385904254 0.0029117556266200
0.3328950483712489 0.8343368642221307 0.9935900193179208
0.1660500492131902 0.5009003356319681 0.0050394287208420
0.4996635983376905 0.1676364406064650 0.9884848522179013
0.1667362770799201 0.6676005498680055 0.0002695719680048
0.3331596694422956 0.0008328008980314 0.9907127413492702
0.9999818027169027 0.3343054517073369 0.0005870717528893
0.3327929047257356 0.1670949661419456 0.9909566119284321
0.0001025596978295 0.5011873066691166 0.0014660958491248
0.1664252981463648 0.8343107288139961 0.9968450721155335
0.1662822760709659 0.0007438998786782 0.9943253631994864
0.8332188188883123 0.3342787266540981 0.9969490393999649
0.9997105892505402 0.6674454303032675 0.0021880743796672
0.0001588591747606 0.8344999446596475 0.0001589470200187
0.8339261938219096 0.5007866011977669 0.0026789133515592
0.8338996888203525 0.6682302164692189 0.0061924978827292
0.6670892521580356 0.3345993140400196 0.9947344190708733
0.6682726847695974 0.5007639139281537 0.0127667967027705
0.4990678914485400 0.3324077988347796 0.9955781605937659
0.6683418786857303 0.6692829049608164 0.0124283023490932
0.4732356333968895 0.5898837523208229 0.2441406377499078
0.5910467564907051 0.4644974529719192 0.1252396011305592
0.3872542239422998 0.4806280615839853 0.1256790590724890
0.4666051798354758 0.4150873908465371 0.2305640855876234
0.5269543207689154 0.2924625027916576 0.1910492827907433
0.5842881114841774 0.6387765716948834 0.1252886787871574

PT6-XL - ΔE = 0.80 eV
1.00000000000000
14.7772044699999991 0.0000000000000000 0.0000000000000000
-7.3886022400000000 12.7974344700000007 0.0000000000000000
0.0000000000000000 0.0000000000000000 18.0009597799999987
C Pt
72 6
Direct
0.5539031079790682 0.9431128864159776 -0.0075111965465415
0.5545868455366043 0.7784715005788979 0.0079562394639171
0.5566591021628560 0.6115060154891543 0.0289307570324610
0.7205985030689128 0.9434162567997963 -0.0039435780856114
0.7226390760481280 0.7787668822683127 0.0099976955983573
0.5546315278636499 0.4422196456284979 0.0089736471941260
0.5541192185052438 0.2771184019023329 -0.0074345102636092
0.7227135211837300 0.6098204401488623 0.0091448054790312
0.8877968181029809 0.9439856003388287 -0.0040793742528190
0.8877167025602674 0.7769798783506952 -0.0008086110683615
0.7206449289329462 0.4433398224761320 -0.0038427431185481
0.5539846393894396 0.1101788677645716 -0.0119469938027069
0.7207034103567886 0.2767296569233065 -0.0106604729042719
0.8875332854685286 0.6097698194692970 -0.0043500048090970
0.0539547391226794 0.9433872062280880 -0.0076006908021486
0.7208676207451409 0.1101090064648714 -0.0107473455152553
0.8874513046213034 0.4434800017756258 -0.0082473914249200
0.0540687427105095 0.7767822832791017 -0.0073749745725387
0.2205548120278594 0.9434784509199842 -0.0101664441533372
0.8875905699005198 0.2768957201313085 -0.0095570012401258
0.0536711594559927 0.6099596564869390 -0.0067493040703011

0.2203910337180758 0.7765974159552713 -0.0069142507030985
0.8873470932201617 0.1099504311310724 -0.0082836583092757
0.0541155754383845 0.4434524002628455 -0.0050263418640893
0.2198762361065701 0.6102141808588134 0.0008783137420803
0.3875286521548366 0.9436218200527006 -0.0101485188050373
0.0543925625641912 0.2769014134590721 -0.0051647392270263
0.2202527617800464 0.4432280921405639 0.0047571169409407
0.3867399955467126 0.7770094267967156 0.0000718485290451
0.0538672766030855 0.1101383638705429 -0.0074392073641460
0.2201937001716780 0.2760538118256931 -0.0004067915704716
0.3844825725438105 0.6097265408392015 0.0203378152340443
0.2207286834582257 0.1102469355218559 -0.0081418020467285
0.3850532207866978 0.4409306305427654 0.0213423111837966
0.3868669010041474 0.2764548499448985 -0.0012335875618059
0.3873965876627254 0.1101580162218479 -0.0106272555784736
0.4982737412362686 0.8325476568577281 -0.0007010703439833
0.4977503399235995 0.6678735250195460 0.0246935088917024
0.4980111315831441 0.4961220422880363 0.0255650324437380
0.6649468231327027 0.8327963263500701 0.0045016742327726
0.6684451648586542 0.6670512299060374 0.0196745238323981
0.4979121589531966 0.1654740400131426 -0.0108313829435042
0.6652106981106449 0.4982740655604164 0.0046836174440792
0.8321367143260140 0.8327653343117927 0.0016034607813578
0.8318341576526836 0.6653205262811889 0.0010474746498878
0.6645858618126796 0.3321633587459203 -0.0078301409134069
0.4977318998729346 -0.0013475541321666 -0.0107024413839586
0.6647171179400425 0.1655209166324073 -0.0117416608518423
-0.0020899490777973 0.8321565758863836 -0.0058774357250030
0.8316449807507857 0.4989131096299687 -0.0058175407346934
-0.0019672957522827 0.6654284973000754 -0.0067465058405400
0.8314225424737336 0.3321051177590949 -0.0098691280631755
0.6646809495160638 -0.0014509060679325 -0.0080096993309944
-0.0021009550090220 0.4988812921509823 -0.0069856423825593
0.8312701561795479 0.1653122554357608 -0.0099168787792073
0.1643965317644748 0.8320774423773107 -0.0088756505539202
-0.0018505529795799 0.3322229653050110 -0.0067240553409343
0.1644283573236063 0.6657318717428123 -0.0048963845185928
0.8316604994848292 -0.0009991164834916 -0.0056706202143286
0.3312600207163186 0.8319603673338916 -0.0064029439168491
-0.0018641424107514 0.1652452224500490 -0.0073140298502454
0.1641875161624113 0.4988676652977585 0.0002087381690159
0.3297623824695803 0.6660975065255130 0.0073682584479706
-0.0019967320917720 -0.0011893548189985 -0.0070174804186391
0.1641027272669796 0.3314321103534392 -0.0000697429891150
0.3285242763538929 0.4973253538611407 0.0153231913230719
0.1647148366351116 0.1653305076007414 -0.0061463539276331
0.3302490020914283 0.3302425283785531 0.0059016947632160
0.1646148562406206 -0.0013197287220163 -0.0093969228788419
0.3312282298523804 0.1656721080969054 -0.0077406732145232
0.3313768908915927 -0.0010578052793838 -0.0111545242403237
0.4983769778257393 0.3322747158956869 -0.0005600421342432
0.4645695803130079 0.4846128925381585 0.2752607390200584
0.5420912660367289 0.6777834712841090 0.2595574516201528
0.6225634473807636 0.6552183294438965 0.1372055212334942
0.4413391553290765 0.4658500147625892 0.1400310806930508
0.4051361498491109 0.3099513128005503 0.2203076778161870
0.4389340980875788 0.6462007197275927 0.1372856482940362

PT6-XLI - ΔE = 0.80 eV
1.00000000000000
14.7772044699999991 0.0000000000000000 0.0000000000000000
-7.3886022400000000 12.7974344700000007 0.0000000000000000
0.0000000000000000 0.0000000000000000 18.0009597799999987
C Pt
72 6
Direct
0.2225230807172736 0.2768335652330904 0.0056384627369539
0.0573122331933931 0.1114713883358718 0.9972150298914499
0.8910618341927758 0.9452707210655049 0.9957880937235188
0.0573905614440235 0.2779911863667195 0.9997557904801795
0.8908146432980573 0.1115075496326341 0.9944109417789591
0.7248807289060437 0.7792920109579882 0.9992924448067058
0.5578804503503036 0.6130778015142795 0.0073427061430706
0.7242008720871027 0.9447597433226562 0.9930652176678478
0.8911796746475389 0.2783159675626266 0.9949512205541495
0.7244250371488974 0.1115646500206751 0.9916232673758572
0.5572864865887794 0.7786501315065593 0.9948097044677269
0.3920765869666880 0.4445408351071407 0.0218895894979383
0.3911290638842644 0.6133423129225690 0.0041864874408617
0.5575916148891835 0.9450753279587190 0.9911127580984740
0.7244761384664500 0.2781310677579256 0.9926270898880531
0.2228299292558447 0.4445365271743000 0.0135036796265311
0.3908046345275267 0.7784665417417074 0.9936791257329034
0.5577925359387663 0.1116424082054195 0.9920467387245608
0.5581213925154245 0.2782172395952216 0.9960758653362802
0.2240701010641928 0.6124738619358538 0.0015362673668875
0.3910521244391418 0.9450670204651104 0.9924773592061982
0.3912058634242896 0.1114566855610022 0.9962738962949302
0.0568817375267372 0.4448054079179329 0.0014571463406909
0.2243542924568942 0.7786312940167761 0.9951991036827081
0.2244178123306000 0.9451357122202921 0.9954432354530383
0.3915696087187541 0.2771168999964360 0.0066223256201212
0.0574787325712904 0.6116328874532400 0.9977188038197369
0.0576526154849262 0.7784738129721518 0.9962617555235198
0.2242720653136914 0.1114556400203739 0.9991289921784059
0.8909338660219390 0.4448538186872142 0.9952317578380061
0.8910547022652082 0.6113861712356368 0.9960192221432536
0.0575243696419463 0.9451161475168703 0.9965067691116118
0.7246664801036218 0.4446862397852129 0.9943210225215751
0.8910647782128720 0.7785081991450795 0.9976908296980156
0.7252457310912988 0.6116103999004494 0.9999771281711674
0.5593206140363876 0.4444722393935976 0.0029797980979893
0.1683676860250927 0.1668327157518732 0.0000463787593077
0.0021182345376758 0.0005320459523404 0.9961999502736418
0.8356979618171110 0.8341582943834425 0.9976559452275993
0.0018896123180738 0.1668979703651559 0.9968015264210521

0.8355051816224943 0.0005288453477945 0.9942684499158787

0.5048816916043037 0.5013018418149713 0.0144356296488510

0.6690705233334100 0.8340606962685797 0.9951662084192847

0.8354485611754825 0.1671183146723507 0.9933099505050293

0.6687075444867716 0.0004315185684405 0.9914121382950967

0.5011058121623648 0.6685937107861122 0.0004451936848824

0.3346053322829334 0.3317349752041423 0.0147168703150271

0.3354896467990898 0.5028534223812358 0.0187616068875229

0.6688242515145788 0.1671162897045519 0.9917314721976282

0.5019799589515017 0.8340944474831247 0.9924560099128001

0.5019885802375867 0.0004662281854664 0.9914089510886370

0.3353812305004240 0.6680732163894589 0.9986583795682762

0.1674169453070675 0.3322166780518856 0.0054275530884382

0.3351764990380275 0.8340547292693259 0.9932746215400954

0.1678619770570080 0.5012536078088061 0.0069868902449244

0.5023047860804226 0.1670717841377609 0.9946904313278822

0.1687808567655154 0.6675355817776989 0.9979010448344070

0.3353996759801490 0.0005413132311958 0.9943794348764712

0.0018616545392334 0.3338970303332900 0.9987384301093130

0.3357145723214856 0.1668784119939843 0.0006236288664141

0.0020015463559560 0.5006895194031316 0.9980541416725544

0.1686130558620818 0.8341002641800728 0.9953570698695415

0.1688997124716920 0.0008736293452358 0.9967532568468940

0.8356077765827692 0.3339139270923965 0.9940046105812215

0.0020858482478232 0.6672118747680145 0.9964677707154692

0.0019503285464992 0.8339331687245775 0.9966322375649312

0.8357896828810425 0.5005973025238859 0.9946709644722631

0.8353271485717428 0.6669928121506459 0.9975492996670852

0.6691536728595437 0.3337307588377598 0.9937240545206620

0.6693466495524376 0.4999072176116286 0.9975896220719065

0.5032032202639343 0.3339099969079200 0.0018523430948392

0.6701263602588057 0.6680042021518346 0.0047291930112721

0.3659371633319068 0.3780386049912750 0.1305334269201310

0.5699979976916509 0.5923586940809926 0.1213619037062514

0.2631094516899211 0.4727727208180283 0.1311882919232090

0.4284174950388504 0.5410769181115356 0.2236186617809111

0.6087908752230859 0.6798283777413019 0.2449508512552967

0.2614627097916866 0.3771057410993564 0.2520442665838019

PT6-XLII - ΔE = 0.81 eV

1.00000000000000

14.7772044699999991 0.0000000000000000 0.0000000000000000

-7.3886022400000000 12.7974344700000007 0.0000000000000000

0.0000000000000000 0.0000000000000000 18.0009597799999987

C Pt

72 6

Direct

0.8872753161732373 0.6064892579779142 0.9932619419894348

0.0532256714193656 0.6065531646240453 0.9931781517212744

0.2199229093013945 0.6072396203649212 0.9975045280442842

0.8872504921039873 0.4404022006770466 0.9928365991310507

0.0542065068722732 0.4402042821394778 0.9939764828270798

0.3851882199105026 0.6060862154831534 0.0122487343954830

0.5551681286272241 0.6069264030410603 0.0150649350211130

0.2197934000076840 0.4394540283932116 0.9974381893968458

0.8868399887969146 0.2733334168842655 0.9926574531913073

0.0535010321178976 0.2735636842095023 0.9932393558632944

0.3852365030040987 0.4391549208260109 0.0025638149787880

0.7214656489675170 0.6073112150228979 -0.0009849699181474

0.5542458821452991 0.4390914948780907 0.0032325297026045

0.2201255273616615 0.2731491746832403 0.9950175745059191

0.8870512787278361 0.1067358479313260 0.9934050426814763

0.7202042880859941 0.4398352743026048 0.9953181929299532

0.3866216157525054 0.2729933402325029 0.9970613605842104

0.0532698194344243 0.1065832569587355 0.9928714416945291

0.8870409320237476 0.9405387007362082 0.9949477986132175

0.5538491964479343 0.2735554769348287 0.9955280974884645

0.2202031998015411 0.1070641115865880 0.9932932038716862

0.0536626532975144 0.9400835528052748 0.9927922047612172

0.7205594686761077 0.2733162418773414 0.9932425451945105

0.3872884106960227 0.1068083918739333 0.9938262032747167

0.2199464214502110 0.9399900185600601 0.9927744260505305

0.8868246601323163 0.7734219119538298 0.9947918916639915

0.5534353462467729 0.1065179161119876 0.9934717934259458

0.3871084347277229 0.9406544000768645 0.9939455250856430

0.0535570733307686 0.7736817544272372 0.9925941025767319

0.7203435530905618 0.1069893389711324 0.9936525991637430

0.5538212457363294 0.9400691457202565 0.9960021545566160

0.2202487967382356 0.7734043054008763 0.9942414367316542

0.7201319897408854 0.9401668140397725 0.9972309154367848

0.3863391418232058 0.7737048708413486 -0.0004746443320550

0.5544293989583572 0.7754576740882198 0.0057453767270577

0.7210208349954436 0.7738219338996496 0.0016530387764448

0.9979333743175842 0.6620725421994522 0.9926646464090333

0.1649541458276225 0.6625898502061551 0.9946606861346819

0.3303173387308762 0.6629783135283001 0.0027771272339763

0.9982619596089950 0.4959739092004667 0.9931812948448406

0.1644431777364951 0.4953525942306857 0.9964157086277348

0.6668829537559318 0.6631087945859583 0.0050019509254318

0.3304941658996753 0.4939067541545602 0.0027504925121865

0.9985508982573519 0.3289403579115262 0.9930960435554419

0.1645590215965108 0.3287476864559663 0.9948755477218613

0.4974577140430438 0.4942630745046017 0.0111039511911038

0.8315237852652226 0.6623912479400713 0.9949364552547066

0.6652955753201998 0.4950919563884325 -0.0006432384537228

0.9979594742634280 0.1619381985997148 0.9928996773717934

0.3308654320272788 0.3285282781856915 0.9977170419956854

0.1649633234452350 0.1621215297133663 0.9933679758109171

0.4985082710909722 0.3285148053103534 0.9985022313824429

0.8321586796933941 0.4958002651367523 0.9934854418534158

0.3315391004104559 0.1627641233783743 0.9944999785664205

0.6648503594784730 0.3289604096287336 0.9946721641292041

0.9980463025626231 0.9957051489622977 0.9932232074862594

0.4978942193278437 0.1618515607029103 0.9939958220879647

0.1645871980299723 0.9953990146437186 0.9926026777266962

0.8314651550697001 0.3287123784429732 0.9928042911759329

0.9982284799709685 0.8289692984572907 0.9930066823145796

0.6651043000816242 0.1622674347683100 0.9932934700700605

0.3317467167430767 0.9957698390733755 0.9932160907656605

0.1645964595026859 0.8291553685953570 0.9928642372367668

0.8314908900304427 0.1624252043292576 0.9931211768687809

0.4979439991075618 0.9958610710015103 0.9940822633449938

0.3312089346086933 0.8286994981726852 0.9953680927038778

0.6647990128730199 0.9955258500150107 0.9955770991974410

0.4987993426338605 0.8299013122057608 0.0000875043674664

0.8318665672196626 0.9957653405064368 0.9950811193832815

0.6650529401344326 0.8296632161617833 0.0018564507803131

0.8314381809028839 0.8288121058916144 0.9968976054424857

0.4983237275647878 0.6641127791762581 0.0166921693497254

0.4972363035615101 0.6693195038659219 0.2639862209568404

0.2993722077608509 0.5741745163850299 0.2353515633103122

0.4304919064116628 0.6355449772364448 0.1274586190680791

0.4019075733380392 0.4741909768045122 0.2346005440421829

0.5486843677260252 0.5485948174810183 0.1253548770497623

0.6025408418412600 0.5745407908429340 0.2605440175178333

PT6-XLIII - ΔE = 0.81 eV

1.00000000000000

14.7772044699999991 0.0000000000000000 0.0000000000000000

-7.3886022400000000 12.7974344700000007 0.0000000000000000

0.0000000000000000 0.0000000000000000 18.0009597799999987

C Pt

72 6

Direct

0.2238513199374452 0.2812968537712521 0.0094942705514924

0.0593438938898743 0.1154269460253801 0.9971293108058106

0.8929569137717692 0.9488434219482116 0.9919411163807155

0.0587919279780564 0.2821454136748685 0.9977149265673049

0.8928685788791171 0.1154965178406187 0.9929939383766424

0.7259557399513739 0.7820237565552262 0.9912438714159961

0.5592686397729523 0.6161164621741904 0.9945262774484434

0.7262724728635632 0.9491833959410556 0.9895362393962230

0.8925325492644660 0.2823244353160348 0.9939387515764224

0.7263942160886501 0.1156661920892148 0.9918742347721121

0.5597655175180023 0.7824160720983642 0.9882024966417902

0.3935183412950352 0.4506419824463492 0.0025462956078215

0.3925294662291776 0.6154514890682918 0.9904578627995804

0.5594523324256926 0.9489611519285788 0.9890801989859455

0.7261320981622887 0.2819924232697417 0.9963865823198006

0.2257759138963564 0.4495963743098699 0.9969291412612051

0.3929007034987464 0.7825395082037616 0.9879864938041862

0.5595750884267900 0.1159123014243590 0.9948200296370828

0.5606406120138558 0.2814663110630917 0.0068327467164195

0.2263552049118971 0.6157561901528581 0.9901720573234059

0.3931367380654649 0.9490880291253276 0.9911047526127490

0.3922618181644282 0.1149373220761163 0.0003115274303553

0.0591064879460674 0.4487179431180977 0.9936545777259553

0.2260560132240386 0.7822175207518924 0.9898286809426509

0.2260681124185098 0.9490716304510869 0.9934270630059530

0.3920753005313315 0.2805215611331704 0.0185516865511062

0.0594798518752455 0.6158019831654471 0.9918054022124423

0.0597136164181507 0.7824340944211698 0.9919125429160189

0.2257705074480967 0.1149031086652954 0.0006860868675815

0.8931335578799349 0.4487625053086433 0.9950192104914350

0.8929511932059100 0.6157369607527556 0.9953897261297726

0.0592903001090050 0.9487514550153406 0.9935838364804468

0.7275356255123810 0.4488249392775572 0.0033298485909299

0.8931659414417368 0.7829363667145870 0.9930624430902881

0.7276321466868083 0.6174016098095905 0.0006195864899468

0.5601910020748306 0.4510901073462534 0.0152468087660935

0.1690604615864970 0.1694269844720466 0.0024180621457717

0.0035203396543224 0.0039449328987402 0.9939585160312774

0.8375463445347648 0.8379144141357244 0.9919245215890911

0.0036454187688477 0.1711261089188199 0.9960678063221238

0.8371457438240597 0.0046189908796563 0.9913097267989244

0.5030656021331268 0.5053067752018450 0.0022539251960865

0.6702062795951349 0.8376119488817864 0.9890503213886319

0.8369118627903944 0.1710213121348687 0.9928502649646305

0.6705415765157312 0.0044358807012728 0.9898517400073530

0.5039420178168257 0.6710339830836958 0.9900021974959614

0.3361502463746149 0.3389431391017936 0.0157612428165876

0.3372791465660541 0.5051866000054801 0.9955492984219418

0.6705809895586512 0.1713070864088593 0.9941941154573257

0.5037209350992526 0.8379293979592575 0.9878162854460726

0.5036114670460705 0.0043707632387537 0.9912250237338327

0.3368364279631493 0.6709963703922668 0.9888423003489422

0.1690391470550807 0.3382115264577692 0.0021489323980433

0.3372578289093795 0.8379274582798715 0.9891102019716271

0.1706810177721252 0.5046399813936944 0.9930117267960483

0.5044677158285253 0.1712111558176304 0.0007775913251251

0.1703682530627049 0.6713741791045038 0.9900933911263294

0.3372543339268645 0.0048479079872905 0.9943632778353333

0.0034581015524964 0.3381559271015036 0.9949132632483355

0.3361276946111431 0.1693098961416908 0.0060148344043398

0.0036562093126449 0.5042892698503678 0.9930928208961021

0.1702786926498732 0.8376688732152004 0.9912777745263028

0.1705166501999003 0.0041221086427825 0.9953436460461234

0.8373868820100157 0.3378168830574353 0.9948932861394653

0.0037252469887363 0.6709615943749796 0.9925690029886738

0.0040510122361113 0.8380764653926818 0.9924646036869120

0.8372544488804010 0.5044487801044184 0.9975733002432321

0.8377064681906887 0.6716174302267675 0.9960028830312169

0.6708465225342621 0.3373819615600908 0.0017032712350868

0.6727200760981447 0.5058654525390267 0.0097289954639308

0.5047836408376511 0.3372305879079249 0.0194043543448714

0.6708763218163085 0.6720368919263606 0.9950345058134076

0.7070621008338946 0.5691291002018986 0.2512862364298130

0.6280428574506516 0.4953087126122142 0.1254065688264987

0.2692103987000962 0.3133509495398599 0.1256204352071109

0.4465973645876389 0.3148029412058122 0.1328098449327086

0.5210886247424185 0.4427842196362093 0.2501122502685718

0.3373699875547231 0.3016536410517574 0.2531815827163015

PT6-XLIV - ΔE = 0.81 eV

1.00000000000000

14.7772044699999991 0.0000000000000000 0.0000000000000000

-7.3886022400000000 12.7974344700000007 0.0000000000000000

0.0000000000000000 0.0000000000000000 18.0009597799999987

C Pt

72 6

Direct

0.2146224505547494 0.2695842615921933 0.9927798814293922

0.0476631328625601 0.1023826936320944 0.9936659587838363

0.8806048764684675 0.9358891470513626 0.9966148614232750

0.0472865649104293 0.2691855035310553 0.9928173853909357

0.8810673092475128 0.1029412183803444 0.9936977739519151

0.7148022153335631 0.7710108471881156 0.0024976463688944

0.5457849461601327 0.6017719834189421 0.9992624875642235

0.7144947974208549 0.9360173242557295 0.9948871493921274

0.8808957993586333 0.2691235504462526 0.9939123138025181

0.7140023908072024 0.1025229403219683 0.9930429875795141

0.5472382628770589 0.7693047195728298 0.9951389454069570

0.3803797099148838 0.4360531600960513 0.9938491778164220

0.3806213007006676 0.6026041325678335 0.9927545985290180

0.5478986662823928 0.9361138267077536 0.9931269865642420

0.7143386664764080 0.2692648443495216 0.9948944652778096

0.2139682729270831 0.4355527290306256 0.9921875606967419

0.3808239789167445 0.7687887101922968 0.9929205403820660

0.5477448808339531 0.1020168765288929 0.9930805731041303

0.5470307738622111 0.2681901957896002 0.9974191536175923

0.2137985195659624 0.6023822362918452 0.9931987809563765

0.3806208369360320 0.9356266485040408 0.9930157358499869

0.3808751618297919 0.1024853178596317 0.9936071104300765

0.0475919677987591 0.4360591276930563 0.9941510614184281

0.2143055509268592 0.7693255661946097 0.9943854380193002

0.2141624572830807 0.9355311918782405 0.9941054624392791

0.3803406378254408 0.2683813457614761 0.9954055217913265

0.0476416938224986 0.6022918325159026 0.9969001262106900

0.0474124226902077 0.7691330181991347 0.9976857087847861

0.2137261784964366 0.1023024924296507 0.9933705828425516

0.8807019805885261 0.4357605553308233 0.9981554416268210

0.8814950977719818 0.6030158524212297 0.0037027240605028

0.0477488648386668 0.9362838593918994 0.9955064309354071

0.7149317738174190 0.4346052458180836 0.0046027690007833

0.8817114387318554 0.7694651892681890 0.0019534293573855

0.7155251487427208 0.6039588118182238 0.0166689890307694

0.5460478795079382 0.4352798262579256 0.0071010616437519

0.1586893443179420 0.1578773384996808 0.9931925924843057

0.9925514756846781 0.9919228395714015 0.9951546258105068

0.8255438519302132 0.8257275168086835 0.0006588378050267

0.9918906537208585 0.1584197754597980 0.9933250565624974

0.8253083749044166 0.9915315389522377 0.9950573398724742

0.4910482177403424 0.4923555411108964 0.9980127687731581

0.6591246148556706 0.8256953948462140 0.9969268046046338

0.8256085609090533 0.1585181415765931 0.9933332333218274

0.6584691717109976 0.9917209584465141 0.9933379186795790

0.4913188338321532 0.6580826782251492 0.9948864092942031

0.3248736463010289 0.3247622511372441 0.9938483115772669

0.3250005061520227 0.4914271882435912 0.9924391391330829

0.6584369482182808 0.1576042922686440 0.9932042227751126

0.4920955956408832 0.8243983344682349 0.9934748099802491

0.4923299824584646 0.9915400107558980 0.9927666846533256

0.3254237246959661 0.6584681898122255 0.9926013023644629

0.1589395369270221 0.3252488046924356 0.9923699512154158

0.3252044077492897 0.8248786758792548 0.9932176057684359

0.1584447818548114 0.4917159120581047 0.9929724940679492

0.4917688685748800 0.1580073614881599 0.9940950281763108

0.1585986707558931 0.6580689198671692 0.9947266290046173

0.3252231144296047 0.9911327150322151 0.9932927975701062

0.9919921734956958 0.3248102229290595 0.9935815357741618

0.3254434522277876 0.1580757819109522 0.9938113581983572

0.9924313154228770 0.4918916594454004 0.9964902494038128

0.1588691432705289 0.8250340218925913 0.9952410885564120

0.1583958326726744 0.9914394072684374 0.9941087467149003

0.8253486591512598 0.3249996924551652 0.9955674622599844

0.9920294146754287 0.6584333494401591 0.9993766590015767

0.9919381197853099 0.8246361250583618 0.9980407184808655

0.8256434280343541 0.4911727769412124 0.0027294397972000

0.8271976957118810 0.6593813898180230 0.0073694871133583

0.6592494063454239 0.3242851853271986 0.9980703291619335

0.6584319946890034 0.4899947776623037 0.0136440026489097

0.4909624128614851 0.3232617346854454 0.9999849150282003

0.6576587558897486 0.6590544251013668 0.0106645477563703

0.6937172824893096 0.6010916936483213 0.2656730290918787

0.5030599332438896 0.5013357915982439 0.2241542445215075

0.5924370704776365 0.4345643028788260 0.1223345717272650

0.6011672966609254 0.4013002431577561 0.2592008805954720

0.6012234707079571 0.7035320948433750 0.2278394179045762

0.6784698661404036 0.6394995623341231 0.1266752004664511

PT6-XLV - ΔE = 0.82 eV

1.00000000000000

14.7772044699999991 0.0000000000000000 0.0000000000000000

-7.3886022400000000 12.7974344700000007 0.0000000000000000

0.0000000000000000 0.0000000000000000 18.0009597799999987

C Pt

72 6

Direct

0.2234566132962144 0.2769759899464503 0.0065990058234036

0.0586702050279015 0.1115446719629674 0.9934634843356207

0.8917109980706144 0.9446405882937157 0.9892895533195585

0.0583673492510286 0.2779536983878756 0.9967887514008638

0.8917245683786632 0.1115454703060053 0.9910134702354725

0.7248016248196265 0.7778908854557258 0.9920037268288908

0.5594804998639518 0.6130976532392580 0.0051469747972206

0.7251909004526098 0.9448650069652444 0.9904410141730864

0.8919306171834407 0.2782285579701522 0.9936731037886588

0.7250375715603496 0.1115208105941008 0.9923602365958146

0.5585810448651642 0.7782173770284260 0.9952653652284269
0.3905155104123637 0.4459961777277925 0.0248367481898768
0.3920997883578821 0.6136194641785622 0.0064183565943807
0.5584390581239873 0.9448314287792101 0.9930072119029560
0.7250380482361862 0.2783020015815154 0.9960478026458119
0.2231576810949250 0.4446643238585253 0.0083243631823606
0.3921128008098691 0.7786626462815818 0.9959091744039554
0.5584781298976225 0.1114705446985340 0.9962142096932567
0.5586456478836368 0.2778227727975491 0.0037859805294431
0.2248943520390796 0.6115696296306581 0.9991740102838946
0.3919636552692722 0.9445695787118424 0.9947102867239934
0.3916767856002821 0.1105161480582595 0.0011592783781467
0.0578306545074483 0.4446554566222716 0.9970518750280633
0.2251031598113116 0.7781581184395847 0.9941576921507291
0.2252645798611681 0.9446243043692846 0.9946442209719635
0.3921943708868270 0.2758434153275431 0.0180310041501599
0.0584904651594300 0.6114495395318258 0.9941146627420991
0.0584025661565803 0.7779168767332933 0.9917067907594088
0.2248447708695451 0.1105556547652995 0.0002387139131628
0.8917908588507757 0.4445839671903897 0.9942355100686697
0.8916903825266331 0.6113298561188500 0.9929814393345779
0.0582090499381636 0.9446798800562064 0.9913669427034719
0.7258299478046979 0.4449547075120535 0.9990389661997483
0.8917623740934175 0.7781197432323452 0.9902373416849528
0.7254356020584680 0.6112562799179244 0.9971918495152252
0.5606393535651009 0.4448673175902300 0.0174858374279836
0.1692971659496578 0.1664969609581988 0.9992810645783763
0.0031641312560993 0.9998398363767009 0.9906739691552175
0.8364457781746282 0.8332950585393704 0.9897640219209549
0.0032466115766709 0.1668626991860407 0.9932421827032201
0.8363573172534160 0.0000240014877733 0.9896875497637438
0.5054652855111286 0.5025202172189225 0.0210347281446985
0.6697485998023538 0.8333007710118849 0.9920619431736171
0.8365565659390626 0.1667615963749896 0.9920999359381852
0.6698133033185485 0.0001566803207140 0.9916596398389075
0.5035882727864518 0.6676502161024800 0.0018940263940337
0.3342385518754014 0.3309969026015054 0.0219307320112279
0.3349913825969182 0.5021200546361158 0.0159385645015035
0.6696942367561576 0.1667460437221351 0.9948444739435303
0.5029844843793185 0.8336241854583193 0.9944483482340090
0.5030294818911472 0.9998396889478087 0.9944757756457747
0.3365264745122545 0.6673485637451009 0.0001605947728223
0.1690760623075889 0.3329107415291190 0.0036474213772451
0.3364630682064274 0.8334732254933215 0.9944939996019428
0.1690759446336827 0.5002111119031127 0.0013782938631977
0.5030086821678097 0.1660993363220200 0.0004499876807884
0.1696913024848286 0.6666346738090212 0.9953658529498952
0.3361342330772956 0.9994974352677346 0.9963673015776493
0.0028263652053653 0.3334809815789939 0.9957202929296471
0.3357622337194002 0.1645352286325448 0.0066747973131029
0.0030509462509016 0.5000600326813611 0.9946898908467361
0.1697696651611054 0.8331446382009631 0.9929935903406459
0.1698517060125937 0.0000460168962704 0.9948446086175977
0.8366772899091046 0.3337131798140049 0.9944477353047674
0.0032109258552779 0.6667786587534792 0.9924080665172568
0.0029230255069308 0.8332648569602981 0.9904294039142698
0.8366342121501233 0.5002428119033340 0.9947466961116689
0.8362777718516909 0.6664182988873648 0.9925118983817640
0.6704268091215582 0.3334802544664157 0.9997643641979295
0.6715666767842734 0.5007809316392695 0.0036207660422960
0.5043888145310049 0.3320602517110203 0.0126199971197849
0.6695645429440091 0.6668891851575296 0.9972292820128033
0.5375896135888638 0.4782574003250417 0.1347580475807817
0.5245703652569631 0.4945845292143858 0.2694886306418738
0.3484089795888536 0.4712847540267830 0.1328698859793889
0.3584583363057234 0.2967721591789640 0.1348969116011745
0.6910570612438676 0.6175086811231623 0.2066184917324705
0.3318701048989752 0.3733900986788186 0.2562208996265127

PT6-XLVI - ΔE = 0.82 eV

1.00000000000000
14.7772044699999991 0.0000000000000000 0.0000000000000000
-7.3886022400000000 12.7974344700000007 0.0000000000000000
0.0000000000000000 0.0000000000000000 18.0009597799999987
C Pt
72 6
Direct
0.2231088855536143 0.2751032916546734 0.9983051268798917
0.0567200306398519 0.1091510086487872 0.9901627684908831
0.8901825636086613 0.9426321587242867 0.9896170760524114
0.0569286524654657 0.2758121411894265 0.9942480192820398
0.8901702426907363 0.1092026962380004 0.9905057849861265
0.7242230322060266 0.7762128604871634 0.9970604176536071
0.5594364149086886 0.6111633488992965 0.0178682671882839
0.7235467648996234 0.9424467651648882 0.9936840362420796
0.8901428156492202 0.2758967916266357 0.9922305590587115
0.7235146675871036 0.1092657893458551 0.9921938244749100
0.5576030925993933 0.7776532074773783 0.0051091356779409
0.3883246882756808 0.4400476560526840 0.0185880750214764
0.3890704574573505 0.6102632973530646 0.0093777272075002
0.5570536820906327 0.9429084687012548 0.9953771690026301
0.7233202422059577 0.2760837812969541 0.9930767609769262
0.2217450625536159 0.4417870832785127 0.0056798512077734
0.3903613825077983 0.7763763829457915 0.9986785771394793
0.5569436196169093 0.1091494600003955 0.9927534581994095
0.5565863534595010 0.2752496504375443 0.9963050261525694
0.2229974364090808 0.6090062049875939 0.9987888780224665
0.3903253706391467 0.9425229225930281 0.9930498267480274
0.3901545005697642 0.1088648502091232 0.9926760259339176
0.0564965562011182 0.4423296374215795 0.9954785322269331
0.2235723192476868 0.7758211091289695 0.9929773632641385
0.2235890122428614 0.9424345485041954 0.9902295749258556
0.3897995556570351 0.2749152579866134 0.9998075049954167
0.0568944603974728 0.6090834718911040 0.9927583450595989
0.0569296961605303 0.7757763374214264 0.9898363499025322
0.2234398978143375 0.1089346951022563 0.9915546358809237

0.8902810114796296 0.4424706048349236 0.9924180019519540
0.8905126198988356 0.6092207973138954 0.9918524272406302
0.0568116976397377 0.9425189691412328 0.9885723910616093
0.7241397090400028 0.4428106115366148 0.9958308375634459
0.8903979696034341 0.7759060244641702 0.9905560716694168
0.7244191796935979 0.6095467075488088 0.9986371754530765
0.5583571879614126 0.4410951529269553 0.0106065434992928
0.1680744985425306 0.1644643221204021 0.9924438629960193
0.0015038535400222 0.9978246477156247 0.9887146901467219
0.8348753078056816 0.8312764992685331 0.9913907248404570
0.0014060154801783 0.1643793264647542 0.9910946851863400
0.8349820237318113 0.9979766401488064 0.9906881104332470
0.5028571081412423 0.4966588123483930 0.0191481564831477
0.6686803865875461 0.8320151820333237 0.9985507926461139
0.8348316385655750 0.1645735680504288 0.9914753911911731
0.6680295368131581 0.9980361491282821 0.9937689417768354
0.5014278800292615 0.6664695412819341 0.0153571485223623
0.3335268037007637 0.3293222001841443 0.0049604485819685
0.3329451321392867 0.4979944041164970 0.0157972064347405
0.6679900169438611 0.1645722861122536 0.9926408297247491
0.5014594254043843 0.8319598809015289 0.9998545212320806
0.5013760180018210 0.9979161878986673 0.9934339217875561
0.3346750858598213 0.6646728231312338 0.0016984165411102
0.1673372857115893 0.3306752796872274 0.9994254992526308
0.3348761301326064 0.8312278602560426 0.9945842693131013
0.1674455468514040 0.4979081125428948 0.0001127421071985
0.5011607506168119 0.1641126990474078 0.9935411519067472
0.1681836404801516 0.6645277440995443 0.9945224151972099
0.3347197212989244 0.9977588279445229 0.9915374353181861
0.0013716594318183 0.3313666691565231 0.9939863042629113
0.3345790595820475 0.1640998657189456 0.9939084679565724
0.0015114699087988 0.4980297521297672 0.9932179570276602
0.1683015097637437 0.8310647641755082 0.9905348569148700
0.1680876411793051 0.9977848740290156 0.9895086161557103
0.8348522002541827 0.3314309180309607 0.9925203219526679
0.0016381830167091 0.6646664235970121 0.9909757353327180
0.0015653237320805 0.8312589260540904 0.9889928701743074
0.8352889399450731 0.4982341841285987 0.9929232124485381
0.8352474914337762 0.6647695855219453 0.9927866100673484
0.6682582479495096 0.3311245412721036 0.9952917036794986
0.6701419803056581 0.4984885859008017 0.0011641126562907
0.5009045361948523 0.3292429285215377 0.0018535810867562
0.6700281461069295 0.6658254200069749 0.0037061846697242
0.6229657043140406 0.3925347070899576 0.2152588322680273
0.5323855376907360 0.6436409872710342 0.1322210724032544
0.5378956949720362 0.4637697456327885 0.1303433273009205
0.3579598436554079 0.4685528411151694 0.1330552488814760
0.4294098909713000 0.5734577369508713 0.2553135173372993
0.5304525529034834 0.4799499736653416 0.2665581341188457

PT6-XLVII - ΔE = 0.82 eV
1.00000000000000
14.7772044699999991 0.0000000000000000 0.0000000000000000
-7.3886022400000000 12.7974344700000007 0.0000000000000000
0.0000000000000000 0.0000000000000000 18.0009597799999987
C Pt
72 6
Direct
0.2159821318641235 0.2755680255567157 0.9996910259499998
0.0505216286703032 0.1098377283569718 0.9956280508528792
0.8842638885882437 0.9435531928681158 0.9948525942828326
0.0508018081185710 0.2765509049961494 0.9952156957152809
0.8840608267857561 0.1100836413577895 0.9976085873331115
0.7182855152400123 0.7776515635165708 0.9972187529332857
0.5528852293710500 0.6098562108506158 0.0096505388260013
0.7176801585035477 0.9433369690049105 0.0016711383030739
0.8842773773492709 0.2769152124380483 0.9988158875916824
0.7176267870075392 0.1097413618649341 0.0021622900905527
0.5515210345453525 0.7772672029843335 0.0104896595305561
0.3842948548872442 0.4430237240518764 0.0044856465954339
0.3831103460270811 0.6098196043662298 0.0055450592286377
0.5510680614005139 0.9433160051335108 0.0056596244721803
0.7173007023863107 0.2763312839168819 0.0027014572909749
0.2172620220696757 0.4435434630240209 0.9961550708158811
0.3843686578814101 0.7767722910212598 0.0054739066934104
0.5510995871796993 0.1097217474188881 0.0030454026863680
0.5506791233143815 0.2758372820854333 0.0002549024938148
0.2174536291989355 0.6098940831784390 0.9986306527169049
0.3843996245605368 0.9430765910220984 0.0023900427322090
0.3842869838459890 0.1094758417058941 0.9999439348910357
0.0507667844758259 0.4433444409125187 0.9959607586434416
0.2176716753014176 0.7766748620033610 0.9988277189226906
0.2178023649507779 0.9433124756963309 0.9976203498726406
0.3847540828529077 0.2754275622497246 0.0006884550228889
0.0512007619658874 0.6101241388554399 0.9961252055637573
0.0511682054093967 0.7767467208786272 0.9939081374265015
0.2174457355831123 0.1095838505281606 0.9981611945096986
0.8846327044474762 0.4433871771447713 0.9995060279244115
0.8847797772795403 0.6102645443255525 0.9946158188230925
0.0508812668564786 0.9434084170285573 0.9942557663830840
0.7182153163913725 0.4430231503948576 0.0072440958064064
0.8846329910374351 0.7771275644074152 0.9913901160270484
0.7188177352728360 0.6121596248191707 0.9940873523675364
0.5496714009968002 0.4425166907767490 0.9953527565751017
0.1617089345949054 0.1650679155419326 0.9975511731122211
0.9955356509243174 0.9987459018590243 0.9944676002590427
0.8290876405511725 0.8326680624630214 0.9934092003767319
0.9953993063939208 0.1653764836306451 0.9959112992319845
0.8288361064989047 0.9989066239057109 0.9977972957992165
0.4968163284463643 0.4978725336667580 0.9976511544530524
0.6626525435708714 0.8326131990159880 0.0034114983652671
0.8289197621926121 0.1654675287585690 0.9995986195294151
0.6623502759767064 0.9989435329824587 0.0038095879499309
0.4955331633230031 0.6656178171245273 0.0151047918196241
0.3281193151637893 0.3306963629090731 0.0056883147017501
0.3275863266912893 0.4995235922156454 0.0000622033451165

0.6620169117368988 0.1651895821405773 0.0027419088827259
0.4957109415440328 0.8322889041978314 0.0081053906156967
0.4956456291013396 0.9986250453778709 0.0040045417431074
0.3287199760247361 0.6655536062301053 0.0031709289595767
0.1615293852813551 0.3316230277684795 0.9957251681105319
0.3291173687892055 0.8320523338261694 0.0023592188477508
0.1621876622713643 0.4991365481093339 0.9962101816652051
0.4956227502766524 0.1651637493935212 0.0012377174936162
0.1622125139433308 0.6656490399980797 0.9976900997245792
0.3288997901226125 0.9984654715995518 0.9999323480169053
0.9953858873801167 0.3323921493690065 0.9961926516008930
0.3289939438404303 0.1647409367554005 0.9996734327977919
0.9959672959428190 0.4990806487660464 0.9968192468318833
0.1624988792093873 0.8321719950345354 0.9966079362619382
0.1624799787721827 0.9989367760997183 0.9964944974828853
0.8291568440206376 0.3324257796397987 0.0008998326813767
0.9959337897254272 0.6657953198266924 0.9943330013259768
0.9958471754777278 0.8325042512658669 0.9926901440919167
0.8301777288410364 0.4995995952595962 0.0005307608113796
0.8296484827748998 0.6664265316221005 0.9915626166800848
0.6619566737345650 0.3309770849801126 0.0037773829789742
0.6622321684516947 0.4999024269909427 0.0044282172324799
0.4950262135872947 0.3310737527938272 0.9971793560349766
0.6641940406012523 0.6670718653569310 0.9979002603610283
0.3578226013562329 0.4085767367200361 0.1201177854826625
0.4013933832142342 0.5016043645983999 0.2443249138962642
0.5322013676511190 0.4562630726718808 0.1909592917526339
0.6996673877348556 0.5023045478270447 0.1193854386774973
0.6904391688379476 0.6356827073205267 0.2030752873781978
0.5170754618932420 0.6066089745666048 0.1270861184710625

PT6-XLLVIII - ΔE = 0.83 eV

1.00000000000000
14.7772044699999991 0.0000000000000000 0.0000000000000000
-7.3886022400000000 12.7974344700000007 0.0000000000000000
0.0000000000000000 0.0000000000000000 18.0009597799999987
C Pt
72 6
Direct
0.2222043010144148 0.2754448819648871 0.9952567728101229
0.0555363886301663 0.1093964911969749 0.9958052857739119
0.8895220320843720 0.9431698751498985 0.9996507225853364
0.0553458536170268 0.2755922776415858 0.0006899797154958
0.8888922797287719 0.1090302528829739 0.9975255103697478
0.7235511616027495 0.7771802275687634 0.0085478877164320
0.5561042472368456 0.6077035616151605 0.0177531509454809
0.7224516551458180 0.9429185649083180 0.9988213393093659
0.8891020047680982 0.2760620404594718 0.9976124769683423
0.7226886881946299 0.1093814679955827 0.9940487519670143
0.5567321526251305 0.7779748373226312 0.0056700457928542
0.3902606808618359 0.4421993812083471 0.9993223259261796
0.3876335495626364 0.6101981672283046 0.0169406695635530
0.5556083332472284 0.9424015916137378 0.9939556827692257
0.7224893247474071 0.2756957420880823 0.9927421367439990
0.2205131519241519 0.4416321502934721 0.0117165432630912
0.3883513626437960 0.7763226020860898 0.9992239481838610
0.5557186276119452 0.1093733822077994 0.9894803229924776
0.5562020987938752 0.2762800761649515 0.9891467009777912
0.2216760492267511 0.6097137371064605 0.0029806334468816
0.3893430448354991 0.9430234504405277 0.9906507551630170
0.3891578620368250 0.1093795061112885 0.9876259176012638
0.0554887084970943 0.4426024575038028 0.0024476338244739
0.2221171634716086 0.7759226960105963 0.9950960897158865
0.2223553367490894 0.9428555482730800 0.9909882179268124
0.3891440392102297 0.2763633213137950 0.9885185754589969
0.0552968090438668 0.6093237179369169 0.9984016606601926
0.0559557495246779 0.7761759381895956 0.9960347582000182
0.2228068171858482 0.1097086906548910 0.9899483129610758
0.8892165736073352 0.4427146322012163 0.9973629400692090
0.8892370626152797 0.6089407530091364 0.9985985466382914
0.0557903821619377 0.9426159783806014 0.9951185167150101
0.7223483399455901 0.4425294029661160 0.9954890450498368
0.8892731958485740 0.7760875556237892 0.0012616391748139
0.7238332192026462 0.6087251950410746 0.0055205587297635
0.5558387352224585 0.4422203183223914 0.9955758065232274
0.1670114047683313 0.1653486665830144 0.9929199886021678
0.9999449475629305 0.9983148306754117 0.9966440221628758
0.8336175314166496 0.8316214495885177 0.0033515292899651
0.9998895802544183 0.1643434215379287 0.9977908000418694
0.8336865813116248 0.9984015468347565 0.9987181668433607
0.4994984663720317 0.4967515616587477 0.0016837574453135
0.6669732504082475 0.8319986074853816 0.0039397903595244
0.8331324011102765 0.1645903736291032 0.9964071754878816
0.6668791011109576 0.9979992274417242 0.9951839900852661
0.5000524773098931 0.6665338103050757 0.0181149787391703
0.3338277187559697 0.3314172919326452 0.9921458136542043
0.3329445778400881 0.4964111181301973 0.0146605810935370
0.6668212617041789 0.1649131420618986 0.9916747044612180
0.5004321159523641 0.8323104553520650 0.9987824732721791
0.4997005718662564 0.9981271671734149 0.9906546589121703
0.3317530232812018 0.6657495927437083 0.0055905719498099
0.1655126764059247 0.3299983382720981 0.0033396865250310
0.3332183583170050 0.8314900605114062 0.9942422005572524
0.1654630827369346 0.4982986230702409 0.0066489380594632
0.5000676536606363 0.1647599279642549 0.9879934448203826
0.1664442008971534 0.6651812072385823 0.9983929296214953
0.3333809560436691 0.9983842758024224 0.9891277427486358
0.9998533897396911 0.3313529129741752 0.0008657572519581
0.3331259907499984 0.1648502989054208 0.9878722750524318
0.9995830892581736 0.4981828006435478 0.9994675500286903
0.1663419475431525 0.8314846914645031 0.9936434941928951
0.1666748926267587 0.9981358339073978 0.9915259802424785
0.8333556002365512 0.3312638299084583 0.9960020697351197
0.0002047873956741 0.6648264913964965 0.9975294392291190
0.0000358627723500 0.8317673396980041 0.9972317467899714
0.8336889073709273 0.4983670802855258 0.9972257469573478

0.8338312046560361 0.6647851310583803 0.0017205940414655
0.6665343404202844 0.3313685656529017 0.9918799953980511
0.6671702843747056 0.4976740524463708 0.9988769935637762
0.5004324798926660 0.3319220190240628 0.9898202092187489
0.6685602454253257 0.6650414127604449 0.0143655479457436
0.5500446221219377 0.6782950499235199 0.2521523446524228
0.4477840316678439 0.4910384109674197 0.2097797077350307
0.4440699003468893 0.6509055319584647 0.1304417044287476
0.6136289279257312 0.6366311416135275 0.1310400554573334
0.2822489970807069 0.3280116909786415 0.2277816556550718
0.2856910135404860 0.4456320926932946 0.1256190745791201

PT6-XLIX - ΔE = 0.84 eV
1.00000000000000
14.7772044699999991 0.0000000000000000 0.0000000000000000
-7.3886022400000000 12.7974344700000007 0.0000000000000000
0.0000000000000000 0.0000000000000000 18.0009597799999987
C Pt
72 6
Direct
0.2192250805899008 0.2754976003621792 0.9970739814366354
0.0530037149842713 0.1093822920467886 0.9943922140964432
0.8870756563450328 0.9434718800636190 0.9961030830047051
0.0535645836827783 0.2763917780961265 0.9983792499445912
0.8867948908708883 0.1094314259059743 0.9982600427064767
0.7215293201885231 0.7777911313879216 0.0024078802535072
0.5540135391642593 0.6111133672042328 0.0117689723235515
0.7197612481371962 0.9426759368025515 0.0000257993380686
0.8868543854108069 0.2759663944611077 0.9986835610393143
0.7199623621562665 0.1097160730428328 0.0005543215259536
0.5530731834944120 0.7767169272823722 0.0022619553419148
0.3851102502212029 0.4415811004521188 0.0110629885044062
0.3869074801201293 0.6101521302473500 0.0049659435381955
0.5535604652792117 0.9429843599166006 0.0000721544777207
0.7208484119024803 0.2764558984805134 0.0003239739093175
0.2199528832433941 0.4429368758870567 0.0029402351776895
0.3864784698858870 0.7762153346682794 0.9993730948651702
0.5532550190600958 0.1091115104748255 0.0021127204775837
0.5543002355748570 0.2761089451911616 0.0073565076763487
0.2199637317768364 0.6097476682980343 0.9992162818035197
0.3866820095183883 0.9428818498705809 0.9972875035907052
0.3867187670436323 0.1089221305253858 0.9968307852792222
0.0536731418458771 0.4428397029288789 0.9981730130694544
0.2201109235148735 0.7761034021772915 0.9944678170191565
0.2196292569319880 0.9425855163975285 0.9920372150732959
0.3845594610715324 0.2746555870956797 0.9965140605153593
0.0531776941037307 0.6091619129857975 0.9934013352197013
0.0532599623198706 0.7762181766050134 0.9902696273212754
0.2196701401341059 0.1094009732972125 0.9920924865138545
0.8869376956120902 0.4428265327837906 0.9946417344526353
0.8871593894466727 0.6092393506125902 0.9889975790155887
0.0534257697141740 0.9428862206258444 0.9916641087842351
0.7203811667833406 0.4422480272629770 0.9921722823739003
0.8867160762965725 0.7763868266634546 0.9919940620355732
0.7213955352760522 0.6093717734953685 0.9922714969243529
0.5552790752172285 0.4438558895521680 0.9955194122863418
0.1640857547397800 0.1646866076311539 0.9936229901142113
0.9977321864207411 0.9985321619329923 0.9935846410513633
0.8317186116804649 0.8320873464529441 0.9966029275045045
0.9975971842797549 0.1648256735592000 0.9968950155401402
0.8314063680980794 0.9985176921947456 0.9983698924325566
0.4982872529833529 0.4978250827928790 0.0103209419932497
0.6645650689868674 0.8325988731282976 0.0018021120322942
0.8310404249718317 0.1651615854547757 0.9994759814860359
0.6639499888409048 0.9982321006788908 0.0003705012427844
0.4970205651134449 0.6660318228151851 0.0058199898538973
0.3293579783585798 0.3298117369316316 0.9997723787388679
0.3302833039338964 0.4984306415627344 0.0076441474820470
0.6644408345040560 0.1648578007492532 0.0017460470334925
0.4975503441064006 0.8317858064145724 0.0002651602426056
0.4973370789969564 0.9981562664147958 0.9998544981267941
0.3309337251648756 0.6652988863916249 0.0009717513981258
0.1640143675707080 0.3315889377162264 0.9996793426812758
0.3306316787958821 0.8314782587275005 0.9968698686425412
0.1641679244212071 0.4983544397117328 0.0002170999311559
0.4969203615613580 0.1636030265903585 0.0031843259370277
0.1645306297311393 0.6650278632400557 0.9955323351105747
0.3310241872206205 0.9983104497171738 0.9948729390119553
0.9979313956385738 0.3317568206504689 0.9986423399979429
0.3302489858900017 0.1646779670998271 0.9937501266176341
0.9978308962119513 0.4985428796712341 0.9952880664438339
0.1640021425394664 0.8316631993245167 0.9918319174446779
0.1638933302880119 0.9978831012151161 0.9913882231216178
0.8312547451631573 0.3316584216402987 0.9978108286895377
0.9973844938006167 0.6645307353112102 0.9903663424655065
0.9975448445104718 0.8314541738327179 0.9906949900147382
0.8313445648069973 0.4980403455257516 0.9910332396097701
0.8309317730184063 0.6648141601395992 0.9897293856271574
0.6659351281780914 0.3323857186910431 0.0003659326774113
0.6642352415792132 0.4977845747135845 0.9901468448377742
0.4979249354371760 0.3319000947442845 0.0036844976555628
0.6663786837635897 0.6656110571894516 0.0047351977088326
0.6359161167001801 0.6441077435909150 0.1205626686630481
0.4344957832447562 0.3390717590056838 0.2410201615655865
0.4516023206942350 0.4533416150129739 0.1235775696749570
0.6143109527818922 0.4788513294470107 0.2041620016141863
0.7527519436624601 0.6574432338423435 0.2259912033981664
0.5052108358087182 0.3077772617644499 0.1208410846808903

PT6-L - ΔE = 0.84 eV
1.00000000000000
14.7772044699999991 0.0000000000000000 0.0000000000000000
-7.3886022400000000 12.7974344700000007 0.0000000000000000
0.0000000000000000 0.0000000000000000 18.0009597799999987
C Pt
72 6

Direct
0.2232155472071966 0.2875124066285295 0.0085416891928531
0.0574681376657153 0.1216957681103281 0.0027392453573825
0.8909373509346850 0.9552398819593861 0.9978234947603255
0.0573210177481229 0.2880677751461960 0.0067421264264524
0.8906476652093787 0.1214221723504068 0.0022014229245713
0.7241766603817865 0.7885528187919988 0.9931769371718246
0.5574263535195314 0.6228164744622191 0.9921223941105239
0.7240485646550781 0.9552281954466579 0.9951249736181822
0.8904082023934947 0.2878437985823155 0.0072189150558870
0.7240275651906032 0.1216568582901232 0.0000975750119849
0.5574524306629201 0.7886578739249401 0.9923042388569101
0.3907871424435996 0.4562764404111590 0.0036450245335615
0.3902804431879048 0.6217480113881848 0.9975322669131828
0.5572412828233055 0.9547194059630613 0.9939724922200384
0.7238448812398133 0.2868655896812982 0.0096492301147251
0.2231159812939580 0.4549236739802183 0.0050949255211776
0.3903190244706565 0.7882200801329020 0.9951788147864207
0.5568460748891937 0.1209832878859913 0.9959652280768125
0.5553890043434535 0.2865658918220717 0.0060158051357035
0.2237014709483773 0.6214861320131746 0.0015067943129310
0.3904408758568536 0.9545607941110674 0.9934628826313769
0.3902076517623314 0.1205494313779454 0.9920361348266837
0.0566735670129290 0.4542963027715174 0.0071094093795097
0.2235549141434134 0.7878884907156234 0.9981044760598508
0.2236974947440373 0.9546445139366142 0.9955184649322675
0.3911497991860813 0.2873893252634758 0.9947222735694510
0.0568548079173965 0.6214046524403614 0.0050171039933247
0.0571923999746318 0.7882748359672505 0.0005833922881706
0.2243018506414316 0.1214619091685591 0.9980249027316717
0.8905489134890630 0.4543945193098153 0.0106667716781885
0.8904172237840768 0.6217601859989443 0.0064893751989672
0.0573257302072534 0.9549787360709274 0.9988246222455928
0.7249308839921866 0.4553187876362230 0.0134651680150597
0.8908060121349095 0.7888936069798476 0.9992573740391251
0.7247578945702813 0.6233875965624094 0.0023406848169358
0.5563276288229275 0.4548938216253617 0.0027298681903574
0.1679974630394341 0.1768678747951142 0.0035262250532853
0.0014708342061454 0.0102757148239476 0.9996565026523072
0.8352293014574599 0.8440254944977212 0.9963943566543136
0.0014614464539306 0.1768685406771127 0.0046030379718047
0.8349306338324425 0.0106041658924809 0.9981263489098495
0.5015653396050865 0.5124006610739542 0.9959960704553339
0.6679571353964846 0.8439034295085150 0.9924295023517118
0.8346444129956083 0.1768076787564610 0.0035761348692560
0.6680653844704523 0.0102355446034821 0.9958345298291533
0.5012388749967229 0.6773633313061751 0.9928927956888600
0.3351968956784930 0.3430818951224808 0.0083445087679834
0.3344166346065407 0.5111779575562210 0.0025963185327811
0.6682592022893346 0.1768348995422926 0.0015328927148914
0.5012059568632026 0.8437992937320828 0.9934309475661536
0.5011015248550663 0.0099881549000145 0.9937070507221861
0.3341762194382056 0.6768894499608109 0.9979146125030667
0.1675898500376221 0.3435592325917867 0.0076116592196200
0.3343330058372089 0.8432261417592457 0.9953195524549159
0.1678571400836972 0.5100360924731504 0.0045329324616148
0.5005845265787059 0.1748950033526739 0.9952808367880834
0.1675890387566170 0.6766643827286245 0.0015960228542795
0.3344060480915942 0.0096422373780953 0.9929409234221254
0.0013927631415598 0.3437478404526573 0.0073225550249205
0.3344668657616268 0.1760489639793335 0.9940837220179546
0.0007483522571121 0.5098289092233088 0.0080202086209553
0.1677119169214834 0.8433208587637893 0.9980038802828304
0.1683799230412646 0.0104122299159357 0.9973305399803678
0.8347372846831718 0.3431257050718912 0.0100916581014019
0.0011346105395091 0.6768586259013816 0.0041726191052973
0.0017700384348629 0.8440596844470605 0.9995230242853239
0.8344700711822810 0.5104091946880089 0.0108595723800136
0.8349730858426838 0.6775950473452355 0.0029349346735756
0.6680474267147218 0.3427421501649519 0.0155528400418987
0.6685516019891296 0.5116898119197515 0.0108382435375631
0.4999249398801382 0.3423217026942353 0.9940482776019195
0.6689370128454044 0.6783170246819310 0.9936775577121040
0.5816378966362095 0.4890012304960507 0.1193248283761577
0.4741437409026901 0.1397540823680288 0.1965764015279490
0.5756826391700329 0.3089142736257671 0.1246519145228504
0.4858277497242298 0.4684763407560482 0.2422952794248161
0.3744505582244173 0.3881363183078577 0.1246145374460994
0.4307983486416305 0.2803260475852909 0.2099298419824649

Table S2. Coordinates (VASP CONTCAR format) for the structures directly related to lowest energy structures proposed by da Silva and co-workers (reference 22 in the main text) and Nakajima and coworkers (reference 20 in the main text).

Pt6-min da Silva
1.00000000000000
14.7772044699999991 0.0000000000000000 0.0000000000000000
-7.3886022400000000 12.7974344700000007 0.0000000000000000
0.0000000000000000 0.0000000000000000 18.0009597799999987
C Pt
72 6
Direct
0.5492415407890491 0.9429766179140443 0.9965190778859627
0.5489331170683799 0.7763099709616477 -0.0008938378991668
0.5497473531138891 0.6113464820536064 0.0142519311289370
0.7157846515536391 0.9423180305393083 0.9974079118183010
0.7162150454330778 0.7761396117834819 0.0000752615912693
0.5503977143867259 0.4427958387076411 0.0152867789016511
0.5495708131138792 0.2747180284870356 0.0041555471921389
0.7162859704739390 0.6098087412317503 0.0006158114005855

0.8824893775233916 0.9426226264104238 0.9950016121344460
0.8827573510017731 0.7762991640547111 0.9947410439598067
0.7163007497326644 0.4431209445354466 0.9980037186729681
0.5494403414521923 0.1091174121480980 0.9979098882359142
0.7158577777658922 0.2761720244909295 0.9960747334775801
0.8825239360446855 0.6095500119828675 0.9930059432697742
0.0491166192722048 0.9428661912529042 0.9935535452392080
0.7157187335089429 0.1090686388171219 0.9957009794212061
0.8824042301714631 0.4424487902934676 0.9920816966913082
0.0489814315509799 0.7759558950242528 0.9923085480111601
0.2158033182199056 0.9426946284634583 0.9947886520808670
0.8825725001522678 0.2759556210833046 0.9926723744726861
0.0491653376061229 0.6092837163855404 0.9908009917902149
0.2157735768153758 0.7761409315329311 0.9926653429204849
0.8823713279295352 0.1092695971788220 0.9940518782848199
0.0492347950301497 0.4427060527022404 0.9906353912808623
0.2157041570874141 0.6092036785814552 0.9913839772872520
0.3824449264773633 0.9425402607427236 0.9960378467537977
0.0491667186113673 0.2758503019211107 0.9920904521186897
0.2160763694283427 0.4427286185786112 0.9928630160230340
0.3825701216258995 0.7761668049221238 0.9946482387474359
0.0491929307788261 0.1093990098797678 0.9934811448033062
0.2157043526058625 0.2760111322876707 0.9951894983481084
0.3825627784527694 0.6099144591116871 0.9965657808293242
0.2153756462052089 0.1090378053798962 0.9960276798329463
0.3810168045740849 0.4420487582403135 0.0033280839054217
0.3809797380686911 0.2747942793253666 0.0042894632049268
0.3824177202146957 0.1091253613466352 0.9998143216636337
0.4935354586248204 0.8322030823071640 0.9964045757483346
0.4931787379180958 0.6665893048410727 0.0018665299517279
0.4923032960745290 0.4990542995800675 0.0163670115855979
0.6608850631836678 0.8324251325196733 -0.0002890723838065
0.6621339533043277 0.6666720260327158 0.0048983133193186
0.4939430194406967 0.1649322092063497 0.0007647144078021
0.6618888264236307 0.4994189931141458 0.0031752539878215
0.8276658963296197 0.8323414638593983 0.9963030303687290
0.8275149501317041 0.6656080622868716 0.9953113605414003
0.6609693761675879 0.3316815477395102 0.9994911534530830
0.4939358166779751 0.9985677596194115 0.9967267952700769
0.6603937887644094 0.1650921751115221 0.9966121020524662
0.9938948237147107 0.8320591788030418 0.9932246564775811
0.8270950010554452 0.4986895142739384 0.9936171951315493
0.9938438955320602 0.6655426111741048 0.9916119363125572
0.8274777545389216 0.3322745765449966 0.9932634933612076
0.6604629098517124 0.9987873413164852 0.9967060050041443
0.9940794818177102 0.4988582324607115 0.9907156655932624
0.8274091902371109 0.1653943265582918 0.9941589977282880
0.1607758906125628 0.8322596623054009 0.9929790850307608
0.9939669732616810 0.3321187134888700 0.9914748887866510
0.1605244400645584 0.6654275669135037 0.9912183597856452
0.8272573286491735 0.9986699529936535 0.9956391653290815
0.3271873757230247 0.8322618014250914 0.9942610861753503
0.9940292694121114 0.1654733580118458 0.9929815894312403
0.1607317743446286 0.4989652993850829 0.9910182236642442
0.3274503973912538 0.6654479421172915 0.9933672268109076
0.9941092222005961 0.9987163352385753 0.9937668397328644
0.1607123733284238 0.3319866955125276 0.9929993656600753
0.3268768143093384 0.4983585182460563 0.9968752596790826
0.1603703132332335 0.1651790364700066 0.9946582228942571
0.3268905700603869 0.3318830632773009 0.0002470883860341
0.1605659873734986 0.9988114681303779 0.9945422265838021
0.3270596940143608 0.1653714252047087 0.9999602252720654
0.3272489625850869 0.9989486744583580 0.9968043700496654
0.4930434636482736 0.3300889185518351 0.0127490986760200
0.5308740891280639 0.7278847100264478 0.1747405789429834
0.5052174716622926 0.4833160889832939 0.3692044442300765
0.5313434598633791 0.3879028418225027 0.1278131429809398
0.5258914308354793 0.6085903924834350 0.2699916476719872
0.4569055672265362 0.4119307010142212 0.2425628780836020
0.5204573272809885 0.5595853482013816 0.1304824357570616

Pt6-min' da Silva
1.00000000000000
14.7772044699999991 0.0000000000000000 0.0000000000000000
-7.3886022400000000 12.7974344700000007 0.0000000000000000
0.0000000000000000 0.0000000000000000 18.0009597799999987
C Pt
72 6
Direct
0.2223371087802946 0.2769643834713875 0.0049723054270672
0.0568243834642743 0.1107270402315876 0.9992336054772011
0.8898580302254615 0.9439749191179487 0.9985252802814486
0.0562388196141086 0.2772928116197519 0.0005100142563208
0.8901439993310660 0.1112567411010730 0.9992567988381893
0.7235764285985766 0.7780611012443757 0.9997330395997892
0.5577257635012813 0.6128530151008178 0.0045503208355342
0.7238319251885130 0.9445090473447877 0.9980885193295279
0.8901238675500025 0.2776627781575698 0.9990022409688137
0.7232889044796167 0.1108446907929803 0.9975272478834185
0.5569080018841390 0.7779384409153565 0.9978623183416531
0.3890496215815915 0.4453106701689933 0.0039870747768589
0.3903786997258294 0.6117388479014139 0.9980260057236308
0.5570399392489662 0.9447100506189869 0.9954607750542408
0.7233061342300502 0.2779185648386360 0.9984915270558758
0.2229658497874425 0.4442972005364112 0.9984888051274652
0.3906222885472843 0.7778607101765900 0.9951752095956508
0.5571463348232655 0.1108930949113329 0.9948588322235210
0.5566181343723642 0.2769807079766551 0.9980177056380199
0.2233858560735413 0.6108451318126669 0.9957593304273900
0.3901376772610590 0.9440633716007127 0.9932901842803830
0.3903719249059421 0.1109736138549806 0.9931430341457883
0.0565157088986723 0.4442831805302845 0.9981223145278690
0.2237187705666202 0.7778955093719375 0.9943965800086190
0.2238676139769851 0.9442211697427965 0.9935200865779237
0.3911718900497050 0.2763807615839724 0.9985738051189799
0.0570494619463418 0.6108453524580639 0.9967614319908762

0.0566843982944789 0.7773115311011054 0.9962137796929937
0.2230443255960708 0.1103209638095777 0.9957756544503482
0.8899230735378652 0.4439688154173211 0.9987563310586722
0.8901916720686458 0.6110052894415290 0.9991725818523278
0.0567403237987421 0.9444637344751285 0.9966357292895580
0.7238099872577095 0.4443138887524825 0.0012535191150178
0.8904228447004101 0.7776982055303776 0.9988231726796570
0.7235129086044836 0.6105534859128028 0.0033289438813782
0.5569026444334497 0.4435977240902531 0.0139872918650497
0.1671629019349723 0.1652795655492341 0.9997577351317233
0.0014678596503330 0.9994041834717038 0.9979550189891739
0.8348133583436450 0.8332887071191024 0.9989359343450346
0.0011625253769765 0.1665221579648701 0.0001148416389114
0.8344856884046266 0.9995100014191962 0.9987313808940712
0.5012936653719891 0.5011279973320697 0.0133756608347895
0.6683430418354277 0.8332849884562492 0.9986205027734130
0.8348317700464349 0.1664083338396551 0.9988482804398657
0.6680547010076694 0.0000289519322223 0.9969854870691890
0.5016148372135447 0.6673110655159320 0.9997879604544124
0.3347196991099395 0.3326483257904584 0.0078558235167776
0.3344315498307893 0.4998490635606387 0.9986333257772841
0.6677428388630489 0.1661489619842484 0.9968923443347251
0.5012391397674278 0.8331334735267717 0.9958752612863080
0.5015947875456774 0.9996889380115590 0.9941469249809600
0.3351241419482847 0.6664467449492122 0.9960009107728283
0.1674968969749386 0.3331263828701196 0.0020115525427755
0.3347224772335267 0.8331930051430092 0.9939891077705312
0.1679204060733213 0.4996713306289493 0.9970704452477719
0.5013208205373871 0.1662867771137755 0.9945833950491547
0.1680433490303415 0.6660095043386249 0.9954639785196733
0.3344426864854384 0.9992723930983658 0.9927488018451385
0.0008640032397622 0.3329531333915625 0.9992238306677805
0.3351604886010406 0.1661258692011884 0.9944309197410490
0.0015133045443392 0.4996642184292028 0.9978892435080553
0.1683366080080617 0.8329827005356094 0.9944494717064529
0.1681118604769125 0.0000548344567832 0.9948470277433472
0.8346730021889002 0.3333881042824203 0.9988915863491812
0.0014968111877280 0.6665073361934759 0.9973042610036416
0.0011499944500457 0.8327237423073370 0.9971298163619764
0.8345165123805316 0.4995956706676452 0.9994704846944558
0.8348562920449822 0.6661805146699109 0.0001086108563939
0.6683160199992386 0.3328256667847711 0.9997414076213360
0.6688596187950182 0.4999739298146295 0.0054706769739141
0.5007688467842399 0.3312866853519409 0.0013953803996642
0.6678266226992164 0.6667814959271041 0.0027035786614888
0.5172682514491456 0.4639451047871219 0.1297551766050162
0.4005469333377505 0.3182645702873899 0.2392064932122793
0.3177187392093899 0.3334417477255514 0.1263043398281312
0.5781389907060159 0.4809055728426017 0.2691726478101941
0.4693877040090300 0.3609125898600922 0.3671937768947870
0.6957112457428707 0.5983720773508381 0.1749559637088751

Pt6-min2 da Silva

1.00000000000000
14.7772044699999991 0.0000000000000000 0.0000000000000000
-7.3886022400000000 12.7974344700000007 0.0000000000000000
0.0000000000000000 0.0000000000000000 18.0009597799999987
C Pt
72 6
Direct
0.2154812836025499 0.2737800964767132 0.9847389125278099
0.0490556115411565 0.1073505501845702 0.9881734740211172
0.8824227183259765 0.9404574005390884 0.9921026829207094
0.0488635640840229 0.2737733493359258 0.9888543596897961
0.8821380588704031 0.1070912556634767 0.9925248518841201
0.7159861362698194 0.7747540982137906 0.9946408100696900
0.5488808412845643 0.6070558288638708 0.0013466612828594
0.7157430040704256 0.9409153281010276 0.9952847002766418
0.8824672161327314 0.2741140522863006 0.9958529572828212
0.7158514728912593 0.1070898249039161 0.9984923517150861
0.5494498370497709 0.7751416455836662 0.0008653761326940
0.3818079545752724 0.4400506961788651 0.9862754996190475
0.3811846872630653 0.6066818298495491 0.9966151917418671
0.5490957519709667 0.9408809732251058 0.9987973320616987
0.7160840760284941 0.2729011930953860 0.0051578214138246
0.2157261868631082 0.4406128859422580 0.9881698644023302
0.3822300184326579 0.7740065863200911 0.9979296051478386
0.5490559704080837 0.1071310728678796 0.9998385443007862
0.5476887023829278 0.2728135028708891 0.9992217274354829
0.2152719181251967 0.6069664732866045 0.9934027635139131
0.3822448237860883 0.9405889188800955 0.9959404656872550
0.3821675228700769 0.1071599648027757 0.9919976829009158
0.0488933338701472 0.4404381961118702 0.9923078633131439
0.2155268492229041 0.7739609719528247 0.9942413264644472
0.2158411786150154 0.9408245502895269 0.9915035644378349
0.3824502938657659 0.2737753454366043 0.9862282555939430
0.0491133685508771 0.6073951661226715 0.9945271628190966
0.0492017519921788 0.7741755117716380 0.9937778483603168
0.2157058587180885 0.1072514984852262 0.9871922822396044
0.8826802915147027 0.4408555417655933 0.9993168376410395
0.8829568842176059 0.6079015251173487 0.9998944923730519
0.0492040450379392 0.9408420533462731 0.9905427791957635
0.7166370324278546 0.4408062869038361 0.0130217454335764
0.8830399217527969 0.7743877197889830 0.9960471001057485
0.7166942243508032 0.6093931137083146 0.0073196812793483
0.5475060632285818 0.4396207236245715 0.9922819135735352
0.1604300596082524 0.1630673566496270 0.9860606982708262
0.9939795957936468 0.9964748280923956 0.9901862853102941
0.8276051328396505 0.8304532927627264 0.9941153163217535
0.9938262844404946 0.1631688594721155 0.9895258394068165
0.8269432917130572 0.9965632530183086 0.9928818269036910
0.4927182857085270 0.4949229496179086 0.9900314702067803
0.6601583696242130 0.8303582224046124 0.9961181279045377
0.8269616365945538 0.1628723698364780 0.9957380365332043
0.6603380586602725 0.9965444556470118 0.9977557715778076
0.4929913905750638 0.6634220445393808 0.0035427767203515

0.3270660147323383 0.3296931832132302 0.9842277044151544
0.3264798967037166 0.4962642544721589 0.9894570284042317
0.6602976044738966 0.1626402763597241 0.0016230999613498
0.4936804346021120 0.8301861385979237 0.9995579886887569
0.4937901610717219 0.9967267581880179 0.9982458347293957
0.3266589536057580 0.6629454488047344 0.9961352560661823
0.1603751557071860 0.3296175509741133 0.9863787165210880
0.3271715811072156 0.8299027183458421 0.9959881769055414
0.1602690714912001 0.4965557514123304 0.9910943212292267
0.4935231361576200 0.1631295686126037 0.9970495998243081
0.1600986018038455 0.6631118853536080 0.9938695774258832
0.3270760242581332 0.9964331921965908 0.9929154182303463
0.9937696652860168 0.3298208890411516 0.9919631589367768
0.3271217990457345 0.1629076790923776 0.9879584577093468
0.9936556271875716 0.4963784197050245 0.9950024467356684
0.1605831064532950 0.8298409541737612 0.9930123844504379
0.1607152003028531 0.9966200433566854 0.9894357466611794
0.8274230020774240 0.3295365584025092 0.0006155391115854
0.9940003236077217 0.6634703250430505 0.9955959147009423
0.9942312318312361 0.8301836070445603 0.9932546741690018
0.8281629128147259 0.4969908273411008 0.0034745831356062
0.8289504935226333 0.6645012957323786 0.0014270736080064
0.6592621612707603 0.3279032219421225 0.0112295415433366
0.6583230799897777 0.4966862071320080 0.0088290689078647
0.4934464650190691 0.3291324928470303 0.9908297686520697
0.6617593982678400 0.6656894703325804 0.9981950322560209
0.6575151324566733 0.5433327920603972 0.1213953963619332
0.5853723392709469 0.5812760341262049 0.2439263813397545
0.5272401724189990 0.6108267405108734 0.1196878911027639
0.6924004359813267 0.3866702113729019 0.1285218974253155
0.5817235265399532 0.4034868886248901 0.2255550117808340
0.5188848857483706 0.4434686774584478 0.3416124367676900

Pt6-min6 da Silva
1.00000000000000
14.7772044699999991 0.0000000000000000 0.0000000000000000
-7.3886022400000000 12.7974344700000007 0.0000000000000000
0.0000000000000000 0.0000000000000000 18.0009597799999987
C Pt
72 6
Direct
0.5541827997590396 0.9441919863838419 0.9973406304314332
0.5536425096298520 0.7775317263305741 0.9942195862347850
0.5535402031600968 0.6102822797150710 0.9912630992095255
0.7202760761993701 0.9436938110002748 0.9973734812457956
0.7208070793471765 0.7775812295635923 0.9960812207036321
0.5541866262272348 0.4442743912400622 0.9934727230409502
0.5537317347363524 0.2777260233151063 0.9968166206576879
0.7203138270166493 0.6108499512713430 0.9945191281158838
0.8870178978739695 0.9442955141946677 0.9981267970693896
0.8869654841570112 0.7770408533727977 0.9975605549448635
0.7203098803731188 0.4437866671663715 0.9965047614946649
0.5536464466965554 0.1104444024573824 0.9984328223851059
0.7208624304912130 0.2776311490422632 0.9983321738399710
0.8874923970109064 0.6109351485114096 0.9974394504447136
0.0541813818016886 0.9442820076989639 0.9985878381169052
0.7203528453620969 0.1109448105502952 0.9985942701141749
0.8870502109771904 0.4442989682874531 0.9980209475711845
0.0536933594629924 0.7776002994897365 0.9985072962495521
0.2203353080051209 0.9437740095717257 0.9986695071012259
0.8870303536880796 0.2771015466156150 0.9983154827665663
0.0536878305636555 0.6104260047680511 0.9977974529696705
0.2209029002472241 0.7775947854045352 0.9976944995888281
0.8875596817098889 0.1109376153895645 0.9983445897605685
0.0542581381532955 0.4442665523830240 0.9966996433239738
0.2204095901109847 0.6109507919028705 0.9952849182365404
0.3870578121199740 0.9442495071303615 0.9980843725783671
0.0537989843274658 0.2776835731769182 0.9966374902667458
0.2204026123884604 0.4437877393113920 0.9929280827954633
0.3869924047542707 0.7770034721810655 0.9952833088161193
0.0537563853437262 0.1105064864367020 0.9976782925111293
0.2209584735057031 0.2776998363123461 0.9943628541798560
0.3874596325437674 0.6108477487509062 0.9910916142395390
0.2203798767168262 0.1110190589795993 0.9974584592021399
0.3869743742593936 0.4443147056012942 -0.0089756907308079
0.3869351914065462 0.2771615004735707 0.9955966953256590
0.3875600710309309 0.1109775789782096 0.9979903128287125
0.4984869196539852 0.8327675263818392 0.9955131134876491
0.4978652257854042 0.6660267138595759 0.9918520914000480
0.4984412487918988 0.5000680708538815 -0.0086700982444772
0.6650267002461454 0.8333269640134859 0.9957617813636153
0.6649628629762533 0.6659616580239329 0.9941217461734376
0.4979925744642764 0.1662179197963465 0.9979309539560319
0.6646367379798771 0.4995080484378502 0.9945370441755865
0.8312398642801474 0.8328035752289036 0.9973817640382584
0.8317164986690958 0.6666214342352674 0.9965587644508517
0.6651197365002728 0.3333773193726252 0.9973091584239790
0.4984745657802184 -0.0000192400304891 0.9980880981412679
0.6651683686186411 0.1662028387614119 0.9985896585792601
0.9984311096365787 0.8328072057677681 0.9984737794646659
0.8317935824550552 0.4995157281632814 0.9974024210322832
0.9979787009291179 0.6661607593234344 0.9981396325735662
0.8313111115383064 0.3328450552165070 0.9983465667521363
0.6646150507988711 0.9994640161284574 0.9978779626523904
0.9985046225616125 0.5000305254163341 0.9976870334465597
0.8318258941921552 0.1667086611484547 0.9985411569943419
0.1651740589713636 0.8333743857223024 0.9985563189981302
0.9985642984300434 0.3328949446134175 0.9972863924151579
0.1652289825145409 0.6661992041521029 0.9970553456131535
0.8318234938170235 0.9995303705880312 0.9981481558426788
0.3313515090502743 0.8327760388565121 0.9972102753940449
0.9980360989752840 0.1662383635208800 0.9976675317328332
0.1646702803805040 0.4994367996401403 0.9949347906656959
0.3318645101799644 0.6666422791862603 0.9936127691306658
0.9984762694959989 0.0000504050856710 0.9983375389093626
0.1652412625887293 0.3334367338272010 0.9944363539567009

0.3318260837306963 0.4995364646929552 0.9906679883025153
0.1652289394848369 0.1662915658468379 0.9966649067544779
0.3312532004428629 0.3329249799067258 0.9931261301923145
0.1646222975850476 0.9995374199221914 0.9984986003577189
0.3318015147523379 0.1667608753773912 0.9970299230446443
0.3318752826217000 0.9995735011057403 0.9984708502211627
0.4986434491275902 0.3330288826586336 0.9951998581591204
0.4725531362552334 0.6381658473895914 0.1783253078844550
0.6584985929748538 0.6948292127268871 0.1701281313863686
0.2827589075933696 0.5614642646670446 0.1758892175537380
0.3394974927000558 0.4321040815236027 0.1818539322134224
0.5454833049756809 0.5051381714749438 0.1792017741765750
0.4161584922120128 0.3189730837576213 0.1757911535159302

Pt6-min Nakajima
1.00000000000000
14.7772044699999991 0.0000000000000000 0.0000000000000000
-7.3886022400000000 12.7974344700000007 0.0000000000000000
0.0000000000000000 0.0000000000000000 18.0009597799999987
C Pt
72 6
Direct
0.2262878458538111 0.2819098515116352 0.0031008986790582
0.0609384354930569 0.1158527343131937 0.9956834682523521
0.8945093198411342 0.9492192193407405 0.9935758720173382
0.0612769349152913 0.2825637779687398 0.9941619667893207
0.8945344983175119 0.1158687149383937 0.9930641020064286
0.7280665131847144 0.7825729001872688 0.9946984630891436
0.5615131846401340 0.6162994491500768 0.9993476286419423
0.7278543540005273 0.9489618240751057 0.9933862559828555
0.8947145141354866 0.2825676086877920 0.9924354109827505
0.7279405772933174 0.1157501105659478 0.9936699966654743
0.5610657773640284 0.7823611762439100 0.9949943055320531
0.3944987871783709 0.4509543541618726 0.0128044166504182
0.3944072094673672 0.6164240639793093 0.9972241672364035
0.5613705420603594 0.9492342879578999 0.9944414637550167
0.7280523548364926 0.2826260537784435 0.9952943453330079
0.2278509111875451 0.4497083123759822 0.9971677093420297
0.3947361217141960 0.7829337045689826 0.9943899180980651
0.5616491664010681 0.1158284263870897 0.9972729410057113
0.5622273923640719 0.2818847495864532 0.0052194971910922
0.2279892908519727 0.6162082089258689 0.9937044889998390
0.3948957066664960 0.9494808358306273 0.9964639438888767
0.3950000580707496 0.1158669062552491 0.0015886533746396
0.0612526582814823 0.4493499829373775 0.9924991333987663
0.2281672651474338 0.7830197790017905 0.9943511155003293
0.2281311059953453 0.9495726037786127 0.9971647646865520
0.3934819434194523 0.2806798095966059 0.0099659429522134
0.0614591071411610 0.6161624354918729 0.9927180524637222
0.0613965848738616 0.7829292014565326 0.9940551026053939
0.2274170177942665 0.1157900679845767 0.0005466093451361
0.8947078094292280 0.4491920353793688 0.9927947041708265
0.8949131778136632 0.6163201898868991 0.9938066174807787
0.0612809875821796 0.9495916123595904 0.9954449038026283
0.7287125103659164 0.4496237832019858 0.9979704494449564
0.8949381360796949 0.7829568198847738 0.9939111263768297
0.7286046566434479 0.6164727437237829 0.9975193705565815
0.5629142168334766 0.4510719889766719 0.0115616399138290
0.1721069409711831 0.1718405673519285 0.9996395612318878
0.0059905797503887 0.0053362685308471 0.9946873746019662
0.8395730520416294 0.8388062468809068 0.9938997734627080
0.0059172212044771 0.1720079867481275 0.9941778936562713
0.8393437516319509 0.0052601554866527 0.9931887076883612
0.5071807783024482 0.5075291244650799 0.0064393725519096
0.6727755992373261 0.8386899273223776 0.9942505778675113
0.8394832420717862 0.1720650673020501 0.9928204068333599
0.6726655134173427 0.0053269059989489 0.9937306592262800
0.5059222351143902 0.6724829796042400 0.9971598786694642
0.3376905857151158 0.3375537955637213 0.0146181727314243
0.3386328102920544 0.5064092871964689 0.0013239271801169
0.6727931809977505 0.1719130022138557 0.9953900086466732
0.5058675849357712 0.8386951494845007 0.9944776046553514
0.5061463616196988 0.0052868762875704 0.9959208422499728
0.3392459952349824 0.6723657368505016 0.9945461083258138
0.1723076013119282 0.3384648556445526 0.9975843493584549
0.3394399463423667 0.8390702298651860 0.9948854769724846
0.1728335340988139 0.5056502868390425 0.9937646597592291
0.5064384059407558 0.1718603095229270 0.0015921239270043
0.1727976807491416 0.6724075793374737 0.9932749816968780
0.3397633087494114 0.0058822491714494 0.9984265275794684
0.0060778282326268 0.3388871143261412 0.9926031802687802
0.3394525971110411 0.1722578275129862 0.0035906661454490
0.0062543373622930 0.5056402097375567 0.9922029875260350
0.1730951700465795 0.8394036059048346 0.9950335129966703
0.1728452492174171 0.0059510581626370 0.9976851942056513
0.8396232733307372 0.3389699084110518 0.9930394729181486
0.0062721552001008 0.6724770647734317 0.9931917346410941
0.0063010547333278 0.8390221954932500 0.9942353784160218
0.8394342630879876 0.5054790385515062 0.9942474643391961
0.8399512284725290 0.6724048757473682 0.9945622406454149
0.6730988670323015 0.3384983759958757 0.9990387379205430
0.6743170030253651 0.5063990850642242 0.0019364268472586
0.5062963456855556 0.3376866529465090 0.0156274854430691
0.6732342358190948 0.6723965827871581 0.9970279580509001
0.2173418327775209 0.3896917018321062 0.2026687903596169
0.5549208635847904 0.5542312855422793 0.3138569771386415
0.5419641514718023 0.4006816581221599 0.1283713455164985
0.5305773783974317 0.3884648796888968 0.2657604802008251
0.3787057788695023 0.4251618567700959 0.2653325632937893
0.3685627634973017 0.4014624897667645 0.1285129969604881

Pt6-str7 Nakajima
1.00000000000000
14.7772044699999991 0.0000000000000000 0.0000000000000000
-7.3886022400000000 12.7974344700000007 0.0000000000000000
0.0000000000000000 0.0000000000000000 18.0009597799999987

C Pt
72 6
Direct
0.2213737822145276 0.2829727536425111 0.9991798435134811
0.0550712813837606 0.1164975227539813 0.9966028876701358
0.8885638925019705 0.9498226043975038 0.9950622687899724
0.0550369941541078 0.2830263871151857 0.9955428886304389
0.8882684021803229 0.1160353778731746 0.9941236892560710
0.7214125684349426 0.7826962248660365 0.9950766572145113
0.5543170643146240 0.6166212460304905 0.9967782963870349
0.7214270933112132 0.9496818946381609 0.9922819808446093
0.8880406521579900 0.2828189331742195 0.9952827358093046
0.7218161619760366 0.1164653320213702 0.9929427037014520
0.5551321503777444 0.7831442310995840 0.9915973838736249
0.3877915009607094 0.4498252586492910 0.9985716964107638
0.3877646971021207 0.6159935606875209 0.9928703647064481
0.5547996399587376 0.9494140577485055 0.9910716200936065
0.7215972116063867 0.2825710715601488 0.9970708078435777
0.2210565667287057 0.4496574586035749 0.9951823748782189
0.3880241524107007 0.7829349756143813 0.9913277117608530
0.5547488443555508 0.1162867295503744 0.9938219895913036
0.5556754084261755 0.2821043427335965 0.0023825051991224
0.2214971307289986 0.6162268852690715 0.9934227364471155
0.3884141114001096 0.9495114584674909 0.9923980873458262
0.3875028594084995 0.1151030293357422 0.9978466785631994
0.0545422903856831 0.4491962009281281 0.9953865189190338
0.2211591464901730 0.7825338977714438 0.9937245927381753
0.2209691988881062 0.9493309584290515 0.9954761052604439
0.3861562159034122 0.2816795978694628 0.0102043286348561
0.0544844330129948 0.6161389920152018 0.9964780362141710
0.0548617811795253 0.7829988618135033 0.9967982335937009
0.2210565703675442 0.1156146996062546 0.9990169906457496
0.8884544482269803 0.4494143770564065 0.9985180285702953
0.8881938855413125 0.6162210783398265 0.0008484459938245
0.0545184140683119 0.9492207741703567 0.9964835969942030
0.7223869872732038 0.4492252254150415 0.0055357663483022
0.8882676425041112 0.7834798339763651 0.9977853868205244
0.7229812479142339 0.6181823000151070 0.0039877915230377
0.5545297752567961 0.4507919617702498 0.0119036956029035
0.1656219780649764 0.1716500515856936 0.9986519930277922
0.9989161871600203 0.0048519338258259 0.9961256302119779
0.8327700950381427 0.8384706214247117 0.9960111715017064
0.9992588442468673 0.1716885251541456 0.9954838934562247
0.8326275119324364 0.0053067044561885 0.9936997405517332
0.4980971884059002 0.5057674290019563 0.0010414735997983
0.6653739850314579 0.8383342008267221 0.9924768884757142
0.8322628539095902 0.1716561861927843 0.9939168020436568
0.6657467609826000 0.0049030723635042 0.9917172673529606
0.4990069103818158 0.6715441524666304 0.9929561560241495
0.3313338922840217 0.3386599216931927 0.0026600105331127
0.3321835941096154 0.5052989597103732 0.9949474785348045
0.6660363523265573 0.1718801165302324 0.9942321550785778
0.4990170342969051 0.8384527886761362 0.9908538423003677
0.4986556050580049 0.0047202571366824 0.9919012314098268
0.3319426041370548 0.6714162381020472 0.9921400275436838
0.1653039408314925 0.3384674433947907 0.9964220295603639
0.3323242886304882 0.8381304580840094 0.9920899677029738
0.1656666314458235 0.5050107124185246 0.9944668417343436
0.4994523726900510 0.1712010787679077 0.9973713122437076
0.1654977006025007 0.6718169036288657 0.9943209471033896
0.3323736535196957 0.0051291595148228 0.9946447063386188
0.9992065496617073 0.3386562401501365 0.9953075454696787
0.3307708248903012 0.1696696810464999 0.0023847742853249
0.9987047701495442 0.5048411864596289 0.9967042047883581
0.1652025104812296 0.8380294948204394 0.9951331710837934
0.1654667296652903 0.0043263247241327 0.9967845953409480
0.8326839684759122 0.3381824594860348 0.9970467842434658
0.9987007926910607 0.6713336658827629 0.9980086965601558
0.9992546258286268 0.8386049216935305 0.9969681008269120
0.8324294491963471 0.5049158321330722 0.0018976959628390
0.8327131695714840 0.6722537179682604 0.0008580540828333
0.6660217208158912 0.3380774535716924 0.0010618345406357
0.6674485553886882 0.5061895545145561 0.0123346020597666
0.4990733194548440 0.3370642784590885 0.0116875054737946
0.6663110877798104 0.6727192753413931 0.9984150139570076
0.4429454977755753 0.2966588106679211 0.1235778461033590
0.3477079815244437 0.2633935585286267 0.2473167768055191
0.4836931846068850 0.4604087834291448 0.2422762778098502
0.5459264689322794 0.3284018691512429 0.2505239376509678
0.6810878597465120 0.5258080519259920 0.2556556545592841
0.6052409903438587 0.4909310880454925 0.1247081847395819

Table S3. Coordinates (VASP CONTCAR format) for the 50 most stable structures optimized without dispersion corrections.

PT6(no-D)-I – ΔE = 0.00 eV
1.0000000000000
14.7772044699999991 0.0000000000000000 0.0000000000000000
-7.3886022400000000 12.7974344700000007 0.0000000000000000
0.0000000000000000 0.0000000000000000 18.0009597799999987
C Pt
72 6
Direct
0.8863267538439601 0.6136990367492672 0.9948289540727444
0.0535391341670746 0.6137401370749700 0.9890686763203078
0.2196599609298246 0.6131971670494138 0.9896583361382838
0.8863040776856650 0.4465135957026902 0.9912605591780945
0.0531077081120230 0.4472184876285183 0.9886375930350653
0.3863182494810928 0.6144129797950910 0.9988815000873572
0.5535855886776133 0.6170052104694292 0.0199959494295712
0.2204790117169679 0.4473343521552735 0.9938328521411464

0.8870843986110231 0.2806648742718352 0.9885508552220656
0.0532405656322865 0.2801173210666477 0.9897256214649417
0.3845525662705001 0.4460480432861829 0.0085678737870296
0.7210813469869848 0.6131418317185791 0.0073822893136679
0.5547823405849712 0.4476021661540699 0.0242390981507867
0.2196602103849230 0.2807743220759633 0.9970403022744208
0.8866728570459017 0.1141347863282149 0.9893018155257778
0.7207033101314479 0.4473090893573257 0.0021718184061754
0.3848971215372217 0.2788210189308558 0.0144202715508186
0.0540858736506493 0.1142012024750088 0.9918454973445137
0.8866665980903647 0.9470733119198371 0.9933631023432810
0.5535390503728692 0.2784865074018867 0.0048570722690897
0.2190241797672527 0.1125171764687707 0.9970957162435781
0.0527549723611003 0.9470682851479211 0.9922930855898784
0.7196351105250258 0.2805310471281999 0.9932680487804646
0.3860938263721323 0.1130326292070123 0.9979522704675716
0.2201104190323449 0.9469051867738045 0.9915806951145569
0.8871229236637888 0.7807673796003272 0.9966395005115487
0.5537720045299963 0.1138564566196365 0.9911415095028460
0.3864078643735169 0.9464216226139825 0.9892994226323921
0.0527869618199333 0.7796947420057236 0.9908254122475526
0.7198269044690448 0.1134420777277967 0.9887759871357460
0.5531909020901580 0.9474481616295094 0.9889159280550857
0.2195734443855741 0.7803742916654102 0.9885383092643494
0.7205635064563012 0.9475614732018585 0.9921099011513945
0.3870161556973954 0.7805690977873709 0.9898520190977322
0.5527559289063362 0.7805019157060187 0.9965651966447666
0.7207457679041198 0.7823994797482072 0.0039630393394887
0.9978275289294558 0.6695775855567874 0.9909225757943645
0.1639854878370315 0.6690389472131599 0.9883805922801017
0.3314386628422739 0.6691724677263267 0.9921110218464761
0.9979563202175399 0.5024838905676177 0.9890918915023619
0.1647739310572476 0.5032048921810244 0.9900870799310511
0.6660029445426758 0.6708990419228229 0.0154339171670870
0.3303819852257774 0.5023767176246992 0.0003444664613426
0.9975311397131534 0.3359620527628522 0.9885426186643613
0.1647512595175655 0.3360187499138334 0.9930200978696888
0.4959397472048508 0.5018859223692829 0.0237068823490247
0.8313991661558191 0.6689309061093951 0.9989439238834592
0.6664698033513616 0.5036097486186151 0.0102420407001986
0.9982805427884713 0.1699947465973253 0.9899282374571925
0.3299329643868134 0.3365648030244728 0.0055896987466895
0.1639210417999593 0.1689302221556730 0.9952671354792528
0.4971637723565721 0.3325421929727099 0.0201071013281862
0.8306147772752581 0.5023778379714372 0.9956360482657942
0.3302452120915049 0.1672742883428668 0.0033168771886807
0.6647316128933980 0.3352499915172658 0.0002952832988541
0.9982610683094677 0.0025929272755576 0.9921037129972916
0.4982930199046791 0.1691501218922653 0.9970520185919156
0.1643522095481345 0.0025926297389489 0.9931801143048702
0.8314218156915274 0.3365137717734754 0.9905998300187591
0.9969439980647792 0.8354673513875994 0.9927934153632449
0.6640941173126151 0.1691386835017212 0.9905714120489506
0.3305159729610239 0.0021023966718587 0.9921464148338330
0.1644024946716911 0.8355429460191388 0.9896327073495428
0.8315240155893875 0.1693235969763762 0.9885846123766555
0.4979335834351915 0.0022492064885498 0.9890252461575244
0.3312797544561406 0.8362626252616536 0.9884611559419341
0.6647024769250152 0.0032710642286702 0.9893175010506710
0.4973319240771161 0.8358352997233141 0.9906082634781157
0.8309587895730886 0.0027690710808344 0.9914999892215874
0.6649610814956191 0.8366292052831525 0.9979738681031094
0.8319832427824849 0.8371668940948140 0.9979430136562399
0.4974329579370433 0.6711309193203334 0.0040173504000265
0.6164778715049692 0.6649955797535938 0.2713397896134282
0.5193706342161519 0.4736857430850350 0.2738436705288194
0.4219435493406962 0.2823529788735399 0.2709337665728313
0.5235247386515525 0.4740784559126467 0.1377792589106761
0.4209997300258124 0.2784267448846549 0.1325884427109045
0.6253705320963903 0.6697002993748242 0.1330832294313140

PT6(no-D)-II – ΔE = 0.03 eV

1.00000000000000
14.7772044699999991 0.0000000000000000 0.0000000000000000
-7.3886022400000000 12.7974344700000007 0.0000000000000000
0.0000000000000000 0.0000000000000000 18.0009597799999987
C Pt
72 6
Direct
0.8820186673553252 0.6136758585389757 0.9969187284820791
0.0481705177480762 0.6131894224896826 0.9898800059061799
0.2146362091830625 0.6130248099507298 0.9891194802319987
0.8812710280447220 0.4468575547974112 0.9927163745940035
0.0477056776496880 0.4464548879627728 0.9900524342546362
0.3812149275575649 0.6134245822938524 0.9933739321928385
0.5472311368908578 0.6147432085854945 0.0059771340802754
0.2140655991117413 0.4463255418860470 0.9937284745938229
0.8809391493691621 0.2801434165542815 0.9898728536826411
0.0473614409567276 0.2797502310944111 0.9908339638318324
0.3798832728769170 0.4473849423422820 0.0057049062128129
0.7165579255497221 0.6138353983173772 0.0138265304931764
0.5488005026261362 0.4457480002321574 0.0155610564331496
0.2127263133790223 0.2790731048789468 0.0005082320601275
0.8810729003870676 0.1134826982006985 0.9893210165445723
0.7149460517962396 0.4467334171871755 0.0001047827403653
0.3807058750938310 0.2780098097211692 0.0135063317870419
0.0477974639484202 0.1133341957555132 0.9908367676594310
0.8812202465188790 0.9467661664482279 0.9910937909617772
0.5478411000282435 0.2796303232413209 0.0000268408409312
0.2141713495475344 0.1126004307324351 0.9955815483591834
0.0478596082214793 0.9467032642570246 0.9900761522157708
0.7143859788257316 0.2802105720424422 0.9931429399654021
0.3809086254403198 0.1125614469710570 0.9966704788470935
0.2146657954721150 0.9463934649117505 0.9901860447871798
0.8819869294748415 0.7804284318762953 0.9959701987501290
0.5477245514012523 0.1133090349925325 0.9926687493237836

0.3813416943112173 0.9463633862294216 0.9898248534242882
0.0481619937280655 0.7798900728931386 0.9903368705435440
0.7143961620011581 0.1136060394013256 0.9899600703523959
0.5480747073117982 0.9468440370824354 0.9902309838090702
0.2147624246752216 0.7797531327004208 0.9881012794519322
0.7148212210790632 0.9472250531760480 0.9911864028904702
0.3815088416418178 0.7799032238228136 0.9892185356806493
0.5482834988255973 0.7805644682106205 0.9940540618839364
0.7155051099640559 0.7818639124314402 0.0010454437629690
0.9932221798447785 0.6687400909086989 0.9916714622641152
0.1596087583860424 0.6683221621268984 0.9883670491850083
0.3262419131095058 0.6683079843434996 0.9898732627129618
0.9928557720258766 0.5020135248340196 0.9906411196026710
0.1593346253646004 0.5016506018064533 0.9904423856409466
0.6596357780941489 0.6706073709862608 0.0128274350113458
0.3257545981568768 0.5018445081086043 0.9964480005459393
0.9923748267492059 0.3352704227194607 0.9899054084379131
0.1586159825170483 0.3349107747032463 0.9948783978611857
0.4916227310897838 0.5029610106780638 0.0140768169747787
0.8278502399505570 0.6695020362050315 0.0022016836639480
0.6607381024747099 0.5012877540344434 0.0084566084778572
0.9924432648683919 0.1685752136547549 0.9900889531850936
0.3239720733484930 0.3349470282212721 0.0123246882383157
0.1586526706033027 0.1680880985665141 0.9952094985560578
0.4932689389162093 0.3338354900855478 0.0083357278434946
0.8266099542610448 0.5022361219712437 0.9966611336430589
0.3250906143589631 0.1667379133459903 0.0018414132132918
0.6594642301465754 0.3351201522516760 0.9971646437544166
0.9927719725015720 0.0017742786257884 0.9900456365476629
0.4923485327697961 0.1679769255213870 0.9965304203886944
0.1594296304806093 0.0017508571377363 0.9910671243895521
0.8259620098556155 0.3354769142625642 0.9916790685137116
0.9928140905299827 0.8351380591836133 0.9912777774386967
0.6591040672225290 0.1686279836475961 0.9916823219939630
0.3258182818903634 0.0013368100514128 0.9914816849814088
0.1595359908659333 0.8350076244966900 0.9888293865277760
0.8258146278022878 0.1687177458432672 0.9894493325346403
0.4925241750165412 0.0016960474746881 0.9906473281845933
0.3261973642677916 0.8349160229342729 0.9883903319773850
0.6592535651416540 0.0021698610821517 0.9901357120917567
0.4928910509290532 0.8352211069432371 0.9906668482972876
0.8259754776019008 0.0021236402479019 0.9903268998920893
0.6596933011605799 0.8359913458169999 0.9953160642734389
0.8264886561157923 0.8359429316127915 0.9956597511293808
0.4927868680323897 0.6688785594772142 0.9966598464239667
0.3468066490372763 0.3176834251456029 0.2667255572267280
0.6776793393937268 0.6468795348376517 0.2670993807710786
0.3466359690948266 0.3057791073157787 0.1293747094203539
0.6890294349953905 0.6471979056476656 0.1296866277029309
0.5180478499933301 0.4762014977153086 0.1314918017511388
0.5127529625667790 0.4817604939860871 0.2669957164317012

PT6(no-D)-III – ΔE = 0.12 eV

1.00000000000000
14.7772044699999991 0.0000000000000000 0.0000000000000000
-7.3886022400000000 12.7974344700000007 0.0000000000000000
0.0000000000000000 0.0000000000000000 18.0009597799999987
C Pt
72 6
Direct
0.8889815754734300 0.6060722960203719 0.9931872727772273
0.0551025106804417 0.6057822338231844 0.9920845453321192
0.2215000811209222 0.6063216534616203 0.9993354277310829
0.8888587035427449 0.4392429344098048 0.9931632398847015
0.0554127191930931 0.4393229954810636 0.9943986624989947
0.3887478998572718 0.6078585720336704 0.0159045988906428
0.5569073279811860 0.6076050011341678 0.0179226542839572
0.2201911043872400 0.4384716815920413 0.0048605476482777
0.8885055993533584 0.2724354750858282 0.9918980593058535
0.0550196448116296 0.2726819216039544 0.9939473161984580
0.3870462782616499 0.4372700625675208 0.0107295795444315
0.7236191624205830 0.6080778348168820 0.0021329603451932
0.5559824656841741 0.4371256596569850 0.0133120875878774
0.2217684642394673 0.2726707101738910 0.9976484572606310
0.8886743448540670 0.1060421334762722 0.9911778284778094
0.7228290603470242 0.4386589394193692 0.0009478732436534
0.3888920229623238 0.2722614461315871 0.9978541097802633
0.0553500714406496 0.1062462253246110 0.9912615785998042
0.8888580862066959 0.9397302535758527 0.9913678229822835
0.5550796974245813 0.2717728088458244 0.9957182131678337
0.2221366739946049 0.1062942745735000 0.9910489273475704
0.0555221926355927 0.9397726069310721 0.9892677740520952
0.7220935727274735 0.2723884043019780 0.9931898534106338
0.3887382183430574 0.1060539204873052 0.9908001171366791
0.2219962587038395 0.9394894760343746 0.9891329277606644
0.8889004276577595 0.7731287813061840 0.9921888568086814
0.5552718051893279 0.1055639529050509 0.9911413461393721
0.3885821771895763 0.9394837283731832 0.9911948415264433
0.0553099556295678 0.7729048207843903 0.9896098987265844
0.7218141409408929 0.1056344925355504 0.9914282442928695
0.5551988367356060 0.9390780301166046 0.9938895323941779
0.2219656886895933 0.7729009728838747 0.9921969830391717
0.7219318395508125 0.9390388051855538 0.9940055337232891
0.3883471095873006 0.7730521167064239 0.9990153737845873
0.5555086700012524 0.7729719267273936 0.0017412346773824
0.7227000462898943 0.7733174900944420 0.9987846544835861
0.9997239784258625 0.6620454164805452 0.9908813308981763
0.1665914845264638 0.6620588251064206 0.9939168250830477
0.3325387453997877 0.6630636326163710 0.0049920066748612
0.0000992967902604 0.4955853332541054 0.9926881611404639
0.1658398286042200 0.4951162214048708 0.9992595391798886
0.6684017941528850 0.6634560916262231 0.0056682956359353
0.3313781369352711 0.4940113120898957 0.0168003643306207
0.9998575887289860 0.3285312227147728 0.9933151047425071
0.1661252991266977 0.3286485052002206 0.9988254585589189
0.4989175868894407 0.4939386542585282 0.0197314454235880

0.8335812866537395 0.6627289999923534 0.9947873225386061
0.6681665449506156 0.4958781470517479 0.0080249493601698
0.9998244519001318 0.1619699723473076 0.9917349428736202
0.3331044410446609 0.3287197950680891 0.0014262640019425
0.1668204799453363 0.1624792174751093 0.9928733378795087
0.4994781827689749 0.3268238962652319 0.0014272847661942
0.8341639957487956 0.4955535765863104 0.9955902188211780
0.3334830184928066 0.1622087752513579 0.9924749936294432
0.6666952850714551 0.3280825918937325 0.9969697683479808
0.0001793438004754 0.9957447161662856 0.9900845871308661
0.4997240526101621 0.1615755985401108 0.9916813904945485
0.1667936371933081 0.9958028245180515 0.9891632630238973
0.8332472695254438 0.3285117601112262 0.9927213614018626
0.0002185806854911 0.8290943191108582 0.9896592389106758
0.6664637066994530 0.1616082953762188 0.9916064672698752
0.3331721367195684 0.9953472510811920 0.9897510998763508
0.1665427678149314 0.8288407762096099 0.9896521298083698
0.8332568078848297 0.1619305147109671 0.9913460121093749
0.4997340315354819 0.9952113004548409 0.9917438454793128
0.3329134095874906 0.8288039241985814 0.9935509175999471
0.6663906275522038 0.9951309857855009 0.9928842206037913
0.4997344430508264 0.8288304891816622 0.9981504685632245
0.8334231761565647 0.9953199722338155 0.9919299996103419
0.6670900108488240 0.8292132376486094 0.9981863524389070
0.8335761979582799 0.8290044256694102 0.9934764828204763
0.5009867935777095 0.6641157573813175 0.0108577701015946
0.3588869108805639 0.5565435361143471 0.1326462247112374
0.3568376203845389 0.5418770506627766 0.2700037419623200
0.5049914881888782 0.5118366178812437 0.2694634716068620
0.5231690509334186 0.5428539163334634 0.1350062053870218
0.6439315524321074 0.4679602965738994 0.2650921964831596
0.6407942725146754 0.4649270467050570 0.1271838067159621

PT6(no-D)-IV – ΔE = 0.14 eV

1.00000000000000
14.7772044699999991 0.0000000000000000 0.0000000000000000
-7.3886022400000000 12.7974344700000007 0.0000000000000000
0.0000000000000000 0.0000000000000000 18.0009597799999987
C Pt
72 6
Direct
0.8934387513812112 0.6158457060670415 0.9896473569274278
0.0600281733205108 0.6158476438815157 0.9916767973104825
0.2266675942724206 0.6161216349875929 0.9970701355407598
0.8935498502878190 0.4492662792238491 0.9931748290141442
0.0598488466331588 0.4490386790537997 0.9941382767953399
0.3940293866559870 0.6172622586781742 0.0025825128507790
0.5606763399209314 0.6163601546471114 0.9958202039560646
0.2251581116126786 0.4485848336366445 0.0019971120106135
0.8935320106074442 0.2825434262210607 0.9930441860782295
0.0601142236283678 0.2826166878609015 0.9929448141101673
0.3939635132815908 0.4489568294927366 0.0117943102077405
0.7267706740413971 0.6157440336874842 0.9909760316453244
0.5609170962383132 0.4505809385459543 0.0024849728075154
0.2265133272947395 0.2821680657864150 0.9966790566808399
0.8933907326092552 0.1159129571173096 0.9899685677362626
0.7272881525774437 0.4495033038685605 0.9968457337247969
0.3919745692631409 0.2816877326800480 0.0031762977704588
0.0600822320405854 0.1159163300583614 0.9895836706640750
0.8934054825328843 0.9493610610250371 0.9872973532758920
0.5605356182265666 0.2818643423603859 0.0110591521964452
0.2270025379269285 0.1160401823304014 0.9923353354008384
0.0601881623038025 0.9492743298200779 0.9877116919283395
0.7271967669998460 0.2826360796154574 0.9985430841620158
0.3933649965454933 0.1153902093170132 0.9972013690159969
0.2267683080777161 0.9492251872210531 0.9904666423310573
0.8933484479195286 0.7824669729370726 0.9870893994679832
0.5599131491333651 0.1154409646791947 0.9981017301875212
0.3935280444268940 0.9492523734632456 0.9934313296184598
0.0601254058302274 0.7826457545281400 0.9885305668090041
0.7267059848253155 0.1161249805044164 0.9936944231761160
0.5601282077715553 0.9490711378341885 0.9928935485104944
0.2268381533089396 0.7826118037716583 0.9920450669357095
0.7267061230828986 0.9492952752419086 0.9896284951678780
0.3935550675587294 0.7828215264153684 0.9945698510794188
0.5602071369618642 0.7826762238220084 0.9925938866931787
0.7267352796638349 0.7825537599168726 0.9885953796669398
0.0048281611965137 0.6711615621934257 0.9894784133910761
0.1716643034140546 0.6711701871938658 0.9933603026030724
0.3381749713588391 0.6716580679479165 0.9982292038549545
0.0046126404606071 0.5045856143843395 0.9926148951985851
0.1709934404682940 0.5043822255364958 0.9976188727093813
0.6712877054630226 0.6712362767773286 0.9909556231042700
0.3373874517281976 0.5049966028423825 0.0107120295805814
0.0047946545436943 0.3379904396441518 0.9931893518381045
0.1709442094164473 0.3378327257519302 0.9968500876178439
0.5061153091922748 0.5053774746691957 0.0024067470880524
0.8380897784613026 0.6711895687791625 0.9885880006222223
0.6718990070601691 0.5051385298626840 0.9959138022685039
0.0047658619542261 0.1712245070709457 0.9903669120019671
0.3372273425889887 0.3364310762873188 0.0030653557686762
0.1715865222734223 0.1714582588159033 0.9922609754663085
0.5044948655138342 0.3384493481126114 0.0119899996472057
0.8382128957559090 0.5046608081511152 0.9926181391522348
0.3377097451953048 0.1709633802676365 0.9968799279250646
0.6728114818320492 0.3385007736779500 0.0028543129195029
0.0048177067242321 0.0046427513347211 0.9877068926705732
0.5041210925571065 0.1696147207948044 0.0024346432950750
0.1714626379953899 0.0045399549315022 0.9896534975359188
0.8383593457609635 0.3380136338209496 0.9946803523030994
0.0049074274916023 0.8378656432718898 0.9872733809533756
0.6716613961592444 0.1711285508423757 0.9974901391920667
0.3381573048319169 0.0045718661243015 0.9932095058273305
0.1714580065112230 0.8378535584128102 0.9899173570260231
0.8381516259563142 0.1712950435821483 0.9921969909800907
0.5045812911021059 0.0043018832689157 0.9945221437491085

0.3380875935155814 0.8379946492203985 0.9930298330966352
0.6713947124491497 0.0044924192849223 0.9919679796981811
0.5048157263565969 0.8380173587127260 0.9932212844001640
0.8381898840534987 0.0045849159981657 0.9886190768240724
0.6713998317780252 0.8379073669521517 0.9896971451751426
0.8380142205454604 0.8378070641305371 0.9871016732760438
0.5050491369825778 0.6717522020888538 0.9967041905415215
0.5409314275757353 0.3194476534538637 0.2654872667349366
0.3734227524946192 0.4905376869163263 0.1259841513046567
0.3749408453289789 0.4856572564341448 0.2652464708228877
0.4275607326277537 0.3721399087849022 0.1898372611153789
0.5412948254521126 0.4859147406299940 0.2978234780243483
0.5460885456872688 0.3180659812214373 0.1262458826089556

PT6(no-D)-V – ΔE = 0.15 eV
1.00000000000000
14.7772044699999991 0.0000000000000000 0.0000000000000000
-7.3886022400000000 12.7974344700000007 0.0000000000000000
0.0000000000000000 0.0000000000000000 18.0009597799999987
C Pt
72 6
Direct
0.8951276300912880 0.6183460656713464 0.9881901447639905
0.0620022567280287 0.6181408595348898 0.9877607943687181
0.2281089078376723 0.6181901066578135 0.9910279825749910
0.8945434624888335 0.4510651428110890 0.9883320104290405
0.0614830762881837 0.4518171427264477 0.9929151406048149
0.3947268599391123 0.6188331950780039 0.9986845007502296
0.5632307124728442 0.6201314842830357 0.0101842927448814
0.2287667984594393 0.4529910868823706 0.0026079541636008
0.8948200204899592 0.2845711362027998 0.9919930851326768
0.0598101221880540 0.2841931582060582 0.0029488272552882
0.3943035182035999 0.4521551340623162 0.0175721812623308
0.7287116348572340 0.6182441114098722 0.9952106940098773
0.5630279255197053 0.4514899037857987 0.0026634870619446
0.2254749443375914 0.2825015788051815 0.0213385326257338
0.8947821534023817 0.1182990719530750 0.9948645691343145
0.7285366509491453 0.4512941298581623 0.9901830278218640
0.3961277921216109 0.2840393439475690 0.0101516115043836
0.0613416543610015 0.1172587882799618 0.0005370511459049
0.8947288079943618 0.9514229587276120 0.9935072559340767
0.5614106800587280 0.2845300754389655 0.9948323002176735
0.2275273484664808 0.1174116376789271 0.0034413128647017
0.0617370982825065 0.9517550609122551 0.9939549834267964
0.7280807925006130 0.2849027095613224 0.9895015198086057
0.3950509874288741 0.1186449171201929 0.9969813828428613
0.2285557535643363 0.9515267288113591 0.9929557038527577
0.8954664817904785 0.7849773583973274 0.9915136964262032
0.5617616828170640 0.1181530488878408 0.9912583570061102
0.3946615185005129 0.9515269640755619 0.9908805851033975
0.0614710496141555 0.7846792527243878 0.9890579402220752
0.7278492388116717 0.1176946418597211 0.9908918261175188
0.5614993556894277 0.9517684169180427 0.9911332725448716
0.2284199065771162 0.7852478169364261 0.9887524761000179
0.7283347836347858 0.9511467280814614 0.9931620453441212
0.3951133598303258 0.7849967104742959 0.9911860474518974
0.5610167822406709 0.7846156027516074 0.9964453199360364
0.7290932522255744 0.7858468109197361 0.9966645866452808
0.0062193426406481 0.6740766718355076 0.9876009766770579
0.1725979073601422 0.6736796941563128 0.9884053176441938
0.3395524512096202 0.6740662336706933 0.9927746453544657
0.0065794155238024 0.5070853526877670 0.9890182211587586
0.1728497788046894 0.5077379098819108 0.9945441274416069
0.6745359898545686 0.6745779540570567 0.0012364727299214
0.3384310557536310 0.5079788890893084 0.0043667992805396
0.0052701495475418 0.3400534531408184 0.9956696546950141
0.1715515970774177 0.3409472360382608 0.0128822813009180
0.5088324309923777 0.5080204121404464 0.0131666547179918
0.8399891512051454 0.6739117789526290 0.9906987287273168
0.6731527894077942 0.5070248418772607 0.9946111005440628
0.0053776782677843 0.1734438841652022 0.0001114263693509
0.3405666269301690 0.3401317518451634 0.0232290591615580
0.1713513454895192 0.1714648843702307 0.0077174545041316
0.5062805652671045 0.3398336971169442 0.0019570437550698
0.8394076711491678 0.5067869789946684 0.9880919660947427
0.3401265783166352 0.1739382411452297 0.0043038734045666
0.6729539017914732 0.3403734727291194 0.9907546451988480
0.0064472906921793 0.0070914403282600 0.9951518608736052
0.5060247340848321 0.1739787455837174 0.9942423327679819
0.1727455735305341 0.0074334502945064 0.9963714456195731
0.8392280996747132 0.3406330890214733 0.9892826353919020
0.0060701708870141 0.8403157806098918 0.9908881875243623
0.6723444702210273 0.1734072023165751 0.9902547352700495
0.3391543029776116 0.0069441848527347 0.9932883344352561
0.1730759717425130 0.8407241856070371 0.9896945021073194
0.8394821799830154 0.1736802246742712 0.9924829985935020
0.5061936690869615 0.0071623986355362 0.9908764518889797
0.3393306390954720 0.8408038385593315 0.9897539152600032
0.6726128287408812 0.0072761357902280 0.9916565551107155
0.5056732445560428 0.8402634981317405 0.9924641475721856
0.8391936468723102 0.0066182998516808 0.9936386585488250
0.6733542150958414 0.8406893133408104 0.9956475661837061
0.8399268133274234 0.8410035427860052 0.9934661735104484
0.5058965794445811 0.6752997841614956 0.0019813872907335
0.1811325571285209 0.3064340786676425 0.2692009051803268
0.1768327436410004 0.3074573997062373 0.1313318236668835
0.5542020868956286 0.5683712141380468 0.2664182710033529
0.3736955818209822 0.4159438016253389 0.1347409771297876
0.3677744393626057 0.4382302400484548 0.2696218210666501
0.5566678965070011 0.5729644910395777 0.1286890363620472

PT6(no-D)-VI – ΔE = 0.21 eV
1.00000000000000
14.7772044699999991 0.0000000000000000 0.0000000000000000
-7.3886022400000000 12.7974344700000007 0.0000000000000000
0.0000000000000000 0.0000000000000000 18.0009597799999987

C Pt

72 6

Direct

0.2217048590466448 0.2711724944977328 0.9945790416661631
0.0552847222535082 0.1047622008613617 0.9911297632982392
0.8885229307210387 0.9381412981594369 0.9919507753131834
0.0551168203979155 0.2714616527428181 0.9934763269461289
0.8887591608880752 0.1050959173023876 0.9908354969169617
0.7220447352822456 0.7722913223885691 0.9945844936201625
0.5557774988156801 0.6055207114708117 0.9964033271675774
0.7221376837416820 0.9385128292975726 0.9904527534042008
0.8888432860864481 0.2715189767986569 0.9929405970004237
0.7220077954293735 0.1050712385061647 0.9907487846474735
0.5553908225875196 0.7719539793370445 0.9921802388378747
0.3887669605344389 0.4374533324733802 0.0049247124557258
0.3893281997453934 0.6068537500026301 -0.0014359615361371
0.5555386504179344 0.9388318182067283 0.9904867137823210
0.7223671743449277 0.2718290166801632 0.9937859123733566
0.2208217917499553 0.4376730054855695 0.0006342903774629
0.3890892979500754 0.7721797720108250 0.9925444184954186
0.5555921454595042 0.1052790813828317 0.9913907735090691
0.5550707733911104 0.2716567910475244 0.9956362747977019
0.2218349042748462 0.6049944087045834 0.9966555071739860
0.3887342442275606 0.9385161275139474 0.9908311451884246
0.3887158563380271 0.1052239094647220 0.9911193389010099
0.0551908809089186 0.4384180453210162 0.9958092223699316
0.2222322549087553 0.7719698879906829 0.9930600180270232
0.2222633298883793 0.9383962004259280 0.9910374139468395
0.3882711615907185 0.2711614614999006 0.9940591528386544
0.0555518373421821 0.6048512949803948 0.9957693235333596
0.0554804954949205 0.7717149227554051 0.9943015095172441
0.2219005812320059 0.1047862530044182 0.9908470739691232
0.8886200608557536 0.4381164274335194 0.9960356589759932
0.8893164447830342 0.6054467771277050 0.9994524409206093
0.0556205019830915 0.9386322216327230 0.9917188056167860
0.7228560416253434 0.4372925901855645 0.0015793157839810
0.8893648596808341 0.7721551468660027 0.9966144723114725
0.7227435157791057 0.6064239214404672 0.0082217700485810
0.5545595020026918 0.4379488221777002 0.0002607010040796
0.1666862649975245 0.1604119728644134 0.9915980288877644
0.0004803357280266 0.9941028820875371 0.9913903919253757
0.8337221917944021 0.8277777852816293 0.9945527246076438
0.0001153648581666 0.1606759477360870 0.9915368918290464
0.8334708769579189 0.9939442202220239 0.9907561878358553
0.5012059747487221 0.4943248803561789 0.9984459545276965
0.6670838101683405 0.8278611438466833 0.9915777721274488
0.8337088317532847 0.1607786253829114 0.9912590244124626
0.6668565718966279 0.9943375483427125 0.9902061428712623
0.5005243299758774 0.6615351509934065 0.9948679220877373
0.3326528674550537 0.3258008156396519 0.9975142029022773
0.3331505887957902 0.4947078335026681 0.0063853684090177
0.6668840580034393 0.1606817277874636 0.9917076513356562
0.5001840930414649 0.8276601517521176 0.9914853742806700
0.5004253093778273 0.9944980948956387 0.9906015683522246
0.3340754829816457 0.6613769582851174 0.9956043458580578
0.1666129321170735 0.3272177216011107 0.9961289730287797
0.3336681481852266 0.8278386655687470 0.9918427095707030
0.1664901857668165 0.4941947197766347 0.9980341845804152
0.5003141356539439 0.1612645299448281 0.9925050929298244
0.1669426629136999 0.6605069882001187 0.9950233102566300
0.3334783527560602 0.9940481524583968 0.9906575955553810
-0.0000057958897075 0.3271672269347345 0.9941672398929439
0.3333815109704849 0.1603632521621045 0.9913598123600645
0.0004347870724097 0.4941736901725762 0.9957482742472236
0.1671346451075694 0.8273583012806009 0.9925067587781689
0.1669433509697435 0.9940924704183585 0.9908356307758971
0.8335304264198663 0.3273916334960585 0.9942603183948385
0.0003451428242002 0.6608697406730296 0.9962938149653068
0.0005222684532956 0.8274636023371951 0.9938804209661580
0.8336704540642538 0.4935119638806199 0.9992086278902125
0.8351338785450554 0.6618224520012459 0.0013649060173215
0.6674020438384221 0.3275653867047446 0.9967046282392770
0.6667663823859329 0.4934003669737611 0.0098995842610768
0.4998150821903631 0.3270669681951617 0.9967513597795661
0.6663644206403532 0.6617349640996552 -0.0010160215757282
0.6981619714063181 0.5548849627275988 0.1241918938006132
0.3592353297328748 0.4657350454520182 0.1214599879297309
0.5208888628824037 0.4827045289719664 0.1832019746710186
0.3637705348532042 0.4563737363834805 0.2604348644936769
0.5472954856397458 0.5903832070322602 0.2998095160949343
0.6829919743277767 0.5397496538670528 0.2639309643370723

PT6(no-D)-VII – ΔE = 0.32 eV

1.00000000000000

14.7772044699999991 0.0000000000000000 0.0000000000000000
-7.3886022400000000 12.7974344700000007 0.0000000000000000
0.0000000000000000 0.0000000000000000 18.0009597799999987

C Pt

72 6

Direct

0.8863146579224050 0.6086496813332190 0.9923767135049815
0.0527542862092645 0.6090117321755102 0.9898150194621920
0.2197691219748705 0.6088810413151577 0.9925360163743377
0.8867485503004957 0.4423433132918504 0.9913589446579181
0.0527133542592182 0.4416523593813437 0.9890404827865069
0.3843590345727677 0.6078662888446686 0.0016068953371260
0.5532394413447079 0.6092409723760497 0.0143148008150362
0.2195853069802354 0.4424936738727965 0.9908891176262600
0.8860196620615994 0.2757018889383929 0.9906287642324969
0.0535609332684857 0.2757954738863830 0.9883539730088913
0.3872061407055369 0.4426159654907380 0.9989380159842156
0.7207012528920487 0.6093399662000323 0.0010534689407322
0.5532245072281583 0.4418885457848347 0.0135554009915353
0.2195744517313045 0.2751535985513485 0.9904698719561651
0.8860015234080620 0.1083244252561713 0.9907202964897479
0.7200828376553261 0.4430310102122093 0.9988648054807854

0.3843644420071897 0.2745094692105425 0.0003252267148568
0.0526967685725737 0.1090892062636826 0.9888260049434550
0.8867197645829776 0.9424069310688736 0.9915893765134243
0.5538721466694909 0.2746265779203725 0.0043411251332515
0.2197598148675581 0.1089243784254847 0.9917619241525770
0.0527324389108514 0.9417728396632441 0.9896409825011432
0.7194405640477939 0.2751801896926409 0.9960725992096684
0.3859911258064983 0.1081530693833912 0.9980361084214096
0.2193864685614813 0.9423606681289129 0.9924755562597980
0.8863018479977072 0.7756942053081630 0.9925459508699319
0.5529399473655090 0.1088242964447161 0.9985194521107914
0.3868154964252639 0.9424179900944480 0.9969159507186944
0.0534139518636039 0.7757269706850067 0.9902763346598604
0.7201072481908355 0.1090569146841673 0.9950306433992608
0.5529371023926046 0.9421168389456582 0.9989770560484459
0.2193875955797822 0.7750738885622184 0.9928246432707368
0.7194176790487958 0.9422509716289227 0.9965518187995954
0.3859843141155253 0.7758615040787760 0.9990611442195174
0.5538686252119120 0.7772388816812992 0.0054435634571703
0.7200462660586950 0.7750384107746555 0.9995144713149742
0.9976173908517225 0.6642477896361072 0.9902764641520960
0.1640750114764344 0.6648137377846552 0.9912339728663895
0.3297078129109607 0.6638646798900396 0.9974951253518469
0.9969108827988720 0.4975150012979128 0.9896463777760687
0.1642575282733745 0.4975177935961952 0.9903280281612084
0.6657690982713262 0.6655813763241980 0.0049820782639927
0.3307243686770747 0.4982093741976712 0.9961555259849959
0.9976178076669697 0.3315451552936537 0.9888598955288685
0.1636558904989585 0.3308544760498435 0.9890312581449265
0.4958918976241620 0.4969222304826246 0.0078954344824993
0.8308684653453895 0.6644406024324141 0.9947970143190972
0.6657769529750936 0.4981625791861148 0.0044555618366999
0.9976046878033742 0.1640901617983701 0.9888007680808286
0.3307286497111050 0.3305667006125006 0.9954775233481428
0.1642414886494024 0.1647838156601509 0.9898098664589554
0.4961359756785981 0.3295013340110700 0.0109085731416982
0.8308834636320341 0.4981946734867080 0.9935670407540727
0.3297149449972565 0.1638552223542860 0.9963072064746186
0.6638928761511522 0.3304838794007097 0.0001207242832066
0.9968837119546023 0.9974027786005308 0.9895683530999477
0.4974024083547022 0.1634991297746424 0.0006404428248246
0.1640580865992618 0.9972930404826954 0.9907680481663945
0.8310151884354440 0.3308701071511848 0.9924222969890977
0.9976010401225324 0.8314006531242200 0.9902733999928017
0.6641390996434851 0.1646170211842204 0.9967721515602719
0.3309450263129960 0.9982236569048908 0.9956239723041378
0.1638421248717918 0.8309381413419530 0.9914539773494226
0.8302613179996641 0.1641303058316481 0.9919815555197360
0.4968741768861378 0.9974314283486265 0.9980462503434993
0.3309471801692965 0.8307391571406342 0.9961437556501593
0.6641290739118233 0.9975125779843026 0.9970929195019451
0.4974220724115668 0.8319011681529389 0.0015936264912284
0.8309874101044983 0.9981424165968988 0.9926979564726892
0.6638465026522056 0.8313762390329984 0.0008853660088519
0.8308565091143763 0.8306958014911956 0.9939423933812321
0.4961571077928468 0.6646096990503594 0.0123372040021650
0.5330191974100202 0.6552018142956584 0.1275027550512675
0.4804234960107152 0.4874896300247400 0.1957753216453497
0.5326450257572759 0.3732910545584431 0.1261851958442861
0.5306684797136825 0.3663307891823351 0.2668881581135594
0.6299438164508970 0.5609363593835113 0.2897247101056308
0.5319450307701885 0.6579982521314918 0.2682948426491052

PT6(no-D)-VIII – ΔE = 0.38 eV
1.00000000000000
14.7772044699999991 0.0000000000000000 0.0000000000000000
-7.3886022400000000 12.7974344700000007 0.0000000000000000
0.0000000000000000 0.0000000000000000 18.0009597799999987
C Pt
72 6
Direct
0.8895812446752203 0.6177564382203045 0.9907132149681601
0.0562489331514442 0.6176730675476705 0.9911855040366504
0.2227516223171691 0.6178772728499737 0.9942985189689963
0.8894511208124598 0.4508815495234373 0.9927392656497247
0.0558388096904778 0.4509466142001770 0.9929002227201522
0.3900017316221351 0.6192011186739705 0.0001099047853188
0.5573233232637094 0.6182718044686411 0.9941254626589071
0.2209467912528780 0.4503428849651741 0.9992635129082146
0.8895749129501525 0.2842952090631741 0.9927135956958892
0.0559865172722738 0.2842933914376715 0.9943909905346118
0.3898277682395701 0.4515472879608495 0.0098352160515134
0.7231437559650331 0.6177610765800239 0.9912433455115277
0.5574360542176464 0.4529331099699334 0.0012161076577257
0.2231345426221836 0.2846647234979685 0.0018212644007463
0.8898293720787507 0.1179964895450638 0.9918946554754342
0.7233571380554125 0.4511911446117338 0.9963136908669483
0.3886894859352665 0.2831076507150883 0.0142584180561940
0.0568219142536250 0.1179503892484703 0.9947060915263890
0.8897353064225939 0.9512318529599710 0.9903258664333450
0.5568685756049305 0.2837995930681387 0.0053264930690773
0.2227376644422208 0.1171616040375909 0.9992594817945317
0.0562394229781162 0.9510992978366701 0.9918529460485530
0.7230145498256846 0.2844173661408504 0.9960759882573598
0.3891253861288249 0.1168520114259337 0.9987266326555044
0.2228224790777489 0.9508354234614487 0.9930049025332366
0.8897463245665591 0.7844760603561554 0.9892267170711762
0.5562051691842669 0.1176559871620100 0.9959348253514477
0.3895581150324361 0.9509321283830658 0.9927522673847378
0.0560663735915128 0.7842690338779690 0.9902410859058506
0.7227094817032409 0.1176745172755744 0.9927491671025379
0.5562581480863358 0.9510881873734576 0.9920038234495792
0.2228875408135380 0.7844592023299484 0.9916260194851105
0.7232457120325080 0.9512427986107355 0.9903945576235458
0.3898062440275112 0.7848131339294326 0.9933284045712796
0.5563999930123842 0.7844445966318148 0.9918804322896762

0.7231941052621593 0.7847692471876115 0.9892285168700710
0.0004532826369470 0.6731048293816571 0.9903003668679915
0.1670469563671233 0.6729471763451826 0.9919359779122487
0.3335406808159576 0.6736271683332191 0.9960681497955406
0.0002450806331993 0.5063960663308364 0.9918962092779822
0.1664854745263042 0.5063423230631656 0.9949745802088046
0.6673111289509563 0.6731611860027797 0.9904465380605174
0.3325291021544530 0.5067277701608006 0.0054503744516907
0.0002921983387481 0.3396612900135736 0.9930259919756637
0.1666677118050899 0.3398833431839563 0.9979443145439788
0.5022004848799426 0.5074486652838104 0.0005691217334700
0.8340577929000403 0.6731474780829600 0.9899043562074752
0.6677882273656905 0.5071787458229196 0.9956505292563875
0.0004965571614690 0.1732476261337723 0.9935367015860095
0.3323343656879487 0.3391323916636253 0.0088699861762578
0.1670019276873944 0.1728335804876622 0.9989777270792928
0.5017937465288682 0.3406971012283577 0.0112717032129055
0.8335788636984347 0.5062524684031828 0.9928999845335014
0.3329493954251816 0.1713083763435819 0.0040242541570734
0.6677311429595534 0.3397743563038489 0.9996822597265975
0.0007500440866366 0.0064727497183341 0.9921651487321199
0.5003575281888430 0.1724398826386988 0.0002712982624615
0.1669892326558795 0.0061453490887118 0.9943045308356275
0.8339193498123620 0.3399164774672059 0.9936002385670690
0.0003920246377191 0.8395715076849086 0.9900084005980645
0.6670980733448886 0.1729156503049225 0.9951534257691961
0.3335763425264275 0.0061794364185417 0.9941130239662783
0.1670790828797593 0.8396182752500820 0.9912262333428643
0.8339362696220718 0.1731880476053007 0.9923440696710202
0.5002564616958267 0.0061590866080081 0.9933252247337094
0.3336987937024318 0.8399787753932557 0.9921968085645005
0.6671676002770681 0.0065582463674971 0.9916144996764160
0.5003228320086706 0.8397816475470847 0.9922891653991712
0.8339188501985646 0.0063860340809399 0.9906979510239537
0.6675078308767510 0.8399210033357818 0.9900606071272051
0.8339853044207786 0.8399917898229177 0.9891899283963639
0.5011779360759476 0.6741187157182793 0.9951816873346715
0.3864156949389610 0.5222271116219588 0.1186759185138158
0.4644366476851616 0.3048843549262514 0.1258657600963602
0.4154634668212154 0.5679387623503160 0.2559952140269743
0.5102033264757111 0.3288534870909601 0.2627703571102913
0.5530486735978286 0.5165563508854376 0.2710971457871736
0.3856871780108122 0.3886884618289059 0.2044075126644387

PT6(no-D)-IX – ΔE = 0.52 eV

1.00000000000000

14.7772044699999991 0.0000000000000000 0.0000000000000000

-7.3886022400000000 12.7974344700000007 0.0000000000000000

0.0000000000000000 0.0000000000000000 18.0009597799999987

C Pt

72 6

Direct

0.8872492805017487 0.6118003422453455 0.9935010009354102
0.0537711900185727 0.6122709754027937 0.9933087081574570
0.2203621240277229 0.6124175620437242 0.9964804808599368
0.8874391018211298 0.4453440983310770 0.9925760130426227
0.0534036632878028 0.4451982070035285 0.9962243094838570
0.3872244223363523 0.6136127771124649 0.0022846150790627
0.5523520806045639 0.6120572643943945 0.0096514811944033
0.2194652338929188 0.4446153307022769 0.0054995697432574
0.8874988123266050 0.2790714512222010 0.9933224341288280
0.0539224782813505 0.2782485780497836 0.9948904446368516
0.3876720733599797 0.4449331587045720 0.9962999685652889
0.7220163778937803 0.6120945094205226 0.0004573552589306
0.5537258413138275 0.4452530765281537 0.0046604198676974
0.2200165413668884 0.2776151435854857 0.9944121619622877
0.8868345317749231 0.1115742667526405 0.9944094873726854
0.7211471458067180 0.4455987324905166 0.9950201354234025
0.3857377899952823 0.2778518006138100 0.9997233775738081
0.0535416448163275 0.1118866134301655 0.9941287482889862
0.8872909892114720 0.9454441279466081 0.9976272034765614
0.5543812914649848 0.2778464662018862 0.0008495310004335
0.2203186863056601 0.1116884810504573 0.9943903778038958
0.0535251019208474 0.9451088199099971 0.9947543222967710
0.7203883582664545 0.2785122333006171 0.9934553785818636
0.3868890831649594 0.1115973408393174 0.9970296043083096
0.2204342074958632 0.9455846503058112 0.9938788243062149
0.8874648741192672 0.7791768300832871 0.9979949533839658
0.5541537241153804 0.1122004434481951 0.9951706654517380
0.3874765018889903 0.9456132150348040 0.9942423136962333
0.0540036226490699 0.7788287992063161 0.9934484276320532
0.7209031723413020 0.1122300701265786 0.9938846533504488
0.5539265056830800 0.9455829830461866 0.9948773504348054
0.2203137330125742 0.7786843065283620 0.9932679283823163
0.7204998780131930 0.9455585500784878 0.9972319403944070
0.3871544070385369 0.7792682102746511 0.9952188505681647
0.5541063276550915 0.7799344372043251 0.9996874995994034
0.7208497104575429 0.7793793099470463 0.0059709818414078
0.9982555510807103 0.6675795243775156 0.9930123926719432
0.1646343540317901 0.6679325149333550 0.9937833464516572
0.3312348766793392 0.6683423095596268 0.9972253239967088
0.9977968454755981 0.5008754186209856 0.9936771117161172
0.1642721264620164 0.5016063346209663 0.0000465269693422
0.6656781954657944 0.6679607194682902 0.0108673915073823
0.3319795552815776 0.5014161242801052 0.0061114451963107
0.9980561086099158 0.3343929292396055 0.9949498336096880
0.1635229792250357 0.3323978037220527 0.9974479514734185
0.4957619259952679 0.4998725253084544 0.9980720566615986
0.8315563731252738 0.6674516337825196 0.9968053450401015
0.6667852146262589 0.5013779776959737 0.9987221746133343
0.9980722090635155 0.1670444940157196 0.9939336828275316
0.3310625204039539 0.3330553426527203 0.9950787259411271
0.1644660202171053 0.1671360195343397 0.9938433746013260
0.4974290098979566 0.3331852698740008 0.0070763479001101
0.8317160943557127 0.5011055233690698 0.9929498172502704
0.3306188558121690 0.1668255460596981 0.9971348183743061

0.6646899946487181 0.3334798532755769 0.9958415987671714
0.9977640966881438 0.0005477281268327 0.9953233635805674
0.4982533914166964 0.1672651345304956 0.9978139577311822
0.1646520633471837 0.0005343756414362 0.9939607766902157
0.8318407970121697 0.3343379730795135 0.9926920212111270
0.9978754795690890 0.8342964035408968 0.9951186212318888
0.6647083053862488 0.1675427024030753 0.9940855758714378
0.3313642109024713 0.0011513128496006 0.9947892195076520
0.1643467271120187 0.8340763524422670 0.9931894928843121
0.8311720880872357 0.1672856228513240 0.9936170723551001
0.4978251914254557 0.0007701629489176 0.9944744506875907
0.3315161457696689 0.8344914536245653 0.9938478220587967
0.6648492379161581 0.0009501746656468 0.9950887073581569
0.4982400061381327 0.8348412025526386 0.9960802365391714
0.8314350275493894 0.0010213440071851 0.9966153508396545
0.6647036957532322 0.8347068236464352 0.0009188893226266
0.8318286580594076 0.8344645428714443 0.0009938850283575
0.4976251990700078 0.6690948576235272 0.0038530431757380
0.2706221083157985 0.4735406970639744 0.1209131900099436
0.4239025364202718 0.4758348124709642 0.1892637271499993
0.5497462457967330 0.4233059599543267 0.1221302805750071
0.5951034227926968 0.6180216918024044 0.1254426293556890
0.2758780634300706 0.4658890567248690 0.2581482957400496
0.5989149093273625 0.5215808439364338 0.2461689387696779

PT6(no-D)-X – ΔE = 0.53 eV

1.00000000000000

14.7772044699999991 0.0000000000000000 0.0000000000000000

-7.3886022400000000 12.7974344700000007 0.0000000000000000

0.0000000000000000 0.0000000000000000 18.0009597799999987

C Pt

72 6

Direct

0.8918846713824422 0.6100149687323935 0.9894636480836070
0.0582078150777221 0.6103059229637395 0.9906224917514805
0.2252522511592261 0.6110539656523670 0.9995408716049354
0.8924283725902811 0.4440521903795940 0.9891850585208388
0.0587975661751514 0.4435170404225275 0.9940159135448638
0.3925837925612399 0.6122972534646038 0.0166403086065827
0.5599517253282400 0.6122372462554111 0.0064002819244706
0.2233291652803544 0.4429630681890444 0.0081347068068496
0.8919683099425555 0.2771439204448072 0.9903887305535619
0.0589519208870755 0.2773917359891485 0.9966380738273415
0.3923253910724611 0.4418414668388166 0.0229766490018193
0.7258651989843443 0.6107762788564770 0.9949622244442011
0.5597682260529808 0.4429380180663642 0.0028496823567892
0.2252459796620485 0.2763572369470566 0.0037178268250244
0.8921169374227844 0.1102868772462244 0.9908424903656652
0.7251154195985876 0.4435477204957792 0.9912815063512852
0.3915531406326451 0.2765512188105532 0.0007188973456564
0.0584404092526114 0.1104944764869842 0.9934479415458100
0.8924155607159889 0.9442389311877122 0.9901483652958518
0.5590704984964248 0.2775066471596901 0.9926246368851892
0.2256962892805277 0.1107733893426044 0.9941184344978140
0.0587128349719990 0.9436566169148719 0.9900441831352680
0.7254489125436834 0.2767810838736366 0.9890725167031675
0.3921417850989926 0.1102370574796652 0.9914709706281997
0.2251278245567292 0.9439200234724225 0.9900428515372326
0.8916698116200550 0.7772093785411514 0.9899568591120484
0.5584045936998336 0.1103740924377377 0.9896706512063815
0.3923884773255040 0.9442504914980034 0.9908069532478123
0.0589419310702439 0.7774604138864660 0.9891621408985927
0.7256908782595133 0.1106685622883674 0.9893790872615966
0.5585299723911774 0.9431539523012731 0.9921045443096403
0.2253298079520363 0.7769943955062786 0.9919153679488488
0.7252108858849695 0.9438900325737123 0.9911003562763554
0.3916404931096409 0.7775419570955719 0.9976139412983684
0.5594351953123606 0.7775341535174007 0.9985087535206532
0.7255505779667999 0.7772005263766459 0.9937175504604596
0.0029175679610844 0.6655731739957673 0.9892035530343790
0.1700869201949402 0.6662558221724737 0.9933686934084065
0.3358007191039221 0.6669643108296128 0.0043606081883638
0.0028108471405730 0.4993482937602209 0.9906658628878731
0.1688231705117857 0.4985926867190642 0.0000284671309743
0.6701631917891149 0.6665898146787228 0.9977131468073281
0.3343849617397581 0.4989386192890564 0.0216873842379073
0.0032788111354591 0.3326740481891051 0.9935615522937340
0.1688505256387387 0.3321714657437909 0.0034993851879932
0.5052193565304961 0.4995501783352410 0.0168542319322853
0.8356798173377484 0.6657276903537337 0.9909641801390663
0.6699753387986078 0.4986805484935388 0.9957494693860269
0.0030754241763304 0.1655083632864915 0.9934752708108903
0.3358246240487617 0.3304723789116437 0.0089321717099509
0.1701626367710745 0.1662251173273717 0.9967343226242136
0.5037946647964588 0.3326912085261284 0.9986214821273478
0.8368309963833696 0.4995981595135746 0.9893755789404892
0.3361965162129650 0.1659737540189710 0.9948044897093951
0.6695969760773011 0.3325409119896960 0.9906903937191771
0.0029963280474306 0.9993630302119385 0.9907267492986946
0.5029851251871236 0.1654792139239234 0.9908880655288499
0.1697931302005387 0.9990969322294987 0.9908799853935548
0.8364764273445644 0.3322609519986131 0.9891847473503645
0.0032564787255822 0.8328846965760874 0.9891379752442830
0.6700414414510405 0.1661234457074201 0.9891354156284322
0.3366256456032062 0.9996504921818925 0.9902846665335900
0.1693891655078161 0.8325870424772717 0.9897856904122548
0.8362343158685803 0.1658158706478332 0.9900103650694305
0.5026310478266964 0.9991199278182208 0.9906027586085457
0.3362305181672909 0.8324036974578064 0.9930214713407324
0.6698323699313633 0.9988643448981520 0.9906329408605714
0.5034587803596438 0.8330968639886223 0.9962847958718299
0.8368424054644876 0.9995449443960496 0.9902835772008274
0.6698407663714931 0.8327125352967926 0.9940656418797928
0.8365269651388942 0.8324092100851388 0.9906624077007038
0.5049091081230728 0.6670010467007046 0.0067470379100314
0.5641386322268644 0.4372641570190865 0.2286329952550687

0.4135367985291865 0.6546535548040708 0.2599969429578834
0.3449868961104841 0.5615557168232854 0.1317890417076271
0.2625432261325500 0.4716547147121659 0.2576514834731185
0.4469645500554620 0.5001644239612588 0.2700207093932363
0.4613553650601503 0.4778637519392888 0.1338721468339443

PT6(no-D)-XI – ΔE = 0.54 eV
PT6
1.00000000000000
14.7772044699999991 0.0000000000000000 0.0000000000000000
-7.3886022400000000 12.7974344700000007 0.0000000000000000
0.0000000000000000 0.0000000000000000 18.0009597799999987
C Pt
72 6
Direct
0.8891494923233196 0.6122476160754928 0.9920361853175734
0.0564694863032571 0.6124285308140784 0.9901110138805436
0.2226453809380544 0.6119019478513223 0.9941470577973064
0.8892939937510107 0.4450883156447105 0.9914289105858103
0.0556217307227129 0.4455075098343002 0.9919544530369606
0.3887607770479065 0.6124929337576717 0.0041308081921017
0.5575005855283948 0.6142966330102837 0.0098424591969390
0.2226730535656287 0.4461625719629509 0.0010466887566878
0.8895970496560892 0.2791040224996024 0.9911825310258138
0.0558362074091150 0.2784323920725029 0.9948265918549239
0.3869772417868944 0.4453343609684097 0.0236051289397352
0.7230804428945703 0.6118979368202702 0.0000094428532122
0.5577383670175067 0.4453079068482637 0.0208276758744574
0.2208293129340220 0.2778960613274393 0.0054654456247007
0.8889221884303353 0.1121800703698668 0.9911205007765105
0.7235423517344728 0.4460357522067113 0.9990654527602629
0.3902574398753060 0.2777193114965044 0.0081125361626704
0.0560812480745412 0.1122907572513796 0.9940780886877221
0.8889988190218645 0.9449297685104909 0.9908285215522667
0.5557973073818943 0.2781652757639534 0.0012084326279265
0.2224184254152561 0.1115613612320132 0.9973668570606478
0.0556272448447714 0.9456654719270148 0.9912804713788361
0.7222014515671660 0.2791216096783273 0.9940197968196998
0.3893087619468432 0.1121450383005751 0.9959973658901973
0.2232215723373372 0.9458393685202040 0.9916030538262177
0.8896710099015692 0.7791560726468489 0.9912468191308648
0.5564016743856186 0.1123902010052973 0.9930776296806627
0.3893672000664310 0.9451443058289968 0.9916110854031359
0.0559661355089318 0.7784997951049348 0.9897718346196669
0.7224622582558098 0.1117867392042058 0.9911090184329296
0.5557599509339042 0.9455342968950191 0.9919720798047607
0.2225432917943024 0.7789830780295901 0.9906991533632805
0.7228883967371402 0.9455529754771490 0.9917237925201121
0.3897971772680719 0.7791828789607393 0.9940622983234633
0.5559579395455287 0.7784716612488296 0.9973882719414249
0.7222000870599175 0.7786932718135802 0.9949196717253059
0.0007386750234772 0.6682049329931772 0.9899451492563784
0.1668036429520470 0.6676563084423961 0.9910462514370906
0.3337624820062501 0.6673963867631159 0.9970866730591297
0.0005941675836283 0.5008684005537418 0.9905373603810546
0.1673025785857831 0.5014676459241940 0.9948682049921160
0.6670290299209398 0.6682576311617723 0.0011303557674864
0.3322008516473716 0.5016987082737216 0.0089496686693522
0.9999206399672005 0.3344475567765244 0.9922225413153640
0.1664560910259780 0.3338615724441851 0.0008273591665855
0.5024539310924396 0.5035946749452407 0.0253185794702873
0.8336330733147648 0.6672458003496686 0.9934961411024616
0.6688572575474296 0.5019296645127369 0.0059782531165880
0.0004467260245704 0.1679857103933458 0.9931991396219075
0.3323825176928636 0.3327571464258696 0.0173087117549997
0.1660555503063250 0.1671345882433783 0.9987470022586535
0.4999244249323596 0.3327961187325812 0.0096492366458207
0.8335789684466448 0.5011981884368524 0.9935672194421983
0.3342157246363868 0.1674746149518782 0.9998770645572677
0.6673603445849068 0.3340757424016374 0.9985954263847177
0.0003038856530182 0.0005491466035608 0.9915175637325788
0.5004857347098337 0.1678308109725606 0.9964361455158794
0.1675163551869758 0.0018453607907745 0.9930358506224124
0.8340043165879010 0.3349585287439893 0.9918847055105005
0.9999880365982463 0.8343430711890960 0.9901880849004741
0.6665259346785462 0.1676090902901066 0.9925529203403372
0.3333672664331431 0.0009214794390218 0.9925304203365783
0.1672659278636104 0.8342646295757135 0.9901048978747440
0.8337125518645152 0.1674432586870296 0.9909870111479933
0.5003840492675096 0.0007182929693457 0.9918141363482107
0.3339417981032611 0.8346576848361380 0.9916342845094661
0.6671523162017436 0.0013617078044348 0.9914294869664459
0.4999253346857770 0.8343331915387040 0.9941900152942367
0.8332097825012639 0.0008370250390968 0.9909189682750821
0.6670674022268486 0.8341482798916644 0.9945620631338841
0.8339345426689064 0.8346329995979644 0.9916822854171912
0.5009948963975503 0.6687373072205247 0.0044019285849615
0.5397184707589062 0.4807392164063131 0.1372730745572923
0.5189830584124451 0.5596301657958165 0.2609039407107261
0.5389244014247510 0.3990393569550008 0.2669511568676484
0.3533475181979711 0.3777583023141489 0.2727081216494582
0.6980086539867898 0.5765036620058126 0.2276663358332982
0.3417145189630517 0.3740741615333150 0.1334657835410837

PT6(no-D)-XII – ΔE = 0.55 eV
1.00000000000000
14.7772044699999991 0.0000000000000000 0.0000000000000000
-7.3886022400000000 12.7974344700000007 0.0000000000000000
0.0000000000000000 0.0000000000000000 18.0009597799999987
C Pt
72 6
Direct
0.2241325414308653 0.2862676380124417 0.0031342800308700
0.0590501550394674 0.1202165776898728 0.9936284888549907
0.8923851179063985 0.9533781404038698 0.9922509624965737
0.0592405131450325 0.2868040234151371 0.9917442317026981

0.8924300954494327 0.1201877235887012 0.9906944836635506
0.7259214285386584 0.7867323018250565 0.9957431907216699
0.5593795815119293 0.6204572063761802 0.0032523489748432
0.7258239378704117 0.9531389956471248 0.9921767820380348
0.8927276772560429 0.2868849030588662 0.9898117137113089
0.7257810917667303 0.1199635112450779 0.9914990376206678
0.5588845994529895 0.7864592977006757 0.9951747117651113
0.3918793924502213 0.4551363426884020 0.0164658423892505
0.3922755677182395 0.6207450806240388 0.9980236282328189
0.5593012727034040 0.9535669756561781 0.9924901985143084
0.7259118357010000 0.2870413682483310 0.9942391045010679
0.2259662018032067 0.4540481409051930 0.9962771249237302
0.3928080890159790 0.7872356348670568 0.9923444230225655
0.5596839239654228 0.1201402450533013 0.9954417375838760
0.5600787812281544 0.2860342914585693 0.0060236477867151
0.2259324019808264 0.6204174458693212 0.9913480809112609
0.3928001703320159 0.9537331350142328 0.9936630984936505
0.3929435661848266 0.1202860075654390 0.0001423205829816
0.0592297917004331 0.4536997082400983 0.9896879471516946
0.2261390970971413 0.7874126629921404 0.9913567324898792
0.2262162761950967 0.9539550634968869 0.9945276333362555
0.3914679623665418 0.2848706550416908 0.0121503368842897
0.0595419358790730 0.6204982403963413 0.9901832288090957
0.0593072387422769 0.7871584023681095 0.9919991308803954
0.2253378017121390 0.1200247714057790 0.9993905403572078
0.8925966816696613 0.4534274726923371 0.9911505274421909
0.8928911212995204 0.6206932603302052 0.9935838775921049
0.0592140406802621 0.9539562064380540 0.9934438770879336
0.7268834766519562 0.4540055404419600 0.9997146772507506
0.8929614039924051 0.7872353583365750 0.9936982950744223
0.7266251091036864 0.6206624307372580 0.0005847637255665
0.5611329488706208 0.4554819094624522 0.0177139623827277
0.1698580633140921 0.1759535680325844 0.9985147282997437
0.0039194398566919 0.0094764488246980 0.9927620927019947
0.8374313322867979 0.8429690513582099 0.9937829053981257
0.0038456812009339 0.1762742646201174 0.9917503103459443
0.8370725660959014 0.0092753647503882 0.9915443107585631
0.5050122997105114 0.5118784624253395 0.0119638709143559
0.6706205212429025 0.8427410043708686 0.9943424920736583
0.8373787753399842 0.1761853138083509 0.9903710076374624
0.6704964466457475 0.0095296979231848 0.9919041170497307
0.5036881600578444 0.6766813492240390 0.9989571687866430
0.3351170345806900 0.3416567382602392 0.0166942244144752
0.3362469209680446 0.5104511258810405 0.0027726907696355
0.6705342221349417 0.1759904698670376 0.9936027741703517
0.5036177101182671 0.8427456373421470 0.9930778037121245
0.5040650272268671 0.0094551450304213 0.9935664806283175
0.3372019809237274 0.6764900697719132 0.9931468001710236
0.1702646648882984 0.3426627208292382 0.9964456309860368
0.3373531322172449 0.8433222109079554 0.9920029093306114
0.1707935625617601 0.5098886734832192 0.9914953963618718
0.5042942106814838 0.1760269281033932 0.0005810871482772
0.1705939296417936 0.6764982450980610 0.9903971932253334
0.3375291593149412 0.0099768176636874 0.9958140424603116
0.0038680224767334 0.3429833391922961 0.9897858872786686
0.3372543106971363 0.1762676156230890 0.0035254222486491
0.0041836307531905 0.5097928309823587 0.9896774881931529
0.1710290361995289 0.8435549212177111 0.9922344623446864
0.1707625502071674 0.0102399892191158 0.9955469797893504
0.8375043977914338 0.3432502144149774 0.9911965635970980
0.0041657083871341 0.6767218978986425 0.9914575821421516
0.0040846356234584 0.8431309513099629 0.9927172416507476
0.8372139573449212 0.5096344272896602 0.9942030200849309
0.8378996292679539 0.6765480309366733 0.9954748413172181
0.6711984366366814 0.3426734959857848 0.9997094355102213
0.6724103494848558 0.5106555975666668 0.0060594391259529
0.5039895372425425 0.3412366595613309 0.0177122596056885
0.6710351028036896 0.6764184870553756 0.0000320585723857
0.5905477212471424 0.4941710522569088 0.2591521784947465
0.5418483656906314 0.3979778602638975 0.1318507225698665
0.2352609530219083 0.4021883086109296 0.2215058268573241
0.4896386020122303 0.2981844534714023 0.2579008465103889
0.3903944902393529 0.3991430479769909 0.2674967324390662
0.3669041784241314 0.3991643422073164 0.1314610529706202

PT6(no-D)-XIII – ΔE = 0.60 eV

1.00000000000000
14.7772044699999991 0.0000000000000000 0.0000000000000000
-7.3886022400000000 12.7974344700000007 0.0000000000000000
0.0000000000000000 0.0000000000000000 18.0009597799999987
C Pt
72 6
Direct
0.8918076685822669 0.6151830816926278 0.9920390234743692
0.0587529112703393 0.6159270167638482 0.9919541295211758
0.2259149777596221 0.6157819209844959 0.9953270594761037
0.8926544449669365 0.4490678062018176 0.9916528660741974
0.0584862129402453 0.4485870286498894 0.9942390776156458
0.3915977184240731 0.6155600637465923 0.0013896800985265
0.5597243339144242 0.6175260111139735 0.0042629900350164
0.2252436748479454 0.4496706879192374 0.0040185980452279
0.8921702601328789 0.2826701016943716 0.9914961393796702
0.0592107018499917 0.2824250633820213 0.9964161269547276
0.3922350313043026 0.4509909300146049 0.0236222530133006
0.7261786208543057 0.6159799401631574 0.9968611078053087
0.5599206512088628 0.4484168102657193 0.0071423384333258
0.2233043100771752 0.2809450757083667 0.0075131470008003
0.8919696453188664 0.1154346211850807 0.9907684801317984
0.7256021056597888 0.4492053192117851 0.9952288876018685
0.3926715089223194 0.2814335408037323 0.0161222372395713
0.0587374881358613 0.1160675448171276 0.9942131131439993
0.8926531685563219 0.9492539365608152 0.9902426102898332
0.5596811553297627 0.2827686739232220 0.9992331565805515
0.2255323410826549 0.1151955184419649 0.0005909086872862
0.0583268058685746 0.9485923886698515 0.9922361854479576
0.7253014028948073 0.2819736607084948 0.9923331228270200

0.3917759232207203 0.1147670280818076 0.9990735728210751
0.2253950435958600 0.9492185368414283 0.9938956648727668
0.8919884395991673 0.7827488257928081 0.9913493808906253
0.5586309680738566 0.1162026631485418 0.9935224310996134
0.3926599577556544 0.9490060357617480 0.9926370329500571
0.0592288402034953 0.7824251432218432 0.9913923997361991
0.7259244156792874 0.1158770294269900 0.9904084293673421
0.5584818411538350 0.9486504489765295 0.9910758827371282
0.2252249486042288 0.7818988587800533 0.9920574678538188
0.7254510973191444 0.9494538557428882 0.9901212725802893
0.3921799973655453 0.7827117003818316 0.9928746597971809
0.5592513571593827 0.7823623651353486 0.9938056844803995
0.7251961898447803 0.7822064348952722 0.9926502402156743
0.0035891799282268 0.6711734483359280 0.9913164291658774
0.1701567641589889 0.6716798278097400 0.9926680029124100
0.3362957767560317 0.6709462646865205 0.9958174064898699
0.0030971030672546 0.5044081551908732 0.9921831496875768
0.1704069786460281 0.5046301511350819 0.9975309818129219
0.6702982235744201 0.6721976292920502 0.9973698288339818
0.3360927800525886 0.5061151005334423 0.0092819188512365
0.0034128345770696 0.3385218133951966 0.9937852031035703
0.1690128992380266 0.3373684731016127 0.0031817124786642
0.5050899362590044 0.5060893125825743 0.0179969482367284
0.8359223208378452 0.6708202485011014 0.9930266322367203
0.6704769799183481 0.5043832552821428 0.9992380259236384
0.0035805261563766 0.1713328635285478 0.9934850365525634
0.3344921212019116 0.3357490827449681 0.0219300512511538
0.1692539190975566 0.1713872577536435 0.0001491291687117
0.5052186904154112 0.3389618924674167 0.0066843728251058
0.8368853921535404 0.5051166900421151 0.9924433879811687
0.3358631219554553 0.1696459957576479 0.0051301343703045
0.6699485508024665 0.3378806941053654 0.9952727725251194
0.0029127935431461 0.0042622629687799 0.9916552935547429
0.5037740369128940 0.1711174262857540 0.9975127830453587
0.1703121204240929 0.0045541913967782 0.9950463228221196
0.8371052301132025 0.3380356160426246 0.9914448592501088
0.0033216568335988 0.8383606637122796 0.9912051638181225
0.6701836340540979 0.1719048311094156 0.9918403555473674
0.3366267515834878 0.0048168847678625 0.9949014425110079
0.1695524594948168 0.8376104536072049 0.9919614814590858
0.8363408821973550 0.1711537475679563 0.9906464003806192
0.5027932171647649 0.0042730713831247 0.9922063202019302
0.3369289784994223 0.8377422946858388 0.9920887639877165
0.6703272829938314 0.0046369930651053 0.9902777775071101
0.5033123194618767 0.8384333397808277 0.9921995440252118
0.8368165002654209 0.0051652389692407 0.9899659079291041
0.6696792256181325 0.8377777249370766 0.9919223107264301
0.8370923098985301 0.8379780201647264 0.9908411257462717
0.5039794326529332 0.6717557751430903 0.9996836319003890
0.5715538026432085 0.6453200704861004 0.2155357714429158
0.4571223216698073 0.4871929159988966 0.1357880530658377
0.3536050765876837 0.2885665556356898 0.1323270796844795
0.3422330961983135 0.2793814152639484 0.2698085079535701
0.4337646234100632 0.4696271734270923 0.2741524064396259
0.4475715757836127 0.6390231897230407 0.3102793982643135

PT6(no-D)-XIV – ΔE = 0.61 eV
1.00000000000000
14.7772044699999991 0.0000000000000000 0.0000000000000000
-7.3886022400000000 12.7974344700000007 0.0000000000000000
0.0000000000000000 0.0000000000000000 18.0009597799999987
C Pt
72 6
Direct
0.8988551395141826 0.6210834585024045 0.9882758753181236
0.0653835726235812 0.6211180046346882 0.9905391832055628
0.2318144618468168 0.6214173882383065 0.9968091160136083
0.8985845816219324 0.4541970870004448 0.9888828340076898
0.0651487220256683 0.4545881251497121 0.9963707980282308
0.3988483937069844 0.6210158096333345 0.9996948461608497
0.5660211096796033 0.6212124545974120 0.9962315129458759
0.2318957129320083 0.4559920527859944 0.0146490572752285
0.8987207162315158 0.2875167552883653 0.9915168795195513
0.0637658235672944 0.2868258906985091 0.0034779807817031
0.4010505231472621 0.4564124833937342 0.0164599553621230
0.7322494626525540 0.6211846922338040 0.9900589590172473
0.5660505898118160 0.4546586496257135 0.9966781673494012
0.2311910310567953 0.2854344610615414 0.0114175370986658
0.8984118044080347 0.1207558023389623 0.9929242547362094
0.7320215595961699 0.4541473006663850 0.9888829433676847
0.3996295464654267 0.2866416392080779 0.0044553052095537
0.0648559443315051 0.1206765826209235 0.9987372003259765
0.8986689713057672 0.9544642119750293 0.9915190188706688
0.5652455633510982 0.2875881644822442 0.9919166138819122
0.2323851687792384 0.1207424784639173 0.9991521306656495
0.0655756386687543 0.9545081945820399 0.9930812576316939
0.7320340512429837 0.2875196798448769 0.9882479365906960
0.3990725315214263 0.1207737447311956 0.9933690377931512
0.2322961343594159 0.9544166111967627 0.9920584423322936
0.8988259710158246 0.7878427545087092 0.9893963483101373
0.5653240318702615 0.1207057044331563 0.9891731229893566
0.3987856880138452 0.9541880294245075 0.9901187625112087
0.0656018154920304 0.7879499481477055 0.9897607412168057
0.7319392478175928 0.1207571220283796 0.9889665038666919
0.5652239514764403 0.9537558435849789 0.9893263814682385
0.2321832296766999 0.7878531953716461 0.9907338309264375
0.7318596628094198 0.9540332289735716 0.9896323965819889
0.3983730352306978 0.7871965169958131 0.9923357714776344
0.5653024721906021 0.7871989105112220 0.9920183316832620
0.7323113381423738 0.7877877478956563 0.9900121799652695
0.0101972614168844 0.6772888651434883 0.9889906107538522
0.1766297406030120 0.6772597659770767 0.9920836873021628
0.3431468800864224 0.6773055357745861 0.9962741015305170
0.0101802791564154 0.5104759537220360 0.9911624798687910
0.1759557062206980 0.5112494940147343 0.0022672626082993
0.6772855401771238 0.6772056107227442 0.9913166895086434

0.3448340029751193 0.5127369641793891 0.0090588169881940
0.0095865775544368 0.3433902888433025 0.9967566704371791
0.1750119134732202 0.3423023625771933 0.0156664218429441
0.5120055482294319 0.5114386427429380 0.0024686581612983
0.8436520716016744 0.6773371440526006 0.9886811256199977
0.6766690713281065 0.5104013088153252 0.9910645933429549
0.0094285884873528 0.1766029899720891 0.9985050125896890
0.3439071123835760 0.3420007216780760 0.0178881466174019
0.1768630003344711 0.1769760067635318 0.0027724114172116
0.5103906719965110 0.3433332668909301 0.9974129304867887
0.8435387539096837 0.5105172605129269 0.9880254083113584
0.3438524823190647 0.1767685229892066 0.9990001886863880
0.6768855917844121 0.3438675535310267 0.9891162954371850
0.0101935726384212 0.0107913338200305 0.9942644142455634
0.5100633826038745 0.1768017269135953 0.9913349885516851
0.1771048859124775 0.0107630164008086 0.9946157291323701
0.8434272487012464 0.3438258711615916 0.9889808576203620
0.0104941882094494 0.8442740693928954 0.9903393036510337
0.6768104579369165 0.1769599290460775 0.9884773486274696
0.3435262936164136 0.0102153079920981 0.9915552637195830
0.1768285207617453 0.8439969324470411 0.9904363971377421
0.8433097788551365 0.1768823427200346 0.9909349867921549
0.5100048496844281 0.0102038337191033 0.9893180207532524
0.3431989214917266 0.8435527075244309 0.9908452036096449
0.6766979242148139 0.0100935018479547 0.9890921816310367
0.5100614758138420 0.8434836783193518 0.9910723202362632
0.8433791803644510 0.0101693720106013 0.9910072231580713
0.6768890751896777 0.8435798951967399 0.9902713053822012
0.8436371452397253 0.8438505442078892 0.9898500886475645
0.5105988600624300 0.6772152256774291 0.9960121464400018
0.3748169206707175 0.4035373129229143 0.1319202356263744
0.4236895390172890 0.5145610272204308 0.2534446846711518
0.3269665529484627 0.3165575252431552 0.2626814990243602
0.2014915422159476 0.4027377806924903 0.1295707448439174
0.2219884451388836 0.4142122001830515 0.2676263269407855
0.5206707668737778 0.4141256294947482 0.2194186057692704

PT6(no-D)-XV – ΔE = 0.61 eV

1.00000000000000
14.7772044699999991 0.0000000000000000 0.0000000000000000
-7.3886022400000000 12.7974344700000007 0.0000000000000000
0.0000000000000000 0.0000000000000000 18.0009597799999987
C Pt
72 6
Direct
0.8877280438495774 0.6138844822521392 0.9973015698287924
0.0545282703551280 0.6138080142054179 0.9910450685738823
0.2208261438037695 0.6136976286289482 0.9899204232475114
0.8874431131287395 0.4470474261170168 0.9950099423341427
0.0541189989070858 0.4473712578189364 0.9936480136896293
0.3878945201281994 0.6148644310807398 0.9967739607508577
0.5546689364022228 0.6169888484753869 0.0182177570140922
0.2206173096153563 0.4471057744030134 0.9971687035979855
0.8877779314093814 0.2807556642843636 0.9943783423750574
0.0538917754429846 0.2805525928846819 0.9987147248416548
0.3861963777216459 0.4480997018359645 0.0067881177881759
0.7220739932534812 0.6131518383419277 0.0087027473985444
0.5539543137784122 0.4458949662104530 0.0174315382611141
0.2196754387193351 0.2798579686834088 0.0084496309094320
0.8877520884938122 0.1143806186451570 0.9954794242862377
0.7213204163956632 0.4472835810581373 0.0022202571218912
0.3887521863814598 0.2794832264188898 0.9990471506126397
0.0543945540635278 0.1137727666086050 0.9974625913671602
0.8876018479621237 0.9472978800313854 0.9964594571108734
0.5542058319523733 0.2801382114242301 0.9964958548035057
0.2203830474369255 0.1132138258718030 0.9933052840016998
0.0540066932382501 0.9473813596412128 0.9927697812676328
0.7208438460027935 0.2809626672320675 0.9945062797939954
0.3877992757340820 0.1141312396594785 0.9886392125434256
0.2212302502760508 0.9472099644237844 0.9872890041225446
0.8880721195131045 0.7806973243237465 0.9981570802041304
0.5547721827203489 0.1141458330562415 0.9889237173688699
0.3875247506626138 0.9470749745800333 0.9858498063443051
0.0540307964137838 0.7802003674268434 0.9910099039920013
0.7208868372149624 0.1139563583948089 0.9917739370489045
0.5545254892416551 0.9478450981533868 0.9887574831239050
0.2209732014027992 0.7807035986337922 0.9869942427347880
0.7214537650676860 0.9476567378700835 0.9946586766403485
0.3879668586924012 0.7807042501090464 0.9883951057955471
0.5538789770716832 0.7810547815043165 0.9962694694800049
0.7219800761281618 0.7824234908178838 0.0040665625516922
0.9988026107615866 0.6695094543794227 0.9926978467548793
0.1653034506105726 0.6690468094526238 0.9886944951680761
0.3324749772171387 0.6695656273284456 0.9907044132985234
0.9989041386998636 0.5027636839530274 0.9929439130646358
0.1653009385226483 0.5027398156537188 0.9929524129375764
0.6670281807331477 0.6707570284372437 0.0146633725718530
0.3317178584558391 0.5026273747264085 0.9992253305163388
0.9983068499993735 0.3360619980381472 0.9954963913367507
0.1647431023146595 0.3363980554666171 0.0024542797965736
0.4972373911617538 0.5022300499468031 0.0206877351801680
0.8322371766675971 0.6688543908353139 0.0009122640660664
0.6662663501216315 0.5027126867900975 0.0102467576082361
0.9986665317957204 0.1696501094000098 0.9978329474375798
0.3321419915561137 0.3355511241921150 0.0088010085480335
0.1643606406701323 0.1678536119015774 0.9998230842678737
0.4985208311377747 0.3347632760137955 0.0024791282454260
0.8319216307699548 0.5026057398988542 0.9980476497578579
0.3326385269623771 0.1691676107787714 0.9922219287843319
0.6657351124430022 0.3357998577430976 0.9981846131585996
0.9989313922180258 0.0024519310537485 0.9950858987953950
0.4988295321868890 0.1696066693005704 0.9903524276147024
0.1653038785644156 0.0029693652828513 0.9901927408943862
0.8321089066065070 0.3364423485799151 0.9946244389034433
0.9980763507460608 0.8355254490708290 0.9935380671441862
0.6651208552882224 0.1692648888704369 0.9913365041371236

0.3316961871143675 0.0023455467709255 0.9863517966421682
0.1654514097223014 0.8359135134155622 0.9877439820698655
0.8322635082964638 0.1694862603125813 0.9940280685849032
0.4989942670847825 0.0027622605090158 0.9870334550007058
0.3320335949804800 0.8361967387367173 0.9862679287882159
0.6654521459901446 0.0031723855419514 0.9913072467908393
0.4985835247843724 0.8360727319977670 0.9899945726369310
0.8319875349565251 0.0027449891687326 0.9956145252511348
0.6658836851063370 0.8366813373892157 0.9987021230018485
0.8327043300526213 0.8368305509964173 0.9994742801311389
0.4981293751907216 0.6709766320579149 0.0024269762199580
0.3983689727670168 0.3158357797332769 0.2407261966220204
0.6214940106650815 0.6651045347673303 0.1303321646211231
0.4990612643059222 0.4677165398525958 0.1373700540648954
0.6190134780528922 0.6834337024497046 0.2671502046330119
0.4744481346767628 0.5034401978580121 0.2696312437615873
0.3028086294559102 0.3098894224080908 0.1236493110740327

PT6(no-D)-XVI – ΔE = 0.65 eV

1.00000000000000
14.7772044699999991 0.0000000000000000 0.0000000000000000
-7.3886022400000000 12.7974344700000007 0.0000000000000000
0.0000000000000000 0.0000000000000000 18.0009597799999987
C Pt
72 6
Direct
0.8886601148861999 0.6122837944023800 0.9880207902239562
0.0547454627897253 0.6116275829623135 0.9887346788622366
0.2214231948366958 0.6122648057674454 0.9923706221390773
0.8881777120010881 0.4455501267180324 0.9903929265173730
0.0554971137018327 0.4456035868187271 0.9907415346818027
0.3886677476784897 0.6128179948608548 0.9996206692483085
0.5559748041760031 0.6136008177923102 0.0113218972159643
0.2215411063198189 0.4450121122739716 0.9949273622658694
0.8883294442412009 0.2783627108099651 0.9926112161330423
0.0547879105743547 0.2789016687113062 0.9924978095775359
0.3867738668855836 0.4449913255026985 0.0045371937990666
0.7216933091847579 0.6126317139008890 0.9941763696038279
0.5569058220396172 0.4464035869315737 0.0048494430305297
0.2215192206527021 0.2787274732921645 0.9964922821191493
0.8885967891646658 0.1122745647966781 0.9935813559251443
0.7216799095293638 0.4449559613104555 0.9943268374437295
0.3867852838778276 0.2777047133615298 0.0048405503933466
0.0547731100194255 0.1118156238673649 0.9926509967786927
0.8883266683728337 0.9459064178066257 0.9926484170068832
0.5560050370894629 0.2782978834702874 0.0117790545644993
0.2215377916901318 0.1124674047489407 0.9953939347928582
0.0554876046467854 0.9458185484337633 0.9910395632058453
0.7226613356340081 0.2791713255123796 0.9977106819819426
0.3886449519533670 0.1117585420048215 0.0002358640257611
0.2213895251882221 0.9450808476294341 0.9928500607960657
0.8881702939147189 0.7785540715401709 0.9904209822451335
0.5548478333348967 0.1112726908032755 0.0008102507269641
0.3882348761278607 0.9455809381202585 0.9963420571369213
0.0547408943825616 0.7790507424870441 0.9888813457928478
0.7214533354628188 0.1123704402842449 0.9968632804068207
0.5554471212360639 0.9456805685911647 0.9976770875055649
0.2220930366764122 0.7790158851638509 0.9914953985618595
0.7214450681635967 0.9450004946685553 0.9967572074545856
0.3882438835841953 0.7786017933349214 0.9960316267865821
0.5548576934030862 0.7794893582487674 0.0003769025359901
0.7226513230809886 0.7794010651455281 0.9974542920304259
0.9987155472438261 0.6673179595836913 0.9879384882278899
0.1662731421184773 0.6674284050454062 0.9903907951926456
0.3329452150813026 0.6679166549442215 0.9955092167247273
0.9995791126267974 0.5013917718250340 0.9894145715694762
0.1656437953930308 0.5007934291657037 0.9922377487858611
0.6677809134534272 0.6685785495390775 0.0008633948275971
0.3325200653963591 0.5002756652377016 0.9995136355655276
0.9996191754571626 0.3341549531805299 0.9915394968248208
0.1660256040074160 0.3345107871813298 0.9943329051480632
0.4994578398654568 0.5010393893511136 0.0124366151892517
0.8326889798786823 0.6680265642893062 0.9897683382649376
0.6654090047860066 0.5006532789246734 0.9959004283795849
0.9989407693113250 0.1674424085046979 0.9927095215901502
0.3316365209661640 0.3337656506901752 0.0016060125961701
0.1660096867636156 0.1674421625187321 0.9945778820975164
0.4994841724014876 0.3343516711867025 0.0127810915457900
0.8326815373319079 0.5005686387985975 0.9897899093722415
0.3325040332479929 0.1681571100430048 0.0000261077371491
0.6677926999513204 0.3351104294746321 0.0011698489122196
0.9996025339139933 0.0013867262647480 0.9917184151861278
0.4984080671357773 0.1657714113548678 0.0046011029851911
0.1656251743814110 0.0007791552384262 0.9927212142655648
0.8325882255448818 0.3341915121569912 0.9931241136577214
0.9995747194402043 0.8341121330178396 0.9895908614333564
0.6662975572890275 0.1672605273267891 0.9990967636399759
0.3329211590372338 0.0009553399722293 0.9960476977249257
0.1662614149872255 0.8347721365155110 0.9906322569485937
0.8329423244926915 0.1680371045357560 0.9943785973591588
0.4992917316676895 0.0009385954765975 0.9982017177862730
0.3322058327695743 0.8340750027114581 0.9943323310735011
0.6654822439203372 0.0006969754834785 0.9973895495391929
0.4992984091195751 0.8342737845103798 0.9979252336030697
0.8329385598328400 0.0008523250629153 0.9943616728519231
0.6662942752293546 0.8349433643948458 0.9988061805611750
0.8325826159541450 0.8343226441515057 0.9930612325683228
0.4984159465856663 0.6685352146733194 0.0040391605172587
0.5311067730026338 0.5609978679932937 0.1266078269626547
0.5458624378743195 0.5784001267059438 0.2668407147829654
0.4382688259693950 0.3875662761854741 0.2155641114374802
0.6330664862986666 0.4844150872313122 0.2009331241285182
0.5445810297058742 0.3034957189240615 0.2674035408474467
0.5309685144804277 0.3058397151464050 0.1270124236095214

PT6(no-D)-XVII – ΔE = 0.68eV

1.00000000000000
14.7772044699999991 0.0000000000000000 0.0000000000000000
-7.3886022400000000 12.7974344700000007 0.0000000000000000
0.0000000000000000 0.0000000000000000 18.0009597799999987
C Pt
72 6
Direct
0.8857685925838936 0.6072818367850488 0.9939293571647383
0.0525046375698253 0.6075207827800284 0.9941707131041255
0.2187451190656233 0.6072868774649578 0.0017126092805100
0.8859765858042650 0.4405146168664444 0.9947056649903132
0.0518782929985164 0.4404925486851781 0.9971445297440695
0.3845507852598899 0.6086012696559493 0.0158456069716095
0.5544824508505926 0.6085441937751952 0.0147728906674303
0.2169341858153402 0.4401210605844099 0.0090542536685518
0.8858880446554878 0.2742405398572032 0.9935535923710646
0.0525422941533407 0.2740779576732493 0.9948708372312751
0.3838810654345011 0.4387659509543909 0.0225786550045370
0.7198192465485320 0.6074757639282709 0.0011969057684382
0.5547800157553127 0.4402883224075040 0.0172078444371238
0.2187132556087903 0.2735012905950427 0.9985751627738892
0.8858591592582599 0.1075286443897028 0.9906019981784411
0.7201732126885503 0.4409190170468094 0.0008411631951333
0.3855363778259786 0.2737243969649583 0.0034074812613767
0.0525154834457098 0.1077024636723891 0.9895674905076888
0.8858492598551635 0.9406854247722549 0.9896279478018215
0.5527023785924143 0.2736081598534597 0.0037314521320297
0.2193658526560256 0.1074843683888034 0.9918746589454344
0.0524121921639917 0.9407995398072870 0.9882424681438806
0.7189933244556386 0.2739551259432034 0.9968162007454211
0.3857658854447052 0.1073127897737862 0.9961669587165716
0.2193650359463462 0.9411129175848245 0.9909839100122539
0.8859396334031331 0.7742460152776758 0.9910294677023117
0.5526927660296934 0.1077728480992448 0.9965948717329951
0.3859708472287977 0.9408735494432037 0.9955067655270255
0.0525444972741198 0.7739878551567756 0.9904034829651565
0.7192382620985001 0.1072949368118401 0.9941212891234628
0.5523821333435492 0.9412145722786605 0.9987601751788020
0.2188924470064535 0.7740372701090550 0.9943845763439967
0.7197768441843095 0.9413558953772991 0.9949133874580482
0.3855557237343206 0.7747787317320629 0.0019964413185320
0.5538443655964969 0.7759744721473538 0.0102558364465963
0.7193972872731607 0.7741577773455148 0.9973999042410711
0.9972638478510873 0.6629094926431023 0.9921951421021333
0.1636281348625559 0.6628540691770652 0.9961469665682330
0.3299207684363452 0.6627756150651152 0.0060740340777201
0.9969450784527751 0.4960935793879884 0.9948897850552640
0.1629001807804045 0.4960237274122505 0.0028299535523928
0.6635427446295381 0.6627566419479436 0.0035078134542630
0.3281529990943497 0.4964681394536044 0.0206210677391212
0.9967811682666010 0.3295866463829782 0.9951356237816711
0.1627681924838100 0.3289905420006818 0.0007769654163567
0.4992081911823547 0.4967923643657244 0.0238034501947979
0.8304044315750048 0.6626963849792560 0.9946074546052159
0.6660833661207164 0.4968385410398355 0.0068337582065112
0.9972565112608720 0.1628102572986663 0.9908085629356478
0.3295119430904023 0.3279080451833210 0.0060942867649487
0.1637808237105247 0.1631329501399321 0.9920665817383707
0.4968788588439779 0.3281168125420848 0.0102805024277188
0.8306949642693056 0.4963711621050422 0.9959603276139717
0.3303821722179947 0.1627426429496026 0.9962601577073187
0.6641161674362905 0.3293111630104164 0.0009356961506555
0.9971103610720959 0.9960878590029978 0.9882774788633810
0.4972344504970323 0.1626846775569533 0.9986816668477445
0.1640800601070183 0.9964129122972381 0.9895216822322652
0.8306838527680185 0.3296017330165526 0.9948737011689559
0.9969967582990549 0.8296263590467774 0.9890906473298671
0.6636995124844915 0.1629274003673871 0.9959886048818376
0.3303279561187438 0.9964114141509910 0.9936492674596664
0.1637880710455661 0.8293502412730405 0.9912461760642515
0.8304613061136763 0.1626670803411727 0.9924717446791647
0.4969009258004533 0.9962342389500591 0.9965944839575798
0.3304722376196310 0.8296317121404897 0.9966537721534792
0.6640720185328561 0.9963499890177943 0.9957127936766383
0.4964187159212585 0.8307107044432911 0.0042804546137205
0.8305892368380086 0.9963262219588955 0.9909513809038231
0.6653427277293531 0.8302484046069623 0.0005098166593172
0.8304429780058697 0.8293722257321932 0.9916821479112556
0.4973950863230030 0.6638195000824609 0.0190945732709764
0.3350522448430269 0.4374714385631080 0.1336208577583591
0.4916278351435679 0.5864487624999910 0.2044060631712767
0.5184092342460289 0.4452557345755608 0.1346958408815908
0.4090484197365569 0.3998906349443700 0.2538876508232164
0.5429376820865244 0.7534907250783931 0.2663217743948678
0.5333860777336383 0.7466826719600377 0.1284172439718674

PT6(no-D)-XVIII – ΔE = 0.68 eV
1.00000000000000
14.7772044699999991 0.0000000000000000 0.0000000000000000
-7.3886022400000000 12.7974344700000007 0.0000000000000000
0.0000000000000000 0.0000000000000000 18.0009597799999987
C Pt
72 6
Direct
0.8930849395386034 0.6140681251780862 0.9950884094692043
0.0596691341022435 0.6144941518465430 0.9975475799822888
0.2266920045945611 0.6150090050770700 0.0019747406860589
0.8931662046024158 0.4474963455915457 0.9977319189260356
0.0589795950417766 0.4472949092851408 0.0013964563568649
0.3934730229281556 0.6151560657823154 0.0136146102477639
0.5613289683655580 0.6142471012132162 0.0004182478886889
0.2242757073807695 0.4458467881371249 0.0063674548100039
0.8931271757367512 0.2810959198041232 0.9967642724300561
0.0587256711424118 0.2799013288222412 0.9964946312889467
0.3932190025960125 0.4471748980115962 0.9969942258794049
0.7273179893428861 0.6145281425510660 0.9947755623840635

0.5607308506411783 0.4491687301632439 0.9986432997540220
0.2256946940718692 0.2801999470187440 0.9966060202678548
0.8928006275625648 0.1139576791639172 0.9947806058132898
0.7266586105352530 0.4479256084898324 0.9976995263355235
0.3920759337692274 0.2806834023918654 0.0066901524019940
0.0598937542277298 0.1142670756484705 0.9942767192040733
0.8935780107383025 0.9477195214650322 0.9945195096390762
0.5608516656567275 0.2806090071249727 0.0022780057540785
0.2266482211369976 0.1137952589961557 0.9958954633155201
0.0597449991053978 0.9472798937913538 0.9933072119698068
0.7265432020790215 0.2806946326785891 0.9977883180178964
0.3928359703247466 0.1137738708051828 0.9987543970750608
0.2265057900686855 0.9476380757211516 0.9944585234546608
0.8933952241363698 0.7811496672024703 0.9938055996976090
0.5599413631881589 0.1145031977044582 0.9977962696344704
0.3936103109994633 0.9477773459347745 0.9962022627695504
0.0600980438940866 0.7808917341414059 0.9939103824308901
0.7267546663524911 0.1143250064942052 0.9966171796575409
0.5598717645451003 0.9475288588707897 0.9983012891419634
0.2264291439822941 0.7808976970991424 0.9960283095012628
0.7269485334598258 0.9482864010437524 0.9971424564133571
0.3930629270574357 0.7814196446067285 0.0000893873157040
0.5616118718260594 0.7827027557719717 0.0047222117337284
0.7269500971755321 0.7811274938422486 0.9974437670915748
0.0042031421935533 0.6696355165554095 0.9952453452387928
0.1705186705620036 0.6700974682277803 0.9979877738140601
0.3365343619195897 0.6706616540073966 0.0037931917104359
0.0032423422993233 0.5028394468282471 0.9990454574834118
0.1694999362666607 0.5032889778802456 0.0056173783055939
0.6712833301232024 0.6699809348819059 0.9971436652341339
0.3364864754092238 0.5011933737281993 0.0079150757700077
0.0033601523128084 0.3361095484113079 0.9980873307876692
0.1687050119590836 0.3342089397891144 0.9992271160673525
0.5045644612324622 0.5035122047541805 0.9961251330640977
0.8375444314533240 0.6697118220569820 0.9941108828695420
0.6714111084815215 0.5037029742851260 0.9965379952434290
0.0038313553911848 0.1693254025049313 0.9945119929923081
0.3367534399403169 0.3368702430991490 0.9977597143527674
0.1703947843017559 0.1694573602009370 0.9954234333617649
0.5050039061562899 0.3374741540678770 0.0078471414261969
0.8370045336108660 0.5030982088735030 0.9966657042141236
0.3361697132933443 0.1681997878253227 0.0002422690942296
0.6705640244770663 0.3361109812114265 0.9992990477396972
0.0037799621108334 0.0026925718415072 0.9937585561915654
0.5043292908230654 0.1696993023707307 0.9998343701676902
0.1706716549152105 0.0025728693337115 0.9939402549242686
0.8374429456468633 0.3363452984378483 0.9974894302367119
0.0039897640368451 0.8363623733821370 0.9932991352932632
0.6707104523720417 0.1698091892178866 0.9974969782382459
0.3372936940330575 0.0031559463677553 0.9959721582454364
0.1704404162655351 0.8361544536620542 0.9943619331491220
0.8370449990399393 0.1693967166986354 0.9957782679308949
0.5037384118180768 0.0029394949035932 0.9970571359828284
0.3375675939712863 0.8365241157144609 0.9967149847698025
0.6711242364422247 0.0032105686493864 0.9974285318156575
0.5045750588188085 0.8371714239919754 0.0013591536185302
0.8375160577675445 0.0032358796409397 0.9952017851778905
0.6711966128066891 0.8368376986783446 0.9997084111441126
0.8375768323267465 0.8363561471103367 0.9949361157185166
0.5063327979139274 0.6706130799526093 0.0108624650785103
0.4500992607518128 0.3375861771523319 0.2586959812752241
0.4094570543020879 0.4527720860459894 0.1857117708449181
0.3441878691327034 0.5626554050584858 0.2480367337421470
0.2604060359045945 0.4687742221674682 0.1238183031407729
0.4494559570972214 0.3198732269458020 0.1224210851198251
0.4462435528322715 0.6376455613084602 0.1264720115365847

PT6(no-D)-XIX – ΔE = 0.75 eV
1.00000000000000
14.7772044699999991 0.0000000000000000 0.0000000000000000
-7.3886022400000000 12.7974344700000007 0.0000000000000000
0.0000000000000000 0.0000000000000000 18.0009597799999987
C Pt
72 6
Direct
0.5591658891449158 0.9447619598824705 0.9940488005077802
0.5582755462756110 0.7779317557123484 0.0035202519008862
0.5568839097664128 0.6117802928581936 0.0201646799226809
0.7256859226561869 0.9446167412796548 0.0019481980818950
0.7266898014830916 0.7800751246565913 0.0138776989337501
0.5596654361056949 0.4435793932488822 0.0014683335774085
0.5591334688362626 0.2778852049546444 0.9909556190771016
0.7265745350407593 0.6105028743722568 0.0168696426979602
0.8922836759173138 0.9450289296937709 0.0057396847461596
0.8928022013481421 0.7783787296290977 0.0088995043332328
0.7254381862647408 0.4442821354486874 0.9967365979241504
0.5589712605963620 0.1112132084138848 0.9897721655721690
0.7257343606896650 0.2779621260997521 0.9909090157625092
0.8928795600225349 0.6114226264833923 0.0018017139377297
0.0590125837946809 0.9450015093501558 0.0032698093792886
0.7257849059410438 0.1113207489962074 0.9941271804455849
0.8920436214135700 0.4449013510979906 0.9939487032046365
0.0589444547948409 0.7782440695363604 0.9989681996361953
0.2254506291881936 0.9447136842161091 0.9952700328748244
0.8922272599117401 0.2780375844332395 0.9948484005075678
0.0588062867445786 0.6112696884991652 0.9937586861781506
0.2256929107942017 0.7782834940973155 0.9920850081545192
0.8927733744414569 0.1118155901385689 0.0014506926782165
0.0587028649352601 0.4448521236129230 0.9941004677787952
0.2252815414338080 0.6115604781182941 0.9918465140776788
0.3922819695023779 0.9449143708509719 0.9908564308695731
0.0590458509835799 0.2786719429985733 0.0022939069856834
0.2248369390833272 0.4448979700103647 0.9991247593949331
0.3921726885540713 0.7780585608791153 0.9928818949980217
0.0587304228114760 0.1111388943346933 0.0070504380365965
0.2246625908287798 0.2779351773125774 0.0155756769208111

0.3915600810952762 0.6115753374032724 0.9987978090702070
0.2250116251580717 0.1113075785648528 0.0020327252550993
0.3903712219873296 0.4444563423870122 0.0109567955889610
0.3927424944739149 0.2768395694146005 0.9991233158406476
0.3923325083468521 0.1114728333638036 0.9921438028837385
0.5032777668040365 0.8336053697467090 0.9961788168338305
0.5022442529122959 0.6679000474436684 0.0066492217713511
0.5032070908461498 0.4991709358554077 0.0131164354808109
0.6704395126387954 0.8341459841848241 0.0070433837210017
0.6714989133410781 0.6684197020403357 0.0248402142451512
0.5031703443974237 0.1668002533314494 0.9898877683628821
0.6695489080152556 0.4987617395509238 0.0036247786938157
0.8369964109821203 0.8344067606331983 0.0094563289856672
0.8378340384511503 0.6672890554348282 0.0102654212228141
0.6701139522847387 0.3336309349662465 0.9916401049756093
0.5032872479349737 0.0002999212177065 0.9908092121794947
0.6700610427427800 0.1668965157346941 0.9907855340834111
0.0033836881580146 0.8336341999404251 0.0035991869681032
0.8369832254798860 0.5004518105915337 0.9974403438125737
0.0032691259190714 0.6671378528858146 0.9978666676513074
0.8365400388088229 0.3337616212145771 0.9925720459973724
0.6699132489813062 0.0002807104613356 0.9964252499910635
0.0033521487562668 0.5007761920762603 0.9935157167069014
0.8367450917973177 0.1672013297440272 0.9966888840284669
0.1698847819294483 0.8340251207085517 0.9949834633390829
0.0032997061206605 0.3339847433064023 0.9966877864069090
0.1699974777936425 0.6671923248981747 0.9919049565986312
0.8368635715079413 0.0004193364156849 0.0033929530239497
0.3363607947138016 0.8336291433127611 0.9912528692057824
0.0030545238985991 0.1670603050015677 0.0046207405733022
0.1694459320778520 0.5005119907734409 0.9939560778253043
0.3364473690789010 0.6672854315591074 0.9933071030307448
0.0035225233817129 0.0004174005590602 0.0052784243140724
0.1690533921157405 0.3342360243330560 0.0057613883300875
0.3353180822952425 0.5010072444696229 0.0017761785115228
0.1694339107488325 0.1658160989393047 0.0091997622756544
0.3368159113568138 0.3324145268143102 0.0102374080708986
0.1698078561110243 0.0005667764004289 0.9997840577321560
0.3369613848773372 0.1671585558185592 0.9970396752983156
0.3365454745624340 0.0002660829306512 0.9921769462274490
0.5038072021794796 0.3333453103633009 0.9949703376993441
0.4407216962374620 0.4137992311391419 0.2451917928342934
0.5035522396552262 0.5282379899440052 0.1276985705165230
0.3206495247995095 0.3445680893820240 0.1273525771997812
0.6992090941577020 0.6298809437821867 0.1357112067916810
0.5980392537907209 0.5938381125760307 0.2581023438274275
0.2653320669350690 0.2590701271462984 0.2525859961040538

PT6(no-D)-XX – ΔE = 0.75 eV

1.00000000000000

14.7772044699999991 0.0000000000000000 0.0000000000000000
-7.3886022400000000 12.7974344700000007 0.0000000000000000
0.0000000000000000 0.0000000000000000 18.0009597799999987

C Pt
72 6
Direct

0.8848665009660266 0.6062748740942396 0.9950810273028452
0.0515712062214178 0.6063107932150800 0.9902998040669644
0.2180715385919996 0.6062411379720700 0.9893632014168148
0.8849042158465537 0.4393727876014424 0.9902879414668035
0.0515254569755470 0.4395604655327361 0.9914194792396458
0.3846265507678410 0.6068769469377244 0.9939805401268416
0.5504681986460795 0.6062102479899067 0.0114975697776387
0.2184274213264885 0.4400082883744219 0.9959811454395364
0.8852676594725466 0.2728811298142517 0.9943575459402823
0.0518393673546171 0.2728425030161361 0.9990593094547933
0.3837557484460206 0.4398855545227107 0.0084546628253861
0.7191537379619106 0.6052510138659883 0.0048102529661449
0.5522789482403212 0.4383603826799174 0.9994043794005094
0.2171110433040226 0.2724859510346889 0.0080937258448870
0.8849045615522755 0.1064120045784307 0.0033036764759942
0.7185404872784815 0.4394469804045755 0.9915037390971193
0.3855367652997685 0.2720732995988939 0.0040896964348818
0.0516833631786184 0.1065429654711281 0.0048400754000752
0.8856815686167465 0.9400249597775172 0.0090801773725531
0.5517588132100073 0.2724182333967562 0.9923610653092467
0.2183489767512512 0.1060839255601067 0.0040232973643839
0.0516937524599115 0.9398714302581297 0.0031956209685688
0.7184485700658172 0.2729792708446297 0.9909406764729525
0.3852482878397083 0.1062631638016072 0.9960358906708606
0.2184612099552510 0.9397381296636240 0.9966559399963089
0.8853925466282604 0.7734313687024255 0.0062783226670931
0.5520199459334520 0.1063240965326813 0.9930727431719788
0.3850161677245652 0.9395297319641429 0.9926308396744901
0.0515490547180022 0.7729074250625914 0.9961468695410289
0.7183984693362291 0.1062918531653878 0.9976140196044412
0.5517104181690229 0.9400104598650998 0.9974305964590826
0.2181643399715796 0.7730868041065975 0.9907815605751793
0.7185595715178863 0.9408665359726385 0.0100507766769979
0.3848807947630064 0.7731536597453612 0.9914802127485274
0.5500515973702491 0.7726077348444349 0.0030769888215048
0.7196466231935048 0.7744022352779609 0.0238434727158889
0.9960260289464529 0.6621599985228457 0.9934635984770068
0.1624878570455266 0.6620203071690440 0.9894873140198541
0.3291467410637523 0.6621938452291403 0.9904138588052049
0.9961861962680487 0.4953040861598339 0.9901225088584482
0.1627366570684003 0.4953693317771695 0.9913467864821541
0.6630353775638156 0.6598437910095285 0.0185319238967523
0.3281272025645237 0.4957907253295559 0.9978346003877050
0.9959653293738739 0.3284563036270782 0.9945443122701292
0.1625947035567776 0.3280546407449592 0.0010453455545374
0.4957626783188189 0.4934062409358972 0.0088557990771037
0.8291881465961524 0.6612820156940629 0.0022462959314424
0.6628369736544002 0.4945974265249973 0.9965992594162785
0.9962771956903822 0.1620405251059367 0.0027872557349014
0.3293107510991433 0.3277467909203011 0.0140440811821918

0.1624286551317340 0.1617730702299411 0.0063247704927463
0.4961582263938737 0.3278492808402120 0.9970375888502190
0.8293216154755427 0.4951778173434747 0.9915591690021301
0.3299526240287278 0.1620469705916534 0.0012971922452252
0.6628198366632176 0.3282632993849575 0.9903125671635351
0.9962983723250858 0.9952981756682391 0.0054517117975195
0.4961025795272178 0.1616646996885791 0.9932024580958156
0.1630925797330463 0.9957906969417820 0.0012522373591963
0.8295439946832488 0.3286906623725017 0.9912238339477071
0.9959014512837143 0.8286907592556432 0.0016066393990997
0.6625246884577436 0.1617911236866902 0.9932234341716679
0.3292932601704379 0.9951936648351563 0.9947256686581767
0.1625821821317643 0.8287857569204249 0.9941149054191811
0.8295054767463768 0.1619634397805356 0.9985877409600050
0.4961449372054147 0.9950307669968268 0.9938937727096828
0.3292105871131881 0.8287413207602796 0.9909841847285534
0.6626848421070122 0.9959073674886412 0.0017234593615285
0.4955368720355366 0.8284759416102432 0.9965753985966899
0.8296522587033976 0.9958295057421296 0.0086372206992706
0.6617729393223755 0.8295641749475493 0.0163186533995798
0.8313431446365698 0.8296796421385295 0.0131628596776991
0.4948629683594561 0.6627283666403656 0.0004398129074232
0.3967743710858471 0.4219102085770601 0.1254808305693089
0.5799901911850966 0.6054518784461038 0.1260264201141794
0.4680068192232198 0.5397313661579730 0.2444472441260572
0.6469199928738334 0.6988061131133065 0.2567731152570261
0.3155207631293422 0.3628752028509581 0.2516194603560820
0.6814041080489162 0.8011409015325128 0.1349695829678623

PT6(no-D)-XXI – ΔE = 0.76 eV

1.00000000000000

14.7772044699999991 0.0000000000000000 0.0000000000000000

-7.3886022400000000 12.7974344700000007 0.0000000000000000

0.0000000000000000 0.0000000000000000 18.0009597799999987

C Pt

72 6

Direct

0.8901732149645625 0.6121190494292819 0.0008971232835009
0.0567491652246090 0.6121409117514887 0.9964513016383378
0.2231348891687759 0.6119627601906856 0.9962171218585638
0.8902602809698053 0.4451242286512951 0.9981926704837107
0.0563985985591984 0.4453338539724427 0.9972230919085874
0.3892839308081477 0.6119436929747408 0.0009360839177432
0.5572196321910994 0.6135734372709578 0.0072336339797658
0.2229344328783114 0.4456640601619455 0.0021915605964651
0.8903066070316115 0.2788980409752142 0.9948195192341629
0.0570549253525812 0.2787979229350910 0.9977535484500279
0.3876733595328687 0.4446111696485886 0.0189697488960903
0.7244639827547132 0.6135126699006221 0.0072059327794705
0.5566702124150922 0.4439400479326636 0.0182826743019078
0.2231018153286115 0.2783002336195253 0.0020456834965046
0.8900739119302870 0.1122079247753973 0.9927864824133223
0.7246610929341912 0.4449362689538958 0.0060641001567490
0.3894073140367382 0.2781213257337711 0.0023520974596707
0.0567208632322647 0.1121300006197217 0.9945270042090826
0.8899269286359370 0.9453485307237770 0.9928014151197004
0.5562621959900085 0.2780631674150555 0.9981660944890365
0.2230683449834245 0.1118094932055342 0.9943277200846197
0.0563984142455709 0.9453881796489867 0.9926018915124004
0.7232430416234621 0.2787172705553118 0.9949792683270360
0.3897399117119349 0.1120403576915052 0.9916364367284487
0.2233268748524324 0.9455605123055406 0.9908032249899392
0.8901556581604737 0.7790920647033701 0.9965939545960296
0.5569178825298664 0.1121885593771523 0.9899843947914349
0.3900657518979571 0.9453687962961297 0.9891144559607170
0.0565902468748405 0.7787252758284495 0.9943804537949603
0.7234162696619251 0.1119853585119728 0.9907346463987352
0.5566180245528471 0.9452499767832307 0.9894020232344118
0.2231451094746362 0.7787353455649040 0.9921588390547029
0.7234488746642569 0.9453629392400629 0.9912739858565018
0.3901004761300655 0.7787883089833656 0.9920429859349795
0.5565804262956675 0.7784155441008380 0.9944496022540008
0.7232464285935478 0.7788147782104176 0.9966028412125212
0.0003630967033885 0.6672654914541241 0.9970962265186003
0.1670249315115910 0.6673225226796689 0.9946677607416561
0.3337912410760993 0.6670180841881290 0.9957905890955772
0.0007480982976915 0.5005949655671031 0.9972011078036758
0.1671743024438896 0.5009457178047327 0.9982037961268588
0.6674119684552977 0.6676859429701949 0.0031810496361615
0.3324548889520287 0.5012053469664366 0.0069807333604217
0.0005674932551045 0.3340987299132365 0.9967674712494201
0.1668610759111928 0.3336739647411520 0.0015878423146987
0.5002130536199658 0.5021404836350030 0.0206994500409294
0.8338487702573545 0.6673957816980405 0.0015868751169990
0.6690291523993679 0.5013300799553235 0.0155339651991682
0.0007139358914401 0.1673196009951354 0.9951004691005423
0.3326262880720918 0.3323008857776273 0.0080961611811432
0.1669832377326230 0.1669270574707298 0.9966549019318549
0.4998297914561789 0.3323058670710495 0.0054625927943732
0.8344106871686989 0.5005856380836704 0.0019104652830819
0.3337001740088184 0.1670411816148629 0.9951779917400785
0.6677145047021469 0.3334974741987580 0.9997158444118455
0.0005519691673399 0.0004133011915854 0.9928782907313689
0.5006624790490264 0.1674182147053358 0.9921800003727128
0.1672706762448755 0.0006549157746107 0.9920381595922478
0.8343061252258011 0.3341585920397918 0.9959836517937006
0.0004200887089851 0.8339278484904042 0.9941360554258694
0.6670841739282878 0.1670958497336059 0.9913270368205360
0.3337529066862359 0.0007266464526268 0.9898050673793222
0.1669914946998148 0.8337299697060985 0.9919557503318046
0.8339542513755944 0.1671534325461224 0.9926266273790176
0.5004709364821167 0.0004119690353050 0.9888098839575434
0.3338987236691011 0.8339673208786920 0.9905287348602769
0.6672541047883058 0.0005284335785731 0.9899760392807693
0.5003303120725420 0.8337315564020074 0.9913348431924831
0.8338299433593548 0.0005132154695247 0.9918965603134211

0.6672687112425351 0.8337434673889561 0.9936582538399179
0.8340837556858816 0.8341011106477367 0.9950248309564245
0.5008764741108322 0.6676409658381246 0.0004312177632997
0.5984185036441261 0.4075740482906696 0.2695598363855325
0.4440862999834891 0.4700289368041126 0.1347353094670609
0.6220823347601865 0.5844345242215709 0.2289138455218165
0.6106822908656184 0.4479204378000006 0.1305598536421329
0.4418665786778178 0.4401199969884573 0.2728579536579545
0.2913151477942648 0.4427145112021122 0.2161142770195781

PT6(no-D)-XXII – ΔE = 0.77 eV
1.00000000000000
14.7772044699999991 0.0000000000000000 0.0000000000000000
-7.3886022400000000 12.7974344700000007 0.0000000000000000
0.0000000000000000 0.0000000000000000 18.0009597799999987
C Pt
72 6
Direct
0.8873155165706237 0.6065785626916451 0.9923683533655279
0.0532914445733610 0.6065820607763399 0.9914671589111350
0.2200041868733607 0.6072971345989302 0.9972746939988539
0.8872715938181415 0.4404774102582607 0.9911612467670849
0.0543429316978958 0.4402888837970309 0.9920176632102553
0.3849833532963842 0.6060202066966980 0.0154965855935814
0.5553759702115997 0.6068937991328198 0.0194333601411216
0.2199325921676731 0.4395254652590808 0.9969547066502429
0.8869023296046095 0.2733357739083786 0.9905245861149936
0.0536209129773084 0.2736645385381138 0.9909028958580279
0.3852697860188314 0.4391547105991265 0.0042623297673998
0.7214527231819261 0.6073919130080938 0.0004656418848512
0.5543483914176051 0.4391154901913623 0.0052795895502271
0.2203013066078583 0.2732928123904443 0.9934579796126926
0.8871182189590314 0.1067453293139948 0.9915027457821139
0.7202091684997001 0.4398810262345592 0.9952598243088602
0.3867151715624857 0.2730368830156777 0.9966445623409470
0.0533356591952625 0.1066157715546652 0.9906462043956168
0.8870273504782915 0.9405406342444351 0.9938583554392153
0.5539012541960204 0.2736373401460384 0.9950809642812430
0.2202913536596967 0.1071585139183284 0.9913552409319308
0.0537455607554591 0.9401411335388801 0.9910374998472093
0.7206501525467246 0.2733829904847482 0.9919398941828703
0.3874008528613899 0.1068648304443585 0.9924698927486588
0.2200167353936351 0.9400055517657577 0.9910834162448410
0.8868104445696332 0.7734144369289027 0.9942586937339826
0.5534788704297640 0.1065012239478591 0.9924282200994270
0.3871457737569699 0.9406447951053138 0.9930958749119654
0.0535894949042230 0.7737447086719627 0.9910347421798633
0.7203613996687892 0.1069829195264660 0.9923506818582197
0.5538780315279936 0.9400326601866951 0.9960896284166054
0.2203549409778276 0.7734423613092360 0.9931395841087678
0.7200997004734475 0.9400709583937896 0.9971572769142014
0.3863296709213770 0.7736493050127748 0.0006276156564269
0.5544225695972358 0.7755183079869923 0.0083954442989977
0.7210922798709731 0.7737974014037547 0.0031298494221232
0.9979459470001899 0.6621008715932817 0.9911617277866682
0.1650777243677908 0.6625904143193324 0.9934864661780054
0.3303433905567488 0.6629727267987278 0.0043621350573417
0.9983565996478561 0.4960498932060204 0.9912695050505036
0.1645190665936056 0.4953810933015106 0.9954394395366677
0.6669996008817947 0.6630881739013574 0.0076058516111388
0.3305095857346032 0.4939357916274076 0.0043998677905535
0.9986767278216817 0.3289791491371687 0.9907999627171691
0.1647384512796108 0.3288845931925195 0.9931981438421218
0.4974340728384234 0.4940790850848700 0.0144159865669593
0.8315091867485123 0.6624400547816265 0.9949265654370834
0.6653165247345498 0.4951175012778108 0.0007506908083030
0.9980195842554025 0.1619710637930964 0.9905831355564914
0.3309621349895764 0.3286243016985537 0.9974726041318362
0.1651076946087144 0.1622070871864736 0.9912498190553478
0.4986323959966725 0.3285470970367257 0.9989495665862762
0.8321966477835474 0.4958303082445212 0.9925616013466652
0.3316802553441534 0.1628676016082480 0.9930957044929869
0.6649503466948801 0.3290432069386426 0.9940864937222287
0.9980994729692441 0.9957558579059267 0.9914260935636250
0.4979529253518535 0.1618788327424667 0.9929438938170279
0.1646564438406344 0.9954461089487054 0.9906678345600071
0.8315149056603772 0.3287396560907609 0.9910478792400639
0.9982847337215759 0.8289813984140650 0.9916237336641700
0.6651902311105218 0.1622591691332431 0.9920455331162756
0.3318525684168776 0.9957624236958580 0.9917860612157838
0.1647011187951009 0.8292136249725530 0.9912646617738758
0.8315659536652191 0.1624548252411913 0.9912661193630825
0.4980322696309472 0.9958584191868809 0.9934180356144040
0.3312525583559705 0.8286480715025419 0.9950158020846587
0.6647957044891015 0.9954636547880469 0.9951325202799595
0.4988490270652264 0.8298505834187040 0.0015413407060905
0.8318945026683551 0.9957190980589203 0.9939660925181713
0.6650718834686842 0.8296967036829201 0.0033623526672102
0.8313969214928747 0.8287665960870214 0.9968002209764251
0.4982867671848581 0.6642218792495100 0.0210221842934786
0.4993434407752275 0.6706365926684086 0.2678451464867493
0.3011959955482979 0.5777707667943304 0.2423055587134257
0.4286456559376006 0.6338551217768753 0.1315564330048176
0.4003391570058596 0.4745920724984245 0.2421757012336414
0.5457777792877465 0.5459936254580313 0.1294323950866385
0.6006837163518526 0.5716571114921933 0.2650671423378412

PT6(no-D)-XXIII – ΔE = 0.78 eV
1.00000000000000
14.7772044699999991 0.0000000000000000 0.0000000000000000
-7.3886022400000000 12.7974344700000007 0.0000000000000000
0.0000000000000000 0.0000000000000000 18.0009597799999987
C Pt
72 6
Direct
0.2249110946596198 0.2736744933610552 0.9992716048801700

0.0583902636304489 0.1077796488085259 0.9895116651261842
0.8922596523504572 0.9416021398691589 0.9897560276793745
0.0588761986180590 0.2747769833560120 0.9922153645015586
0.8921111848958574 0.1078680366049081 0.9885032663172418
0.7263504462794989 0.7750491787545668 0.9998964651957205
0.5612527948457567 0.6100835791487634 0.0236105709309720
0.7252779328857907 0.9411722300428916 0.9928016908314996
0.8917325074129362 0.2745265136832058 0.9891001845040952
0.7255663862726338 0.1082778121484362 0.9894831144145876
0.5598701282323024 0.7768215556890807 0.0050694217079930
0.3898761536307971 0.4387791288962778 0.0229738116633212
0.3908879262701632 0.6091103727876832 0.0095878926189823
0.5591161808830730 0.9417003443648714 0.9930079512912032
0.7252258701287913 0.2747578912006812 0.9907204940148254
0.2231520116103008 0.4402061964788708 0.0045668096471663
0.3923847557196662 0.7750509337910682 0.9959303960966422
0.5586842233500580 0.1079643995212850 0.9911880540400723
0.5588511096023233 0.2743142010097545 0.9979764863423042
0.2250546036299996 0.6077107420597341 0.9956702575129431
0.3925166759859643 0.9415384404525386 0.9909136691460143
0.3921797486265769 0.1076369150074884 0.9933125736674517
0.0582917687014645 0.4409024124770469 0.9924133106803723
0.2256810983894555 0.7744030435640001 0.9903843669809962
0.2254322388224203 0.9411331619219744 0.9893402061511078
0.3909069578562830 0.2732873404961938 0.0034442282263445
0.0585600320885457 0.6076429565880228 0.9903525375420048
0.0590317415016347 0.7746893858923746 0.9890530312482736
0.2254387534288241 0.1079001076261552 0.9922943046590998
0.8921489532163384 0.4414281730078878 0.9903739517839757
0.8924000535620177 0.6079046150930951 0.9922976130372773
0.0588728851219955 0.9411807939664615 0.9884960592822242
0.7257366924075687 0.4414339054056660 0.9966150430727352
0.8922239058585285 0.7746522557566904 0.9922239351488381
0.7267383378277333 0.6089839450492641 0.0027571778501393
0.5599951705925290 0.4404736598638516 0.0117128596714188
0.1700178768869248 0.1634995362636464 0.9928186645597700
0.0031743942014774 0.9968182691848360 0.9884865064132080
0.8365468359168855 0.8299759450893280 0.9931330558878932
0.0030603773832622 0.1630881928644088 0.9894432058634841
0.8369524515256097 0.9969997543136044 0.9897864483183773
0.5042773986501338 0.4955664104920516 0.0240109740723655
0.6704920610667456 0.8312289897682468 0.9992504029742134
0.8363888739389953 0.1636257561312483 0.9886342750665332
0.6698342328539307 0.9968780616572304 0.9916253917858378
0.5031956048812848 0.6656937084867709 0.0173656466366583
0.3348740317515616 0.3279874885399039 0.0081060275409184
0.3342479738557794 0.4967912007689557 0.0172809145650801
0.6699033148211435 0.1636742029671022 0.9902588334296231
0.5035857180565568 0.8310325145583780 0.9979251722544039
0.5030850872275484 0.9969822182341963 0.9911160977709372
0.3365181471085104 0.6635023498208080 0.9991178318750320
0.1687586678847708 0.3295815956291532 0.9985725253898974
0.3368376828074333 0.8299512274609824 0.9917944496247214
0.1690538776218838 0.4964852282262460 0.9974473005074753
0.5029952607488524 0.1630370997632044 0.9939632238120067
0.1701892420439960 0.6633415301986416 0.9915181941982470
0.3367832267202622 0.9969606259110552 0.9906339426974065
0.0031627557836739 0.3302938300367026 0.9910484918245381
0.3361971748956126 0.1633081315951772 0.9957109318500845
0.0030378068715989 0.4969927019223817 0.9904638597040979
0.1701092479787605 0.8300753679658013 0.9888874132789113
0.1698942545954580 0.9965877902146767 0.9893348467116020
0.8364222194194973 0.3301902211029600 0.9897535939958786
0.0032930521881195 0.6633944173571389 0.9899855722657094
0.0035336616833770 0.8302352486754943 0.9892108359711206
0.8370434216894020 0.4972013107184752 0.9927226432028822
0.8368576379330218 0.6638919914915677 0.9951170838690653
0.6697428118413999 0.3301934825378842 0.9945055480328855
0.6718325988786220 0.4973752643363706 0.0043828645073258
0.5029198488545786 0.3285463157671680 0.0063831135591859
0.6719566706408675 0.6650361450448957 0.0086245546843235
0.5775221185028044 0.4115859071618611 0.2586558427766175
0.5254606191615842 0.6469500088548230 0.1347071410269008
0.5411255839322632 0.4459691785638453 0.1291418365840632
0.3541672145213262 0.4749515544362501 0.1345867068145897
0.3914862492318938 0.6088553045612282 0.2419377172537245
0.4907865258676419 0.5145265945662291 0.2510380939862309

PT6(no-D)-XXIV – ΔE = 0.79 eV
1.00000000000000
14.7772044699999991 0.0000000000000000 0.0000000000000000
-7.3886022400000000 12.7974344700000007 0.0000000000000000
0.0000000000000000 0.0000000000000000 18.0009597799999987
C Pt
72 6
Direct
0.2162470444831272 0.2758443948633044 0.0176897606827656
0.0510100508441708 0.1098249849056978 0.0055990775115357
0.8851266968283814 0.9442087777041911 0.0026204872028279
0.0519205412166667 0.2776712772914151 0.0025267816856202
0.8854400383749716 0.1107621259213851 -0.0005613528398907
0.7195596167009423 0.7790895179091647 0.0087526982011798
0.5524525530130721 0.6129169166937425 0.0188281035719978
0.7183943565033079 0.9436910890716725 0.9976903142398649
0.8852156499432995 0.2771076315834405 0.9938156700512791
0.7186184727711370 0.1105322977618900 0.9919734737231840
0.5512637276232821 0.7773373920006207 0.9985694684406620
0.3842937288013499 0.4445445545288043 0.0174936654106474
0.3849530310748739 0.6109052437728324 0.9990628162419197
0.5519834199528904 0.9440471630162977 0.9902754627592354
0.7185285828822102 0.2773064480943503 0.9902615895304938
0.2183238063785081 0.4441456797397858 0.0017228420133800
0.3850174234999443 0.7771101525334596 0.9900034672986419
0.5518943910375590 0.1106305328738073 0.9888051044414536
0.5517561668512800 0.2772473872209942 0.9929394924809312
0.2182904339814038 0.6104940687228528 0.9914611019311199

0.3851156620647129 0.9439827972948270 0.9885631768228132
0.3851133751745079 0.1105810873095879 0.9925588776498535
0.0517583264346932 0.4439143050985468 0.9938967539388164
0.2184454577873605 0.7771782572286644 0.9893544881756822
0.2182787202680119 0.9436431723300425 0.9932588165589119
0.3852572757016974 0.2758359947355118 0.0044967690621807
0.0516306677497876 0.6102838037715985 0.9908843612115857
0.0515728085862353 0.7771046250378462 0.9943082644754491
0.2178418882895806 0.1098085031557759 0.0027964612594445
0.8849930954094076 0.4437404161576797 0.9915733824243627
0.8852201380172771 0.6103645449360875 0.9954023045957130
0.0515794957144018 0.9437840439247163 0.0003304604523110
0.7185160039359781 0.4436586982749277 0.9948309189352509
0.8852089267082305 0.7773721104181682 0.0019123145234561
0.7197554949475516 0.6102759342642016 0.0065804606853879
0.5528836900691266 0.4431823557915441 0.0057072222914769
0.1615762230308140 0.1640288375945691 0.0094100190480142
0.9959987747993073 0.9991031997608513 0.0028359574847112
0.8300262795829478 0.8332772396034887 0.0046426836469640
0.9957487972692537 0.1657362589179267 0.0034246103486794
0.8296459456493415 0.9994380423587690 0.0003237555961389
0.4971102209144294 0.4988721812655569 0.0185297898404310
0.6632387734412605 0.8336062601252898 0.0017384079406283
0.8294864288415975 0.1661590369395439 0.9950101093453680
0.6626131016081652 0.9993985918448084 0.9928456582594075
0.4957313790408102 0.6673554428265883 0.0045044952200735
0.3287091072436528 0.3304046677579309 0.0184006313545341
0.3283795465915580 0.5000435392630976 0.0048897262588112
0.6626846288827376 0.1660206903580833 0.9896847031108092
0.4959600148713759 0.8326026727773095 0.9918719813504194
0.4959987968567662 0.9994160200351301 0.9884641033849598
0.3294159780446637 0.6661709044017298 0.9923515724396512
0.1614719717158580 0.3329279451960242 0.0076625956799874
0.3291327767561378 0.8325396513086385 0.9885585046834231
0.1625495396085264 0.4994817368104865 0.9948768586091687
0.4959176465021436 0.1660788129152016 0.9905718611616875
0.1626807112784530 0.6659846340385599 0.9898463105374322
0.3291683329946610 0.9992101263050441 0.9908258558917542
0.9959675374979511 0.3328382716028109 0.9964309087156749
0.3297733778040824 0.1660630631570313 0.9991902334661107
0.9961535054685232 0.4995534837835052 0.9915411802763969
0.1624434356522216 0.8327392698542150 0.9917627974229325
0.1625303869481822 0.9992007554970898 0.9983905165051876
0.8293671153167740 0.3327879693772918 0.9913240650506273
0.9957854260027958 0.6659190064042431 0.9930052645781871
0.9956441484034979 0.8323372693812104 -0.0014421505452073
0.8294604068445663 0.4992817757036409 0.9934524260421563
0.8292101728518135 0.6656237461774863 0.0009981974922950
0.6628441616442442 0.3327997425395673 0.9918525583661469
0.6628954662730577 0.4989592340524951 0.0016520226927501
0.4964055259391326 0.3325648799374578 0.9997466633498963
0.6649067180380525 0.6672684472499683 0.0166977059969155
0.3380574922083072 0.4032406645709270 0.2566541107339365
0.4408951636716130 0.4740444415596028 0.1297356100130528
0.6985561900233783 0.6982099020377744 0.2621382573406438
0.6188801201969149 0.6551652015685350 0.1331262543225439
0.5263798617787605 0.5418417012977849 0.2559377562746063
0.2736934657406750 0.2985439541048390 0.1350995551197949

PT6(no-D)-XXV – ΔE = 0.79 eV

1.00000000000000

14.7772044699999991 0.0000000000000000 0.0000000000000000
-7.3886022400000000 12.7974344700000007 0.0000000000000000
0.0000000000000000 0.0000000000000000 18.0009597799999987

C Pt
72 6

Direct

0.8937793148654833 0.6172808097447486 0.9933597832780308
0.0605404152206148 0.6172485300892419 0.9952301281661420
0.2268417492968098 0.6177824791292181 0.0020751970173478
0.8936867451339765 0.4504690300455394 0.9956311059762939
0.0600732908791954 0.4509340525899361 0.0022266628453309
0.3946119628457936 0.6192330493660521 0.0091222342933364
0.5618909864224122 0.6195090009320836 0.0013607181431183
0.2253474672724067 0.4507050237282755 0.0204228786594740
0.8943239871603197 0.2842774931083412 0.9960237630492728
0.0603579440019075 0.2837838894837574 0.0030228655674733
0.3964289184655598 0.4514574854304882 0.0231507550257248
0.7270627324742236 0.6169178267426858 0.9955820063906984
0.5617270967537493 0.4505001962950885 0.0110567054137789
0.2250401964509550 0.2815972428937528 0.0190750976694289
0.8942364918654633 0.1175818563953399 0.9939376820196131
0.7276523973031672 0.4505959563219122 0.9984516267079755
0.3942685537006412 0.2818763274669394 0.0090149542110396
0.0600509010661980 0.1164428426945037 0.9995693114150370
0.8934780655419345 0.9503348578205006 0.9911910005696853
0.5602435178084662 0.2833997602852989 0.9989992252021480
0.2265452935046568 0.1160036424881881 0.9999668211957911
0.0602661262748256 0.9504866921188224 0.9925012693371968
0.7272131255533196 0.2842540886833120 0.9948316022302848
0.3939550763378392 0.1171446403116505 0.9947343571522254
0.2274908963084883 0.9503822352571163 0.9913379841201575
0.8937645810361232 0.7837855575102850 0.9905505454342176
0.5607902090846935 0.1172421052750892 0.9920369977029253
0.3938437057210535 0.9503997653214640 0.9904779213399877
0.0602917909649818 0.7836744928573509 0.9909665810506496
0.7274583956855167 0.1175441016825474 0.9915529320725298
0.5607742882299505 0.9509337912669977 0.9904570175843617
0.2274272098192256 0.7842212343735184 0.9922273916646986
0.7274656263510124 0.9508138615666013 0.9902887978726014
0.3940984344399183 0.7838801418446906 0.9943407303494141
0.5604971390618587 0.7843480069482140 0.9941312387720416
0.7271837470224298 0.7842858899805094 0.9914303383652836
0.0049417627449628 0.6729845650594299 0.9925719241261035
0.1716398756320672 0.6726307365444057 0.9958163143634877
0.3388884063733855 0.6736580298916195 0.0014715707180670

0.0050118507309591 0.5064587561119893 0.9972663395034331
0.1707846189155404 0.5072703007784014 0.0085179128849333
0.6715809530590775 0.6730931848176667 0.9950165471997039
0.3393200552339835 0.5087891846363917 0.0250441524464051
0.0047480400800310 0.3397689443327394 0.0003843642126000
0.1693110765821970 0.3382687643704116 0.0130248147318071
0.5066358570530127 0.5077652149420970 0.0099532566413814
0.8380408328618785 0.6724976462294805 0.9922894736877623
0.6725995019187749 0.5066825149018257 0.0017283957856193
0.0048207055271519 0.1724990517576970 0.9985756472368550
0.3385380010893897 0.3372147960948197 0.0234354131933827
0.1703853813851097 0.1704829983190663 0.0064189506551102
0.5048285419803804 0.3377525769757384 0.0061468764930055
0.8383495519622102 0.5061831323154280 0.9953344989527153
0.3386151742397203 0.1720301627909109 0.0005178667288561
0.6721025023369549 0.3395504027696390 0.9976384256254676
0.0048799690443388 0.0056388739822353 0.9938187913499945
0.5049451653827504 0.1726771325280438 0.9949191986887698
0.1715805045059042 0.0061994269020644 0.9938318363069101
0.8385274042219919 0.3399972055146279 0.9954271527132832
0.0046842501289461 0.8390647599382675 0.9906200066548223
0.6717419236073141 0.1728415518713433 0.9926270174872300
0.3380888178596351 0.0056292173139454 0.9915075393350321
0.1717838769178570 0.8393776952665348 0.9908140096106877
0.8387360168057185 0.1730653940483009 0.9935712214462313
0.5051894084809589 0.0062094400702009 0.9905129637467738
0.3382064154522340 0.8395902080875501 0.9916687905000359
0.6718587952260293 0.0064788942167482 0.9904039588631832
0.5050645503612827 0.8396627391294373 0.9926474703356334
0.8383775870109673 0.0060031156220859 0.9913870567216918
0.6719099016446606 0.8397030050669372 0.9911928446527511
0.8382659641908816 0.8395586955859926 0.9903314053994308
0.5053165803651396 0.6740885370836907 0.0011424599989383
0.1892162540448865 0.3518613200169014 0.2491850363668959
0.5702181186323472 0.5096008357266584 0.2528856254646712
0.4932796973338114 0.4764320855635802 0.1254471671215356
0.3837005095845001 0.4498381470297446 0.2471514311571426
0.2694206752333699 0.3050163761939828 0.1360966607471141
0.2732696438953255 0.4782434758519898 0.1372431706228667

PT6(no-D)-XXVI – ΔE = 0.79 eV

1.00000000000000

14.7772044699999991 0.0000000000000000 0.0000000000000000

-7.3886022400000000 12.7974344700000007 0.0000000000000000

0.0000000000000000 0.0000000000000000 18.0009597799999987

C Pt

72 6

Direct

0.2282316746789366 0.2783504294361632 0.0061595277175391
0.0610275140926928 0.1128799151231649 0.9973093281952901
0.8946307221863036 0.9469266701556396 0.9943143605986740
0.0605321775053802 0.2795132347377097 0.0020464579479618
0.8948157596700196 0.1132987760283633 0.9949081727005697
0.7283467346606233 0.7804481691172250 0.9987866185601328
0.5637986903822974 0.6144244894377081 0.0117023074499294
0.7281538068512445 0.9469129947018129 0.9961346250124175
0.8950985061732576 0.2799358685092519 0.9926283893579458
0.7282193454502773 0.1135111362684796 0.9922365459452698
0.5623867389867064 0.7819367525187462 0.0075156745804354
0.3946181874523073 0.4441247123581433 0.0230552907391168
0.3939569761359724 0.6149620989401904 0.0249178036355019
0.5615097107975444 0.9464549555022727 0.9953357318423185
0.7284516155805992 0.2798375136841417 0.9905834226574433
0.2264090196466925 0.4465009510405172 0.0106420350965423
0.3942621764284135 0.7802297199677535 0.0015109247507926
0.5613606291823189 0.1130612531112354 0.9906202691938188
0.5615452305834541 0.2800788016375521 0.9933897857890297
0.2277889476967410 0.6142353990263056 0.0018949261172363
0.3949421123507366 0.9468466993434991 0.9917520010894122
0.3950375377571824 0.1129879549768716 0.9913424556986923
0.0615059232529523 0.4469561283906580 0.9959195926345399
0.2280706422704224 0.7800528182276205 0.9931233540720201
0.2279517068254892 0.9464633687940340 0.9905596283641048
0.3944655379678345 0.2788595535433767 0.0000627010251513
0.0612148690322348 0.6132480111385670 0.9918482399837433
0.0612808051036329 0.7800612524982209 0.9903660379826636
0.2281844442739966 0.1131791936143561 0.9947112735036375
0.8948741497358109 0.4467499636834298 0.9906997971729894
0.8947564526674945 0.6130692515680131 0.9910855366715782
0.0613970344197823 0.9465333174979624 0.9921289105590863
0.7282549463409959 0.4462169736347441 0.9933186728299255
0.8943882574059048 0.7797692694517862 0.9925429960722483
0.7282760675501146 0.6131288166398753 0.9973239680630036
0.5627206531562498 0.4455897378871132 0.0054575740983015
0.1728641162960454 0.1685815005444371 0.9988512762033682
0.0059352296932538 0.0024766808500831 0.9941390501610566
0.8387987369213942 0.8353951380390972 0.9946486325317948
0.0054941123144197 0.1683799190454006 0.9982996393115283
0.8394864763951166 0.0022541424309484 0.9946363886656370
0.5081012138451800 0.5020626923756144 0.0180859981281831
0.6730555684166479 0.8362963885426393 0.0008453150047872
0.8393765450541127 0.1689700905637302 0.9932290020540481
0.6726151963483596 0.0021615010189053 0.9945243863217945
0.5078618161915377 0.6708133919623533 0.0194581306849881
0.3388110180468745 0.3330368775383894 0.0087628144329962
0.3367302760653437 0.5004800450388884 0.0233270647306471
0.6728543582669947 0.1688494501453154 0.9907957025622829
0.5063064729309240 0.8364077505056509 0.0015743576387786
0.5057215551938061 0.0022638932033590 0.9920719840939639
0.3385539755231083 0.6701097346256908 0.0094100018378014
0.1713543304294359 0.3341467680145253 0.0112920649469177
0.3390440086953589 0.8352464860084967 0.9946065500811013
0.1711863681812996 0.5030467873666131 0.0022914399209845
0.5056360801532604 0.1683374232961008 0.9911038382286708
0.1726231836533198 0.6693074265306981 0.9948497721945136
0.3392925363334583 0.0020057924827397 0.9905250281642322

0.0060497771786931 0.3353229793352622 0.9961882002352955
0.3391710800802523 0.1686369121561836 0.9944598302287950
0.0059320064704803 0.5025595907986398 0.9921816970287071
0.1723629550696445 0.8355987752556706 0.9906787284823050
0.1723530874237828 0.0018981214377163 0.9918496158005129
0.8393668760844000 0.3354794284822215 0.9908308924115907
0.0056764877117309 0.6687883084080560 0.9904304308650312
0.0056761132559069 0.8355167341461565 0.9910009597311519
0.8396565392901907 0.5023084668226151 0.9910565944942040
0.8388811497764371 0.6687876731299310 0.9930104126463051
0.6728602651346141 0.3356652868200066 0.9921923512919887
0.6730544989348866 0.5018536492091277 0.9985831766918976
0.5065385096530705 0.3349064861343078 -0.0007057597414958
0.6727075215297834 0.6688592605527157 0.0014821844567285
0.4646028962143475 0.4808682465496996 0.1353010835764900
0.1662391988450451 0.3881373956959393 0.2543891439686373
0.4617719945233637 0.6507576920138907 0.1366624993214315
0.5475684405619764 0.6085760113270258 0.2476670599918460
0.2409940613304618 0.4299985263463779 0.1262194391147090
0.3530590963836300 0.5128085541619215 0.2472856585532725

PT6(no-D)-XXVII – ΔE = 0.81 eV
1.00000000000000
14.7772044699999991 0.0000000000000000 0.0000000000000000
-7.3886022400000000 12.7974344700000007 0.0000000000000000
0.0000000000000000 0.0000000000000000 18.0009597799999987
C Pt
72 6
Direct
0.8842721737430850 0.6026217352889844 0.9983307596972182
0.0507595867702761 0.6028287916436028 0.9979990721561975
0.2165344142053343 0.6022411870229263 0.0028397253969885
0.8844060748598892 0.4360913876368784 0.9946407752019155
0.0509715036382090 0.4362856429704962 0.9953091255904525
0.3829971950243092 0.6014462772721814 0.0065609241011302
0.5503174432010098 0.6015926783735068 0.0154777370771981
0.2176755821122427 0.4359454229845596 0.9962596460397961
0.8845793509851703 0.2698941210656400 0.9920024160257768
0.0510398006028581 0.2693329940002300 0.9926454100604971
0.3841221793347813 0.4351685705813395 0.9979925816561988
0.7187308007471884 0.6019511917205378 0.0048654431580033
0.5504731407440886 0.4358659393990933 0.9985886899970851
0.2173344790602982 0.2692201931432052 0.9923884550609472
0.8840802347651575 0.1027829275925640 0.9929704104432204
0.7177456908460300 0.4365169431011111 0.9956445910172391
0.3842885543047387 0.2694572132678417 0.9927316810281752
0.0509804188103189 0.1030322048676240 0.9933277685000235
0.8844951958723399 0.9363426543413311 0.9982053874854486
0.5511839214868957 0.2695940536563413 0.9920053005195300
0.2177076836931775 0.1026662890349499 0.9925718331015574
0.0507149725287590 0.9360317805394658 0.9964787655671969
0.7176094047115313 0.2697513424330182 0.9914658627002595
0.3842478086647247 0.1028899034759618 0.9915303487244813
0.2176942264110124 0.9363023670042949 0.9947313603679007
0.8844386862600118 0.7696886390190514 0.0018996433543208
0.5512261273630372 0.1034008845228769 0.9903894447050021
0.3847030459166589 0.9365333607680739 0.9931690019585417
0.0509249411373602 0.7691633836093743 0.9980700365568111
0.7179076216014337 0.1029980989194996 0.9908060965842651
0.5511021348958636 0.9367814904697198 0.9918188931889347
0.2172680314480075 0.7693822726930790 0.9986252147335009
0.7176889589047377 0.9366219666685751 0.9953246952165173
0.3845729163228100 0.7709995353297217 0.9999822878664730
0.5515123226330445 0.7712530612093431 0.0006687811396588
0.7181795994915063 0.7705630239427705 0.0078293885253231
0.9954972126959944 0.6584858969590996 0.9980006565930140
0.1615027832391220 0.6583704005931423 0.0002570040662064
0.3280540935490137 0.6588876872035527 0.0082919186263837
0.9951724307641641 0.4915820753508697 0.9961773131756217
0.1619724673537632 0.4920727532851004 0.9980019817275405
0.6633762295143910 0.6584552931707393 0.0140976627189460
0.3272584625165535 0.4896491529243221 0.9996550786739036
0.9952910283599863 0.3251436312018399 0.9930281925494668
0.1617670987598387 0.3245661035774532 0.9932456784699326
0.4944497652835551 0.4900346188066749 0.0035372557336899
0.8288943955749772 0.6580119951445411 0.0017949149441989
0.6625169656508092 0.4916925304545003 0.9995312029980283
0.9956234879407120 0.1584218372622672 0.9927626395150568
0.3286169872637288 0.3245863863427303 0.9935287919808147
0.1617905807499653 0.1582034864583335 0.9923401101803222
0.4950964746365258 0.3251772762907095 0.9937847338210233
0.8286605654591597 0.4918167875280517 0.9962034110002946
0.3283793906741224 0.1580420218261409 0.9918606346818137
0.6618494623948052 0.3249042745604100 0.9926791463723532
0.9950907716750379 0.9914030966646408 0.9958447172067011
0.4954035295223278 0.1586659882403225 0.9908684721192103
0.1620153261894330 0.9914393529772241 0.9943285598460960
0.8289079952778167 0.3252549837559542 0.9924680959876753
0.9948942095356372 0.8249399225335523 0.9987307508229435
0.6618565623328010 0.1586397554781271 0.9903836160989243
0.3285761948135075 0.9917913118500010 0.9928343347902739
0.1619051065788923 0.8246233624617574 0.9970309134795201
0.8286403966680709 0.1582751183975191 0.9915764129989100
0.4952245924220335 0.9919328617641554 0.9912368942612488
0.3291777910335796 0.8253425597018023 0.9969517278209281
0.6622860568590880 0.9921917720098179 0.9918190359929397
0.4957437122318069 0.8261966409438699 0.9958046285511273
0.8286220526223715 0.9919943429406786 0.9955374866039080
0.6616736018081824 0.8256248051008015 0.0006238600390702
0.8288261388182221 0.8254366048478161 0.0031485519220738
0.4948604187353780 0.6596366386257273 0.0112194654656150
0.4057758760295016 0.6442406918514649 0.1201242786038108
0.3216865689735044 0.6263765785383484 0.2443723463532734
0.5785780051881488 0.5124891291373928 0.2304068339899317
0.4796139994676665 0.6138201020960352 0.2439611759908544
0.6097639635815852 0.6500333109257426 0.1270381785138355

0.6765468085757576 0.7045370440780090 0.2617708159479903

PT6(no-D)-XXVIII – ΔE = 0.81 eV

1.00000000000000

14.7772044699999991 0.0000000000000000 0.0000000000000000

-7.3886022400000000 12.7974344700000007 0.0000000000000000

0.0000000000000000 0.0000000000000000 18.0009597799999987

C Pt

72 6

Direct

0.2189243343709766 0.2794549936156804 0.9977736765414316

0.0525522009763652 0.1125942843434387 0.9941942368337054

0.8858236592587195 0.9462302773620549 0.9948471506543480

0.0520243214628088 0.2789733498636467 0.0007866391385217

0.8860551689889434 0.1128374830053562 0.9948410339568149

0.7199264206529032 0.7800131649302594 0.0012174650408458

0.5529616324338448 0.6146531448570093 0.0163898022636669

0.7191568688836251 0.9455400825914941 0.9933189998954862

0.8859771139902224 0.2790182562617633 0.9975162048914623

0.7190292199739479 0.1125414553823748 0.9917006507261517

0.5523182166710345 0.7797377739644020 0.9951492361652251

0.3844550000705328 0.4439100304878532 0.0062443942675630

0.3865032511483363 0.6147713202887037 0.0007085658996677

0.5528857872506036 0.9463243247411277 0.9881394081433187

0.7196758853201326 0.2795721825040668 0.9955239548560101

0.2178204182666230 0.4453189944231750 0.0113089666093127

0.3858567346880782 0.7792849665350943 0.9892010708217244

0.5527915817299122 0.1124144406494496 0.9879455298073659

0.5521553945986726 0.2786089663522444 0.9948239919199986

0.2183905617433142 0.6127652750167701 0.9960252462607764

0.3856636886588621 0.9461291496143851 0.9854969514256060

0.3862295837924268 0.1131296375764293 0.9870762774199819

0.0525867750255005 0.4463596116062081 0.0007969385258743

0.2194124539319001 0.7797604362246620 0.9887929562454945

0.2192473360968490 0.9460080450221280 0.9871748287840897

0.3861365944403745 0.2788486302362591 0.9949955304769552

0.0522751321283849 0.6123506871339335 0.9957679118040870

0.0522900319055162 0.7795308828678069 0.9927830389852995

0.2189858161693672 0.1128687068588815 0.9895519491377409

0.8857302192589113 0.4458726916571251 0.9991739529442705

0.8864384961106812 0.6131288379824051 0.0008814574032648

0.0528674054751362 0.9465591297608442 0.9915496746740383

0.7201687890794805 0.4452558140179143 0.0059408291451825

0.8861886254569247 0.7796344685723030 0.9982950935778748

0.7204063030546681 0.6144352568140941 0.0097432553006522

0.5526928715102752 0.4432482093879443 0.0177987398757176

0.1635964125915329 0.1682018880492983 0.9932030739454552

0.9974108844480958 0.0019515623419263 0.9932342287735039

0.8305453851521453 0.8352276131100446 0.9979332184609212

0.9966801800410635 0.1680238987839866 0.9965403407057692

0.8304193793999255 0.0012950923496453 0.9941475206665021

0.4956637722893264 0.5009535583984838 0.0190829248821616

0.6641591335242959 0.8355210540698939 0.9964935766538370

0.8305679180254663 0.1683875594032140 0.9948379899367722

0.6633713888783701 0.0014839752290712 0.9905032662545686

0.4967685548347422 0.6697213404594866 0.0020690518024869

0.3300159249631570 0.3349117087176566 0.9987170392308693

0.3300247557297276 0.5017591939721683 0.0113252290801230

0.6636130787525194 0.1678393219513287 0.9912090856377135

0.4968395918185746 0.8347600664861659 0.9897675318825264

0.4972307059855557 0.0018895444906953 0.9863189532696167

0.3306647389551699 0.6690099915318555 0.9939765560941137

0.1631112987273552 0.3340119779311018 0.0044150462057698

0.3298908682445182 0.8350090524260640 0.9869340931988362

0.1625663950951468 0.5021552885721920 0.0031420441201541

0.4968928966238675 0.1682838638812661 0.9886140180116791

0.1634460149444797 0.6679271949809910 0.9927635161307193

0.3302432813779993 0.0014591796514029 0.9857174390190332

0.9967829567132237 0.3344896454347767 0.0006165419191709

0.3307717870989562 0.1686560463223190 0.9894156899191641

0.9972391799147975 0.5021067359656110 0.9982642509018302

0.1637745728042132 0.8352053901231408 0.9889364283923219

0.1635920612841694 0.0019402994313561 0.9887991513925847

0.8302064036729032 0.3348328606963628 0.9976778666116644

0.9964516031214257 0.6682809638925401 0.9961780889973467

0.9971198835324941 0.8348975188482467 0.9938363999511566

0.8309035910333993 0.5013523934702725 0.0021508800112396

0.8307185967600176 0.6690522248499549 0.0027460880120693

0.6642128637933761 0.3349210788395189 0.9990693162333832

0.6653143430109373 0.5018847070738136 0.0160762270854333

0.4963241734868982 0.3327723941217609 0.0003583378594597

0.6651246571796949 0.6694809986720656 0.0095212044454403

0.3989331312206028 0.4290302302177951 0.2360659556521818

0.5007870688600898 0.5490040969423259 0.1318910793284601

0.3052547886790043 0.5035649340803331 0.1287311279627830

0.6014587557670404 0.4487504040322255 0.1308644001247563

0.5303744030423547 0.3762520932946183 0.2556413759163452

0.4103484164280195 0.6044970346504289 0.2405622526585551

PT6(no-D)-XXIX – ΔE = 0.82 eV

1.00000000000000

14.7772044699999991 0.0000000000000000 0.0000000000000000

-7.3886022400000000 12.7974344700000007 0.0000000000000000

0.0000000000000000 0.0000000000000000 18.0009597799999987

C Pt

72 6

Direct

0.8918197457564148 0.6121426847154083 0.9957522866766837

0.0582546477824408 0.6121727967467478 0.9923941068794591

0.2245559985142265 0.6120541566293838 0.9933355410393361

0.8915353297712443 0.4454494454806976 0.9908065141844276

0.0579112824905792 0.4454319095982271 0.9939233679569313

0.3904496011960177 0.6124282415671587 0.9968081208631148

0.5567817598441280 0.6129569392516885 0.0032936862242181

0.2245965576081161 0.4458530354865644 0.0013831967161124

0.8915940868976904 0.2788896690537328 0.9908861403349576

0.0580221898574536 0.2788056255788973 0.9970871045004515
0.3913697985830566 0.4469004537652026 0.0136676668367173
0.7255888283824561 0.6114504979385345 0.0057164984827551
0.5590680094912983 0.4441109489580768 0.9949538426298830
0.2229357801323317 0.2778856706109138 0.0062697777253931
0.8915163812545757 0.1123257828878508 0.9930187119831544
0.7251413419445214 0.4451497017268125 0.9909072128550918
0.3923555689346472 0.2781065132157750 0.0083572302118142
0.0580515580949807 0.1122314519787153 0.9964573748806558
0.8916371083346988 0.9457446443075739 0.9953927302677670
0.5585896798500158 0.2786175495905781 0.9909253604631374
0.2242443865718329 0.1113301929411818 0.9982432838893587
0.0578495858125763 0.9455602570378545 0.9947668873797468
0.7249226462883414 0.2785872461715684 0.9879660294599120
0.3913852334707144 0.1117046390432108 0.9942736574154196
0.2248859954014577 0.9454741242562719 0.9926573368673672
0.8920179574791547 0.7793527121319102 0.9988797100392546
0.5581459704803891 0.1122582515376962 0.9890492083027667
0.3915943699118714 0.9453610589558963 0.9898327941220728
0.0582881641940460 0.7787771597281790 0.9938661325399920
0.7248940866407736 0.1120834389986598 0.9892532238236313
0.5581247842306922 0.9455676588710844 0.9899510872697093
0.2247351725826974 0.7787748878256622 0.9909542831050331
0.7250220027561554 0.9458588237590106 0.9931593353805255
0.3915208909529326 0.7790470969987240 0.9906112262391105
0.5579731603832059 0.7788699351056394 0.9948007450744711
0.7256931076745019 0.7808149080861497 0.0020140100676613
0.0030205113804200 0.6679151054890440 0.9936884662672938
0.1692737380388607 0.6677438684142984 0.9918683551153009
0.3356047364217716 0.6676259373831570 0.9928275307865988
0.0028151378806172 0.5010079585027398 0.9922287606635365
0.1692567713196311 0.5011016551686964 0.9959502411545813
0.6696577080121031 0.6687874009785801 0.0098646057594252
0.3350172977536374 0.5020831944135580 0.0041044457293822
0.0025072957416370 0.3343692271787972 0.9939259525153545
0.1686717780443345 0.3338841199390430 0.0023341064008164
0.5032731193256907 0.5008550607342741 0.0051166600788903
0.8370086966985539 0.6678735456027027 0.0011891170381091
0.6694711946742302 0.4997241615685724 0.9949019099443035
0.0026122081837912 0.1677579984108704 0.9955970501154567
0.3348262077993809 0.3332626283816111 0.0149845874698826
0.1685011192611086 0.1671199009531819 0.0001970271127689
0.5040822808312839 0.3344768143626808 0.9959177138422390
0.8364274004715142 0.5011310083978913 0.9923017885874899
0.3355881798071394 0.1662674118611349 0.0008162896984132
0.6694925190513175 0.3340518052556334 0.9884987070636768
0.0026980108586159 0.0008675400317983 0.9951112006767886
0.5026979261807938 0.1673237767568168 0.9913382395205943
0.1693774296350341 0.0011284504198272 0.9944959470500265
0.8361301230240130 0.3344233035838613 0.9892813206254942
0.0025077624534049 0.8343962161261231 0.9954619872115060
0.6692722532398250 0.1676827238646084 0.9881876311300317
0.3357797699942253 0.0007189442855235 0.9917236430748488
0.1692742358086292 0.8342679142433767 0.9920845628806347
0.8359946454817830 0.1676544536937854 0.9907788028196691
0.5025009767003397 0.0007070307296928 0.9892996690630795
0.3360169184209383 0.8342657962120938 0.9899305585969316
0.6694446793328552 0.0012072453430960 0.9904011270854483
0.5025818331055234 0.8343950918578216 0.9913034712717064
0.8360613740617993 0.0012221575525686 0.9938068674973408
0.6697199731656269 0.8347903065665179 0.9967003451184269
0.8364976714116281 0.8350811485102057 0.9986211216485117
0.5020246655863971 0.6683689409448732 0.9972413116608720
0.4738507126889431 0.3941612131513139 0.2425545994808473
0.3336151677233445 0.2136624538978751 0.2541875674340943
0.6807523807505333 0.6252676464925457 0.1223571962825645
0.3534794040197227 0.2855153069981640 0.1252752023640369
0.5955247848842831 0.5834300330121849 0.2490208206183127
0.4824210027071700 0.4983146469845536 0.1219624718268832

PT6(no-D)-XXX – ΔE = 0.83 eV

1.00000000000000
14.7772044699999991 0.0000000000000000 0.0000000000000000
-7.3886022400000000 12.7974344700000007 0.0000000000000000
0.0000000000000000 0.0000000000000000 18.0009597799999987
C Pt
72 6
Direct
0.8983612201591669 0.6174546545998894 0.9914084588762293
0.0657415787553006 0.6174523725712930 0.9905327985384318
0.2319265771454226 0.6169078227975007 0.9921825048659443
0.8984242574843577 0.4499308375904079 0.9910829552750755
0.0650365549996010 0.4505985796102010 0.9916916563768368
0.3982904280295188 0.6174988844505407 0.9977024507429135
0.5659747128626691 0.6175042187895983 0.9994899948430813
0.2323803545950227 0.4511220505752505 0.9994224301794006
0.8992488641449725 0.2839698800345332 0.9912232312163383
0.0653028294199132 0.2834711011488267 0.9956166738940055
0.3987847732569278 0.4522263646228808 0.0191631927440454
0.7319086479117871 0.6170717936312400 0.9944220727726005
0.5663636125093205 0.4525851979384754 0.0053954209389531
0.2300133895630623 0.2834268276474532 0.0099966632188071
0.8986746041752482 0.1173962699723745 0.9927718227188222
0.7326007350924684 0.4507666011151414 0.9954968754348172
0.3988668612244481 0.2821319225379497 0.0211501230207674
0.0657887725085402 0.1174080281086702 0.9986092052237865
0.8985791058446821 0.9501964702431849 0.9929718743520084
0.5662481179823899 0.2829509594262873 0.0032535405278225
0.2314260297684427 0.1162310303775413 0.0055334142376324
0.0649975999245953 0.9506861434242637 0.9956676335711023
0.7319189092010987 0.2838023882830143 0.9930601024158250
0.3982328432335009 0.1167226072889420 0.0014653032921785
0.2323225959961022 0.9506917810718605 0.9959564292622431
0.8990725978894432 0.7841658380724965 0.9920385946754635
0.5657730555651455 0.1172860821354433 0.9944077239243825
0.3986671314300736 0.9501208135341912 0.9930391802642191

0.0650864822379376 0.7835324027012405 0.9920876932016398
0.7319237805972065 0.1166885456763680 0.9914856283181450
0.5651162803631564 0.9505251769333540 0.9917189350462152
0.2318498987548665 0.7841659102367657 0.9914673993572336
0.7324635848384204 0.9506480594536555 0.9917852236643938
0.3991649758427513 0.7841349850516153 0.9917960466158533
0.5650511532638802 0.7830836653748818 0.9937485259589423
0.7318260526683531 0.7841049585378315 0.9927491974462157
0.0099878465057373 0.6732196690759267 0.9908931759149553
0.1760493954206908 0.6726553197358598 0.9908431864065363
0.3431904330862849 0.6725182727216463 0.9931774180857147
0.0099850323303770 0.5058528351263334 0.9905986298320641
0.1768919587417059 0.5065225467126737 0.9933963785261568
0.6763064777583239 0.6726418808396204 0.9950728143252121
0.3424110884978546 0.5069268803508251 0.0043284854750070
0.0095943480027358 0.3393209759573423 0.9922499779552894
0.1762433636541871 0.3389610863638666 0.0013594930601428
0.5114429067527695 0.5070857352324509 0.0072427416723642
0.8434494373455479 0.6726224837092687 0.9920219718093790
0.6767099109855650 0.5068194214200261 0.9975750300594655
0.0100925498551732 0.1731346497985342 0.9956122897388084
0.3410859358684419 0.3395991422123927 0.0244254232428389
0.1754960312350065 0.1722879994452455 0.0054396380815973
0.5115513328395522 0.3397553066339754 0.0154550563227147
0.8423738636861984 0.5057113725903051 0.9922064374452049
0.3428440141636955 0.1712623204310475 0.0091074983052906
0.6770732048720376 0.3390549121448174 0.9966990044717079
0.0100096493256387 0.0057619249776977 0.9955449668575724
0.5103799938622444 0.1726661782407959 0.9998081951544506
0.1767681064730198 0.0066271597294758 0.9989555194004680
0.8436357010182221 0.3398807982582213 0.9912518421284560
0.0095438602520233 0.8393325024836287 0.9928867255451905
0.6762849696408537 0.1725173207740198 0.9927659998780385
0.3428621007528037 0.0059855568632869 0.9962047074185989
0.1767528161021232 0.8394749675988109 0.9927652037213761
0.8435362099445456 0.1725722840237935 0.9915636083383959
0.5099136162923017 0.0055905165751966 0.9927147972442754
0.3433829397615966 0.8398745034994448 0.9916403063148209
0.6768022385745525 0.0064356406281618 0.9915639053224723
0.5092977042818490 0.8390871597351079 0.9921687132269525
0.8428721496896259 0.0058472669170868 0.9924065257353760
0.6767537317245882 0.8391129653921325 0.9926222551607395
0.8434892944781396 0.8398297502431475 0.9922119841145474
0.5101641801798991 0.6734127403731165 0.9974653774727074
0.4273695043609393 0.3702870563443597 0.3760366868050298
0.5048088513487770 0.4039707387951097 0.2503037274102056
0.3394014374581218 0.4175621153519913 0.2791170365685360
0.2515975826734760 0.4704922736794614 0.1892753841786572
0.3644919776001672 0.4010803093340911 0.1381579800924442
0.4728927857230545 0.3090371209592959 0.1316956009724919

PT6(no-D)-XXXI – ΔE = 0.83 eV

1.00000000000000

14.7772044699999991 0.0000000000000000 0.0000000000000000
-7.3886022400000000 12.7974344700000007 0.0000000000000000
0.0000000000000000 0.0000000000000000 18.0009597799999987

C Pt

72 6

Direct

0.5595464435861536 0.9417286574075115 0.9937761725906035
0.5603696287365860 0.7768097942859706 0.0050876266836228
0.5619527677996259 0.6093806580626926 0.0115141517448085
0.7266092813137570 0.9422591395630450 0.9953584559667438
0.7265217504088757 0.7756705849996952 0.9997178966046746
0.5605390422515069 0.4408844518076646 0.0065041398848753
0.5595987637997963 0.2751794372204635 0.9954200770081969
0.7265135309633142 0.6081634611862170 0.0000884734669166
0.8928334997475945 0.9418536343287165 0.9942578935456247
0.8928563835132977 0.7750895351583175 0.9949873702348547
0.7265958293274573 0.4415873222523921 0.9964609814025092
0.5598235897786129 0.1086294563554817 0.9915733232589106
0.7264652647531479 0.2751397815543292 0.9930721240404784
0.8927735081293174 0.6082255057455793 0.9947980863485690
0.0593823441130998 0.9417148403410128 0.9927681951371091
0.7264752382696544 0.1085024526863057 0.9924684130357864
0.8930739407747349 0.4416938485627367 0.9941888006095965
0.0593260429618234 0.7749339420467418 0.9929288115751264
0.2263054224926916 0.9418689326217031 0.9916860053554469
0.8936351809667149 0.2754358243617427 0.9938430378747606
0.0594812706108089 0.6085012225711439 0.9946985237819073
0.2259991690188186 0.7751648260928846 0.9939724303348781
0.8931686662693631 0.1086597356207986 0.9936162336149668
0.0600031646311188 0.4421624401285951 0.9970424153156672
0.2258036324777536 0.6087805255439606 0.0009697206878371
0.3930698655960285 0.9420109397247909 0.9917962725478729
0.0600698851117642 0.2751835946833377 0.9969258955193309
0.2264115343579855 0.4419019494497191 0.0041278625667445
0.3926538007149240 0.7756360673142375 0.9988302734774257
0.0595860369758492 0.1083807797104028 0.9942851391516787
0.2259876911792631 0.2745111317002899 0.0009050547823180
0.3912651792873225 0.6086773907393604 0.0170355837994746
0.2260873570356213 0.1082724011471479 0.9941002183617443
0.3912567811702307 0.4398784822454851 0.0189355364896104
0.3926263569051960 0.2746144312049836 0.0000045949400511
0.3930513844685044 0.1085101865984549 0.9923969818407935
0.5037482411569143 0.8311747937689186 0.9984783620549070
0.5051073726194506 0.6656290128589880 0.0164762442480512
0.5053887899884675 0.4968723528736226 0.0175018361726168
0.6708354286490711 0.8310670571275500 0.0000520709964446
0.6705402954887700 0.6637873152109075 0.0031712483425835
0.5035384895442601 0.1636486655988535 0.9926445243278651
0.6707546318071106 0.4968223559555900 0.0009793538175507
0.8367460343236174 0.8304444532164073 0.9959502594242070
0.8366780327121280 0.6634220280883251 0.9962614440733771
0.6705143786133299 0.3303665714207564 0.9949129506414188
0.5034527945607414 0.9971070522248418 0.9917918344988496

0.6703417691489425 0.1637383878644108 0.9920752071086570
0.0031102434227085 0.8301238646465949 0.9931592555900504
0.8370905472299908 0.4970221290129815 0.9948482131207953
0.0034648882183529 0.6638051798731658 0.9937338197528303
0.8372614418543782 0.3305783193517673 0.9935726431786832
0.6705483373087545 0.9972728186869747 0.9936596540414169
0.0037938241528102 0.4972758135203538 0.9951900409555381
0.8372725813415793 0.1637357096654901 0.9931862515502136
0.1700911913085049 0.8303326519634014 0.9923810595616772
0.0038075600044465 0.3304603544261952 0.9958763853408893
0.1699668699056053 0.6639840966895756 0.9960170376094510
0.8371677426139789 0.9971893077237036 0.9940724376954287
0.3368951016014810 0.8304432779246766 0.9940542352765754
0.0038857095890141 0.1636980584362653 0.9947827449731053
0.1701135812895702 0.4975196641364088 0.0008253643063043
0.3360793416845725 0.6646168186826803 0.0056847210392586
0.0035619157929745 0.9968752065263728 0.9933517545671205
0.1701020626301054 0.3298258148819481 0.0008801260288845
0.3346704812842833 0.4959338556743562 0.0122257872825423
0.1701046177267314 0.1632944990762950 0.9958045326516169
0.3361347789966898 0.3287126209750718 0.0062176300346695
0.1701696176324643 0.9969806055817804 0.9923735882583221
0.3369243898826356 0.1637928244124014 0.9947704821557793
0.3367983827130874 0.9970820144144991 0.9913338418211187
0.5038212951522721 0.3299444037969749 0.0005231388736592
0.4539811958268949 0.4754964672446109 0.1325443983220456
0.5322084649806303 0.5305172384792166 0.2665014359218356
0.5192183337184946 0.6976061753157214 0.2640488300081724
0.4562941627822654 0.6526483225057204 0.1305690376808798
0.3749520060074924 0.3468339100519812 0.2440821364260025
0.3537858757759583 0.5139175401697775 0.2438119287158280

PT6(no-D)-XXXII – ΔE = 0.84 eV

1.00000000000000
14.7772044699999991 0.0000000000000000 0.0000000000000000
-7.3886022400000000 12.7974344700000007 0.0000000000000000
0.0000000000000000 0.0000000000000000 18.0009597799999987
C Pt
72 6
Direct
0.8850316175045876 0.6092217298894838 0.9948375456537093
0.0515438646811575 0.6090987695291972 0.9890045875907205
0.2178715630681936 0.6087447489947095 0.9895794464064096
0.8853375282832445 0.4423189187558947 0.9946873083880163
0.0513416854494224 0.4420953746999245 0.9883224280191669
0.3840332588328224 0.6092768793085241 0.9985381045541004
0.5520586517351660 0.6110439839613946 0.0143015413649721
0.2179914873765796 0.4422117878988772 0.9908233217154958
0.8848569697276929 0.2755323116200046 0.9922589007368359
0.0513252048324517 0.2752820932400581 0.9885959470764405
0.3827572798983709 0.4416040553469287 0.0038865016192204
0.7183543388913023 0.6091172096374962 0.0014894879395726
0.5520712262509235 0.4430378703421951 0.0202434700734102
0.2177708468407218 0.2755558594188514 0.9931597526551030
0.8843957714421435 0.1086173253040954 0.9909584439670951
0.7198839676684301 0.4436797287649696 0.0107051654503749
0.3829773017280687 0.2745243505062902 0.0062450447055653
0.0513178182753009 0.1088995438162996 0.9895634789120180
0.8844605015033693 0.9417953372896477 0.9912287176660826
0.5498372252340573 0.2730890581851426 0.0093166712737442
0.2175570396623172 0.1086545642675318 0.9936061282787207
0.0511090354685919 0.9422526047415403 0.9897216312011992
0.7188813479056506 0.2748601781470370 0.0041026656818133
0.3840916728164032 0.1085960531531853 0.9987594066662950
0.2180228965278417 0.9424622058129657 0.9915749963251628
0.8847831282493033 0.7755502638815486 0.9925848096844447
0.5517316195019859 0.1087476981462174 0.9992119123153316
0.3847323917226717 0.9423161911839628 0.9941700212384035
0.0513326040505220 0.7755764683995992 0.9894520812867924
0.7181293082633857 0.1084414208803253 0.9962144625150771
0.5513925265520569 0.9425448282784217 0.9963608785606937
0.2179660353233359 0.7758227340545929 0.9899161871447859
0.7179628298622802 0.9419480994171678 0.9952786344229025
0.3848446977417197 0.7760089396354672 0.9942608433255131
0.5513018642253442 0.7762706636357137 0.0006165714253754
0.7179891410943000 0.7754862305772434 0.9983647539398177
0.9963586979447996 0.6652363250570303 0.9902908654786202
0.1626954278847208 0.6650645519785812 0.9887596351792993
0.3293485603121908 0.6648607768371022 0.9933758679761340
0.9960958498942318 0.4982257197805069 0.9896621254462872
0.1627992156567544 0.4985261440683146 0.9886727719483943
0.6639036651194772 0.6662714220769175 0.0042986718859339
0.3285482297929718 0.4978653618167854 0.9968031456555337
0.9961399894960721 0.3316645018883833 0.9890195596621538
0.1626219652055809 0.3314387735351048 0.9900491192312728
0.4938178148858441 0.4986026496320406 0.0173016302925433
0.8297555466454725 0.6650154914815687 0.9957725457799995
0.6642274307569878 0.5001027144678503 0.0081051468637767
0.9960870140721241 0.1649147349017497 0.9891948022013395
0.3285655099801517 0.3314510582655004 0.0004648209970455
0.1623768138574917 0.1647358112760813 0.9917566279202319
0.4940721908987129 0.3292360683941524 0.0171102460759442
0.8306475438250729 0.4989413997539174 0.9998142553170410
0.3288297908102464 0.1647205919774564 0.9995091982757458
0.6628615993194629 0.3300546246414697 0.0127580893195187
0.9959910134488652 0.9979439268382251 0.9897217086157681
0.4957023279511716 0.1644769617618564 0.0017452109594558
0.1627170164491787 0.9983854847384990 0.9911107821842293
0.8301438229727225 0.3316321913565830 0.9967893514587729
0.9959530993702401 0.8314767432624990 0.9901377637732054
0.6626963420009631 0.1641841027992825 0.0002314695275629
0.3291897319259718 0.9985207770430335 0.9944297606661507
0.1629123802386658 0.8316990683039265 0.9896823888004462
0.8294119152309491 0.1645865923884031 0.9931517219534598
0.4961215756475639 0.9983657526173957 0.9962348178017422
0.3295908350049359 0.8320599407871043 0.9922772751428042

0.6626159751311462 0.9981335858757703 0.9960306586156165
0.4956645876704897 0.8319412479632788 0.9968932066635787
0.8291219995122958 0.9980015553518271 0.9923201583499690
0.6625017420399004 0.8316718007994339 0.9985131132819447
0.8295563266468449 0.8316890608267897 0.9934077221966646
0.4952239218973205 0.6662710139668206 0.0055713287575045
0.5145297435241361 0.5703349335365715 0.1308709009284286
0.6919708201604706 0.3709000379464058 0.1289629099848639
0.5367252717266879 0.3982906988398298 0.1325426934578076
0.6011025716220360 0.5278571103762886 0.2447553912569305
0.6241750276356868 0.3732131054011631 0.2577684296183236
0.5371334596665776 0.6508155852378579 0.2576670681825988

PT6(no-D)-XXXIII – ΔE = 0.85 eV
1.00000000000000
14.7772044699999991 0.0000000000000000 0.0000000000000000
-7.3886022400000000 12.7974344700000007 0.0000000000000000
0.0000000000000000 0.0000000000000000 18.0009597799999987
C Pt
72 6
Direct
0.8901722669302430 0.6130953102477648 0.0020266701588696
0.0565223262155854 0.6125905516199168 0.9982759796646477
0.2227931185634020 0.6127610890773028 0.9972257823371109
0.8896561478493297 0.4457324607848747 0.9979310683018312
0.0565289101322790 0.4462510183116564 0.9974669243412748
0.3895984628927067 0.6131957493640101 0.9993583674435271
0.5567918064971877 0.6129709535954717 0.9994403702798422
0.2231826367037613 0.4464285306920530 0.0010814678406774
0.8900184126807886 0.2792149415385552 0.9949811428524171
0.0563810227211903 0.2793360631911526 0.9984316431459064
0.3897659694692095 0.4470698064723990 0.0125235087233477
0.7237608567067610 0.6139859138861183 0.0045566884042074
0.5563493214346593 0.4456579173478232 0.0091384194231878
0.2222468823357175 0.2790194652978002 0.0046731912468090
0.8899922771947359 0.1129137166020300 0.9948338063182121
0.7240096888129059 0.4453769422932083 0.0032965790978565
0.3899697825957702 0.2781586612092255 0.0010808938464208
0.0564364528874606 0.1125738969996206 0.9980252861076693
0.8896552890262583 0.9460893166181066 0.9959197240481998
0.5562865566264605 0.2788728923690584 0.9954841253272235
0.2227523995631060 0.1124129980957633 0.9981345238835573
0.0565850157363244 0.9463541873676533 0.9971860091451745
0.7234414547998256 0.2797276458250337 0.9937341653514054
0.3899541777281215 0.1127438004720815 0.9934693498968059
0.2232137915167840 0.9460057350296651 0.9950058822009282
0.8900698502062596 0.7796065972281596 0.9988815158713464
0.5567030673718278 0.1125792962963246 0.9908046349791704
0.3896319998410007 0.9460278930236399 0.9919416629642370
0.0562535552125922 0.7794706602478669 0.9980704331057240
0.7229327286918377 0.1125562474381212 0.9916612110228016
0.5566679813017501 0.9462273073192193 0.9912451620085037
0.2232568487488322 0.7797349132446456 0.9953819003063131
0.7231508734840446 0.9456240730202907 0.9928486955674032
0.3898572442011172 0.7794464663761218 0.9938547069104189
0.5560507213550849 0.7790588484574599 0.9943195689292850
0.7235698115885256 0.7796286545292332 0.9959845572953512
0.0003936427876852 0.6683384634862364 0.9995594335273097
0.1671020413802893 0.6681610937231213 0.9968806658220331
0.3340842068645600 0.6686799498378591 0.9964739027089422
0.0010963692704564 0.5018178546121987 0.9978171568907399
0.1672758129635668 0.5019359879864922 0.9982568744125473
0.6682433854239491 0.6685552229749092 0.9990325099642305
0.3330213470374161 0.5023426802093311 0.0042052783259123
0.0006738653519207 0.3347775748155613 0.9969072442626228
0.1673138060257244 0.3349003883361519 0.0017457385553996
0.5020408275441355 0.5038592522010745 0.0053308743503990
0.8345170041088537 0.6688351129804957 0.0020394170069338
0.6682946237255862 0.5019915090786640 0.0120018806788096
0.0005894117671303 0.1682718705498800 0.9973049407114729
0.3336008923947773 0.3339027210510324 0.0101173157091807
0.1669733829880755 0.1677715615639741 0.0003072068818568
0.5003401898837581 0.3331435869891806 0.0005470611301277
0.8340772590778514 0.5010994457890234 0.0013216714773918
0.3342209499127122 0.1679204000009591 0.9970428364648214
0.6681982433377414 0.3351328411572823 0.9967560308010093
0.0009519243397946 0.0017091852205340 0.9969812725888190
0.5007314615030225 0.1682814771955705 0.9923762320852134
0.1673698755829278 0.0017893334554415 0.9966033591553796
0.8341937291107513 0.3351009798380957 0.9954087525143578
0.0008140111144002 0.8348837080166973 0.9979616574458348
0.6673259614899223 0.1679448920449502 0.9913459155352911
0.3339855253952138 0.0013741007050498 0.9930148934598293
0.1675639750506335 0.8352345657872178 0.9959792338213234
0.8343610975785083 0.1684277959757949 0.9935856523077007
0.5009194889773454 0.0017072525157360 0.9908070424836666
0.3338272218898695 0.8350886635219155 0.9933777902566092
0.6671933017079610 0.0015485052899038 0.9915171816269250
0.5004392395049351 0.8345745008430612 0.9928508497823856
0.8340337869430954 0.0012839853254007 0.9942796843813468
0.6676912260933392 0.8350807430417291 0.9938406833845406
0.8342458822420582 0.8351380029539754 0.9969071861066041
0.5005939453029100 0.6685275964975474 0.9975767806202285
0.4141465569214731 0.2906498061553009 0.3348568892630439
0.3803260201148362 0.3933554354807640 0.1270846940445338
0.7376964155551136 0.5051986002838262 0.2126967197042902
0.5834910717819142 0.4556897532011348 0.1274941477496938
0.5637190228131317 0.4332919103258632 0.2664680885669739
0.3835244575352732 0.4137026296163242 0.2623852110125569

PT6(no-D)-XXXIV – ΔE = 0.85 eV
1.00000000000000
14.7772044699999991 0.0000000000000000 0.0000000000000000
-7.3886022400000000 12.7974344700000007 0.0000000000000000
0.0000000000000000 0.0000000000000000 18.0009597799999987
C Pt

72 6
Direct
0.5497924775028071 0.9436526828817564 0.9947915537313071
0.5494420457854031 0.7770095073886065 0.0004610387728639
0.5501739588606686 0.6122238113420289 0.0190354797081577
0.7163113902277731 0.9430109873418532 0.9965018337084375
0.7166882124976039 0.7768329380899532 0.0019733584203988
0.5508343049945728 0.4433530044042597 0.0185657327185553
0.5500523006738521 0.2753657189100386 0.0044595266224885
0.7167837651464608 0.6105542369782033 0.0035817452721528
0.8829779740778071 0.9433141045639256 0.9938764359872776
0.8832041740880958 0.7769867226541380 0.9947965860304804
0.7167708374499490 0.4438299601008128 -0.0007353992629650
0.5499725111145168 0.1098440560123257 0.9958777575649012
0.7163577982042069 0.2769236192788720 0.9948111105950084
0.8829547918984756 0.6102628263788332 0.9929873768462006
0.0496467851450653 0.9436038112088124 0.9915831578336067
0.7162542561861885 0.1098032322524172 0.9935074805796134
0.8828854683746662 0.4432196762846827 0.9908749050655755
0.0494584112520349 0.7766730140073077 0.9906368788402253
0.2163560284748185 0.9434519106532006 0.9923174411845475
0.8831231459701880 0.2767446190618886 0.9904569076795791
0.0496843153928705 0.6100265210814314 0.9891632472714548
0.2163357760605115 0.7768855065871860 0.9905385709318530
0.8829457854237210 0.1100360240361835 0.9918334924613338
0.0498297211073186 0.4434899023752469 0.9888262965645611
0.2163210964384488 0.6099552271361730 0.9902751554627014
0.3830043961368810 0.9432812586032585 0.9935984045404528
0.0498015752571319 0.2766651685554224 0.9900449935336080
0.2167466577826401 0.4435535807621934 0.9925138715725662
0.3831696667747559 0.7769137864298878 0.9935956637637237
0.0497957508633493 0.1101909582718044 0.9913088761734077
0.2164020534614013 0.2768692451118201 0.9943584695042502
0.3831789825992537 0.6107385848834457 0.9985044069746559
0.2159734586783662 0.1098224922000135 0.9941399882819916
0.3815237154017128 0.4428349032947561 0.0057957446199433
0.3814904084927379 0.2754431351292163 0.0048451234659682
0.3829623104676277 0.1098395900397457 0.9981132137669622
0.4941164902071044 0.8328606494160301 0.9957546562906855
0.4937641012909413 0.6674096380929916 0.0051818353278135
0.4926236439529610 0.4997596444521096 0.0206241337564954
0.6613958617896547 0.8331776380040153 0.0006133129314270
0.6626098641444913 0.6674391147740265 0.0086272327677262
0.4944845020587301 0.1656563740170418 0.9994858868551219
0.6623828366534282 0.5000789799823820 0.0062097109486162
0.8281096803840641 0.8330207827591571 0.9965902706352316
0.8279127392023655 0.6662883717895808 0.9963763096263354
0.6614795296270550 0.3323719953026545 -0.0003313394982437
0.4944866235770607 -0.0007140149055319 0.9944291852864614
0.6609220140304178 0.1658216069698159 0.9946555484163694
0.9943628586701313 0.8327648057738802 0.9919037349713470
0.8275336428795871 0.4994052929203886 0.9936742407453656
0.9942832185078919 0.6662407178667400 0.9903959760590302
0.8279816684794400 0.3330317879505861 0.9916301495293403
0.6610110792981374 -0.0005024519732304 0.9949778182514157
0.9946043995744268 0.4996038067863391 0.9890611375582349
0.8279642966856429 0.1661560329280357 0.9918519407903117
0.1612981854300114 0.8329938895597075 0.9906954654985906
0.9945542700300448 0.3328899131600474 0.9893740381350904
0.1610965440041727 0.6661562715019866 0.9894474319243857
0.8278025649706295 -0.0005901132679303 0.9941471763893975
0.3277695265443879 0.8330000858580479 0.9921518850374029
0.9946214325776342 0.1662593073589243 0.9907328713280401
0.1613541836188797 0.4997290400358579 0.9898641024819732
0.3280871199669030 0.6661837401986794 0.9932612360231443
0.9946478377131323 -0.0005327405038100 0.9918597820374968
0.1613751810605078 0.3328144312365549 0.9918401785770374
0.3274595279204553 0.4991592931715153 0.9984429863272968
0.1610178170523054 0.1660043895248161 0.9928833327454611
0.3274691010290418 0.3326642889249152 0.0010179607372722
0.1611357341796531 -0.0004174081711680 0.9922158771735439
0.3276217099000746 0.1661078747382798 0.9988430149371926
0.3278144788680539 -0.0002973881910686 0.9943706194823688
0.4934826819638267 0.3305767976438572 0.0144388536665808
0.5279952518213180 0.7225992208407152 0.1882674159293080
0.4897980024526198 0.4642102671528323 0.3747196471988963
0.5298841714765755 0.3861101869682706 0.1304536612532094
0.5163253903142141 0.5947956893373884 0.2781699031482901
0.4498915443943781 0.4008599759774062 0.2455760304784873
0.5182636932720934 0.5575222797091098 0.1360998544590156

PT6(no-D)-XXXV – ΔE = 0.85 eV
1.00000000000000
14.7772044699999991 0.0000000000000000 0.0000000000000000
-7.3886022400000000 12.7974344700000007 0.0000000000000000
0.0000000000000000 0.0000000000000000 18.0009597799999987
C Pt
72 6
Direct
0.8874562061865916 0.6067602960470850 0.9975748565671978
0.0537799441745150 0.6066288064364684 0.9927927183965437
0.2202554689797793 0.6069012233624491 0.9928447848527000
0.8871294736513704 0.4401574729899806 0.9922281777461848
0.0540467974182164 0.4403064067554681 0.9927282560754236
0.3869452256573425 0.6073037506090557 0.9993671080986317
0.5532484651133274 0.6076088045755696 0.0169832865220627
0.2205689071107315 0.4403994937987932 0.9983599103653233
0.8874218324770098 0.2733584724276099 0.9918413102507557
0.0540554320117366 0.2736594480402772 0.9961593300954164
0.3855067626568811 0.4397661520222869 0.0123688276346030
0.7215376156025428 0.6064973167645036 0.0084662396029742
0.5547512764748674 0.4392868358212496 0.0038994645471320
0.2199333123043630 0.2730696959858605 0.0019823000854942
0.8874312101126733 0.1069710771770644 0.9953544360731286
0.7206904027942471 0.4400142105322402 0.9953960910776871
0.3870536732180341 0.2726551894870752 0.0002409491443913

0.0536636279248341 0.1066356368620802 0.9982322404655903
0.8872430203066841 0.9405842868936034 0.0000565053104680
0.5539370172425038 0.2735743411742177 0.9924842792055273
0.2202716394736086 0.1068216892999700 0.9976206952715145
0.0539713369438815 0.9404255298737709 0.9986556739877344
0.7208624828260497 0.2737591078582682 0.9902218661514866
0.3875529138250400 0.1069163965611679 0.9925192911879819
0.2205066426731506 0.9400560543515866 0.9941186767435184
0.8874160153182373 0.7736044072543251 0.0034116869811882
0.5540246708916214 0.1067429080130111 0.9895088868010120
0.3872258802930304 0.9402481118023545 0.9905155672080781
0.0536914859086295 0.7735013235724324 0.9963810394138619
0.7205418511282673 0.1067988389641172 0.9911063867849208
0.5541693015769766 0.9402778525106186 0.9914634152554314
0.2206295413839570 0.7735889447621318 0.9919362282553337
0.7205148672372630 0.9397906786005450 0.9966492214622846
0.3871151538391118 0.7734063884219182 0.9922302423314306
0.5534636757952143 0.7735611905286035 0.9991098729788987
0.7216661067648218 0.7751860553200487 0.0084024978445498
0.9977370052854795 0.6622408849624009 0.9953305258613696
0.1645980916942520 0.6623348933731208 0.9920701471153706
0.3312565602556887 0.6626209858101078 0.9939291045966954
0.9982088124484889 0.4959411090851376 0.9922042619818328
0.1643797838166989 0.4957840728982106 0.9940748753586774
0.6663646621529864 0.6633375686548533 0.0183611854981507
0.3300655968609192 0.4960787862300080 0.0029947652098343
0.9980582303558236 0.3289279380587331 0.9933723086733224
0.1645088078692467 0.3288537378035841 0.9992329244661136
0.4978888277728544 0.4941260030830250 0.0155113677044909
0.8313413507808747 0.6620634779599968 0.0033818211011365
0.6648729279221399 0.4953435596178579 0.0017719325128596
0.9978374903865301 0.1621587195297991 0.9967586748774551
0.3301455716108848 0.3273762104246316 0.0065028383042502
0.1643787190681607 0.1618384868517992 0.9992961507757840
0.4985977869033391 0.3287488968172454 0.9977617931359646
0.8314982721596706 0.4955261052135427 0.9948505172675510
0.3316196090074115 0.1626150127674251 0.9962963447057192
0.6649887325253587 0.3291821332191560 0.9919139353595909
0.9981827238947432 0.9958319819159911 0.9989518513027278
0.4977596228210857 0.1621139390008963 0.9907940592421269
0.1644028474312407 0.9956115846417859 0.9966348768166924
0.8312727848122634 0.3290051087552683 0.9909560652869018
0.9975933577991327 0.8286826143224175 0.9995456860940877
0.6646293401536241 0.1621302838944843 0.9896601342698617
0.3312883616941917 0.9955590590405379 0.9919283484898216
0.1645667043979699 0.8291856025927160 0.9938360068454273
0.8315244670439270 0.1624529201214102 0.9925466046881510
0.4980438235031599 0.9956380690522977 0.9898808334776277
0.3310684308575844 0.8288586278588781 0.9910017688893689
0.6644194530820329 0.9954638952240700 0.9925539357716673
0.4979531925608995 0.8286939097642616 0.9935271562572581
0.8315211663133155 0.9956508421031600 0.9975229342005676
0.6652043059098318 0.8296017542569771 0.0012463910045355
0.8315000803277854 0.8294398924373283 0.0042837143048544
0.4970200372035407 0.6629896482427426 0.0040422179359396
0.5950370605814186 0.6265553733865730 0.1307182210469264
0.5451275002375908 0.6787984054284308 0.3531903703133423
0.3509567568187322 0.3605064539057778 0.2534273996128107
0.4895153553565237 0.5437908224950476 0.2538121997491558
0.4220669478778092 0.4321337657009110 0.1280106726307508
0.6716242982185392 0.7085358758085079 0.2530746572839675

PT6(no-D)-XXXVI – ΔE = 0.85 eV
1.00000000000000
14.7772044699999991 0.0000000000000000 0.0000000000000000
-7.3886022400000000 12.7974344700000007 0.0000000000000000
0.0000000000000000 0.0000000000000000 18.0009597799999987
C Pt
72 6
Direct
0.8875514148452339 0.6047314356740046 0.9937877794418313
0.0536433475984026 0.6042571882763212 0.9926198626823819
0.2200254703569087 0.6044948219373367 0.9965124313985427
0.8869706432408222 0.4381129891798778 0.9929079645779026
0.0538684997410925 0.4379390310373523 0.9923294481100058
0.3858663394010691 0.6043243017345290 0.0067249026251019
0.5553451005092143 0.6058905095285283 0.0164172650851171
0.2201347143969272 0.4376987884938686 0.9955502764763153
0.8868069724479852 0.2711169046147219 0.9912014917903917
0.0537571828230625 0.2715909150823634 0.9902835712157625
0.3854701459243230 0.4371061599875929 0.0038802413877477
0.7210361680554342 0.6049320568612302 0.0004904431565933
0.5545800671353049 0.4365181840918737 0.0130152895048710
0.2207071523520625 0.2714724011420842 0.9923291052907786
0.8872942798490513 0.1047446456297649 0.9901381605605479
0.7205987355129011 0.4380168827348427 0.9987254448093736
0.3867077582528395 0.2702136956089802 0.9976602001438479
0.0536936936552266 0.1045048923347380 0.9888605020229591
0.8869752030610201 0.9381607545782913 0.9906529310027565
0.5535672245371188 0.2706794153541381 0.9981239992146627
0.2202012414125747 0.1046652510269439 0.9903203799365627
0.0539482600807730 0.9380210479679292 0.9893606795212975
0.7204408609747830 0.2715614584462429 0.9942278975849206
0.3874066957457742 0.1044339001066064 0.9933502583019447
0.2203983294568488 0.9376089481808663 0.9912403765944475
0.8871449946800780 0.7710360772258227 0.9923694302696546
0.5536931359356601 0.1042539478111877 0.9941131007304236
0.3871356742863483 0.9380452793070901 0.9945388675338762
0.0537003163244307 0.7713000716328295 0.9911810178422797
0.7202723963558242 0.1048778203805867 0.9928112884593077
0.5542268003210680 0.9384984834468924 0.9965462487348375
0.2206816516272809 0.7712083854179639 0.9940448498906420
0.7202404220070804 0.9376910958831743 0.9947428401474312
0.3870425166988767 0.7712696728082236 0.0000291279894427
0.5542155455740385 0.7732622499977353 0.0058882865303076
0.7212684222005024 0.7716435129281649 0.9987440948391750

0.9983012910657507 0.6600581863395121 0.9921961587174266
0.1651900032786813 0.6598839133906793 0.9941561699181634
0.3315519860218927 0.6599648534050004 0.0012866828698321
0.9984882851883725 0.4937415627035975 0.9923625970715477
0.1646003977250317 0.4932688412974429 0.9947362216561046
0.6667157359355471 0.6608613528861511 0.0043301141426255
0.3311627645782167 0.4931002069835344 0.0014891848714313
0.9984920898428697 0.3267386587864962 0.9909496764473005
0.1650266633249018 0.3270372754220645 0.9922184754637513
0.4968681945262432 0.4918290081324557 0.0155092599842988
0.8320307199809633 0.6603516657600181 0.9949094060518675
0.6663544922436984 0.4936289287649913 0.0043702447218550
0.9982428935617875 0.1599851464013256 0.9893139061712475
0.3308878644021505 0.3263445901161504 0.9973314128540807
0.1652080560568052 0.1598729971734230 0.9898328246153909
0.4979666587747360 0.3246884574740392 0.0031134174186747
0.8321510481789218 0.4935257507027160 0.9948191851084047
0.3318797874077504 0.1602913947790867 0.9930295259800346
0.6650765049408847 0.3267060171540663 0.9973701137363022
0.9984722383462028 0.9935132462953646 0.9890615389260162
0.4978875255913948 0.1594034203552113 0.9947730783094215
0.1648841548904372 0.9932374811311320 0.9897848683417436
0.8314465660483634 0.3267469373381715 0.9927262149616283
0.9984542650450479 0.8264668362636343 0.9904396409374101
0.6649612006346430 0.1598812046930362 0.9936668105108808
0.3318699859198517 0.9930848012245121 0.9926579696165874
0.1653448945482481 0.8268843357866515 0.9917796547384725
0.8317348105614570 0.1603706206466526 0.9911999441795558
0.4983650931688715 0.9936255311666997 0.9948340623714387
0.3316659148471075 0.8263537397435172 0.9960412660912255
0.6647086687710626 0.9934912955809310 0.9946922026848526
0.4986209695742545 0.8272821721146926 0.0008770843282022
0.8319514458497963 0.9933411943186812 0.9913179153401899
0.6654015591673925 0.8277644492342446 0.9998972647783404
0.8314535841451232 0.8265433567355274 0.9931798666167424
0.4976622969940721 0.6613124142199709 0.0156627372476095
0.4318005859676575 0.4750511057829243 0.2531402374462530
0.6257241019893058 0.6095181002095345 0.2501129384272716
0.4908339165691018 0.6713385665186777 0.2600788564231351
0.5402620442009649 0.6427328754061534 0.1293449924950494
0.5666467110865057 0.4128076697872842 0.2537794023881901
0.5123709735831952 0.4497607137006412 0.1272238953008227

PT6(no-D)-XXXVII – ΔE = 0.87 eV

1.00000000000000

14.7772044699999991 0.0000000000000000 0.0000000000000000
-7.3886022400000000 12.7974344700000007 0.0000000000000000
0.0000000000000000 0.0000000000000000 18.0009597799999987

C Pt

72 6

Direct

0.2201881182295335 0.2772033513345310 0.9938785345432551
0.0536243237602622 0.1107017457206925 0.9892145856542944
0.8874794380663226 0.9444810484571767 0.9916304962758964
0.0536070962931913 0.2773667513911897 0.9937254925549845
0.8872391984080789 0.1106335647465841 0.9911226069056083
0.7218397820531226 0.7781826416776122 0.0024051924903609
0.5563193135826436 0.6116499644437319 0.0208478478923728
0.7205901245102182 0.9440004361075641 0.9963679829171538
0.8870046008462207 0.2772483622477742 0.9928303466433240
0.7207015278208397 0.1107897797867459 0.9939282387841801
0.5553394814242560 0.7797108499805985 0.0105308218498125
0.3862596012892300 0.4417719095469492 0.0088581966789641
0.3852107098425671 0.6108111566971760 0.0244776589334482
0.5542764403116068 0.9441500063690208 -0.0008320522735278
0.7207158120822443 0.2773690176145976 0.9942104188204354
0.2185664065661527 0.4426187073648807 0.0066362610877168
0.3870756095366021 0.7775627114589969 0.0052535583011749
0.5540348661925438 0.1104347469863311 0.9953340474072910
0.5545548541815982 0.2768530292220870 0.9988863555616404
0.2203566193432698 0.6112337786072028 0.0029643900666326
0.3876842421758918 0.9443422107547654 0.9959026316873433
0.3877156821567700 0.1106051380289486 0.9934472564575763
0.0539586950003562 0.4439705609513267 0.9969735425500249
0.2210603280932349 0.7775883027051971 0.9954576916381521
0.2207714907790707 0.9441247254329838 0.9907101213046049
0.3862197778263217 0.2760664415364534 0.9965038547993832
0.0539323961601886 0.6106629930082351 0.9938745571426830
0.0543811978927898 0.7777550121118421 0.9909073359328937
0.2205211315850117 0.1109729169878887 0.9891924521758994
0.8875860808225088 0.4442906938114871 0.9934726056474221
0.8878943636136323 0.6108989519012642 0.9933018564575998
0.0542150724752759 0.9443167794572950 0.9889587808122901
0.7211889463476000 0.4443720542134094 0.9968345783092882
0.8876914700644581 0.7776818788817628 0.9934019154033575
0.7221674247472671 0.6114404159093142 0.0011962896669940
0.5556701216585296 0.4437644700720100 0.0071608782745957
0.1649985326888351 0.1664299962584726 0.9896694865261082
0.9983351663061403 -0.0001920300344443 0.9891576466038549
0.8318721654822400 0.8330741628228652 0.9951070772469737
0.9980296333846984 0.1660337987649465 0.9905646012820647
0.8320142770641965 -0.0002144721830707 0.9923946956096145
0.4980901364854721 0.4978621202081765 0.0146428275405421
0.6657277573592403 0.8341538703379990 0.0025951576694504
0.8315453551295646 0.1663979457437212 0.9923136563821789
0.6649523658163732 -0.0003565399813626 0.9964095107642063
0.5004798506070047 0.6692383159696036 0.0244348824046167
0.3306387338211449 0.3320439556153697 0.9975037100975594
0.3299539545501740 0.4973455809828440 0.0164936003756571
0.6651553921042217 0.1663087261301118 0.9945699187877568
0.4988577420266761 0.8337409320534692 0.0054250530495416
0.4983003067871039 -0.0003081546290827 0.9966238684842064
0.3306810696741262 0.6670806080420394 0.0103747952194102
0.1639953237534277 0.3324502778326631 0.9977093719507225
0.3317388266030877 0.8328771934714561 0.9984211810148329
0.1641029873798133 0.4993330779588294 0.0030289883181089

0.4982938583295843 0.1658067781268245 0.9958295930009557
0.1654344903529132 0.6665464735476446 0.9966695035765695
0.3321162960499571 -0.0000937014893697 0.9928159472627185
0.9982658169974720 0.3330423641222231 0.9944920412336818
0.3316973707588728 0.1665896736048877 0.9918990436058170
0.9983645810601034 0.5000449638853564 0.9944417325904098
0.1654771250517923 0.8334613569006063 0.9916937155722072
0.1650715979200885 -0.0001604388372837 0.9888250150383050
0.8315955572630599 0.3329738052503038 0.9933656469052378
0.9987991741418020 0.6665480612033794 0.9920669972541507
0.9987881248178889 0.8332305804944150 0.9904088990219135
0.8323855552973302 0.5000951403272408 0.9941489231757322
0.8324788432211436 0.6669160294758943 0.9953967049470636
0.6648861050841058 0.3328083920369745 0.9964725345954024
0.6674278470523654 0.5005238093402027 0.0013720834667175
0.4980442123386460 0.3316518235149464 0.0042686850574115
0.6673105849723213 0.6674774301321301 0.0085839610132744
0.4611285476045783 0.4916188223286866 0.2414424000250327
0.4356431163133124 0.6456342582861334 0.2514710676262046
0.3448067524716461 0.5391576396609541 0.1343101463834457
0.5428944117133979 0.6433489658267646 0.1356082419910671
0.4980730616764453 0.3476827260480543 0.2457515069154398
0.5011789644250120 0.4217665536932042 0.1204458410890914

PT6(no-D)-XXXVIII – ΔE = 0.87 eV

1.00000000000000
14.7772044699999991 0.0000000000000000 0.0000000000000000
-7.3886022400000000 12.7974344700000007 0.0000000000000000
0.0000000000000000 0.0000000000000000 18.0009597799999987
C Pt
72 6
Direct
0.8927848821422799 0.6150141236634710 0.9887325617022498
0.0589301885061175 0.6147842045976830 0.9944019976319325
0.2255817462415663 0.6156150724383025 0.0060498002578768
0.8927383201967984 0.4486313530004651 0.9894853542036941
0.0596445565985988 0.4484448294114500 0.9966268760828285
0.3928661034733381 0.6173009475872178 0.0241663565023629
0.5602641282625527 0.6142449647942456 0.0049121034486745
0.2240116981342481 0.4470591621752860 0.0096210855329915
0.8925434294799786 0.2815399086268116 0.9892509160151306
0.0594452284283804 0.2822395191977733 0.9921541824967406
0.3927059976256757 0.4473603618906026 0.0170369984104823
0.7259377977468304 0.6151016684479629 0.9910140476214835
0.5599727979407163 0.4487338112076671 0.9967595230814155
0.2265031622908751 0.2820564761223494 0.9968517461056123
0.8931007231980175 0.1153981100126202 0.9875906591262051
0.7259076282820516 0.4483277307366862 0.9886818180483559
0.3928898842159541 0.2813647388346823 0.0077562966081146
0.0589822981831816 0.1148512412879370 0.9880747023631287
0.8922215754293674 0.9486191607949763 0.9878352974732465
0.5597702509583726 0.2825458970296921 0.9929456738196691
0.2237655185560783 0.1138707018655438 0.9946135916016132
0.0589188965479224 0.9481062032592433 0.9886626214784116
0.7263379722504979 0.2818059310160024 0.9884687433981725
0.3931098466721181 0.1141148396440315 0.0000675384997635
0.2254684368459223 0.9477425621730333 0.9946365684621696
0.8920893977541198 0.7812912302826760 0.9883979594446615
0.5589875472421326 0.1149743113904620 0.9929810226546394
0.3925059778609779 0.9483184144184307 0.9970795916457647
0.0589288508442110 0.7817585762880839 0.9909907023471831
0.7258563308906005 0.1154082569343515 0.9894373789743582
0.5594766025476900 0.9485453145068234 0.9958940329261523
0.2258864956558710 0.7816238159844318 0.9976242858348030
0.7256774260741423 0.9483961665798120 0.9915404905622580
0.3919085386661365 0.7817248991404568 0.0035729644746496
0.5600540610636529 0.7832260463630689 0.0039319255207744
0.7261006974929742 0.7818041026025639 0.9935160211142602
0.0035886825973250 0.6702963481990736 0.9909028914339899
0.1704635573986195 0.6704369389166303 0.9991997149716383
0.3361383735585619 0.6716990676459247 0.0109277908593555
0.0037978451516594 0.5041235898766629 0.9932544808454224
0.1695108053074108 0.5031583404284490 0.0050298095273362
0.6706188025870503 0.6701116978372994 0.9955966812077435
0.3349718797959795 0.5018974807904968 0.0242912164872493
0.0041495837668819 0.3372380226529259 0.9925245980791715
0.1702355933104158 0.3375281555949741 0.9986148420218868
0.5055494127393558 0.5045694724399326 0.0055508449329977
0.8370888507382404 0.6705576145202414 0.9886297466496003
0.6706598982431800 0.5038582226261568 0.9912591284725778
0.0035442545317323 0.1704651142950908 0.9881858954658185
0.3354054405157711 0.3362412051695927 0.0042592769475007
0.1702917389844458 0.1700979451855815 0.9915078424182653
0.5053901415518567 0.3377530612458841 0.9978493498459073
0.8374370817096022 0.5038358189509040 0.9883770473131506
0.3354771995911108 0.1685089058632983 0.0039491830420673
0.6705377951615930 0.3374872754941336 0.9890969101821128
0.0035646482549936 0.0039341037009848 0.9874126460404682
0.5038428068558147 0.1700871370123380 0.9953686092973228
0.1693210201945305 0.0030270649918194 0.9918318601254370
0.8369411990317843 0.3370253097719313 0.9887335989278796
0.0034813634143376 0.8366970888592391 0.9887096528172563
0.6708317680448062 0.1705878447662457 0.9898438921628099
0.3368933476712996 0.0027967476742035 0.9975140402890972
0.1702908045991691 0.8372814120697640 0.9940323189071805
0.8372815492467112 0.1709509875089665 0.9882459989649632
0.5035986847852243 0.0039486802212849 0.9954528117689136
0.3365242937711344 0.8366459993452011 0.9988453613323784
0.6701669924297278 0.0037520958831365 0.9921130437782750
0.5040679197603311 0.8375844485470694 0.0016712729767789
0.8373795565960691 0.0038754467746998 0.9882336623644505
0.6707110690289255 0.8378455731354322 0.9959513370287141
0.8363438494907740 0.8366923133988422 0.9892768084822094
0.5054675526343857 0.6717669019422274 0.0156980400601157
0.4650040016194410 0.6624751148182355 0.1336885126329292
0.3149281243374347 0.2969582412272871 0.2098185389930478

0.3767092769124858 0.4655157599314421 0.1351583284070514
0.4295895556961398 0.4721024113017478 0.2666513537927315
0.3736878572330369 0.2156574042505568 0.1179696876367942
0.4536049444968597 0.6496686409460608 0.2711034292676331

PT6(no-D)-XXXIX – ΔE = 0.87 eV

1.00000000000000
14.7772044699999991 0.0000000000000000 0.0000000000000000
-7.3886022400000000 12.7974344700000007 0.0000000000000000
0.0000000000000000 0.0000000000000000 18.0009597799999987
C Pt
72 6
Direct
0.8846900873535333 0.6052291967964010 0.9937804953165070
0.0514714539283574 0.6050943316472370 0.9904246836505095
0.2176133597005716 0.6047734473343240 0.9924296138215780
0.8844117465904091 0.4381227276227122 0.9912014378971392
0.0512150030415270 0.4385825792011886 0.9905167679111742
0.3843714922320487 0.6056803283231460 0.0010561915155165
0.5508374851937248 0.6064763040963825 0.0194362454625576
0.2181072097662238 0.4383998430179048 0.9948134745654755
0.8846940975950304 0.2717640028810280 0.9906542592145797
0.0510075344618670 0.2715326280771819 0.9911916827514062
0.3824728371448884 0.4371495090334321 0.0055245568910820
0.7191176046895720 0.6044963287217868 0.0044659184085560
0.5520591222689148 0.4378690129894025 0.0135850628382670
0.2176664795218244 0.2719046201261364 0.9941713523799436
0.8844121539782748 0.1052206067892740 0.9921911707590851
0.7185375573415129 0.4387311640071658 0.9974296526989619
0.3840838367980126 0.2710735431857643 0.0007351353461331
0.0514403485350385 0.1052266626622682 0.9920035970230430
0.8844046463408546 0.9384292683763320 0.9955779961223854
0.5510774676255608 0.2710008522982363 0.9994012101654661
0.2173653897601397 0.1047766625392654 0.9932482060410095
0.0510510277278726 0.9387488248824596 0.9928254887212447
0.7176662884423948 0.2720802504480488 0.9932313852275527
0.3844636124887089 0.1051873991998420 0.9948365881927046
0.2180666001738345 0.9384804514450664 0.9918973775393312
0.8850476885001086 0.7721480114711099 0.9972357736643715
0.5515302487546734 0.1050771929053340 0.9932485041297667
0.3844155051954843 0.9381853411905183 0.9921090325203963
0.0509350199223562 0.7714529161885224 0.9917540278118153
0.7176046591885168 0.1048259761930055 0.9921100144072312
0.5512430606453833 0.9387918250661258 0.9931224275428647
0.2178219439422691 0.7719406320185627 0.9910810350272570
0.7179959446902515 0.9383089352715359 0.9957159674108311
0.3847570517158942 0.7718363708181286 0.9938007260919690
0.5506601280458696 0.7718654358856938 0.0000277316013779
0.7186446743618049 0.7733355915449636 0.0062329066501050
0.9960558148908802 0.6613357768461654 0.9914607673102722
0.1623848689697454 0.6608491274998016 0.9908406570172943
0.3293886113877420 0.6609274403225669 0.9952697166216637
0.9960849610748497 0.4943888911312939 0.9902787231229766
0.1625492449949064 0.4946303410841821 0.9922337922473901
0.6640509720869829 0.6618009353915042 0.0150880895508578
0.3284003291471720 0.4936537038419999 0.0007261127806260
0.9955836146413475 0.3275151120134225 0.9905377371351065
0.1625408683140463 0.3275600737912256 0.9930633626905845
0.4939211279523548 0.4924299886768821 0.0188686095883668
0.8296053314029379 0.6607750533166765 0.9979403020575504
0.6644266028216990 0.4950498130135301 0.0038453989654670
0.9958894725766001 0.1612394649034243 0.9915416699933672
0.3282262735314703 0.3274097278034276 0.9994023671813395
0.1620446199872845 0.1605022961947142 0.9926645107798109
0.4951342678970505 0.3262110078284053 0.0057362267555021
0.8291714510525736 0.4942042088839074 0.9935941970635369
0.3292973324088635 0.1610381477803600 0.9958120510813586
0.6626291958939916 0.3272855895580804 0.9967639301338309
0.9960903988640197 0.9942943123369048 0.9930867929912282
0.4957855719504849 0.1610169954780005 0.9956472779689989
0.1624451057416536 0.9945619609302980 0.9922716898635713
0.8292114323273907 0.3279786772688240 0.9913384375515690
0.9952693860354600 0.8272838748804077 0.9936065977626427
0.6620405837931429 0.1606245092744700 0.9927004997933082
0.3289067419461489 0.9941888883624870 0.9925636400034676
0.1626683578651296 0.8276669564200461 0.9911289010249007
0.8292935723756187 0.1609598798112586 0.9914920423745954
0.4959263943661085 0.9941468402974252 0.9924442283423431
0.3292617297377589 0.8278974401233583 0.9918588053320789
0.6623496706051313 0.9945915957012161 0.9932772260539124
0.4954640179637266 0.8273916800432488 0.9948961762127269
0.8290068089404983 0.9942288647197088 0.9946753089108284
0.6627892394481023 0.8283061839572596 0.0007411859349276
0.8296681252990439 0.8284527946046794 0.9996672090489582
0.4950165547648524 0.6621160392029140 0.0061614860444124
0.6793663922700119 0.7359138420894382 0.2518763228466980
0.5438634595289997 0.5377627252880686 0.2569970767122314
0.6149309857248255 0.6443645091588337 0.1301706348914138
0.5417278672877970 0.6629101201019125 0.3482316514139043
0.4717594264916585 0.3482714771356683 0.2538902075515068
0.5095163442141839 0.4358120099889717 0.1300026934909297

PT6(no-D)-XL – ΔE = 0.87 eV

1.00000000000000
14.7772044699999991 0.0000000000000000 0.0000000000000000
-7.3886022400000000 12.7974344700000007 0.0000000000000000
0.0000000000000000 0.0000000000000000 18.0009597799999987
C Pt
72 6
Direct
0.8916918132432770 0.6223290412790021 0.9872423432794761
0.0586598731112886 0.6222306833024760 0.9884779392368870
0.2251682464709361 0.6221913101219130 0.9898332714340654
0.8918956593711727 0.4554199014446567 0.9926126647828752
0.0583959522483468 0.4556024320835945 0.9928787789193123
0.3920140951713833 0.6227227279580418 0.9898141339468651

0.5587973725771107 0.6226603450420001 0.9883132934978462
0.2250863280413498 0.4556563447445612 0.9954831039368628
0.8920725449519011 0.2889442747562541 0.9948586080861830
0.0580719137291652 0.2887402706028936 0.9958581223447283
0.3923581838032533 0.4573429686857295 0.9983361481483328
0.7249210706710230 0.6220337082949925 0.9873514089587232
0.5592546962778187 0.4573049485627152 0.9978424843892668
0.2236167692426676 0.2885732297699448 0.0031938957469464
0.8918352065952391 0.1224724226785838 0.9930769798214101
0.7258060762312368 0.4556927738590346 0.9951298841232017
0.3905142951452021 0.2877424106428776 0.0119544445109838
0.0588386666142426 0.1222886524466844 0.9933981330593369
0.8918052890779578 0.9555909935181077 0.9881275023383793
0.5597884143846130 0.2885414663991455 0.0135151259104376
0.2249944473156020 0.1214714172081912 0.9953708647213091
0.0583445691571498 0.9556495398817333 0.9877783437713319
0.7257070616781292 0.2892607899770638 0.9994714567779397
0.3915539808303095 0.1217511626695469 0.9991499651912363
0.2253733024784665 0.9554938944571063 0.9895405959835699
0.8918483799439585 0.7889186742163403 0.9851860705720696
0.5583055306174742 0.1220599739165635 0.9999604798515023
0.3917555173605578 0.9553195058912820 0.9922816829275831
0.0583562243370892 0.7888234176245774 0.9860214457809633
0.7249238035841330 0.1223807562495907 0.9957302274946969
0.5584815987396610 0.9557992186225164 0.9920048982415324
0.2252099265113330 0.7890418100724332 0.9879350526191217
0.7254070029643600 0.9559173861300607 0.9899235340498933
0.3921451864079586 0.7890650948810389 0.9889200947505898
0.5585609494640522 0.7890686454573981 0.9877650957212509
0.7250681084500314 0.7890915756984853 0.9859637266729777
0.0030438149719600 0.6775325253922730 0.9867386297701231
0.1695972618946513 0.6772436476891386 0.9883322239303496
0.3365203686599827 0.6775606626351944 0.9891190128418046
0.0031924042513793 0.5107936299842422 0.9910413806022476
0.1699095316729853 0.5108690081458249 0.9924822820796564
0.6696208764550562 0.6775701784868886 0.9864638524224958
0.3366530935051131 0.5111342632349718 0.9941028311398412
0.0028968537382852 0.3442511789563483 0.9944273036879849
0.1693072197983767 0.3444065741756432 0.9985042990715627
0.5035017351635744 0.5116144574636223 0.9936616119150017
0.8365010999133986 0.6773851263743111 0.9859302231541918
0.6697793221020234 0.5113283574275371 0.9925932084731741
0.0030601773630536 0.1778082273043466 0.9943562380104041
0.3360970235533003 0.3456757426586066 0.0086627488402442
0.1689042366006461 0.1769333154006247 0.9974850320348523
0.5041019227217305 0.3459367225992480 0.0119750020135498
0.8360330793725907 0.5105465341108086 0.9910381543060112
0.3355090841455777 0.1758287947292203 0.0012468644195067
0.6714618108422101 0.3448789810896216 0.0029787675137527
0.0031411651326323 0.0106418668939980 0.9893665007393579
0.5028100823203872 0.1763323203366269 0.0049820367399960
0.1697967118052688 0.0109020329216065 0.9900717196014099
0.8368349618999247 0.3446081378834975 0.9953568610876644
0.0029759428560396 0.8440250247580607 0.9857571533914822
0.6698709032780030 0.1775084662905897 0.9993740221062453
0.3362365675483474 0.0105892362254423 0.9930833446673191
0.1699531168502091 0.8441891879934218 0.9873327708811175
0.8366557547899163 0.1777187681869421 0.9947963116306298
0.5029981463969690 0.0105739587314702 0.9946089633500605
0.3363717077390760 0.8443156421529494 0.9894026909744440
0.6697508675051935 0.0110708971338056 0.9927564107001174
0.5028631180833543 0.8441625503258194 0.9892477593996816
0.8363012005919117 0.0108330853812078 0.9903804125123941
0.6698042200360774 0.8442454673485713 0.9874855848269846
0.8364745811932508 0.8443167213416487 0.9859304069221793
0.5031150419868808 0.6779410618890864 0.9880743849272662
0.4536247328091392 0.3669055778621129 0.2467915543655721
0.2670772154737975 0.3093411645518316 0.2513930324782407
0.6247755006019418 0.3604867038981041 0.2449397575936345
0.5981606676951543 0.4710927219746424 0.3354572157532942
0.3324935205955128 0.3053651255994936 0.1246443650773799
0.5271677588888437 0.3284077789541868 0.1255508364453206

PT6(no-D)-XLI – ΔE = 0.87 eV

1.00000000000000
14.7772044699999991 0.0000000000000000 0.0000000000000000
-7.3886022400000000 12.7974344700000007 0.0000000000000000
0.0000000000000000 0.0000000000000000 18.0009597799999987
C Pt
72 6
Direct
0.8929390309865681 0.6126459174517223 0.9888420656525412
0.0594850820675674 0.6128917268865379 0.9934330425178572
0.2262828829560348 0.6140796619575752 0.0046242080618271
0.8929401302783617 0.4460833803844722 0.9908536271522337
0.0595450104359472 0.4465046836828463 0.9990880658264700
0.3937320854137951 0.6157663160125892 0.0226751887242216
0.5610816275484680 0.6118654630870139 0.0040780726815512
0.2246465697895275 0.4457825323961160 0.0150614976729457
0.8934061826428987 0.2794980846049100 0.9925747551982909
0.0590430250018699 0.2790151212285394 0.9995161643511636
0.3954615492887328 0.4459340939801706 0.0233831827121875
0.7263070918131334 0.6126599938402393 0.9914192037602234
0.5605885989097530 0.4459888755176209 0.9990325925063246
0.2251645134380738 0.2777682528200813 0.0068083088687558
0.8930639969292500 0.1128192267440440 0.9927680990762013
0.7262910284057398 0.4456751166302837 0.9901978545614583
0.3935990940226333 0.2781491185418830 0.0067524893008297
0.0593456566904038 0.1125320270256083 0.9950680208869329
0.8928854710509100 0.9462683031302817 0.9915280751942390
0.5598646174758457 0.2794217500575016 0.9948336332828163
0.2264531857307119 0.1125591421753143 0.9974499830553896
0.0597476495847786 0.9461828646485984 0.9911473276477949
0.7267113151007294 0.2793286119075020 0.9907619861907762
0.3933478319776640 0.1125760194689747 0.9955317251979636
0.2263080585057722 0.9461503284311306 0.9922883586524023

0.8927501864974374 0.7793348377668536 0.9892815864602298
0.5596850049802029 0.1126306117197586 0.9926012699814564
0.3930206210101801 0.9463550041304529 0.9931596282288808
0.0595027367440011 0.7795460324238732 0.9899799100053528
0.7263093356911554 0.1126514367150975 0.9921478346544319
0.5595588547626349 0.9459660406135626 0.9956068450737661
0.2262200927111522 0.7797492058200959 0.9939896738068583
0.7264271849672426 0.9462089650441712 0.9946887363903443
0.3922206851643821 0.7798720241673038 0.0004284829972363
0.5603703488790970 0.7808146571527601 0.0057537230676630
0.7265101562812362 0.7797045457877019 0.9944460538578994
0.0039714811621607 0.6683324904106343 0.9900493509818986
0.1707857110544992 0.6688208856991480 0.9968010008468156
0.3368802406618698 0.6700296067113456 0.0084389185693325
0.0041146740102960 0.5018975319003403 0.9940823375700347
0.1694525800549016 0.5024012557505628 0.0074522045633856
0.6709788435624446 0.6678208403785320 0.9955083716356654
0.3361590217062584 0.5018660564862500 0.0272268151531705
0.0040984905259336 0.3348060808981117 0.9965393163225755
0.1695068821313157 0.3332861560589677 0.0081191398317344
0.5059490541109071 0.5018680655073666 0.0076043212784498
0.8373781152640021 0.6683002335577086 0.9890224180811416
0.6706376666718228 0.5013430620243540 0.9923293000531785
0.0039550662828916 0.1680582210844861 0.9953589993489729
0.3369960639004645 0.3327429547480207 0.0159587460782120
0.1708981741071085 0.1682233486253253 0.9990818517155091
0.5050397353403966 0.3344926670985728 0.0004831559402163
0.8376100132020738 0.5016257853071480 0.9892549047991537
0.3373939397964989 0.1674979078168803 0.0000554233187913
0.6711500901497445 0.3350741236326868 0.9914351084816886
0.0041939992160779 0.0018454304327307 0.9920132512439466
0.5040815109325436 0.1680207298310350 0.9943151185357166
0.1707686837582614 0.0017416379597464 0.9930568400513096
0.8375775808804491 0.3350231365583696 0.9910392176193028
0.0041145334604025 0.8351346117185017 0.9894361285159903
0.6710646515062706 0.1682267940263529 0.9916743444734522
0.3374870773321987 0.0014552011172668 0.9931585485125183
0.1705672644393843 0.8351255428238034 0.9914731619775310
0.8376792344397828 0.1682509326383013 0.9922380587304787
0.5039405800779022 0.0016809682132717 0.9934928980823585
0.3370972236672642 0.8350760065725567 0.9948659500199639
0.6708450898725289 0.0016151636296158 0.9942419454442586
0.5042184618023882 0.8357855980542652 0.0008737462570352
0.8377613116991611 0.0016370053097248 0.9924400987566884
0.6716141540830733 0.8355098486159847 0.9983076904028394
0.8371139248867010 0.8348525921454453 0.9910053221431241
0.5057395959293487 0.6686815715186327 0.0128074449238653
0.3777770675421550 0.3809805454827995 0.1343456394199691
0.2478262359315693 0.4446582381065056 0.1325623817275101
0.4845613630202479 0.6857508755129729 0.1266227764710095
0.2656694909161246 0.3585048318138035 0.2542067245798449
0.4129992660983390 0.5389924787177023 0.2230992004885977
0.5418475029257550 0.7192125252177277 0.2588121451988386

PT6(no-D)-XLII – ΔE = 0.88 eV

1.00000000000000

14.7772044699999991 0.0000000000000000 0.0000000000000000

-7.3886022400000000 12.7974344700000007 0.0000000000000000

0.0000000000000000 0.0000000000000000 18.0009597799999987

C Pt

72 6

Direct

0.8842053480936301 0.6094762815588695 0.9994947061622810
0.0501137647237471 0.6089840254587386 0.9921455546125415
0.2161131036162374 0.6087222515712511 0.9929477123609090
0.8831612049307012 0.4424244015981742 0.9916679321251110
0.0497966696273835 0.4424545680264558 0.9906335914640323
0.3824716331989606 0.6094462903310358 0.0022958628447398
0.5477787691291596 0.6099844369070695 0.0243355770490581
0.2162956297440672 0.4421713520467350 0.9957568318992642
0.8831056784433713 0.2757917971898465 0.9901677758207228
0.0493861631770827 0.2754353852977616 0.9914633060556213
0.3809201427563451 0.4412432102697323 0.0090815396908255
0.7182314704938193 0.6085242849098194 0.0176506130384553
0.5500431107103481 0.4408663711897773 0.0158748825179202
0.2157340522308004 0.2754282207625991 0.9961752052935324
0.8826893803909783 0.1089930585196299 0.9921661604120189
0.7175383524727081 0.4424111170985299 0.9992947187661514
0.3808346927350073 0.2741403870645129 0.0023975962576728
0.0493783740906082 0.1087622184723713 0.9927102446934271
0.8827320478698866 0.9421228170965108 0.9966895340582269
0.5500576646104065 0.2745736451508662 0.0048952984426336
0.2157745319976172 0.1086924959492848 0.9946820604809830
0.0497669944745169 0.9426627519279265 0.9949329687255286
0.7164339588903275 0.2758776145266353 0.9930904384898724
0.3832251135735376 0.1089215248576565 0.9966788514492322
0.2165121775383057 0.9423497570381443 0.9936962964270677
0.8840826341916923 0.7762854270636694 0.0037448066591281
0.5498128031408882 0.1085155253076877 0.9957505945111649
0.3829433798191531 0.9421184288032478 0.9941730360640264
0.0498784125380993 0.7757528611039390 0.9955184777676465
0.7160174596921536 0.1087719235921654 0.9929836219701755
0.5497926484575757 0.9423617963424036 0.9957313999398067
0.2165523209151132 0.7758123877476422 0.9927235881288397
0.7164004034980707 0.9421375700022381 0.9977741461574041
0.3831885926375520 0.7756654104076830 0.9957372205919484
0.5493325882267399 0.7758916355938155 0.0044059114467956
0.7174814228438606 0.7774513890138479 0.0100068378771283
0.9945609458168647 0.6647563157409806 0.9952870999401853
0.1608771787097751 0.6643586577880569 0.9920174256318788
0.3275633303900847 0.6643808593224847 0.9961081228670139
0.9945173489560659 0.4980573353347992 0.9910292738755047
0.1606733961468265 0.4979741455601641 0.9925547722684200
0.6629634954968751 0.6668259080174721 0.0245107749359477
0.3263633891543236 0.4973694203985204 0.0026562660278273
0.9939503504251661 0.3311053729568343 0.9903439209793845

0.1604794871765733 0.3309539712562639 0.9942499214076150
0.4918730799489310 0.4965185806751151 0.0213939159528849
0.8296257657738053 0.6651282231362146 0.0079216809173204
0.6619083190010571 0.4965387831195898 0.0104028668859115
0.9938025521578169 0.1643928673817427 0.9916974690930545
0.3260660847369650 0.3303122070870472 0.0020489186842525
0.1602668690931779 0.1639942910819201 0.9940034520286076
0.4925653450458611 0.3291217238624995 0.0084717509424834
0.8279870985958055 0.4980240930562871 0.9958877606724101
0.3269940130728841 0.1642153101596335 0.9974253152475896
0.6618898172611978 0.3308256545374135 0.9977971065453133
0.9942258484022091 0.9977295114008697 0.9942708330594670
0.4936129358416537 0.1633792177944073 0.9990779557546645
0.1605474273240759 0.9976945112494917 0.9939232968324490
0.8276449232478527 0.3314603813018167 0.9908281245698234
0.9942100331518660 0.8310293076875439 0.9977130799385421
0.6603834267956472 0.1638750709583974 0.9938406245008480
0.3273764033786932 0.9976448849336919 0.9942089679182118
0.1611027878793081 0.8312067836181143 0.9934325874399974
0.8273733221615487 0.1643595551034167 0.9914110620507586
0.4940525063325225 0.9973869749964024 0.9947629107158633
0.3274113875350082 0.8310617350857044 0.9936350508317915
0.6604278056329207 0.9976299277454928 0.9953149644575277
0.4938618229640070 0.8308537719553613 0.9981065600775949
0.8270817369955239 0.9975332239850090 0.9953304992695138
0.6610695279877632 0.8314810614857748 0.0044006501037046
0.8278599171771006 0.8319389693699293 0.0030202088864257
0.4929153415654355 0.6654496557216092 0.0098911779315074
0.5880692545679480 0.4757382815860538 0.2424434674454474
0.7037546010629256 0.6375372988339194 0.1350406076162187
0.5470290035374461 0.3638064581338014 0.1221258947168877
0.5511829050201769 0.2949994329297283 0.2480341894074485
0.5055168660584641 0.5437419473170522 0.1358736483161564
0.6122669757202104 0.6549294995283503 0.2512286334092764

PT6(no-D)-XLIII – ΔE = 0.88 eV

1.00000000000000

14.7772044699999991 0.0000000000000000 0.0000000000000000
-7.3886022400000000 12.7974344700000007 0.0000000000000000
0.0000000000000000 0.0000000000000000 18.0009597799999987

C Pt

72 6

Direct

0.8884546085912675 0.6122932107783328 0.9945676550954943
0.0544699659522365 0.6120371905162756 0.9940080011593935
0.2208917990210395 0.6125989831190566 0.0022699538687334
0.8881195109117002 0.4456854535644936 0.9944204812194926
0.0544897558675217 0.4453433976085961 0.9970721147804298
0.3856900916465378 0.6123079897814847 0.0241361168049323
0.5568490107857258 0.6132065972574665 0.0220266767236481
0.2190989978433535 0.4440182253448341 0.0068364484567667
0.8875602126775277 0.2786424493114126 0.9934595256699836
0.0541540983166016 0.2787428002074819 0.9942287108188168
0.3868057712066673 0.4431604749838911 0.0101256918982742
0.7227155616798768 0.6128299987190360 0.0029826867456677
0.5561676923262979 0.4451028607805583 0.0094720302415467
0.2207989458056119 0.2786059176428139 0.9948743358371885
0.8878201364757317 0.1120245116267924 0.9916579806990384
0.7217369159307125 0.4457700722138398 0.9987588094572430
0.3868598895689033 0.2774782773284770 0.9979871799665716
0.0542221507126541 0.1120869656460215 0.9899312864080017
0.8880502869797766 0.9458532653487879 0.9922907820713078
0.5550692283498933 0.2782847577035881 0.0003761674104723
0.2211232126337634 0.1123733659192538 0.9900795574638934
0.0548014100410370 0.9457028902211633 0.9895982911751986
0.7212851790010575 0.2788059584460569 0.9953783551035755
0.3883108004025431 0.1120245746084620 0.9942179461454614
0.2213638388945185 0.9455206174424902 0.9909388330306896
0.8882649285269437 0.7790547520158242 0.9944324421594217
0.5546127309551281 0.1118693054982955 0.9958459153043577
0.3882723648872215 0.9457475364188639 0.9956118191039565
0.0549296528675995 0.7791285458733270 0.9913542626350420
0.7212695186642506 0.1122049486112147 0.9943402463476048
0.5548577268900772 0.9455611269743471 0.9988510448428869
0.2216464835397076 0.7789822855104660 0.9949543808353667
0.7211708856597099 0.9453887327395947 0.9966016621827620
0.3876536038815317 0.7789422567338349 0.0043859792345700
0.5558521673963526 0.7811104960513546 0.0104136960088468
0.7224037184324601 0.7795812234085417 0.0032071596578831
0.9993275861427620 0.6679255845447329 0.9926989273441293
0.1659911294071037 0.6679142573746475 0.9961633725248760
0.3312477417133977 0.6684684832209058 0.0095408049545327
0.9989120348177565 0.5014288878136668 0.9947859227271252
0.1646469182152686 0.5007215622340624 0.0027856771999097
0.6678665909575372 0.6689188745208838 0.0098757246347105
0.3304365822930890 0.4987812785354393 0.0168806448690830
0.9988131893433660 0.3344102318490556 0.9948923793950755
0.1645533342428891 0.3338109534354672 0.9983064137271711
0.4986152316046883 0.4993409716817112 0.0163792840384005
0.8330295592159942 0.6682864268486597 0.9968374279222587
0.6679392815109324 0.5018880733808473 0.0035891596456210
0.9985987449083922 0.1674085986987066 0.9911884804735394
0.3312358952166790 0.3334227876652491 0.9988761498567840
0.1656026132564534 0.1678230682156254 0.9905813705678668
0.4985953200499935 0.3330308750473989 0.0058528580394395
0.8329333809790924 0.5014807167494340 0.9955885735373116
0.3323113892055716 0.1680028288881417 0.9930209262809555
0.6654662495247479 0.3342277103092499 0.9981327291674731
0.9989274811314743 0.0011903732134328 0.9898415193765970
0.4988625567685432 0.1672372895874688 0.9967786188132450
0.1656554284077529 0.0012368637774784 0.9894008677753092
0.8321443481497113 0.3343742734330064 0.9943178574247042
0.9993534870176433 0.8345993785349819 0.9911269509564207
0.6657258475592087 0.1677191982168296 0.9952665214504393
0.3327020032140524 0.0013015170831636 0.9930182503846083
0.1660521661603767 0.8348423068893425 0.9916909473879159

0.8321299642404227 0.1677960928261726 0.9928620932656784
0.4988998919731813 0.0011129278911497 0.9964915287659437
0.3323213552576973 0.8342691945315011 0.9977180526972731
0.6655279697827439 0.0010422521121072 0.9964608305624338
0.4993962904077733 0.8351124087209953 0.0048344086023491
0.8325947097230397 0.0011631132786576 0.9929085236821891
0.6663027397085770 0.8355572684311525 0.0028820551248216
0.8324448260878654 0.8344507589216477 0.9959559288531139
0.5009023981883942 0.6707289018686566 0.0245905899245003
0.3441737930944342 0.5415689833519366 0.1343956779157338
0.4951666243544324 0.3517698854235647 0.2487884191051322
0.5007908834316837 0.4252194879368929 0.1227860255626894
0.4339438526187749 0.6504965346273011 0.2509680211446579
0.4584333562067187 0.4957056244533717 0.2428305030874540
0.5422851908630832 0.6464257654515819 0.1364207698675060

PT6(no-D)-XLIV – ΔE = 0.88 eV
1.00000000000000
14.7772044699999991 0.0000000000000000 0.0000000000000000
-7.3886022400000000 12.7974344700000007 0.0000000000000000
0.0000000000000000 0.0000000000000000 18.0009597799999987
C Pt
72 6
Direct
0.8910333310086216 0.6177101373516933 0.9938211438461622
0.0577110004590935 0.6177632491638505 0.9922312773332678
0.2243937636295570 0.6178761726378497 0.9953463901250572
0.8909460070402275 0.4507070712942252 0.9934429642873610
0.0569172812639209 0.4506514866074127 0.9934319534742784
0.3912130773003568 0.6190658778134051 0.0033448608298130
0.5586307133058028 0.6186768358031145 0.9976399515961418
0.2219635005482772 0.4502540719903507 0.0011837078866535
0.8905081801560897 0.2841989238558398 0.9912738243424215
0.0568164883398623 0.2838494610840883 0.9948552441805418
0.3911721712383454 0.4526932329535072 0.0142882052313453
0.7252941728594422 0.6182706001848928 0.9976889341893909
0.5593569151456919 0.4532439178085212 0.0161188340461109
0.2219644512741468 0.2831751082203837 0.0075448047094753
0.8904540583443392 0.1175069997413161 0.9898191404124361
0.7252121974204471 0.4513905656514083 0.0020223205903349
0.3888214984694116 0.2812606358784109 0.0223477136337422
0.0573544073081962 0.1176465533583695 0.9940931075470871
0.8908426165756893 0.9508816398258588 0.9887167717386944
0.5585721109294965 0.2835766269989293 0.0081106330242804
0.2235023465377424 0.1165186168573769 0.0003880949229966
0.0571777646795866 0.9509216638388693 0.9910570344258858
0.7238806261122548 0.2842147664623127 0.9950307508959639
0.3904126682266025 0.1169868360330497 0.0023898489232863
0.2243214565794531 0.9510775505906039 0.9938439370178216
0.8911979890701929 0.7846783306562841 0.9902501330555324
0.5575322135405827 0.1178617731042166 0.9958767549928780
0.3909477880949055 0.9510082772328019 0.9938677916651386
0.0576893760508099 0.7843104590214836 0.9909454797948456
0.7237860150836184 0.1172733759349640 0.9904296193232014
0.5571205508137638 0.9508182174786555 0.9910942983321149
0.2241250717893095 0.7844099772942812 0.9925280176550686
0.7241590504209441 0.9508660922479848 0.9882384406003126
0.3909808302210109 0.7848675598646579 0.9940766659718463
0.5575279009961989 0.7840995846031902 0.9912449064961422
0.7242086481849910 0.7842692124168025 0.9897547831078768
0.0022742675906429 0.6734784108402181 0.9917914198807196
0.1685691142109320 0.6733116954315506 0.9927492963951821
0.3347822709242934 0.6737944232940194 0.9975863710755206
0.0017321190304429 0.5064453727422560 0.9925492453507019
0.1681469110927978 0.5063517469804140 0.9962172172176054
0.6690571859660395 0.6734987849276237 0.9941002201266613
0.3337198462816531 0.5070256786692440 0.0071940634368630
0.0013803064915550 0.3398350641780112 0.9927096233417316
0.1671376588534983 0.3392956289179523 0.0007472853707000
0.5029950485420969 0.5089037294972627 0.0089010068764352
0.8356702662628663 0.6733467405603903 0.9934643070463167
0.6707271948329705 0.5080549522981954 0.0061817496037548
0.0015556992456425 0.1730640219067965 0.9927324797598231
0.3329601418183756 0.3388431375431011 0.0196289694706238
0.1673986565527841 0.1724880678715692 0.0009598727173241
0.5040120387169011 0.3396213719064107 0.0226865855229050
0.8353550623554113 0.5067714825011009 0.9960754743042344
0.3339555409812505 0.1709630760173297 0.0078887394668214
0.6689788335705487 0.3394943298114583 0.0012738645246984
0.0017243645186085 0.0062208206150842 0.9907198667866552
0.5021644926349182 0.1731508981905279 0.0024344924507460
0.1686446172554668 0.0067059286298559 0.9941987636312319
0.8353953528917728 0.3401011806916117 0.9927683495213415
0.0019541771040039 0.8400552047364158 0.9901412923862054
0.6682090920357169 0.1731455350441280 0.9935772620318275
0.3349759322186046 0.0065567470613956 0.9961744952415827
0.1685645520478047 0.8399055216236633 0.9918514534907956
0.8348948224659907 0.1729775298417024 0.9901151471608571
0.5015717974332716 0.0063792691730384 0.9934182501074318
0.3352403981062722 0.8402524872300745 0.9930403995067820
0.6684619083795766 0.0065887167029004 0.9895489984681092
0.5015128395834338 0.8400710365964343 0.9920149178287829
0.8349890570496896 0.0063873822257037 0.9884766759207579
0.6687133150289171 0.8397232917724935 0.9888925956657175
0.8355751223208543 0.8399956486772808 0.9890886313379639
0.5029893542571884 0.6746098719443836 0.9960899892081372
0.4284714040154043 0.4542030727684079 0.2409593311375176
0.3461740165710765 0.3046928962720230 0.1339299693809579
0.4523138669993259 0.2990125631373530 0.2492942867079648
0.5443260661631868 0.4083788866145568 0.1334081730648151
0.3887157043927232 0.5264786915279274 0.1207853355380877
0.3911049366198043 0.5977758304954079 0.2469354336054224

PT6(no-D)-XLV – ΔE = 0.88 eV
1.00000000000000
14.7772044699999991 0.0000000000000000 0.0000000000000000

-7.3886022400000000 12.7974344700000007 0.0000000000000000
0.0000000000000000 0.0000000000000000 18.0009597799999987
C Pt
72 6
Direct
0.8937171143074778 0.6185610717667913 0.9882658134184936
0.0608599574110684 0.6187084701598380 0.9897075517522467
0.2272871489906194 0.6183927153175617 0.9929964303888639
0.8939342614513208 0.4513701385529032 0.9901445069986110
0.0601710766587118 0.4516823086267507 0.9921680506439046
0.3937329695572060 0.6190779435984055 0.9966898073402533
0.5613259374194968 0.6189331241959763 0.9939028228229176
0.2270052872068291 0.4522929494715839 0.0000357121423278
0.8943554382057144 0.2853108376774927 0.9908208915775205
0.0606683230255740 0.2848989117068328 0.9946176120406420
0.3949235311574384 0.4536068136638960 0.0127188523098383
0.7274878834231444 0.6185090423590296 0.9894873550652576
0.5618320072515672 0.4537180800441121 0.0001291514571164
0.2254150852825418 0.2845050842468595 0.0066937931680116
0.8939045506390002 0.1185784756800103 0.9906919665828937
0.7278251951065329 0.4520056575448663 0.9926641691949598
0.3942506499324097 0.2834132213000018 0.0186318965583894
0.0609830870186983 0.1188337085791105 0.9952611623884735
0.8941576529521740 0.9517555573342307 0.9891353674316079
0.5616657946787029 0.2844702610717604 0.0024647102756461
0.2273065011624169 0.1177977439020097 0.0011156466846671
0.0604343194049761 0.9519203938177156 0.9915603566145137
0.7272663061087385 0.2850265577387887 0.9925522956681334
0.3934060451316697 0.1176193605763487 0.9984576652249828
0.2275046202087054 0.9519907182393439 0.9925438797464778
0.8943218894066760 0.7855172607594767 0.9875739407217452
0.5608696982857424 0.1186952803455412 0.9933504682866356
0.3941222515594873 0.9514697049188143 0.9911875361319176
0.0606811590252718 0.7849952133265319 0.9889585548934363
0.7273452983639785 0.1182250413399544 0.9903376884851340
0.5604023295120939 0.9517459305575358 0.9899545528765614
0.2271383371738978 0.7852688228970521 0.9902257452162146
0.7275958239036768 0.9520367794482070 0.9887167595017630
0.3943295444964932 0.7855497490607064 0.9909719011393250
0.5605291762672024 0.7845922783164241 0.9904074026100389
0.7272458732482150 0.7853651254776963 0.9880229867795904
0.0054843820361938 0.6741135558435190 0.9884781358774077
0.1716753097218557 0.6739022918162973 0.9906416919272374
0.3384615220904266 0.6738194295688515 0.9933394865173852
0.0052202789445701 0.5069497772289466 0.9903114081141737
0.1721604071146743 0.5074567459337089 0.9946332319275655
0.6719366888953573 0.6740501329509883 0.9897542842438654
0.3379599643258047 0.5082809643645092 0.0039325152379988
0.0049462817499517 0.3406917327300576 0.9921663402757659
0.1711304950492902 0.3399971863652880 0.0001036773737937
0.5071207609247921 0.5084195831342484 0.0007707746471581
0.8388145681060450 0.6737808485035188 0.9877279146732150
0.6723198856417198 0.5078977745282387 0.9931394151614228
0.0054534681296587 0.1741127739224240 0.9934193778700973
0.3370151976649893 0.3406767095641428 0.0194785937337798
0.1712678743440250 0.1738663092843709 0.0015572077267052
0.5070308353039650 0.3414207426788138 0.0112063864101657
0.8380409958837944 0.5070645368565607 0.9898617955367115
0.3379673979526245 0.1719132585288321 0.0055561722916906
0.6725460701999140 0.3403211982330774 0.9955355723396266
0.0053878435803156 0.0070160806080537 0.9917327555314586
0.5056716025531074 0.1734781689318297 0.9983716948581858
0.1722509560100249 0.0075255564820154 0.9947146804699400
0.8390186533192860 0.3408786244681039 0.9907704428443083
0.0052200075168898 0.8406921145346047 0.9888712162543314
0.6718411431102567 0.1738534924728583 0.9921554164381945
0.3383097093561531 0.0071980267023832 0.9933131024298021
0.1720156713822760 0.8404379573682377 0.9901622147021669
0.8387382977051629 0.1736956633448585 0.9904460203520671
0.5051180552653989 0.0067012241298912 0.9912114010384272
0.3387634930375825 0.8408806809184100 0.9904755316124110
0.6721787404288975 0.0074069527154279 0.9895560695048289
0.5048324186828594 0.8404796006292514 0.9902974453372266
0.8384802041754114 0.0072373833084356 0.9893316156436001
0.6720368278710538 0.8402678031773121 0.9886563206071131
0.8389906959587989 0.8408647722116172 0.9876930757049251
0.5054579413373546 0.6744309479577879 0.9936269603692978
0.5357018793407633 0.4755425435774825 0.2434633788856786
0.3686587220364075 0.4838460580084032 0.2586282068188623
0.5404797934858436 0.3061581206915847 0.2555570198573136
0.3648363076193206 0.4122631739093805 0.1283729519413583
0.3630005481989329 0.3046517549335235 0.2523050873402113
0.4678395018127972 0.3083466956834684 0.1267192839957900

PT6(no-D)-XLVI – ΔE = 0.89 eV
1.00000000000000
14.7772044699999991 0.0000000000000000 0.0000000000000000
-7.3886022400000000 12.7974344700000007 0.0000000000000000
0.0000000000000000 0.0000000000000000 18.0009597799999987
C Pt
72 6
Direct
0.8923200365550343 0.6110060741616863 0.9873890285433049
0.0586211192608772 0.6108078571847315 0.9897876241829664
0.2252760612423685 0.6116606104879025 0.9976934784302500
0.8921688087443727 0.4444092536379571 0.9875907331741374
0.0589973639783778 0.4443090940870320 0.9916356496353984
0.3931419519523160 0.6131293332541716 0.0121687058188158
0.5593888098022006 0.6113223160330108 0.9969469428913271
0.2237520304396696 0.4435258599297001 0.0027105115091501
0.8919889574868165 0.2774436015984563 0.9885407272162183
0.0585065288848128 0.2776735854172472 0.9931111608857961
0.3937471773290753 0.4446072009987319 0.0129716238521524
0.7256634057712645 0.6113574304044533 0.9889376600559316
0.5596113291221556 0.4447795084514397 0.9952073942463713
0.2239829745080542 0.2765372497317031 0.0027716850184802

0.8921607985163291 0.1110428664463257 0.9892418924085220
0.7253625433722348 0.4441096884470781 0.9882573401888664
0.3925835096184898 0.2765258269259405 0.0033987122828592
0.0583819807462191 0.1108123183621430 0.9929236052989907
0.8921428285978834 0.9446424903966815 0.9890194909883903
0.5587295137503148 0.2779048178530985 0.9926713213856644
0.2255742572363317 0.1110186347098363 0.9971722039260342
0.0590319893840245 0.9445559955247944 0.9911301618141124
0.7255302296997144 0.2776431048658807 0.9884125953646361
0.3925212764543105 0.1108563337121424 0.9952027865340440
0.2255329302288871 0.9443553816735530 0.9932348054779041
0.8920685985122176 0.7776867026237966 0.9881396392489918
0.5585830606572415 0.1107397897318947 0.9913845189040771
0.3922212988613865 0.9446646141511152 0.9935019469249653
0.0588989618831874 0.7780028738839775 0.9897030387461783
0.7252024472861223 0.1109306986038305 0.9889452237671676
0.5587493574468425 0.9439557270099854 0.9925437026447028
0.2257550211371111 0.7780306367501524 0.9932961797822912
0.7253122939748522 0.9441437403961288 0.9896284251283021
0.3919735795524346 0.7780995776320694 0.9978488442766320
0.5590956224176651 0.7777463887934317 0.9958613284283615
0.7258378867731494 0.7778957681025034 0.9899402261204528
0.0033932197533986 0.6667587460754660 0.9885153092650256
0.1703820276788264 0.6667620027258891 0.9930532381508499
0.3362658038408810 0.6681864806006814 0.0029556900454324
0.0034938725029932 0.5003820852095586 0.9891344690888104
0.1694846720491867 0.4996196235294903 0.9970253725100449
0.6704682701150375 0.6669430888419470 0.9910759140818755
0.3355492276750311 0.5000542025399994 0.0142212661705230
0.0034057342154057 0.3333186114716682 0.9906673905323160
0.1693503527590252 0.3322219743891756 0.9989733727208687
0.5049914515443135 0.5008914294796085 0.0009305738082261
0.8367799195717325 0.6670755085150191 0.9876269179882584
0.6699651484099860 0.5000854961968955 0.9899132619415099
0.0030527004593068 0.1665010387196233 0.9915268125955023
0.3358481770842658 0.3316584796986319 0.0113633588112236
0.1699514300172282 0.1668949891021327 0.9975655700092148
0.5039894424786979 0.3330927442233858 0.9970880464752554
0.8369519586579202 0.5002272524509834 0.9872156725709686
0.3367833655371868 0.1663750767650995 0.9988566107642569
0.6702346006925168 0.3337303735803161 0.9892967717461758
0.0034885588468683 0.0003861213198277 0.9906873990155560
0.5031845753983859 0.1664011581799230 0.9932212544951327
0.1701517288561405 0.0004162071236919 0.9936457221486847
0.8366328808020995 0.3333955826062009 0.9878021179468774
0.0038160253720463 0.8337236592242618 0.9892576088893605
0.6701482222176836 0.1666721635482205 0.9893978740315532
0.3368315604953196 0.0000241323341115 0.9938688957857664
0.1701799956546424 0.8337850461174199 0.9918022374080380
0.8367226486808121 0.1668580210411363 0.9885659062848546
0.5032523195605592 0.0001775107591584 0.9925411386755556
0.3363878984928661 0.8336012149281657 0.9947005929519790
0.6698810233385970 0.9998482912359847 0.9902048087832540
0.5034998288607468 0.8336362255600065 0.9958460255344690
0.8369190435641514 0.0000457408318297 0.9888873207317914
0.6704289882957042 0.8337013179207347 0.9912689931639846
0.8367705722536911 0.8333948945406817 0.9885819163041845
0.5049672601364890 0.6679305751910007 0.0015049065904265
0.5217400004955763 0.6352348946053752 0.3212994219500800
0.3897418648423141 0.4770401623605380 0.2537177694619857
0.3919947857798931 0.6530871276753487 0.2468196127313860
0.3469022301191700 0.2945293400121187 0.2503608002825928
0.3661818097353091 0.5584897850465893 0.1264656797019299
0.3770732756561159 0.3827979415028722 0.1265282265564878

PT6(no-D)-XLVII – ΔE = 0.89 eV

1.00000000000000

14.7772044699999991 0.0000000000000000 0.0000000000000000
-7.3886022400000000 12.7974344700000007 0.0000000000000000
0.0000000000000000 0.0000000000000000 18.0009597799999987

C Pt

72 6

Direct

0.8860917570293481 0.6054682898641772 0.0007056771438485
0.0519051028809443 0.6048441342273847 0.9927308851863899
0.2181489160347851 0.6051007138418143 0.9932838178417640
0.8851873064425106 0.4381898531551158 0.9958472539051257
0.0518748132379443 0.4384808427385849 0.9928976261097517
0.3844264839372897 0.6056363691795212 0.0015184570062274
0.5507269136415900 0.6060443312369870 0.0200389320660861
0.2183542245835710 0.4385771395020939 0.9968829209561463
0.8853738743977786 0.2714591421349297 0.9922213634334227
0.0518351457395738 0.2716170404852036 0.9933956492416485
0.3832457919320262 0.4378950280899758 0.0078087691902056
0.7210462015735004 0.6053412100021163 0.0222747705735031
0.5507225048385962 0.4360654378155786 0.0087165120582853
0.2175567250079595 0.2711276429804954 0.9982003793316849
0.8852459615762314 0.1050232304978067 0.9925450426053715
0.7192771237945195 0.4369530329760707 0.0057327429478349
0.3849573427558823 0.2703676138955302 0.9964994322049776
0.0515790257554798 0.1046838091272519 0.9935624196396873
0.8850275440266913 0.9383935024454999 0.9964692915769362
0.5521648202658227 0.2714805549967210 0.9941426399302955
0.2180635524312464 0.1048845954860482 0.9924915522690156
0.0518098991624356 0.9385614573140799 0.9935208726788574
0.7189148075465397 0.2715897921461590 0.9935847714855583
0.3856199971879875 0.1052460351244733 0.9894001070948235
0.2185130830622413 0.9383842406520984 0.9898925426001526
0.8856312871607699 0.7718081915073398 0.0021446815107069
0.5522063577566882 0.1049721207999994 0.9897194313901920
0.3852478591726367 0.9385834854483122 0.9892552014528420
0.0516940775573431 0.7718084684105193 0.9933885419375841
0.7185122396702326 0.1048858839420248 0.9908361086407709
0.5521080804257323 0.9387286536943478 0.9916859836115677
0.2185497546418418 0.7719700401015501 0.9905005642811133
0.7185275402030058 0.9382420537500522 0.9949495973306597

0.3850906817068704 0.7718838934449508 0.9936061966333227
0.5515140457705456 0.7724422957443835 0.0017700773705158
0.7196193326126235 0.7739677818067037 0.0081335951817749
0.9962818259568422 0.6607310653992897 0.9946894462632443
0.1629371565673381 0.6607574520687436 0.9915890279569979
0.3295600928980917 0.6610899634493208 0.9956438291485625
0.9968351616786038 0.4942412128457789 0.9934413050584894
0.1628536948922061 0.4942777243140526 0.9939703299418525
0.6641952171155481 0.6636468605583445 0.0224297297997538
0.3282710304009058 0.4946799380445981 0.0019167615435123
0.9965303534335064 0.3271783680283988 0.9927218182159478
0.1630772669966234 0.3270582598859164 0.9959864092365720
0.4950464439413409 0.4921844849912489 0.0147578750169401
0.8315993276301086 0.6613891558626435 0.0076139083821971
0.6627284183359734 0.4917588143861806 0.0155973806397043
0.9963038841687748 0.1605203950602245 0.9931801468466830
0.3287217676915475 0.3258720781087874 0.0039157353212573
0.1627117134311717 0.1600836345623620 0.9946116357757688
0.4965588941490466 0.3263430181402640 0.9976830497627347
0.8304396660250433 0.4936067356550282 0.0015123228343228
0.3300445147793738 0.1608756894554730 0.9917383258784227
0.6636090785320263 0.3267002311280010 0.9975429097470467
0.9965363287133471 0.9939310120562936 0.9941460166965470
0.4965795891474372 0.1607004574846869 0.9901637946624433
0.1629372607758270 0.9941424804054151 0.9913915571188028
0.8299100528450083 0.3272340929175570 0.9938991174295921
0.9962958661016401 0.8271025594969217 0.9956813319870861
0.6631433764673673 0.1602830764412104 0.9907567686320036
0.3299471739753201 0.9941244444712183 0.9888108211753277
0.1631521654718355 0.8276933879396537 0.9905930072176972
0.8299706975134811 0.1606289782834338 0.9915956094811236
0.4965889055646002 0.9940895633730236 0.9896021622805407
0.3295838848193782 0.8274594612285924 0.9905369524423335
0.6628655706485276 0.9940285932789195 0.9919312591849447
0.4963131508673797 0.8273178294253540 0.9950541047320272
0.8298345668533003 0.9938729908699244 0.9944479220357536
0.6635840462942681 0.8284266085921388 0.0010417718961762
0.8300388024050207 0.8279444146100943 0.0024383311122165
0.4953756669293128 0.6617434473505810 0.0082373737172432
0.4188751360519802 0.4190238934086778 0.1214219794105418
0.6934529090971751 0.5364476637010327 0.1329203104621328
0.5326839646475392 0.4896899383899651 0.2410946660059565
0.7113475909540696 0.6454237477496463 0.2490623079594059
0.5984210086111972 0.6396895651019676 0.1342456413380262
0.3517559469220828 0.3467499515510326 0.2478883884100256

PT6(no-D)-XLVIII – ΔE = 0.89 eV

1.00000000000000
14.7772044699999991 0.0000000000000000 0.0000000000000000
-7.3886022400000000 12.7974344700000007 0.0000000000000000
0.0000000000000000 0.0000000000000000 18.0009597799999987
C Pt
72 6
Direct
0.8834726237844279 0.6088133076050894 0.0024631196285156
0.0496510283450533 0.6084823824025278 0.9990587107388009
0.2162914480819680 0.6083164379593029 0.9982068447310795
0.8838784895066212 0.4414966942825274 0.0046036484394349
0.0495370146793235 0.4413606590563219 0.9998733813833596
0.3823147087101759 0.6078381420733621 0.0004547117920524
0.5502005493252184 0.6103795877117761 0.0025924876395251
0.2158785594561294 0.4417168402016145 0.0018381002190822
0.8828350265024483 0.2752051138916087 0.9980212671331898
0.0500595663933581 0.2752115912371949 0.9969201484931602
0.3817944386142784 0.4410571752977717 0.0113548057060413
0.7169104542644575 0.6096386959229889 0.0040678642823693
0.5479440037594330 0.4403578116405598 0.0182507298353869
0.2163938173317277 0.2746208319398846 0.9973278438804840
0.8829800514673138 0.1083968287346622 0.9923118299777656
0.7165220830253745 0.4401310912994845 0.0136863048383020
0.3825610675521816 0.2742087173228427 0.9977757713153039
0.0495264861700306 0.1083505768667266 0.9910634890348149
0.8829944043693487 0.9417663417278206 0.9895091710839878
0.5492329910064200 0.2743955192780376 0.0008146708116001
0.2162521063611820 0.1083256365499139 0.9900049973443643
0.0493399347779757 0.9413348556119416 0.9888976426331624
0.7156845645005845 0.2743033770178300 0.0002648086041219
0.3827698844078355 0.1078757273223090 0.9909227384624586
0.2160486136383994 0.9416472040658377 0.9890621123885950
0.8826697879801984 0.7749331092323928 0.9921058932215203
0.5494305412863056 0.1081878123699980 0.9928312854922154
0.3831018626776057 0.9416780674233109 0.9901941183625809
0.0496768286803544 0.7751625390022099 0.9928607150396260
0.7164384696244781 0.1083593923048838 0.9931297986558434
0.5495281997036585 0.9413785667655645 0.9909324873337937
0.2160407386773571 0.7747157732495680 0.9930513903618774
0.7162497966166796 0.9417311568346278 0.9904839777081733
0.3827233850849439 0.7749086305486017 0.9934233772652519
0.5497651420557688 0.7752059829096893 0.9936705375532995
0.7163583226732300 0.7750878095831411 0.9930317190006619
0.9940869876443870 0.6636591270495202 0.9979808788046753
0.1605510963916075 0.6637614260073263 0.9965608531551027
0.3269720449927291 0.6633118321725391 0.9969992726563532
0.9941605960758366 0.4969534560430660 0.0013307268253087
0.1607483428156158 0.4970329139950707 0.9999483739425798
0.6611576557129482 0.6643370135436939 0.9990864598835429
0.3266609967211807 0.4975008931663538 0.0052368159199361
0.9942368683838581 0.3306017570426647 0.9986080214875201
0.1603706633094717 0.3301928118369091 0.9992657226743589
0.4940891424874820 0.4986862383559370 0.0113775988933398
0.8272241869598744 0.6641742149185674 0.9982876789324919
0.6625408023215726 0.4983936713696906 0.0192278685612379
0.9941303022133923 0.1635145201029786 0.9933292945380643
0.3264288225577872 0.3289603065711617 0.0019130550754767
0.1607232559722149 0.1634256303785477 0.9922031782600982
0.4933211245669327 0.3290444550725908 0.0048466824867575

0.8288352655765650 0.4975922268055299 0.0093425748252045
0.3271824526155740 0.1634712745457563 0.9915514469808855
0.6598076303460942 0.3281905184754166 0.0048986461549063
0.9938192971114574 0.9967988679122897 0.9892021151302117
0.4938209768057007 0.1631742610530011 0.9940466138678801
0.1607496935681922 0.9967324306297272 0.9886902200745453
0.8277754891479532 0.3302456892886667 0.0015324221730779
0.9940847010544118 0.8303563530401803 0.9906691623729884
0.6605374193194606 0.1634106824054982 0.9950778955438295
0.3274648099380926 0.9970222863577050 0.9894397419752039
0.1603990926834271 0.8301466311235330 0.9911664877855770
0.8271384555411103 0.1635272084231616 0.9944057640051867
0.4936944057123895 0.9967387975530002 0.9907422068485801
0.3272742824585535 0.8299120966384308 0.9917781284889813
0.6606214779708068 0.9967629668461484 0.9910585314763978
0.4939470607453771 0.8303474846408889 0.9922565296250241
0.8273419129321269 0.9970483119425353 0.9903163552850458
0.6604672604275379 0.8302478852656350 0.9918023012046007
0.8273494214263764 0.8302874699091447 0.9908421024261216
0.4943282224437695 0.6639171477332155 0.9984708597783936
0.7346244875181327 0.4966773782314320 0.1259897075224217
0.4743496847088977 0.4471463044331117 0.1235474884006180
0.4166427282438363 0.4154863638441810 0.2594240523394760
0.5938908001702501 0.5792092232261510 0.2380375472785516
0.5806635513520249 0.4032540148605506 0.2174302001004804
0.7607230855721596 0.5687652477728093 0.2561578083857654

PT6(no-D)-XLIX – ΔE = 0.90 eV

1.00000000000000
14.7772044699999991 0.0000000000000000 0.0000000000000000
-7.3886022400000000 12.7974344700000007 0.0000000000000000
0.0000000000000000 0.0000000000000000 18.0009597799999987
C Pt
72 6
Direct
0.8852127815116262 0.6058647416694569 0.9999779963727349
0.0514191442251217 0.6052562718913137 0.9955114837228649
0.2176615466025851 0.6054161235627262 0.9957703376821101
0.8847022947639331 0.4386338226856523 0.9974716384984461
0.0517257081880516 0.4390254250963395 0.9948005767229304
0.3845865095430980 0.6062726750515992 0.0032054600946694
0.5521073839369066 0.6074949141779058 0.0182687260436296
0.2182212766929368 0.4387963005037463 0.9979467118518173
0.8849891298756276 0.2719840179998201 0.9945782911459347
0.0513601832263433 0.2721308682791488 0.9947959568270903
0.3826857875955696 0.4380743001477114 0.0094581096449105
0.7192011899867765 0.6073377638850630 0.0081607416442679
0.5513118200800307 0.4372711389744026 0.0202823511557142
0.2181093938577590 0.2723748944973678 0.9990102403007270
0.8849366520848605 0.1055988328087807 0.9939789857292709
0.7192940438161486 0.4382951394268417 0.0075011548603428
0.3844402213283615 0.2716177537270994 0.0050100571878957
0.0515466107482396 0.1054826309996599 0.9947574372197039
0.8847053317922047 0.9389530276812863 0.9953048473559107
0.5511843200033582 0.2711547395884253 0.0032254874491642
0.2178230551875728 0.1055384256280831 0.9967549005714673
0.0516639560279231 0.9391802008809549 0.9946146016747974
0.7184603221704435 0.2722976256618637 0.9976593289833460
0.3849215486019730 0.1057525935334311 0.9973735937816599
0.2182160282503602 0.9389510083986039 0.9946173395192233
0.8850507419773948 0.7724370065007236 0.9979733305678593
0.5518301492017841 0.1053661524449581 0.9956104240682819
0.3846631647825163 0.9390075193905005 0.9946119771162003
0.0511896543815666 0.7722957269851847 0.9952405433717999
0.7179166270729809 0.1052405110479953 0.9944767597817190
0.5516862328973957 0.9392368889002327 0.9952466125255839
0.2182513859895110 0.7725404509378819 0.9945295480682788
0.7180685611811128 0.9383610035914671 0.9961272090136859
0.3848948595033903 0.7723699920920097 0.9965424243534144
0.5510690248552663 0.7726149072185322 0.0011303675322836
0.7187381726027837 0.7726687711026443 0.0015004497838405
0.9954375245659079 0.6609319563569400 0.9965876899943709
0.1624602076634076 0.6607950524057884 0.9948688600125735
0.3295663014962855 0.6613140244116025 0.9978694253514249
0.9963228649440694 0.4945694491507595 0.9955482666608830
0.1624344936793989 0.4945804079429976 0.9956874195545851
0.6642793413363464 0.6621220941004893 0.0085169725785050
0.3282827530405612 0.4938043792921860 0.0033109544672527
0.9959937213570953 0.3274187290462862 0.9944827019478879
0.1627152249993600 0.3277020871314420 0.9970595806407729
0.4938629926483671 0.4941317712436160 0.0219160269271086
0.8296065075998200 0.6616212583191867 0.0015432211462283
0.6641523770354993 0.4949072423236487 0.0170559383929501
0.9958866618346960 0.1609693300749910 0.9942999490547066
0.3286002071229817 0.3274625892834351 0.0045940249991503
0.1625044724391742 0.1607286362700862 0.9966295381293548
0.4952649454914848 0.3257902966796564 0.0098110783132555
0.8296689893454143 0.4940206881556648 0.0014615465674979
0.3294889803750962 0.1612230372158123 0.9994021639474013
0.6632642459997697 0.3272374805033280 0.0029773801923483
0.9962584685870510 0.9944684459506732 0.9945464025808164
0.4960020176311630 0.1610857042692189 0.9980143213207455
0.1626167873223849 0.9944971454869744 0.9949291268909661
0.8293725414779800 0.3276655893338614 0.9964684713652900
0.9960650329118437 0.8275588976970596 0.9954696446919797
0.6624971194441187 0.1604395048927643 0.9955256877547072
0.3293442143958316 0.9944568206630038 0.9950700706638997
0.1627964559529858 0.8279141308102496 0.9943526118296617
0.8295182347457128 0.1609714236694160 0.9941553469254529
0.4961484050244991 0.9945558911455876 0.9947201401570069
0.3291186480358945 0.8279430288184315 0.9947724287109381
0.6623160881347800 0.9942262074355455 0.9950572117635588
0.4956495354925323 0.8276194758976771 0.9972438923022935
0.8292987515799055 0.9939692013098949 0.9948929301405727
0.6628578522414799 0.8280486278370347 0.9996553558350385
0.8294683012863473 0.8279024495045917 0.9979027293898568

```
0.4953849945682123 0.6622945679532037 0.0075473747289934
0.4488350089215984 0.4707947799052121 0.2628546667180700
0.5698773652978204 0.6671221253429707 0.2538000579269521
0.6074919544516888 0.4443607950507840 0.1317497676407484
0.6910853497373779 0.5939360362727371 0.2347309083684195
0.5139233081727284 0.5559698118816385 0.1338273787581912
0.5731458793287914 0.4009295203236505 0.2687015001775279
```

PT6(no-D)-L – ΔE = 0.90 eV

```
1.00000000000000
14.7772044699999991 0.0000000000000000 0.0000000000000000
-7.3886022400000000 12.7974344700000007 0.0000000000000000
0.0000000000000000 0.0000000000000000 18.0009597799999987
C Pt
72 6
Direct
0.8923376549842459 0.6125702297253568 0.9948224675322948
0.0585982251470369 0.6123743419923713 0.9938754286439888
0.2246924134024795 0.6127307404168647 0.0010679020320410
0.8922224187122438 0.4458393113075658 0.9928651920742979
0.0583957982171128 0.4456632104232128 0.9944950556275671
0.3932928245584293 0.6143656958408599 0.0126440918154884
0.5604330048535928 0.6143663894204110 0.0073101514018745
0.2232972852836781 0.4454375425243171 0.0069739079816884
0.8919363150599864 0.2791038989915648 0.9908494026134775
0.0584045656232632 0.2792017268873437 0.9965462571878163
0.3906440525203365 0.4451377599838935 0.0263545775709275
0.7265598892169720 0.6133465575778558 0.0008730015779221
0.5601488207701024 0.4453829790917112 0.0150542855281444
0.2236536026965013 0.2778687323955253 0.0084983866342725
0.8920581861884997 0.1125732396648544 0.9901647308693811
0.7261302205146620 0.4457440444525531 0.9996438936766765
0.3924891419665997 0.2776875282759477 0.0064234458805643
0.0584436176165752 0.1124197597974685 0.9950308668053509
0.8921348715290307 0.9459525707767753 0.9901112232584879
0.5588101507263303 0.2789819028987353 0.9981696385175880
0.2253836466958603 0.1124567709987900 0.9975899713013163
0.0588834912829483 0.9460847013736355 0.9923747584833933
0.7253587749895587 0.2792766138590466 0.9921792815759147
0.3923565872281998 0.1125022746826261 0.9946785571737422
0.2257642997551841 0.9461270398642938 0.9931081824755097
0.8923546767139143 0.7794922352875133 0.9924008565335072
0.5588449617863489 0.1124851659364410 0.9913377329914397
0.3923873312897683 0.9462382132855964 0.9928563510345256
0.0590151280862301 0.7794502463651867 0.9927367178081639
0.7253120406339306 0.1124836700021703 0.9889825381490951
0.5586634499076482 0.9455998935729895 0.9911624339262204
0.2256248521977184 0.7794912682780932 0.9952335968723034
0.7255295120355498 0.9458878349807449 0.9893721879757251
0.3924357661284930 0.7801647407590409 0.9991952149821515
0.5594467527984506 0.7795002106143585 0.9960709662874834
0.7256744105594706 0.7792624374549177 0.9930834063678162
0.0033543748916927 0.6681754668393012 0.9933141005625927
0.1699453713012247 0.6680363743801294 0.9962120673117809
0.3360137590178738 0.6691693869114843 0.0065381496615373
0.0031371654106067 0.5015780807414174 0.9931190119360878
0.1688665963269713 0.5008960073840925 0.9997892173680540
0.6707266640104876 0.6689391020988467 0.9993534498409318
0.3344639384828199 0.5022772586198414 0.0199972897533200
0.0029243846312639 0.3347411384990551 0.9934371510201530
0.1688263301930277 0.3347757291159255 0.0043100132428151
0.5058664513558568 0.5037444122934147 0.0223095877573343
0.8368266864338878 0.6683978714147543 0.9955329091037655
0.6716874584050672 0.5021452534940707 0.0067112452391171
0.0029985972960418 0.1680376889156534 0.9937698850221039
0.3352529261504884 0.3323275565393686 0.0173166626188319
0.1693502823007691 0.1678395330346447 0.0003196198645270
0.5031251778166990 0.3331409142812944 0.0058337928932701
0.8369306966297714 0.5016740230997243 0.9954758003944022
0.3366677122681736 0.1678387439229141 0.9988677531815995
0.6702542260128439 0.3348671769574807 0.9965591038198909
0.0033000729004442 0.0015031984580602 0.9919640248217689
0.5032050909345784 0.1679105415722120 0.9942125039909939
0.1701698357031418 0.0018938617106912 0.9938304724470655
0.8365822913960272 0.3348803960719025 0.9916148507815166
0.0036312345692338 0.8350467868542850 0.9919257076166090
0.6698808665539744 0.1681010254880562 0.9903689442962715
0.3366308486464362 0.0015688539046863 0.9929860069809848
0.1701455395166889 0.8349495662270527 0.9932107469026761
0.8364962319539728 0.1680792575044876 0.9895725925578844
0.5031710734330375 0.0014840591714105 0.9914469598864883
0.3365992869995438 0.8351207317785310 0.9955979717151493
0.6698410302559097 0.0012914841296521 0.9892504813160343
0.5031569301060799 0.8349937067565847 0.9950404805410784
0.8366110608945974 0.0013746193677235 0.9892805907677271
0.6701574254700020 0.8349026013160383 0.9920470474322940
0.8369158072925913 0.8348746508710647 0.9911408127464156
0.5056055554419103 0.6696486591829967 0.0042640149153144
0.4046464217247063 0.6713620225556340 0.2537780162271943
0.3607735470509894 0.5911016213868194 0.1272556857069418
0.3519370229649468 0.3754876340390396 0.1341436471702337
0.4508979233151607 0.5368011033521967 0.2285492188608202
0.5495435467786791 0.4716799439639345 0.1336261297495582
0.4582723703228098 0.3749323806916038 0.2552334806425094
```
